# *Helicobacter pylori* triggers gastric mucosal remodeling toward a fetal-like transcriptional program via stromal IL-1β signaling

**DOI:** 10.1038/s41467-026-77520-1

**Published:** 2026-09-05

**Authors:** Giulia Beccaceci, Stefanie Müllerke, Hilmar Berger, Christian Täger, Ronja Möbius, Anne-Sophie Fischer, Kimberly Hartl, Jonas Wizenty, Hans-Joachim Mollenkopf, Michael Naumann, Manqiang Lin, Michael Sigal

**Affiliations:** 1 Department of Hepatology and Gastroenterology, Charité – Universitätsmedizin Berlin, Berlin, Germany; 2 Berlin Institute for Medical Systems Biology (BIMSB), Max Delbrück Center for Molecular Medicine in the Helmholtz Association (MDC), Berlin, Germany; 3 Institute of Experimental Internal Medicine, Magdeburg, Germany; 4 Berlin Institute of Health at Charité – Universitätsmedizin Berlin, BIH Biomedical Innovation Academy, BIH Charité Clinician Scientist Program, Berlin, Germany; 5 Max Planck Institute for Infection Biology, Berlin, Germany; 6 Cluster of Excellence ImmunoPreCept, Charité - Universitätsmedizin Berlin, Berlin, Germany

**Keywords:** Adult stem cells, Gastric cancer

## Abstract

In the gastrointestinal tract, Wnt and BMP signals control *Lgr5*⁺ stem cell activity during homeostasis, whereas injury elicits an *Lgr5*-independent, fetal-like regenerative program driven by YAP. *Helicobacter pylori* (*H. pylori*) infection activates YAP, but whether fetal-like reprogramming contributes to gastric pathology, and what drives it, has remained unclear. Here we show that *H. pylori*-induced gland hyperplasia is accompanied by YAP-dependent fetal-like transcriptional response and loss of epithelial BMP signaling. Epithelial BMP inhibition alone is sufficient to induce this program in vivo, through an epithelial–immune–stromal cascade: BMP-deficient epithelial cells secrete chemokines that recruit IL-1β-producing immune cells, and IL-1β drives enrichment of pro-regenerative fibroblasts producing prostaglandin E2. In gastric epithelial–stromal assembloids, IL-1β elicits stromal prostaglandin E2 production and subsequent epithelial YAP activation. Stromal deletion of the IL-1 receptor abrogates *H. pylori*-driven reprogramming and pathology. These data define a cascade that converts BMP loss into a fetal-like regenerative state and shapes *H. pylori*-associated gastric disease.

## Abstract (web summary)

During tissue homeostasis, gastric epithelial maintenance is governed by Wnt and BMP signaling, whereas injury induces a YAP-driven fetal-like regenerative program. Here, Beccaceci et al. show that *H. pylori* infection reprograms the gastric mucosa toward a fetal-like state through BMP deficiency and IL-1β-dependent immune-stromal-epithelial crosstalk.

## Introduction

*Helicobacter pylori* (*H. pylori*) is a pathogenic bacterium that causes gastritis and has been causally linked to gastric carcinogenesis^1,2^. Infection leads to chronic inflammation and premalignant pathology, such as hyperplasia and metaplasia*. H. pylori* colonizes the gastric mucosa via attachment to epithelial intercellular junctions^3,4^. Upon attachment, the bacteria exploit a Type 4 Secretion System (T4SS)^5^ to translocate their main virulence factor, CagA, into host cells to hijack cellular signaling^6^. The presence of the T4SS also induces NF-κB signaling in gastric epithelial cells^7^, which has been linked to the release of ADP-heptose (ADP-H), a small metabolic precursor of bacterial lipopolysaccharide (LPS)^8–10^. Although *H. pylori* preferentially attaches to short-lived differentiated mucous pit cells^11^, a subset of bacteria migrates towards the gland base and makes direct contact with long-lived stem and progenitor cells^12,13^. Several studies have demonstrated that it is particularly the interaction with the gland base cells that initiates inflammatory responses via the ADP-H/NF-κB axis, while differentiated pit cells do not show these responses^14–16^.

In addition to direct cell-intrinsic effects, antrum gland-associated *H. pylori* interfere with the complex multicellular network that controls epithelial stem cell fate. For example, infection enhances the expression of stromal R-spondin 3 (*Rspo3*), which increases Wnt signaling at the gland base^17–19^. At the same time, BMP signaling is suppressed in the epithelium through increased expression of BMP inhibitors, such as Gremlin 1, and a loss of BMP ligands secreted by the stroma upon infection^20^. BMP ligands produced by stromal cells surrounding the pit compartment usually promote terminal differentiation, while inhibition of BMP signaling is important for maintaining the *Lgr5*+ stem cells^21^.

Perturbations of epithelial homeostasis in the gastrointestinal tract can cause widespread tissue remodeling. While Wnt signaling is crucial to regulate proliferation of stem/progenitor cells in homeostasis^22^, various studies in the intestine have revealed a stereotypic reprogramming of the epithelium into a highly proliferative regenerative state that resembles the fetal intestinal epithelium^23,24^. This fetal-like regenerative epithelium is characterized by the induction of unique signaling pathways such as Hippo/yes associated protein 1 (YAP), while *Lgr5*+ cell signature genes are repressed^25^. Similarly, gastric premalignant pathology has also been linked to increased YAP signaling. Furthermore, overexpression of *Rspo3* causes activation of fetal-like genes in the gastric corpus of *H. pylori*-infected mice, while uninfected mice do not display these responses^19,26^. However, the mechanisms by which fetal-like reprogramming is regulated are poorly understood in both the intestine and the stomach.

Here, we demonstrate that *H. pylori* infection does not simply cause an expansion of homeostatic stem cells, but that gland hyperplasia involves fetal-like transcriptional reprogramming. We reveal that Inhibition of BMP signaling in vivo is sufficient to induce these responses. Deprived of BMP-driven suppression, the antral epithelium upregulates proinflammatory chemokines in response to PAMP stimulation, facilitating immune cell infiltration into the gastric mucosa. These immune cells, in turn, secrete IL1-β, which promotes activation of the cyclooxygenase2 (COX2)/prostaglandin E2 (PGE2) pathway in fibroblasts, ultimately contributing to a fetal-like transcriptional response in the adjacent epithelium. Accordingly, depletion of the IL-1 receptor in stromal cells prevents epithelial transcriptional changes and gland hyperplasia in response to *H. pylori* infection.

## Results

### BMP deficiency induces a fetal-like transcriptional response

We have previously described that *H. pylori* causes gland hyperplasia in the gastric antrum, the region where the majority of cancerous lesions occur, which we linked to downregulation of BMP signaling in the epithelium^20^. We now aimed to explore the molecular consequences of BMP downregulation in more detail. For this, we exploited the *Axin2*^*CreErt2*^*Bmpr1a*^*fl/fl*^ conditional knockout (KO) mouse model, which enables BMP signaling in epithelial cells to be blocked via depletion of the BMP receptor 1a (*Bmpr1a*) in *Axin2*+ progenitor cells^20^. While BMP signaling activity is restricted to the pit of the gland, *Bmpr1a* is normally expressed throughout the gland. Using in situ hybridization (ISH), we confirmed an almost complete loss of *Bmpr1a* expression in the epithelium two months after tamoxifen administration, while expression in the stroma was unaffected (Supplementary Fig. 1a). Gene expression analysis using qPCR additionally confirmed downregulation of *Bmpr1a* and of the BMP signaling target gene *Id1* in *Bmpr1a* KO antral tissue (Supplementary Fig. 1b).

**Fig. 1:**
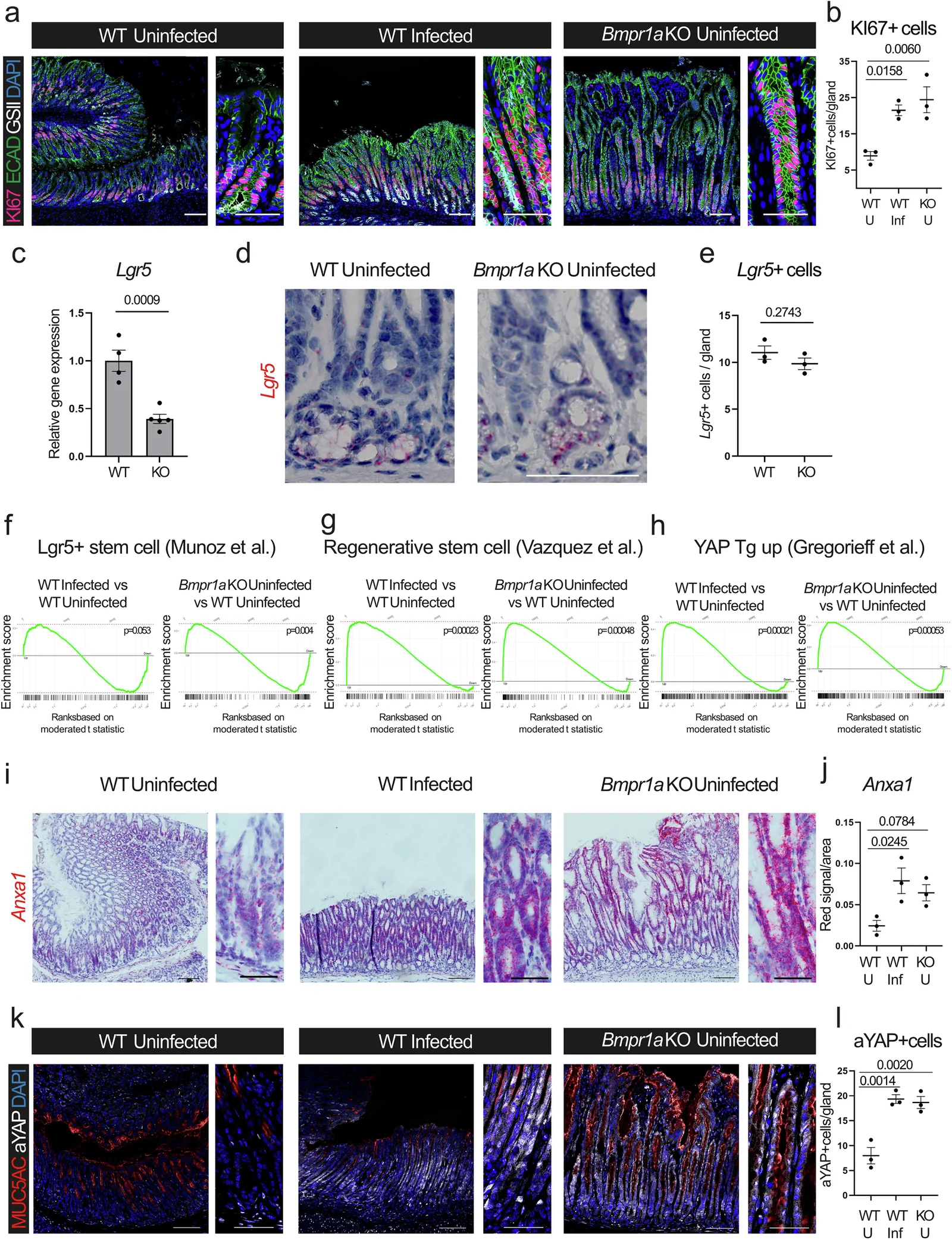
Downregulation of BMP signaling induces the epithelial fetal-like regenerative transcriptional state. **a**, **b** Confocal microscopy images and quantification of KI67, E-cadherin (ECAD), GSII and DAPI co-staining in gastric antral tissue from uninfected, infected WT, and uninfected *Bmpr1a* KO mice (*n* = 3 mice per group). Scale bars: 100 µm, inserts: 50 µm. **c** mRNA expression of *Lgr5* in antral gastric tissue of uninfected *Bmpr1a* KO (*n* = 5) and WT (*n* = 4) mice. **d**, **e** In situ hybridization (ISH) images and quantification of *Lgr5* in uninfected WT, infected WT, and uninfected *Bmpr1a* KO antral tissues (*n* = 3 mice per group). Scale bar: 50 µm. Gene set enrichment analyses (GSEA) of microarray data comparing antral tissue from infected WT vs uninfected WT mice, as well as from uninfected *Bmpr1a* KO vs WT mice, for Lgr5+ stem cell gene signature (**f**), regenerative stem cell signature (**g**), and YAP target gene signature (YAP Tg up) (**h**) (*n* = 2 mice per group). **i**, **j** ISH images and quantification of *Anxa1* in sections from uninfected WT, infected WT, and uninfected *Bmpr1a* KO mice (*n* = 3 mice per group). Scale bars: 100 µm, inserts: 50 µm. **k**, **l** Confocal microscopy images and quantification of MUC5AC and active YAP (aYAP) in antral gastric tissue from uninfected WT (WT U), infected WT (WT inf), and uninfected *Bmpr1a* KO (KO U) mice (*n* = 3 mice per group). Scale bars: 100 µm, inserts: 50 µm. Data are presented as mean ± SEM. Statistical analyses were performed using Student’s *t*-test (two-tailed) for **c** and **e**; using one-way ANOVA, followed by Tukey’s multiple comparisons test for **b**, **j**, **l**; using unadjusted *p*-values of permutation test provided by R package fgsea for (**f**–**h**). Images are representative of at least three biological replicates. Source data are provided as a Source data file.

We confirmed our previous observation that *Bmpr1a* KO alone is sufficient to cause gland hyperplasia, as evidenced by expansion of the proliferative KI67+ compartment from the base to the pit of the gland, which was also observed in WT epithelium after infection with PMSS1 *H. pylori* (Fig. 1a, b). WT mice were infected for 2 months, a timepoint where we expect to observe gastritis and early premalignant lesions, mimicking the pathology associated with *H. pylori* in human carriers. Successful infection was confirmed via colony-forming unit (CFU) analysis (Supplementary Fig. 1c). We observed that *Bmpr1a* KO glands were higher than hyperplastic glands in infected WT mice (Fig. 1a). This may be due to the fact that *H. pylori* infection causes merely a reduction of BMP signaling rather than an almost complete loss of the pathway. To explore the mechanism that causes increased epithelial proliferation upon *H. pylori* infection and understand the contribution of reduced BMP signaling to this process, we carried out an unbiased transcriptome analysis, comparing *H. pylori*-infected to uninfected WT mice, as well as uninfected *Bmpr1a* KO (*Axin2*^*CreErt2*^*Bmpr1a*^*fl/fl*^) to uninfected *Bmpr1a* WT (*Bmpr1a*^*fl/fl*^) mice. Although stem/progenitor cell proliferation throughout the gastrointestinal tract is known to be promoted by Wnt/Lgr5-driven signaling, surprisingly, gene expression analysis revealed that *Lgr5* expression was instead downregulated in *Bmpr1a* KO tissue (Fig. 1c). To explore whether the *Lgr5*+ cell compartment was indeed altered in *Bmpr1a* KO mice, we performed ISH for *Lgr5* and observed that *Lgr5* expression remained restricted to the gland base, similar to uninfected WT mice (Fig. 1e, Supplementary Fig. 1d), while proliferation was observed in a large section of the gland reaching from the base to the pit region (see Fig. 1a). Furthermore, we performed transcriptome analysis of gastric tissues and Gene Set Enrichment Analysis (GSEA) failed to show enrichment of the *Lgr5*+ stem cell signature following either infection or *Bmpr1a* KO (Fig. 1f). These data indicate that the increased epithelial proliferation observed upon infection or *Bmpr1a* KO is not driven by an expansion of homestatic *Lgr5*+ stem cells.

Another signaling pathway known to be involved in cell proliferation is YAP signaling^27,28^. In the intestine, YAP signaling is activated upon injury and mediates the fetal-like regenerative transcriptional state^29^. We asked whether this state is also induced upon *H. pylori* infection, and indeed confirmed upregulation of a fetal-like epithelial regenerative stem cell gene signature by GSEA (Fig. 1f). Notably, uninfected *Bmpr1a* KO mice also showed a significant upregulation of this signature (Fig. 1g). Moreover, transcriptome analysis showed enrichment of YAP target gene signatures (Fig. 1h, and Supplementary Fig. 1e). Consistently, ISH revealed increased expression of the fetal epithelial marker *Anxa1* in both infected WT and uninfected *Bmpr1a* KO mice (Fig. 1i, j). While the epithelium of uninfected WT mice was characterized by low numbers of cells with YAP in its active nuclear form (aYAP), *H. pylori*-infected WT and uninfected *Bmpr1a* KO tissue contained a significantly higher number of aYAP+ cells (Fig. 1k-l). Interestingly, the *Anxa1* + aYAP+ compartment expanded beyond the base, where cells are exposed to high levels of Wnt ligands, towards the pit region.

To further characterize gastric epithelial cells in the context of BMP signaling deficiency and infection, we performed single-cell RNA sequencing (scRNA-seq) on epithelial cells isolated from the murine stomach (Supplementary Fig. 2a). We identified distinct antral cell subsets, including stem cells, transit-amplifying (TA)/progenitor cells, pit cells, and surface mucous cells (Supplementary Fig. 2b–d and Supplementary Data 1). In addition, we captured corpus cell types (e.g., Parietal, Chief, TA corpus, and Pit corpus), as well as enteroendocrine cells (e.g., EEC Chga, EEC Sst, EEC-Pro/EC cell, EC-like cell, and G-cell) and tuft cells (Supplementary Fig. 2b and Supplementary Data 1). For the following analyses, we focused on the main epithelial cell types in the antrum. We observed an expansion of TA/progenitor cells and a reduction of pit cells upon *H. pylori* infection, whereas no significant alteration in the proportion of cell subsets was detected in *Bmpr1a* KO mice (Supplementary Fig. 2e, f). This suggests BMP deficiency leads to the expansion of multiple cell lineages without significantly altering their relative proportions.

Next, we analyzed transcriptional changes within antral epithelial subsets. Differential Expression Gene (DEG) analysis showed that all antral epithelial lineages underwent profound transcriptional alterations following *Bmpr1a* KO or infection (Supplementary Data 2). As expected, BMP target genes *Id1* and *Id2* were downregulated in *Bmpr1a* KO pit and surface mucous cells, while infected pit and surface mucous cells showed decreased expression of *Id2* (Supplementary Fig. 3a). Notably, GSEA revealed that the regenerative stem cell signature was enriched across all antral cell types, including stem, TA/progenitor, pit, and surface mucous cells, following *Bmpr1a* KO or infection (Supplementary Fig. 3b). Moreover, YAP target gene signatures were enriched in most antral cell types upon *Bmpr1a* KO or infection (Supplementary Fig. 3c, d). Consistently, the upregulation of regeneration-associated genes (e.g., *Ly6a*) was broadly observed in antral epithelial cells for both conditions (Supplementary Fig. 3e, f), while *Anxa1* was significantly upregulated only in *Bmpr1a* KO TA/progenitor, pit, and surface mucous cells (Supplementary Fig. 3e–g). In addition, YAP target gene *Ccn1* (also known as *Cyr61*) was upregulated in *Bmpr1a* KO surface mucous cells as well as infected pit cells, while another YAP target, *Ccn2* (also known as *Ctgf*), was increased in *Bmpr1a* KO TA/progenitor cells (Supplementary Fig. 3g). These data indicate that the regenerative transcriptional responses are not confined to a single cell type but are rather broad responses involving various gland cell lineages.

We also analyzed the *Lgr5*+ stem cell signature and found that it was either unchanged (e.g., infected stem, TA/progenitor, and pit cells; *Bmpr1a* KO TA/progenitor and pit cells) or downregulated (e.g., *Bmpr1a* KO stem) in most cell types upon *Bmpr1a* KO or infection (Supplementary Fig. 3h). Although *Lgr5*+ stem cell signature was enriched in *Bmpr1a* KO or infected surface mucous cells (Supplementary Fig. 3h), actual *Lgr5* expression was barely detected in this epithelial subset. Moreover, we did not observe significant alteration in *Lgr5* expression in any infected or *Bmpr1a* KO epithelial subsets (Supplementary Fig. 3i). Taken together, our data suggest that rather than a Wnt-driven homeostatic stem cell program, a YAP-associated regenerative stem cell state likely plays a key role in driving the glandular hyperplasia observed in *H. pylori*-infected and BMP-deficient tissues.

To explore how loss of BMP signaling leads to upregulation of YAP, we exploited the 3D organoid system, which allows culturing of pure primary gastric epithelial cells. The standard gastric organoid medium contains Noggin, a BMP signaling inhibitor. We therefore tested whether removal of Noggin alone would affect epithelial cell gene expression, comparing cells grown in 100% Wnt, R-spondin and Noggin (100%WRN) to cells grown in Noggin deprived medium (0%N) (Supplementary Fig. 4a). Removal of Noggin was not sufficient to upregulate expression levels of pit cell markers (*Muc5ac, Gkn1* and *Gkn2*), despite inducing the expression of the BMP signaling target *Id1* and partial downregulation of *Lgr5* (Supplementary Fig. 4b, c). In this condition, the expression of YAP target genes (*Ctgf, Cyr61* and *Ankrd1*) was not significantly altered (Supplementary Fig. 4d).

**Fig. 2:**
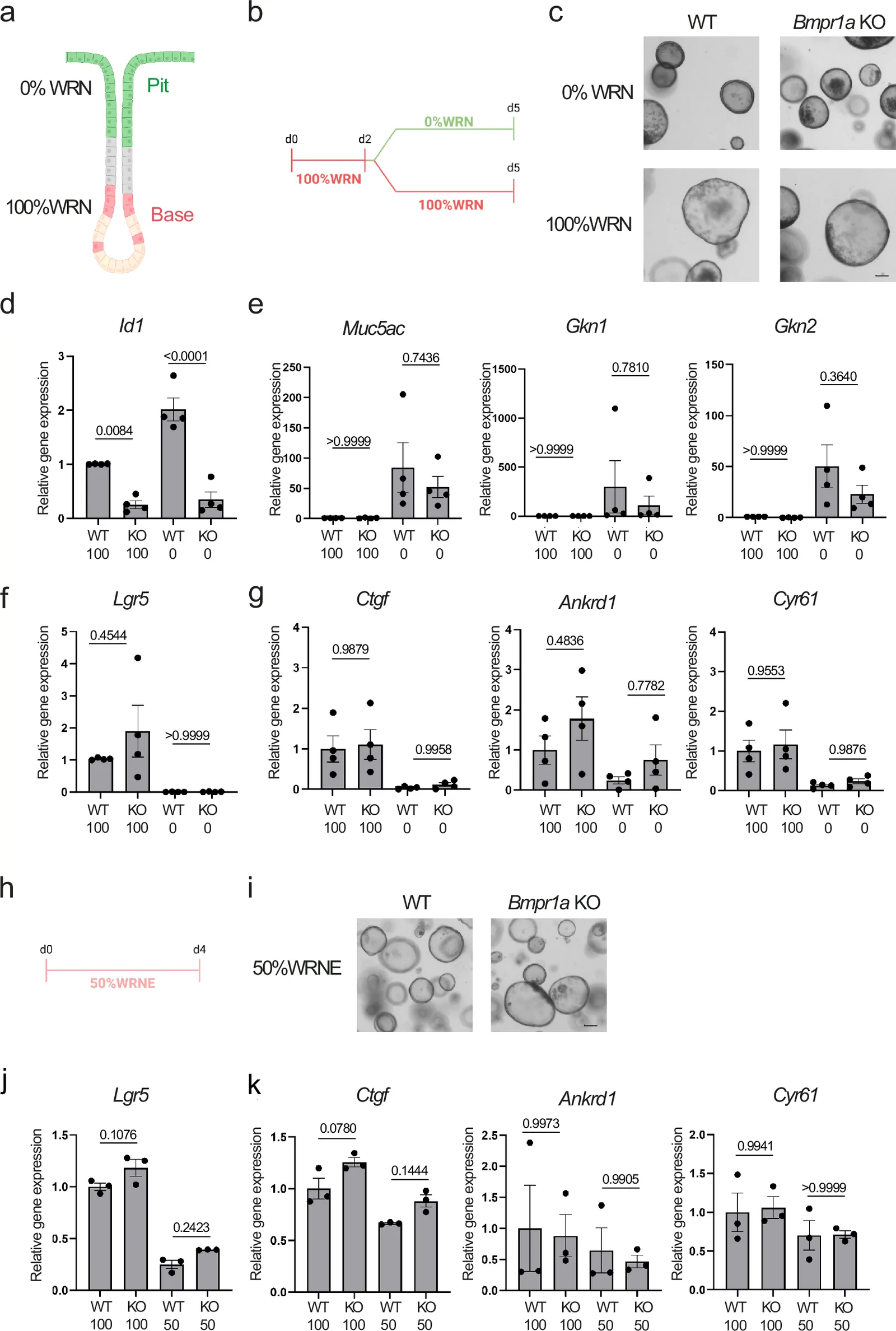
Blocking BMP signaling in vitro does not affect YAP target gene expression. **a** Schematic of the epithelial cell compartment in antral gastric glands and the concentration of Wnt (W), R spondin-1 (R) and Noggin (N) used in in vitro organoids to mimic the base (100%WRN) and the pit region (0%WRN). The image was created in BioRender. Müllerke, S. (2026) https://BioRender.com/tlbwkqw. **b** Organoid treatment schematic. The image was created in BioRender. Müllerke, S. (2026) https://BioRender.com/tlbwkqw. **c** Brightfield image of WT and *Bmpr1a* KO organoids grown in 100%WRN medium for 5 days or grown in 100%WRN medium for 2 days followed by 3 days in 0%WRN medium. Scalebar: 100 µm. mRNA expression of the BMP target gene *Id1* (**d**), differentiated pit cell markers *Muc5ac, Gkn1* and *Gkn2* (**e**), the stem cell marker *Lgr5* (**f**), and YAP target genes *Ctgf, Ankrd1* and *Cyr61* (**g**) in WT and *Bmpr1a* KO organoids grown in 100%WRN or 0%WRN medium (*n* = 4 biological replicates per group). **h** Organoid treatment schematic. The image was created in BioRender. Müllerke, S. (2026) https://BioRender.com/tlbwkqw. **i** Brightfield images of WT and *Bmpr1a* KO antral gastric organoids grown for 4 days in 50% WRNE medium. Scale bar: 100 µm. mRNA expression of *Lgr5* (**j**) and the YAP target genes *Ctgf, Ankrd1* and *Cyr61* (**k**) in WT and *Bmpr1a* KO organoids grown in 100%WRNE or 50%WRNE medium (*n* = 3 biological replicates per group). Data are presented as mean ± SEM. Statistical analyses were performed using one-way ANOVA, followed by Tukey’s multiple comparisons test for **d**–**g** and **j**, **k**. Images are representative of at least three biological replicates. Source data are provided as a Source data file.

Modulating the medium composition allowed us to mimic the high-Wnt/high BMP antagonist signaling at the gland base, which enriches for stem/progenitor cells (100% of the standard concentration of Wnt, R-spondin and Noggin, labeled as 100%WRN), while complete removal of Wnt, R-spondin and Noggin (labeled as 0%WRN) instead drives differentiation of stem/progenitor cells into pit cells (Fig. 2a). We generated *Bmpr1a* KO organoid lines by treating cells with tamoxifen in vitro. KO organoids showed similar proliferation levels (Supplementary Fig. 4e), while gene expression analysis confirmed that the expression levels of *Bmpr1a* and of its target gene *Id1* were significantly downregulated, compared to WT organoids (Supplementary Fig. 4f). When we cultured organoids either in 100%WRN or 0%WRN (Fig. 2b), we did not observe morphological differences between WT and *Bmpr1a* KO organoids (Fig. 2c). Removal of growth factors normally causes differentiation of cells into pit cells and upregulation of BMP signaling. Indeed, WT cells upregulated expression of the BMP target *Id1* when grown in 0%WRN medium, while *Bmpr1a* KO organoids failed to do so (Fig. 2d). Despite lacking the ability to upregulate BMP signaling, KO organoids nonetheless upregulated pit cell markers (*Muc5ac, Gkn1* and *Gkn2*) upon growth factor withdrawal (Fig. 2e). Moreover, both WT and KO organoids upregulated the expression of the pit-associated BMP ligand *Bmp2* at comparable levels when grown in 0%WRN medium, while *Bmp7*, which is mostly expressed in gland base cells^20^, was higher in KO organoids grown in 100%WRN and was downregulated both in WT and *Bmpr1a* KO organoids upon growth factors withdrawal (Supplementary Fig. 4g). scRNA-seq of gastric epithelium confirmed that *Bmp2* and *Bmp7* are mainly expressed by pit/surface mucous and stem/TA/progenitor cells, respectively, while other BMP ligands such as *Bmp4* and *Bmp5* could not be detected (Supplementary Fig. 4h, i). Additionally, in the 0%WRN condition, both WT and KO organoids lost the canonical stem cell marker *Lgr5* (Fig. 2f) and downregulated YAP target genes (Fig. 2g).

Next, we grew organoids in an intermediate condition, containing half the standard concentration of Wnt, R-spondin, Noggin and EGF (50%WRNE) (Fig. 2h). In this condition, both WT and *Bmpr1a* KO cells still retained organoid-forming ability (Fig. 2i), while showing reduced expression of *Lgr5* (Fig. 2j). However, we did not observe differences in YAP target gene expression in *Bmpr1a* KO organoids compared to WT, with both lines showing a partial reduction of YAP target expression levels (Fig. 2k). Thus, depletion of BMP signaling alone in organoids is not sufficient to induce alterations in the fetal-like regenerative state genes.

### BMP signaling controls epithelial proinflammatory responses

**Fig. 3:**
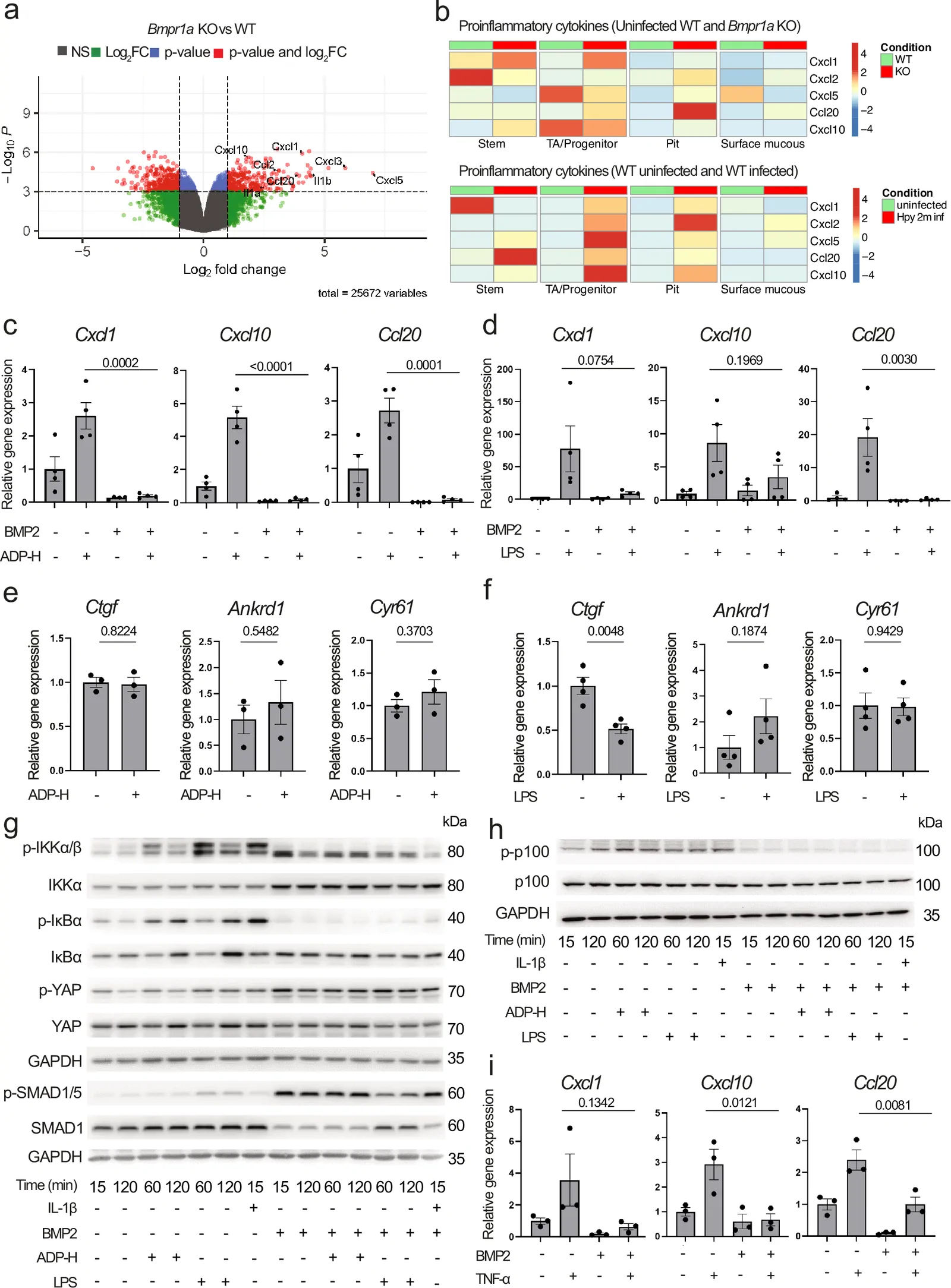
BMP signaling regulates the proinflammatory capacity of epithelial cells. **a** Volcano plot comparing microarray data of uninfected *Bmpr1a* KO vs WT antral tissue. Proinflammatory cytokine genes are highlighted. **b** Heatmap of single-cell RNA sequencing (scRNA-seq) showing scaled average expression of pro-inflammatory cytokines in gastric epithelial cell populations (*n* = 2 mice per group). mRNA expression of *Cxcl1*, *Cxcl10*, and *Ccl20* in organoids treated with BMP2 and/or ADP-heptose (ADP-H) (**c**), and with BMP2 and/or LPS (**d**) (*n* = 4 biological replicates per group). ADP-H/LPS treatment was performed for 2 h on the last day of culture. mRNA expression of *Ctgf*, *Ankrd1*, and *Cyr61* in organoids grown in standard medium and treated with ADP-H (*n* = 3 biological replicates per group) (**e**) or ADP-H (*n* = 4 biological replicates per group) (**f**) for 2 h. Western blot showing relative levels of p-IKKα/β_,_ IKKα, p-IκBα, IκBα, p-YAP, YAP, p-SMAD1/5, and SMAD1 (**g**) and relative levels of p-p100 and p100 (**h**) in organoids treated with BMP2 and/or ADP-H or LPS for the indicated time points (*n* = 2 biological replicates per group). IL-1β treatment was used as a positive control for NF-κB signaling activation. GAPDH was used as a loading control. The experiment was performed two times independently with similar results. **i** mRNA expression of *Cxcl1, Cxcl10 and Ccl20* in organoids treated with BMP2 and/or TNF-α (*n* = 3 biological replicates per group). TNF-α treatment was performed for 2 h. Data are presented as mean ± SEM. Statistical analyses were performed using one-way ANOVA, followed by Tukey’s multiple comparisons test for **c**–**f** and **i**. Data are presented as mean ± SEM. Statistical analyses were performed using one-way ANOVA, followed by Tukey’s multiple comparisons test for **c**–**f** and **i**; using unadjusted *p*-values derived from moderated *t*-test as provided by R package limma for (**a**). Source data are provided as a Source data file.

To identify the mechanism that drives epithelial reprogramming upon BMP loss in vivo, we explored transcriptional alterations in *Bmpr1a* KO mice via bulk microarray analysis. We found that they displayed a significant upregulation of various chemokines that are known targets of NF-κB signaling (Fig. 3a). As previous reports indicated that epithelial cells can express these genes^15,16^, we exploited our scRNAseq data to further explore which epithelial subpopulations upregulate proinflammatory chemokines, both in the *Bmpr1a* KO model and upon *H. pylori* infection (Fig. 3b). We observed that this upregulation occurred across diverse cell lineages. Consistently, the hallmark of inflammatory response was enriched in most cell types for both conditions (e.g., infected stem, TA/progenitor, pit, and surface mucous cells; *Bmpr1a* KO TA/progenitor, pit, and surface mucous cells) (Supplementary Fig. 4j).

To test whether epithelial cells that lack *Bmpr1a* show intrinsically higher NF-κB activity, we exploited *Bmpr1a* KO organoids. Gene expression analysis revealed that KO cells grown in the progenitor cell-enriched condition (100%WRN) had higher levels of the proinflammatory cytokines *Cxcl1*, *Cxcl2*, *Cxcl5*, and *Cxcl10* even in the absence of external proinflammatory triggers (Supplementary Fig. 5a); differentiated KO organoids (0%WRN) also displayed higher expression of *Ccl20* compared to WT organoids. To further investigate whether these alterations are directly driven by BMP signaling in the antral epithelium, we generated gastric organoids and either maintained them in standard full medium containing the BMP inhibitor Noggin (labeled as +BMPi) or treated them with recombinant BMP2 (+BMP2) to induce activation of BMP signaling. Upon BMP2 treatment, we removed the BMP inhibitor Noggin from the culture medium, as previously described^20^ (Supplementary Fig. 5b). BMP2 treatment inhibited organoid growth as evidenced by loss of KI67 expression and induced a shift from a progenitor cell phenotype to a differentiated MUC5AC+ cell type (Supplementary Fig. 5c). BMP signaling activation was validated by gene expression analysis, which showed upregulation of the BMP target gene *Id1* and a concomitant loss of the stem cell marker *Lgr5* (Supplementary Fig. 5d). The inflammatory capacity of epithelial cells with either active or inactive BMP signaling was then tested by exposing organoids to different pathogen-associated molecular patterns (PAMPs). Specifically, organoids were treated with either ADP-H to mimic responses to *H. pylori*, or with LPS from *Klebsiella pneumoniae*, to model a more general responsiveness to Gram-negative bacteria, either in the presence or absence of BMP2. While organoids grown in standard medium strongly upregulated the expression of proinflammatory cytokines upon treatment with ADP-H, this effect was completely inhibited in cells treated with BMP2 (Fig. 3c and Supplementary Fig. 5e). Treating organoids with LPS showed similar results in gene expression (Fig. 3d and Supplementary Fig. 5f). We then asked whether the exposure to PAMPs, beyond its effect on activating proinflammatory pathways, could additionally be involved in the activation of YAP signaling in gastric epithelial cells. Therefore, we measured the expression levels of YAP target genes upon ADP-H or LPS treatment in organoids grown in standard medium. However, treated organoids did not show significant upregulation of YAP target genes (Fig. 3e, f).

Next, we wanted to gain deeper mechanistic insights into the pro-inflammatory ability of epithelial cells in relation to their BMP signaling status. Western Blot analysis revealed that in the absence of BMP2, treatment with ADP-H or LPS induced phosphorylation of inhibitory kappaB kinase α/β (IKKα/β) and nuclear factor of kappa light polypeptide gene enhancer in B-cells inhibitor alpha (IκBα), which execute downstream activation of canonical NF-κB signaling. By contrast, BMP signaling activation via BMP2 inhibited the phosphorylation of IKKα/β and IκBα (Fig. 3g and Supplementary Fig. 5g). This suggests that this is a conserved mechanism that tightly regulates the epithelial response to PAMPs. We then assessed the phosphorylation levels of SMAD1/5, which is a crucial mediator of canonical BMP signaling, observing that p-SMAD1/5 was strongly upregulated upon BMP2 treatment (Fig. 3g and Supplementary Fig. 5g). Interestingly, we found that the epithelial baseline YAP activity was suppressed upon BMP2-driven BMP activation (Fig. 3g and Supplementary Fig. 5g), whereas epithelial BMP inactivation via *Bmpr1a* KO was not able to upregulate YAP in vitro (see Fig. 2g, k). Moreover, ADP-H and LPS treatments induced the phosphorylation of p100, which activates the alternative NF-κB pathway, while treatment with BMP2 inhibited this effect (Fig. 3h and Supplementary Fig. 5h).

We then wondered if the effect we observed was strictly linked to an epithelial response to PAMPs or if it could be generalized to other inflammatory triggers. Therefore, we repeated the experiment, this time treating organoids with the pro-inflammatory cytokine TNF-α and measuring gene expression changes by qPCR. Similarly, to what was observed for ADP-H and LPS, TNF-α induced upregulation of proinflammatory cytokines in organoids, whereas this effect was strongly inhibited by BMP2 (Fig. 3i and Supplementary Fig. 5i).

Furthermore, we analyzed the expression levels of different downstream mediators of the NF-κB pathway in response to inflammatory triggers and found that *A20*, *Traf6*, *Myd88*, and *Tifa* were inhibited in organoids upon exposure to BMP2 (Supplementary Fig. 6a–d), thus suggesting that epithelial cells with active BMP signaling are less responsive to inflammatory triggers as a result of reduced basal NF-κB pathway activity.

We then asked whether the effect we observed was due to a shift in cell lineage induced by BMP2 treatment or whether BMP signaling was the main driver of the epithelial response. To this end, we performed a short-term treatment (2 h) with BMP2 in organoids (Supplementary Fig. 6e). Gene expression analysis revealed that this was sufficient to induce BMP target genes such as *Id1*, while cell type-specific markers (*Lgr5* and *Muc5ac*) were not yet significantly affected (Supplementary Fig. 6f). However, when cells were simultaneously exposed to BMP2 and to LPS for 2 hours, they showed a diminished chemokine response (Supplementary Fig. 6g), suggesting that the BMP-driven anti-inflammatory effects occur rather early and precede the enrichment of differentiated pit cells.

Overall, these findings suggest that BMP signaling activation is involved in inhibiting proinflammatory response in antral gastric epithelial cells.

To assess whether epithelial BMP signaling loss has a direct impact on *H. pylori* infection, we infected *Bmpr1a* KO mice with PMSS1 *H. pylori* for 2 weeks or 2 months and performed gene expression analysis on bulk tissue RNA. The results confirmed that *Bmpr1a* and *Id1* were already significantly downregulated in *Bmpr1a* KO mice at the early time point, and that this effect was more pronounced after two months (Supplementary Fig. 7a, b). Both *Muc5ac and Lgr5* were downregulated in KO mice at both timepoints, while *Axin2* was downregulated only after 2 months. CFU analysis revealed that after 2 weeks of infection, a time point at which we usually observe the highest number of bacteria in the tissue, *Bmpr1a* KO mice were significantly less colonized than their WT counterparts (Supplementary Fig. 7c). We explored the transcriptome data of uninfected *Bmpr1a* KO mice and found that loss of epithelial BMP signaling triggered the high expression of several antimicrobial genes such as *Reg3g, Reg3b, Lyz2, Duox2* and *S100a9 and S100a8* (Supplementary Fig. 7d) and the enrichment of antimicrobial gene signatures (Supplementary Fig. 7e, f). We further confirmed in epithelial organoids that the antimicrobial genes *Reg3g*, *Reg3b*, and *Duox2* are indeed expressed in progenitor cells (+BMPi) and are significantly downregulated upon activation of BMP signaling (+BMP2) (Supplementary Fig. 7g).

Together, our organoid data reveal that exposure of epithelial cells to BMP suppresses the ability of epithelium to activate NF-κB signaling upon exposure to PAMPs. Loss of BMP signaling in vivo causes enhanced NF-κB signaling, which is linked to tissue inflammation and elevated antimicrobial responses.

### Stromal-derived PGE2 promotes epithelial YAP activation

**Fig. 4:**
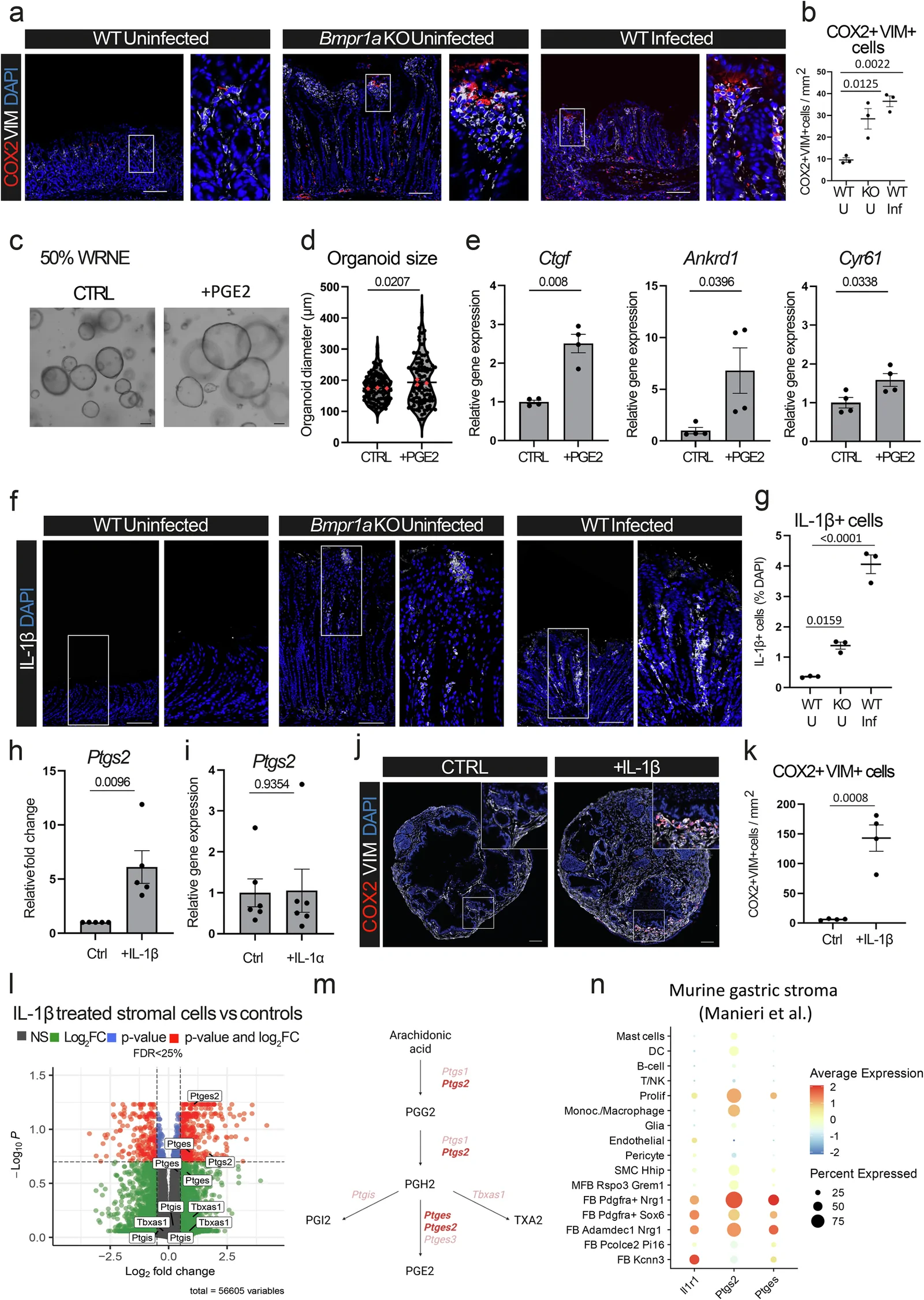
Stromal cells promote epithelial YAP activation via the COX2/PGE2 axis. **a**, **b** Confocal microscopy images and quantification of COX2 and vimentin (VIM) in antral gastric tissue from uninfected WT (WT U), uninfected *Bmpr1a* KO (KO U), and 2-month infected WT (WT Inf) mice (*n* = 3 mice per group). Scale bars: 100 µm. **c** Brightfield images of WT antral gastric organoids grown for 4 days in 50%WRNE medium with or without PGE2. Scale bars: 100 µm. The experiment was performed three times independently with similar results. **d** Quantification of organoid diameter (*n* = 3 biological replicates per group). The mean values for each biological replicate are highlighted in red. **e** mRNA expression analysis of *Ctgf*, *Ankrd1* and *Cyr61* in WT organoids grown in 50%WRNE medium with or without PGE2 (*n* = 4 biological replicates per group). **f**, **g** Confocal microscopy images and quantification of IL-1β and DAPI staining in antral gastric tissue of uninfected WT (WT U), uninfected *Bmpr1a* KO (KO U), and 2-month*-*infected mice (WT inf) (*n* = 3 mice per group). Scale bars: 100 µm. **h**, **i** mRNA expression of *Ptgs2* in murine primary gastric stromal cells upon IL-1β (*n* = 5 biological replicates per group) or IL-1α (*n* = 6 biological replicates per group) treatments. **j**, **k** Confocal microscopy images and quantification of VIM + COX2+ cells in untreated or IL-1β-treated gastric assembloids (*n* = 4 biological replicates per group). Scale bars: 100 µm. **l** Volcano plot comparing microarray data of IL-1β-treated and untreated primary stromal cells. Genes related to arachidonic acid metabolism are highlighted (*n* = 2 biological replicates per group). **m** schematic illustration of the metabolic pathway for PGE2 synthesis from arachidonic acid. Genes upregulated in the IL-1β-treated stromal cells are highlighted in dark red. The image was created in BioRender. Müllerke, S. (2026) https://BioRender.com/iakux39. **n** Dot plot of scRNAseq data from murine gastric stroma (Manieri et al.)^37^ showing the expression of *Il1r1*, *Ptgs2*, and *Ptges* in stromal cell subpopulations. Circle size indicates the within-cluster probability of gene detection; fill color represents the normalized average expression level. Data are presented as mean ± SEM. Statistical analyses were performed using Student’s *t*-test (two-tailed) for **d**, **e,** **h**, **i**, **k**; using one-way ANOVA, followed by Tukey’s multiple comparisons test for **b** and **g**; using unadjusted *p*-values derived from moderated *t*-test as provided by R package limma for (**l**). Images are representative of at least three biological replicates. Source data are provided as a Source data file.

Since we failed to reproduce regenerative reprogramming in epithelial organoids lacking *Bmpr1a*, and since the induction of YAP and the regenerative fetal-like state has been attributed to several growth factors secreted by the stromal compartment^19,25,30^, we hypothesized that the upregulation of YAP signaling in vivo might be induced by signals coming from the mesenchymal niche. Screening our in vivo transcriptome data for differentially expressed genes related to pathways involved in YAP regulation, we identified *Ptgs2* to be upregulated in *Bmpr1a* KO tissue and confirmed this observation via qPCR (Supplementary Fig. 8a). *Ptgs2* encodes COX2, an enzyme involved in the conversion of arachidonic acid into the YAP activator PGE2^31^. Previous studies found COX2 to be expressed in the colonic stromal compartment^31,32^. To examine whether it is expressed in gastric stroma as well, we co-stained antral tissue sections for COX2 and the stromal cell marker vimentin (VIM) (Fig. 4a). We found a significant increase in COX2+ stromal cells, both upon epithelial *Bmpr1a* KO and upon *H. pylori* infection (Fig. 4a, b). Notably, co-staining of COX2 and aYAP showed a close spatial correlation between COX2+ and aYAP+ cells (Supplementary Fig. 8b–e).

To test whether activation of YAP signaling in the tissue is linked to increased COX2 activity, we treated murine gastric organoids with PGE2 and analyzed gene expression levels of YAP target genes via qPCR. When grown in standard medium (100%WRNE), we observed that PGE2-treated organoids grew bigger than untreated controls (Supplementary Fig. 8f, g), but we did not observe significant differences in YAP target gene expression (Supplementary Fig. 8h). In this condition, however, organoids are exposed to high levels of growth factors that boost proliferation and stem cell-related features and may therefore not faithfully recapitulate the homeostatic cell state in vivo. Therefore, we used the 50%WRNE medium characterized above (see Fig. 2h). When organoids grown in 50%WRNE medium were treated with PGE2, they not only grew bigger in size (Fig. 4c, d), but also significantly upregulated the expression of YAP target genes (*Ctgf*, *Ankrd1* and *Cyr61*) (Fig. 4e). Moreover, aYAP+ cells were enriched in the organoids upon PGE2 treatment (Supplementary Fig. 8i). Thus, we conclude that gastric epithelial cells with low basal Wnt activity upregulate YAP signaling upon treatment with PGE2.

To understand how epithelial cells sense stromal PGE2 signals, we analyzed PGE2 receptor expression using our epithelial scRNA-seq. *Ptger4* was broadly expressed in antral epithelial cells, including the stem, TA/progenitor, and pit cell lineages (Supplementary Fig. 8j, k). However, *Ptger1*, *Ptger2*, or *Ptger3* were not expressed in antral epithelial cell types (Supplementary Fig. 8j, k), indicating that *Ptger4* is the primary PGE2 receptor in the antral epithelium. Interestingly, the expression of *Ptger4* was upregulated in the epithelium upon *Bmpr1a* KO (TA/progenitor, pit, and surface mucous cells) (Supplementary Fig. 8l), suggesting an increased responsiveness to PGE2.

We next asked how PGE2 expression in the stroma is induced. Transcriptome analysis revealed a strong upregulation of IL-1β production-dependent gene signatures in *Bmpr1a* KO mice, as well as in *H. pylori*-infected mice, compared to WT controls (Supplementary Fig. 8m). Since IL1-β has been previously associated with inflammation and metaplastic reprogramming in the stomach^33,34^, we asked whether this cytokine might indeed be the trigger for reprogramming via effects on *Ptgs2*/COX2. Immunolabeling confirmed that IL-1β is upregulated in the inter-gland regions of *Bmpr1a* KO tissue as well as upon *H. pylori* infection (Fig. 4f, g). To determine whether IL-1β drives upregulation of *Ptgs2*, we isolated and cultured primary murine stromal cells. qPCR analysis showed that *Ptgs2* was upregulated upon treatment with IL-1β (Fig. 4h) but not IL-1α (Fig. 4i). Despite both cytokines acting on the same receptor, *Il1r1*, they were previously found to have non-redundant effects^35^ in the colonic mucosa. In line with this, our data suggest that IL-1β, and not IL-1α, is responsible for upregulating *Ptgs2* expression in primary gastric stromal cells.

We then asked if we could exploit the assembloid system, a novel 3D in vitro co-culture system combining epithelial and stromal cells, which was previously generated in our lab^36^, to validate these findings in a system that allows cross-compartmental signaling. We thus generated gastric assembloids by combining primary epithelial and stromal cells with gastric organoids. Upon IL-1β treatment, we indeed observed a significant increase in the number of COX2-expressing stromal cells (Fig. 4j, k).

Transcriptome analysis of IL-1β-treated stromal cells additionally revealed that IL-1β induced the upregulation of multiple genes involved in the PGE2 biosynthetic pathway, such as *Ptgs2, Ptges*, and *Ptges2*, while genes involved in the synthesis of other prostanoids were not significantly affected (Fig. 4l, m). To determine which stromal subsets that can produce PGE2, we analyzed a published scRNA-seq dataset of murine gastric stroma^37^ (Supplementary Fig. 9a, b and Fig. 4n), revealing that *Ptgs2* and *Ptges* were mainly expressed in lamina propria fibroblasts (FB *Adamdec1 Nrg1*) and pit fibroblasts (FB *Pdgfra Nrg1* and FB *Pdgfra Sox6*), and those subpopulations also expressed high levels of the IL-1β receptor *Il1r1* (Fig. 4n). Consistently, a published human stomach atlas confirmed that fibroblasts highly express *IL1R1*, while human gastric fibroblasts expressed PGE2 biosynthetic pathway molecules *PTGS2* and almost exclusively *PTGES* (Supplementary Fig. 9c–e).

**Fig. 5:**
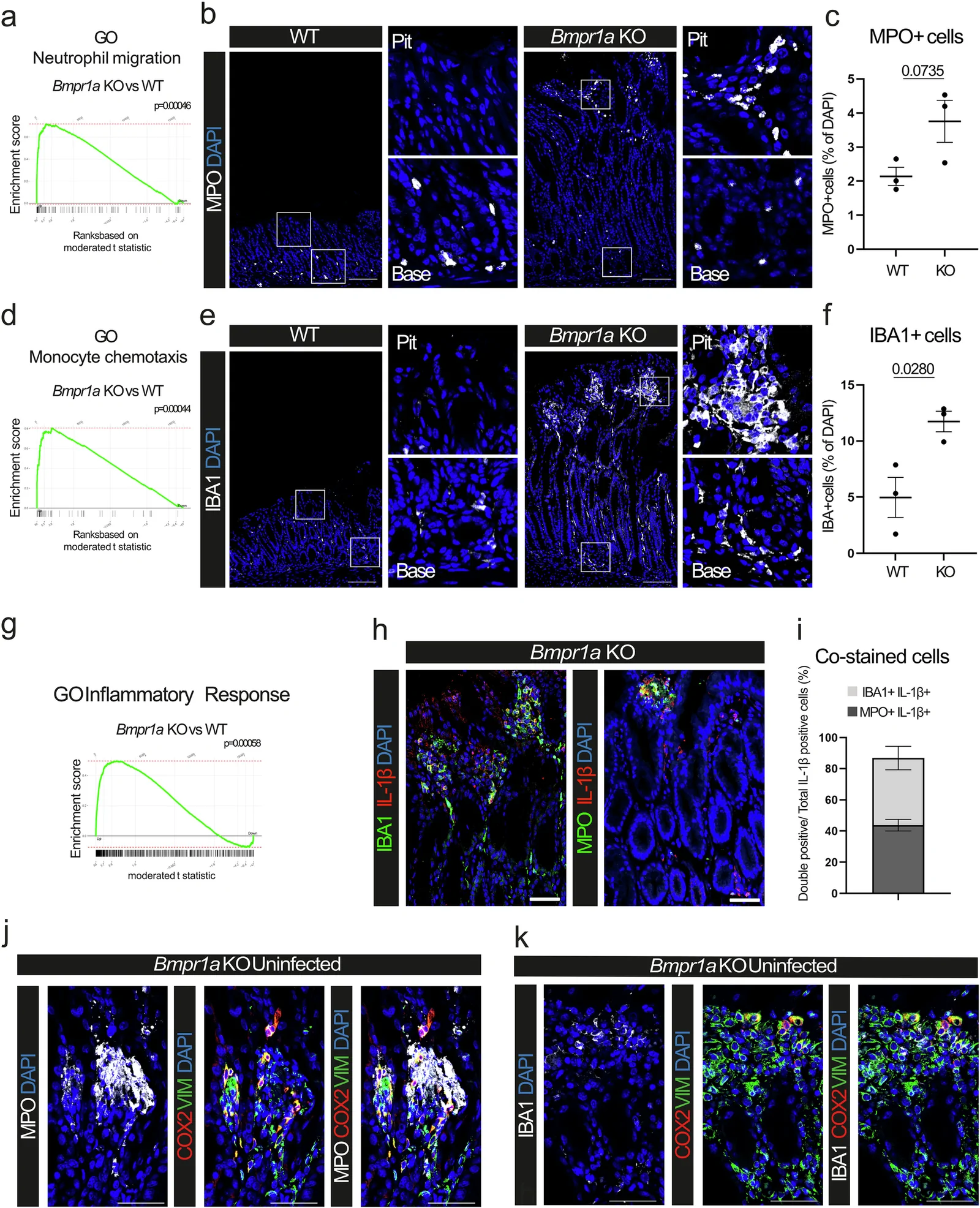
BMP downregulation causes influx of IL-1β-producing immune cells. **a** GSEA of microarray data comparing antral tissue from uninfected *Bmpr1a* KO vs WT mice, for neutrophil migration gene signature (*n* = 2 mice per group). **b**, **c** Confocal microscopy images and quantification of MPO+ cells in antral gastric tissue of uninfected WT and *Bmpr1a* KO mice (*n* = 3 mice per group). Scale bars: 100 µm. **d** GSEA of microarray data comparing antral tissue from uninfected *Bmpr1a* KO vs WT mice, for monocyte chemotaxis gene signature (*n* = 2 mice per group). **e**, **f** Confocal microscopy images and quantification of IBA1+ cells in antral gastric tissue of uninfected WT and *Bmpr1a* KO mice (*n* = 3 mice per group). Scale bars: 100 µm. **g** GSEA of microarray data comparing antral tissue from uninfected *Bmpr1a* KO and WT mice, for inflammatory response gene signature (*n* = 2 mice per group). **h** Confocal microscopy images of co-staining of IL-1β with IBA1 or MPO in antral gastric tissue from uninfected *Bmpr1a* KO mice. Scale bars: 50 µm. **i** Quantification of IL-1β + MPO+ (*n* = 4 mice) or IL-1β + IBA1+ (*n* = 3 mice) co-stained cells normalized to the total IL-1β+ cells per sample, expressed in percentage. **j**, **k** Confocal microscopy images of COX2, VIM, and MPO, and images of COX2, VIM, and IBA1 in antral gastric tissue from uninfected *Bmpr1a* KO mice. Scale bars: 50 µm. Data are presented as mean ± SEM. Statistical analyses were performed using Student’s *t*-test (two-tailed) for **c**, **f**; using unadjusted *p*-values of permutation test provided by R package fgsea for (**a**, **d**, **g**). Images are representative of at least three biological replicates. Source data are provided as a Source data file.

To characterize which cell populations express IL-1β in the gastric mucosa, we further investigated both murine and human stomach scRNA-seq data. These analyses revealed that IL-1β-expressing cells are mainly myeloid cells, including neutrophils, monocytes/macrophages, and dendritic cells (DCs) (Supplementary Fig. 10a, b). In congruence with this, transcriptome analysis of *Bmpr1a* KO mice showed an enrichment of gene signatures related to neutrophil infiltration (Fig. 5a), and immunofluorescence confirmed a markedly increased infiltration of neutrophils (Fig. 5b, c) in gastric tissue of *Bmpr1a* KO mice, which expanded beyond the base compartment. Similarly, *Bmpr1a* KO showed enrichment of gene signatures related to monocytes/macrophages chemotaxis (Fig. 5d), which was again confirmed via immunolabeling (Fig. 5e, f). Furthermore, the inflammatory response gene signature was upregulated in *Bmpr1a* KO mice (Supplementary Fig. 4j and Fig. 5g). We performed immunolabeling for IL-1β and markers of either monocytes/macrophages/DCs (IBA1) or neutrophils (MPO) in *Bmpr1a* KO antral tissue sections, observing that the majority of cells expressing IL-1β belong to these two lineages (Fig. 5h, i). We confirmed this finding also in *H. pylori-*infected WT tissue (Supplementary Fig. 10c, d). Additionally, we investigated whether the IL-1β receptor IL1R1 is altered upon infection, analyzing both human and murine^20^ existing datasets. However, in both cases, we did not detect significant changes in its expression levels (Supplementary Fig. 10e, f).

We then performed co-staining of COX2, VIM, and either MPO or IBA1. In *Bmpr1a* KO tissue, COX2+ VIM+ cells were present in close proximity to clusters of MPO+ neutrophils and IBA1+ monocytes/macrophages/DCs (Fig. 5j-k). Similarly, in *H. pylori*-infected WT mice, we observed that COX2+ VIM+ cells were located adjacent to both MPO+ and IBA1+ cells (Supplementary Fig. 10g-h). This together provides further evidence that stromal cells respond to IL-1β released by immune cells that infiltrate the mucosa.

### IL-1β activates epithelial YAP via stromal COX2-PGE2

To explore the role of IL-1 signaling in the stroma in vivo, we generated *Col1a2*^*CreErt2*^*Il1r1*^*fl/fl*^ mice, hereafter referred to as *Il1r1* KO, an in vivo model in which *Il1r1* is conditionally depleted in the stromal compartment. We confirmed the efficiency of *Il1r1* KO in the stromal compartment of stomach tissue via ISH labeling (Supplementary Fig. 11a).

During homeostasis, different epithelial cell marker genes, such as *Lgr5*, *Lgr4*, *Axin2*, *Gif*, *Muc5ac*, and *Bmp2*, were not affected upon stromal *Il1r1* KO (Supplementary Fig. 11b). Moreover, the gastric mucosa of uninfected *Il1r1* KO mice did not exhibit morphological changes compared to WT mice, and the proliferative cell compartment was not altered (Supplementary Fig. 11c, d). Additionally, both uninfected WT and *Il1r1* KO mice showed very low levels of epithelial active YAP, as was previously observed under uninfected conditions (Supplementary Fig. 11e).

**Fig. 6:**
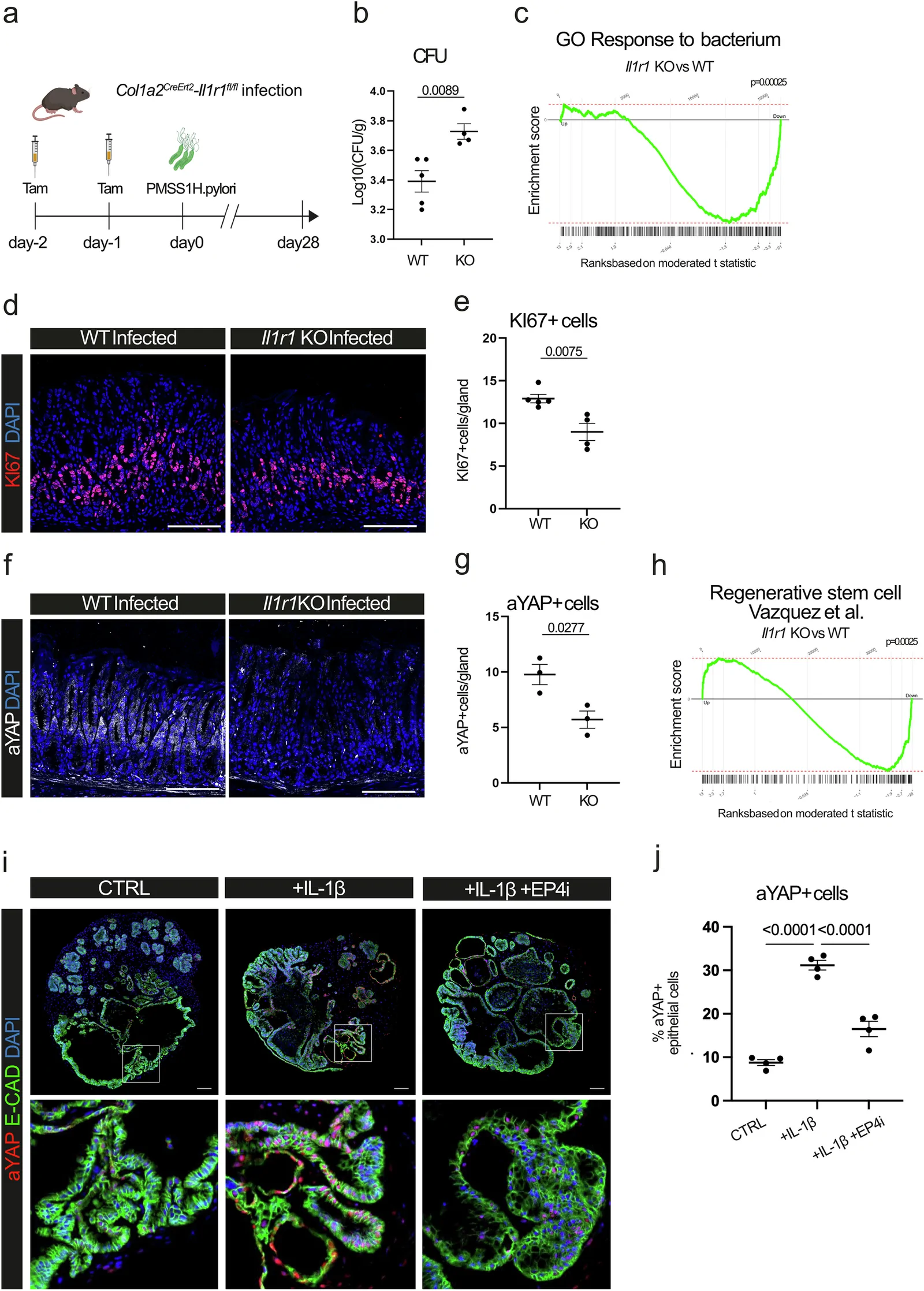
IL1R1-driven stromal cell response promotes activation of the regenerative state in the epithelium. **a** Schematic experimental protocol for the generation of *Il1r1* KO mice in combination with *H. pylori* infection. The image was created in BioRender. Müllerke, S. (2026) https://BioRender.com/a5b2rbw. **b** Colony-forming unit (CFU) quantification of 2-month infected WT (*n* = 5) and *Il1r1* KO (*n* = 4) mice. **c** GSEA of microarray data comparing antral tissue from infected *Il1r1* KO and WT mice, for antimicrobial defense response gene signature (*n* = 2 mice per group). **d**, **e** Confocal microscopy images and quantification of KI67+ cells in antral gastric tissue of WT (*n* = 5) and *Il1r1* KO (*n* = 4) mice infected with *H. pylori*. Scale bars: 100 µm. **f**, **g** Confocal microscopy images and quantification of aYAP+ cells in antral gastric tissue of infected WT and *Il1r1* KO mice (*n* = 3 mice per group). Scale bars: 100 µm. **h** GSEA of microarray data comparing antral tissue from infected *Il1r1* KO and WT mice, for regenerative stem cell gene signature (*n* = 2 mice per group). **i**, **j** Confocal microscopy images and quantification of E-cadherin (E-CAD)+ aYAP+ cells in control, IL-1β-treated, and IL-1β/EP4i-treated gastric assembloids (*n* = 4 biological replicates per group). Scale bars: 100 µm. Data are presented as mean ± SEM. Statistical analyses were performed using Student’s *t*-test (two-tailed) for **b**, **e**, **g**; using one-way ANOVA, followed by Tukey’s multiple comparisons test for **j**; using unadjusted *p*-values of permutation test provided by R package fgsea for (**c**, **h**). Images are representative of at least three biological replicates. Source data are provided as a Source data file.

After two-month infection with PMSS1 *H. pylori* (Fig. 6a), we observed that *Il1r1* KO mice were colonized more densely than their WT counterparts (Fig. 6b), suggesting that the lack of stromal IL-1 signaling activation impairs tissue responses that can counteract bacterial colonization. Consistently, we found that *Il1r1* KO mice had a significantly reduced anti-bacterial gene response signature compared to WT mice (Fig. 6c). Despite this, *Il1r1* KO mice developed less hyperplasia compared to WT mice and had reduced numbers of proliferative epithelial cells (Fig. 6d, e). *Il1r1* KO mice also failed to upregulate epithelial YAP activity and contained reduced numbers of aYAP+ epithelial cells (Fig. 6f, g). Furthermore, transcriptome analysis showed that the regenerative stem cell gene signature was downregulated in *Il1r1* KO antral tissue (Fig. 6h).

Next, we used the assembloid system to analyze whether we could observe differences in epithelial YAP activity upon IL-1β exposure in a controlled in vitro system. We found that assembloid treatment with IL-1β led to significantly higher numbers of epithelial cells with nuclear aYAP (Fig. 6i, j). To confirm that IL-1β activates epithelial YAP via stromal COX2-PGE2 pathway, we added PGE2 receptor EP4 (encoded by *Ptger4*) inhibitor L-161,982 to IL-1β-treated assembloids. aYAP staining showed a significant reduction of aYAP+ epithelial cells upon EP4 inhibition (Fig. 6i, j).

To investigate the direct effects of IL-1β on epithelial cells, we treated epithelial organoids with IL-1β at different concentrations and time points. Unlike PGE2, IL-1β failed to promote organoid growth (Supplementary Fig. 11f, g). Furthermore, qPCR and immunofluorescence of IL-1β-treated organoids did not show significant upregulation of YAP signaling (Supplementary Fig. 11h, i) that was observed in PGE2-treated organoids (see Fig. 4e and Supplementary Fig. 8i), suggesting that IL-1β induces epithelial regenerative state indirectly.

Together, these data demonstrate that IL-1β secreted by invading immune cells acts as a crucial signal that triggers stromal remodeling upon infection, leading to subsequent reprogramming of the epithelium into the regenerative state (Supplementary Fig. 11j).

## Discussion

The gastrointestinal epithelium undergoes a constant turnover. Mature differentiated cell types, such as mucus-producing cells, have a limited life span and are continuously replaced by progenitor cells. In adult tissue homeostasis, epithelial proliferation is mainly regulated by high Wnt activity in the antrum gland base, which is tightly controlled by growth factors secreted by peri-cryptal stromal cells. Several studies have highlighted that upon injury to the gut mucosa, epithelial regeneration is driven by signaling pathways other than Wnt^23,25^. Specifically, YAP signaling, which is crucial in regulating embryonic development but silent in adult tissue, becomes reactivated during injury-induced regeneration^29^, leading to the fetal-like regenerative state in the epithelium. Activation of this state was shown to be induced upon parasite infection in other regions of the gut, although the underlying mechanism remained unresolved^23^. We previously showed that *H. pylori* infection induces hyperproliferation in epithelial cells and upregulation of *Rspo3*, which in the corpus is required to induce the epithelial regenerative state via YAP signaling activation^17,19^. However, overexpression of *Rspo3* alone was not sufficient to induce gland hyperplasia or activation of YAP^19^, leading us to hypothesize that an additional trigger is necessary to induce epithelial remodeling. The nature of this additional trigger has so far remained elusive. Here, we demonstrate that in the gastric antrum, the activation of IL-1 signaling in stromal cells is key in driving tissue reprogramming. Our data indicate that the stromal niche is not only required to provide the growth factors necessary to guide the stem cell fate during homeostasis, but that it is remodeled upon tissue inflammation, whereupon it drives regenerative fetal-like transcriptional reprogramming.

IL-1β is a key proinflammatory cytokine that is elevated in the stomach of *H. pylori*-infected patients and is known to amplify inflammatory responses^34^. While polymorphisms of the *IL1B* gene have been linked to gastric cancer risk^38^, the cell type-specific effects of IL-1β have not been investigated in detail. We find that the mesenchymal stromal cell compartment expresses the highest levels of IL1R1 and is highly responsive to IL-1β. Stimulation of this pathway induces various genes in the COX2-PGE2 axis, including *Ptgs2*, which is linked to gastrointestinal regeneration. The effect of IL-1β signaling via the IL1R1 receptor in stromal cells has been previously investigated in the intestine, where it was shown to upregulate Wnt and R-spondin signaling upon injury^39^. Noticeably, we also find that IL-1α is not capable of inducing *Ptgs2* upregulation. This result is in line with a recent publication that highlighted that IL-1α and IL-1β act in a non-redundant manner, and that IL-1β is responsible for driving tissue repair in the colon upon colitis^35^. *Ptgs2*/COX2 is an enzyme involved in arachidonic acid metabolism. In contrast to the constitutively expressed isoform COX1, COX2 is inducible via a number of different stimuli, including inflammatory triggers^40^. COX2 is involved in the production of PGE2, which has been shown to activate YAP signaling via the prostaglandin E receptor 4 (*Ptger4*)^31^. Previous research showed that COX2 is expressed in stromal cells and can promote tumorigenesis in the colon^31^. Moreover, animal studies have highlighted that COX2 inhibition is linked to tumor suppression in the intestine^41^, while transgenic mouse models of PGE2 induction in the stomach showed that activation of the COX2-PGE2 pathway induces hyperplasia and metaplasia^42^. COX2 is upregulated both in gastric cancer and in premalignant lesions^43^, suggesting a potential role in driving the initial stages of malignant transformation. Furthermore, recent studies highlighted that administration of non-steroidal anti-inflammatory drugs (NSAIDs), which block COX activity, can play a role in the prevention of tumorigenesis in the gastrointestinal tract^44^. Clinical studies additionally showed that *H. pylori* eradication correlates with downregulation of COX2^45^, thus supporting the hypothesis that infection can trigger COX2 activation. Here, we reveal the mechanism responsible for these diverse observations, by showing that, upon IL-1β stimulation by invading immune cells, COX2-PGE2 signaling in mesenchymal stromal cells promotes YAP activation in the neighbouring epithelium and contributes to fetal-like transcriptional changes. This illuminates the interplay between innate and adaptive immune responses as well as different mucosal compartments in triggering tissue regeneration, but can ultimately also lead to transformation.

YAP activation has been described as a crucial pathway involved in multiple cancer types, including colorectal and gastric tumors^46^, where it was shown to act synergistically with Wnt signaling to promote malignant transformation^26,47^. While YAP overexpression is linked to gastric cancer progression and aggressiveness^48^, it is already upregulated in early stages of carcinogenesis, such as in preneoplastic lesions^49^. Although various studies on intestinal injury models have revealed stereotypic reprogramming of the intestinal epithelium into a highly proliferative regenerative state that transcriptionally resembles the fetal intestinal epithelium^24,50^, little is known about fetal-like transcriptional reprogramming in the stomach. Indeed, the mechanisms by which gastric epithelial reprogramming is induced and how stomach tissue reprogramming affects host-pathogen interactions, such as in the case of *H. pylori* infection, have remained unclear.

Previous research showed that differentiated cells lack the ability to upregulate proinflammatory cytokines upon exposure to *H. pylori* or its PAMPs^14–16^. Our findings clarify that this is linked to the BMP signaling status of differentiated epithelial cells, which suppresses epithelial NF-κB signaling, and that this effect is not limited to PAMPs but can be generalized to pro-inflammatory triggers. As the luminal surface is more likely to come into contact with microbes or bacteria-derived products, this suggests that active BMP signaling in the pit region of the mucosa not only drives differentiation, but also renders differentiated epithelial cells more tolerant to the presence of microbes. Reduction of BMP signaling therefore de-represses proinflammatory epithelial responses and ignites tissue inflammation. Previous studies indeed noticed that the upregulation of Noggin, a BMP signaling antagonist, causes enhanced inflammation in the stomach upon *H. pylori* infection^51^, and we provide here a molecular framework for the role of BMP in regulating the balance of tolerance and inflammation.

In summary, we unravel a crosstalk between epithelial, stromal, and immune cells that induces the fetal-like regenerative transcriptional state and demonstrate that chronic activation of this pathway, as in *H. pylori* infection, can drive premalignant tissue pathology.

## Methods

### Mouse experiments

Our study complied with all relevant ethical regulations. All animal experiments were performed under guidance and approval of institutional, local, and national legal authorities (Landesamt für Gesundheit und Soziales (LaGeSo) Berlin, licenses G0084_17 and G0144_22). Animals were maintained in autoclaved microisolator cages and provided with sterile drinking water and chow ad libitum. The mice were bred at the animal care facility on a 12-h light/12-h dark cycle in a controlled temperature (22.5 ± 2.5 °C) and humidity (50 ± 5%) environment. C57BL/6 mice were obtained from Charles River Laboratory. *Axin2*^*CreErt2*^*Bmpr1a*^*fl/fl*^ (epithelial-specific *Bmpr1a* KO) mice were generated in our lab previously^20^. *Il1r1* ^*fl/fl*^*Col1a2*^*CreErt2*^ (stromal-specific *Il1r1* KO) mice were generated by crossing *Il1r1* ^*fl/fl*^ mice^39^ with *Col1a2*^*CreErt2*^ mice^52^. Male and female mice were used for *Il1r1* ^*fl/fl*^*Col1a2*^*CreErt2*^ mouse experiments, and they were randomly allocated to experimental groups. To induce KO, tamoxifen (Sigma, 4 mg/25 g body weight, diluted in 200 μl corn oil) was injected intraperitoneally as a double dose on two consecutive days at the indicated time points. *H. pylori* infection in mice was performed 7 days after tamoxifen treatment, for 2 weeks or 2 months. Unless stated otherwise, male 6- to 12-week-old mice were used for this study.

At the experimental endpoint, mice were euthanized by cervical dislocation performed by trained personnel. Death was confirmed by respiratory arrest before tissue harvesting. The forestomach was removed, and the glandular stomach was opened along the lesser curvature and flattened on paper. The stomach contents were removed. Corpus, antrum, and pylorus were identified in terms of color and structure^53^. The tissue was divided into three parts along the greater curvature, one used for histology, one for CFU counts (in infected animals), and one for transcriptome analysis. Experiments were performed in at least 4 biological replicates per condition.

### *H. pylori* infection and CFU analysis

Mice were orally infected with a single dose of 10^8^ *H. pylori* strain PMSS1 and euthanized at the indicated time points. A longitudinal section of stomach tissue was weighed, and tissue was mechanically homogenized in brain-heart infusion medium (BD Bioscience). Colony-forming units (CFUs) were determined after plating serial dilutions of tissue homogenates, counting the number of colonies in each plate. Bacterial counts are expressed as the number of colonies per gram of stomach.

### Tissue processing

To obtain longitudinal gastric tissue sections, samples were first fixed in 4% PFA overnight and washed with PBS. Samples were then embedded in paraffin blocks and cut into 1–2 µm thick sections by the Charité Core Unit Immunopathology for Experimental Models.

### Immunofluorescence staining and imaging

Tissue and organoid formalin-fixed paraffin-embedded (FFPE) sections were rehydrated and, after antigen retrieval in either 1x citrate or TRIS-EDTA buffer, were blocked for 1 h at room temperature with blocking buffer (0.1% Tween 20 in PBS (PBS-T) supplemented with 5% FBS). Sections were then incubated overnight with primary antibodies diluted in blocking buffer at 4 °C in a humidified chamber, followed by 2 h incubation at room temperature in a humidified chamber with secondary antibodies diluted in blocking buffer. Sections were counterstained with DAPI (Cell Signaling, 1:1000) for visualization of nuclei. The following antibodies were used: mouse anti-COX2 (BD Biosciences, 610204, 1:150), mouse anti-E-cadherin (clone 36) (BD, 610181, 1:200), Alexa Fluor 647-conjugated lectin GSII (Thermo Scientific, L32451, 1:200), rabbit anti-IBA1 (Fujifilm Wako Chemicals, 019-19741, 1:500), rabbit anti-IL-1β (WH295161) (ABClonal, A11370, 1:150), rabbit anti-KI67 (D3B5) (Cell Signaling Technology, 9129S, 1:200); mouse anti-MUC5AC (45M1) (Invitrogen, 12178, 1:200), rabbit anti-MPO (Abcam, ab208670, 1:200), goat anti-MPO (R&D, AF3667, 1:200), rabbit anti-vimentin (D21H3) (Cell Signaling Technology, 5741S, 1:200); rat anti-vimentin (R&D Systems, MAB2105, 1:200), rabbit anti-active YAP1 (Abcam, ab205270, clone EPR19812, 1:150); goat anti-mouse Alexa Fluor 488 (Jackson Immunoresearch, catalog 115-545-003, 1:300), and goat anti-rabbit Cy3 (Jackson Immunoresearch, catalog 115-165-146, 1:300), donkey anti-rabbit Alexa Fluor 488 (Jackson Immunoresearch, catalog 711-545-152, 1:300), donkey anti-mouse Cy3 (Jackson Immunoresearch, catalog 715-166-020, 1:300).

Samples were imaged with a Leica Stellaris8 and a Falcon-STED confocal microscope. Images were analyzed with Leica Application Suite X (LAS X, v3.7.4.23463) and with Fiji/ImageJ (1.53c).

The total number of MPO, IBA1, and IL1-β positive cells was calculated with Fiji/ImageJ. The values are expressed as percentage of the number of positive cells to the total number of DAPI+ stained cells in the tissue section. The *muscularis mucosae* was excluded from the analysis. For COX2 positive cell quantification, the number of double positive COX2 and vimentin (VIM) cells was normalized to tissue area. KI67 and aYAP positive cells were quantified by counting the number of positive cells per gland. COX2-aYAP co-staining images were analyzed using QuPath (v0.6.0). Single cells were segmented via DAPI signals with a 3.0-µm cytoplasmic expansion. Cell phenotyping utilized intensity-based classifiers: mean cellular intensity for COX2+ cells and mean nuclear intensity for aYAP+ cells. To assess spatial association, 2D centroid distances from each aYAP+ cell to the nearest COX2+ cells were calculated.

### Organoid culture

Organoids were generated from mouse antral gastric glands. Antrum tissue was isolated and incubated in PBS with 0.5 mM DTT and 10 mM EDTA for 20 minutes at 37 °C. Gastric glands were mechanically dissociated from the tissue and washed with PBS. Glands were mixed with 30 µl Matrigel (BD) and plated in 24-well plates. After polymerization of Matrigel, murine gastric culture medium (FM), composed of Advanced Dulbecco’s Modified Eagle´s Medium/F12 (Gibco) supplemented with 10 mM HEPES (Gibco), 2 mM GlutaMAX (Gibco), 100 U/ml penicillin/streptomycin (Gibco) 1xB27 (Gibco), 1xN2 (Gibco), 1.25 mM N-acetyl cysteine (Sigma), 50 ng/ml mouse epidermal growth factor (EGF) (Invitrogen), 100 ng/ml mouse noggin (PeproTech), 100 ng/ml fibroblast growth factor 10 (FGF10) (PeproTech), 10 nM gastrin (Sigma), 10% Rspo1-conditioned medium, 500 nM A83-01 (#616454, Merck) and 0.328 nM surrogate Wnt (sWnt) (U-Protein Express) was added to the wells. The medium supplemented with growth factors was replaced every 2–3 days. Rock inhibitor (Y-27632, Sigma) was added at 10 µM concentration after passaging and was removed after 2–3 days. For BMP2 treatment, organoids were first grown in complete murine gastric medium for 2–3 days, then treated with 50 ng/ml of recombinant BMP2 protein (R&D, 355-BM-010) for 3–5 days. Noggin was removed during BMP2 treatment*. Klebsiella pneumoniae* LPS (1 µg/ml; Sigma-Aldrich), ADP-heptose (1 µM; Invivogen), and IL-1β (10 ng/ml; Preprotech) treatments were added to the medium, and cells were harvested at the indicated times. For short-time BMP2 treatment, organoids were first grown in complete murine gastric medium for 5 days, then treated with 50 ng/ml of recombinant BMP2 protein (R&D, 355-BM-010) 2 h. *Klebsiella pneumoniae* LPS (1 µg/ml; Sigma-Aldrich) treatment for 2 h was performed together with BMP2 treatment. To generate *Bmpr1a* KO organoids, 800 nM 4-OH-tamoxifen (Sigma) was added to organoid cultures from *Axin2*^*CreErt2*^*Bmpr1a*^*fl/fl*^ mice for 48 h. *Bmpr1a* KO organoids were selected by treating cultures with BMP2 (30 ng/ml) for 5–7 days. qPCR for *Bmpr1a* was used to validate the KO efficiency. For PGE2 treatment experiments, organoids were grown either in murine gastric organoid medium (FM) or in standard medium containing 50% Wnt3a, 50% R-spondin1 conditioned medium, 50% Noggin, and 50% EGF (referred to as 50%WRNE). Organoids were treated with PGE2 (20 μM, Biozol) from seeding and harvested together with controls on day 4. Brightfield images were obtained with an EVOS M5000 microscope (Thermo Fisher Scientific), and organoid size was measured manually with Fiji/ImageJ (1.53c) software. For staining, organoids were fixed in 3.7% formaldehyde for 3 , washed twice with 0.1% BSA in PBS, and embedded in 2% agarose in PBS. Samples were then manually dehydrated, paraffinized, and cut into 5 μm thick sections.

### Stromal cell culture

After removal of the forestomach, the stomach was opened along the lesser curvature. Tissue was cleared of the gastric content and washed in PBS. Epithelial cells were removed by incubating tissue in PBS with 0.5 mM DTT and 10 mM EDTA for 20 minutes at 37 °C followed by mechanical agitation. Stomach tissue was then mechanically dissociated into small pieces, which were washed and incubated in calcium- and magnesium-free HBSS (Gibco) containing Liberase TL (1 unit/ml; Roche) and DNase I (1 unit/ml; Invitrogen) at 37 °C. This solution was vigorously pipetted every 10 minutes. Every 20 min, the digested fraction was collected, mixed with ice-cold medium containing Advanced Dulbecco’s Modified Eagle Medium/F12 supplemented with 5% HEPES, 5% Glutamax, 10% fetal bovine serum (FBS) (Gibco), 100 U/ml penicillin/streptomycin, and 10 μM Rock inhibitor (Y-27632), and left on ice. This cycle was repeated three more times for a total digestion time of 60 min. The isolated stromal cells were seeded in 12- or 6-well plates and cultured in stromal cell medium for at least one passage before experiments. IL-1β (50 ng/ml, Preprotech) treatment was performed 3 times (every 2–3 days), and the last treatment was performed one day prior to harvesting. IL-1α (50 ng/ml, Preprotech) treatment was performed in the same way.

### Assembloid culture

Gastric assembloids were generated according to the protocol published in ref. ^36^ and cultured for a total of 5 days. For the first 4 days, assembloids were grown in gastric organoid medium deprived of sWnt, R-spondin 1 conditioned medium, and Noggin. During the last day of culture, A83-01 was removed. EP4 inhibitor L-161,982 (20 μM, Sigma-Aldrich, SML0690) was supplemented from the beginning of culture, and treatment with IL-1β (10 ng/ml) was performed for the last 48 h. Assembloids were harvested in 4% PFA and then embedded in 2% agarose for subsequent immunofluorescent analysis. For quantification of COX2+ VIM+ cells, the number of double-positive cells and the area of each assembloid section were quantified. For quantification of active YAP (aYAP) epithelial cells, the number of aYAP+ E-cadherin (E-CAD)+ cells was quantified and normalized to the total E-CAD+ cell number.

### Microarray analysis

Microarray experiments were performed as independent dual-color dye-reversal color-swap hybridizations using 2 biological replicates. Quality control and quantification of total RNA were assessed with either an Agilent 2100 Bioanalyzer or an Agilent Fragment Analyzer System (Agilent Technologies) and a NanoDrop 1000 UV-Vis spectrophotometer (Kisker). RNA labeling was performed with a dual-color Low Input Quick-Amp Gene Expression Labeling Kit (Agilent Technologies). In brief, mRNA was reverse transcribed and amplified using an oligo-dT-T7 promoter primer, and the resulting cRNA was labeled with cyanine 3–CTP or cyanine 5–CTP. After precipitation, purification, and quantification, 600 ng of each labeled cRNA was fragmented and hybridized to whole-genome mouse microarrays (Agilent-074809, SurePrint G3 Mouse Gene Expression v2 8x60K) according to the supplier’s protocol (Agilent Technologies). Scanning of microarrays was performed at 3 × 3 micron and 20 bit image depth resolution using a G2565CA high-resolution laser microarray scanner (Agilent Technologies). Microarray image data were analyzed with the Image Analysis/Feature Extraction software G2567AA 12.2.07 (Agilent Technologies) using default settings and the GE2_1200_Dec17 extraction protocol. The extracted files were analyzed further using R version 4.3.3^54^ and the package limma^55^ for normalization and differential gene expression analysis.

### Gene set enrichment analysis (GSEA)

We performed GSEA on genes ranked by gene expression–based t-score between antral gastric tissue from epithelial *Bmpr1a* KO vs WT controls, IL-1β-treated vs untreated stromal cells, and stromal *Il1r1* KO vs WT controls using the fgsea R package^56^ with 5000 permutations. We analyzed gene sets from MSigDB v7.1^57^, a stem cell signature of LGR5+ cells in intestinal crypts^58^; a gene set of the signature of fetal-like regenerative epithelial cells^59^; and YAP target gene signatures: YAP Tg up and YAP KO down^25^.

### Single-cell RNA-Sequencing analysis

For murine scRNA-seq analysis, we performed epithelial cell isolation from *Bmpr1a* WT and KO mice (uninfected *Bmpr1a*^*fl/fl*^ and *Axin2*^*CreErt2*^*Bmpr1a*^*fl/fl*^, antrum and the transitional zone between antrum and corpus) and from 2-month *H.pylori* uninfected and infected WT mice (the whole stomach), following the method described in the section “Organoid culture”. Two mice for each condition (eight mice in total) were used. Once gastric glands were isolated, samples were treated with TrypLE at 37 °C for 8 minutes, in order to obtain a single-cell suspension. Via fluorescence-activated cell sorting (FACS), we sorted EPCAM-positive cells (PE-conjugated rat anti-CD326 (EpCAM), Miltenyi Biotec, 130-117-779, clone caa7-9G8, 1:200), excluding CD45-positive cells (APC-Cy7-conjugated rat anti-CD45, BD, 557659, clone 30-F11, 1:200) and dead cells (BD Horizon™ Fixable Viability Stain 450 (562247) (FVS-450), 1:2000). FACS was perform on the sorter FACS Aria III (BD) with the software FACSDiva 9.4.

Sorted cells were further processed with Chromium Fixed RNA Profiling kits (10x Genomics) according to the manufacturer’s instructions. Single cells from eight mice were sequenced together without pooling in one multiplexed run using 10x GEM-X Flex fixed RNA profiling, and reads were processed using 10x CellRanger v9.0.1 with transcriptome version GRCm39-2024-A and Chromium Mouse Transcriptome Probe Set v1.1.1. The R package Seurat v5.0.1 was used for the analysis, keeping cells with between 1000 and 10,000 detected features and at most 5% mitochondrial reads. We applied cell normalization using method LogNormalize, followed by identification of variable features using method vst. Sample integration was performed using Harmony, correcting for sample effects, and UMAP and neighbors were calculated using the first 15 principal components. After excluding contaminating stromal and immune cells, epithelial clusters were identified with a resolution of 0.3. We adjusted scaled expression values for cell cycle effects. Cluster marker signatures were determined using the function FindAllClusterMarkers using standard settings. We used the expression of *Pdx1*, a gene specifically expressed in the stomach antrum but not corpus^60^, and identified clusters with higher mean normalized expression (mean > 0.2) as of antral origin. We then repeated the full preprocessing for those antral clusters as described above, setting the clustering resolution to 0.65. Pseudo-bulk analysis of scRNA-seq data was performed by aggregating counts per sample and cluster and computing differential expression using R package DESeq2^61^ (unadjusted and Benjamini-Hochberg adjusted *p*-values of Wald-test as provided by DESeq2). Differences in cluster proportions between conditions were assessed using the propeller method from R package speckle^62^.

We constructed a human stomach scRNA-seq atlas from published data sets (GSE206785^63^, GSE249874^64^, GSE263018^65^, and Tsubosaka et al.^66^). Each data set was first independently preprocessed using Seurat with doublet identification with scDblFinder^67^ followed by integration with Harmony^68^. Global clusters were defined for each data set using known marker genes, and clusters with high proportions of doublets or low-quality cells were flagged for exclusion. Finally, data sets were integrated with Harmony and preprocessed using Seurat. After global identification of major lineages on the combined atlas data set, we performed separate processing and cluster identification for epithelial, stromal, and immune cells based on known marker gene expression. From the atlas, we selected normal samples (mainly from sleeve resections) and non-atrophic gastritis samples with or without *H. pylori* infection and visualized average expression and detection rates in dot plots for selected genes.

For the analysis of mouse stomach stromal cells, we obtained 4 samples of gastric mesenchymal cells provided in GSE224737 (Manieri et al.)^37^ and processed the scRNA data as described for the global analysis of the human stomach scRNA atlas above.

### RNA in-situ hybridization (ISH)

RNA in-situ detection was performed on FFPE tissue sections using the RNAscope Red Detection Kit (Advanced Cell Diagnostics, 322360), according to the manufacturer’s protocol. Positive and negative control probes were used for each experiment according to the manufacturer’s instructions. The following probes were used: mouse *Anxa1* (catalog 509291), mouse *Bmpr1a* (catalog 312421), mouse *Il1r1* (catalog 413211), and mouse *Lgr5* (catalog 312171). Images were acquired with a Leica MICA microscope and analyzed with Leica Application Suite X (LAS X, v3.7.4.23463) and Fiji/ImageJ (1.53c). Quantification of *Lgr5*+ cells was conducted as follows: for each biological sample, the number of cells positive for the *Lgr5* signal was counted in 20 glands. Mean values per biological sample were calculated and plotted. Values are expressed as mean ± SEM.

### Quantitative RT-PCR

For antral gastric tissue transcriptome analysis, RNA was extracted from snap-frozen tissue using the NucleoSpin RNA isolation kit (Macherey & Nagel), including on-column DNase digestion. Organoid RNA was extracted with the same kit, according to the manufacturer´s instructions. Quality and quantity controls were performed for each extract using the NanoDrop 1000 Spectrophotometer (Thermo Scientific). cDNA was generated using the iScript cDNA synthesis kit (Bio-Rad). Reactions were performed in a reaction mix of 20 μl containing 100 ng cDNA, 10 μl SYBR Green (Thermo Fisher), and 0.1 μM primer mix. Reactions were performed with a QuantStudio Real-Time PCR System (Thermo Fisher) according to the subsequent protocol: 10 min at 95 °C followed by 40 cycles of 15 s at 95 °C/60 s at 60 °C. For each oligonucleotide pair and RNA sample, the reaction was performed in duplicates. The amplification plots obtained from RT-PCR were analyzed with the QuantStudio^TM^ Design and Analysis software (version 1.4.3, Applied Biosystems). The expression levels of the target genes were normalized to the levels of *Gapdh* or *β-Actin* expression in each sample in tissue or organoids, respectively. Primer sequences were either designed using the NCBI Primer-BLAST tool or adopted from previously published literature, and all were synthesized by Sigma-Aldrich. Detailed information on primers can be found in Supplementary Data 3.

### Western Blot analysis

Organoids were either grown in gastric organoid medium (FM) for 5 days or grown for 2 days in FM and 3 days in medium supplemented with 50 ng/ml of recombinant BMP2 protein (R&D, 355-BM-010). Cells were treated with ADP-heptose (1 µM, Invivogen), LPS (1 µg/ml, Sigma-Aldrich), and IL-1β (10 ng/ml, Preprotech) and harvested at the indicated time points. The organoids were first incubated with organoid harvesting solution (R&D Systems) for 20 min on ice, washed with cold PBS and then incubated with RIPA buffer (50 mM Tris (pH 7.5), 150 mM NaCl, 2 mM EDTA, 10 mM K_2_HPO_4_, 10% glycerol, 1% Triton X-100, 0.05% SDS) supplemented with 1 mM Na_3_VO_4_, 1 mM Na_2_MoO_4_, 20 mM NaF, 10 mM Na_4_P_2_O_7_, 20 mM Glycerol-2-phosphate, and 1× EDTA-free protease inhibitor mix (PI) (cOmplete™, Mini, Roche) for 30 min on ice. Lysates were obtained after centrifugation (13,000 × *g*). The total protein amount of lysates was measured by the bicinchoninic acid (BCA) method using Pierce^TM^ BCA Protein Assay Kit (23225, Thermo Fisher Scientific). Then, 20 µg of lysates were mixed with Laemmli buffer, heated at 95 °C for 5 min, separated on SDS-polyacrylamide gel, and blotted onto 0.45 µm Immobilon®-P PVDF membrane (IPVH00010, Millipore). The membrane was blocked with 5% dry skim milk in TBS-T (20 mM Tris-HCl, pH 7.6, 137 mM NaCl, 0.1% Tween20) at room temperature (RT) for 1 h. After overnight incubation with a specific primary antibody at 4 °C, the membrane was probed with a secondary antibody conjugated with horseradish peroxidase for 1 h at RT. Proteins were visualized using chemiluminescent substrate (WBKLS0500, Millipore) and ChemoCam Imager (Intas). The following antibodies were used: p-IKKα/β (CST #2697, 1:1000 in 5% BSA), IKKα (CST #2682, 1:1000 in 5% BSA), p-IκBα (CST, #9246, 1:1000 in 5% milk), IκBα (CST, #4812, 1:1000 in 5% BSA), p-SMAD1/5 (CST, #9516, 1:1000 in 5% milk), SMAD1 (CST, #6944, 1:1000 in 5% milk), p-YAP (CST, #53749, 1:1000 in 5% milk), YAP (CST, # 14074, 1:1000 in 5% BSA),p100 (CST #4882, 1:1000 in 5% BSA), p-p100 (CST, #4810, 1:1000 in 5% BSA) and GAPDH (Millipore MAB374, 1:5000 in 5% milk).

### Statistics and reproducibility

Data are expressed as mean ± SEM. For comparison of two groups, a two-tailed, unpaired t-test was performed. Two-tailed ordinary one-way ANOVA was used for statistical comparison of more than two groups. All analyses of statistical significance were calculated and displayed compared with the reference control group unless otherwise stated. *P* < 0.05 was considered significant. Unless stated otherwise, experiments were performed at least three times independently with similar results, using a minimum of three biological replicates in total. No statistical method was used to predetermine sample size. No data were excluded from the analyses. The mice and organoids/stromal cells/assembloids were randomized for respective treatments. Group allocation and analysis were done in a blinded fashion. GraphPad Prism 10.3.1 and R 4.5.2 were used for data visualization and statistical analysis.

### Reporting summary

Further information on research design is available in the Nature Portfolio Reporting Summary linked to this article.

## Supplementary information

Supplementary Information

Description of Additional Supplementary Files

Supplementary Data 1

Supplementary Data 2

Supplementary Data 3

Reporting Summary

Transparent Peer Review file

## Source data

Source Data

## Data Availability

The data for GSEA of the microarray data in this manuscript from uninfected *Bmpr1a* KO and WT antral tissue can be accessed under the Gene Expression Omnibus database (GSE283811 [https://www.ncbi.nlm.nih.gov/geo/query/acc.cgi?acc=GSE283811]). The data of microarray analysis from infected *Il1r1* KO and WT antral tissue can be accessed under the Gene Expression Omnibus database (GSE283927 [https://www.ncbi.nlm.nih.gov/geo/query/acc.cgi?acc=GSE283927]). The data of microarray analysis from primary stromal cells treated with IL1-β can be accessed under the Gene Expression Omnibus database (GSE302280 [https://www.ncbi.nlm.nih.gov/geo/query/acc.cgi?acc=GSE302280]). The bulk RNA-seq data for WT infected vs uninfected antral tissue^20^ can be accessed via the Gene Expression Omnibus database (GSE136658 [https://www.ncbi.nlm.nih.gov/geo/query/acc.cgi?acc=GSE136658]). The scRNA-seq data for primary gastric epithelial cells can be accessed in the Gene Expression Omnibus (GSE319373 [https://www.ncbi.nlm.nih.gov/geo/query/acc.cgi?acc=GSE319373]). Source data are provided with this paper.

## Code availability

All computational codes can be accessed at https://github.com/Sigal-Lab/Beccaceci_Stomach_Bmp1r_KOhttps://github.com/Sigal-Lab/Beccaceci_Stomach_Il1r_KO, and https://github.com/Sigal-Lab/Beccaceci_Il1b_stroma.
