# Supplementary material for: Helicobacter pylori triggers gastric mucosal remodeling toward a fetal-like transcriptional program via stromal IL-1β signaling
Source: Nat Commun. 2026 Sep 5;17:9510. doi: 10.1038/s41467-026-77520-1 (PMC13546300; doi:10.1038/s41467-026-77520-1)
Supplement: Supplementary file 1 [file 41467_2026_77520_MOESM1_ESM.pdf]

Supplementary Information

Helicobacter pylori triggers gastric mucosal remodeling toward a fetal-like transcriptional program via stromal IL-1β signaling

Giulia Beccaceci, Stefanie Müllerke, Hilmar Berger, Christian Täger, Ronja Möbius, Anne-Sophie Fischer, Kimberly Hartl, Jonas Wizenty, Hans-Joachim Mollenkopf, Michael Naumann, Manqiang Lin, Michael Sigal

This PDF file includes:

Supplementary Figures 1-11

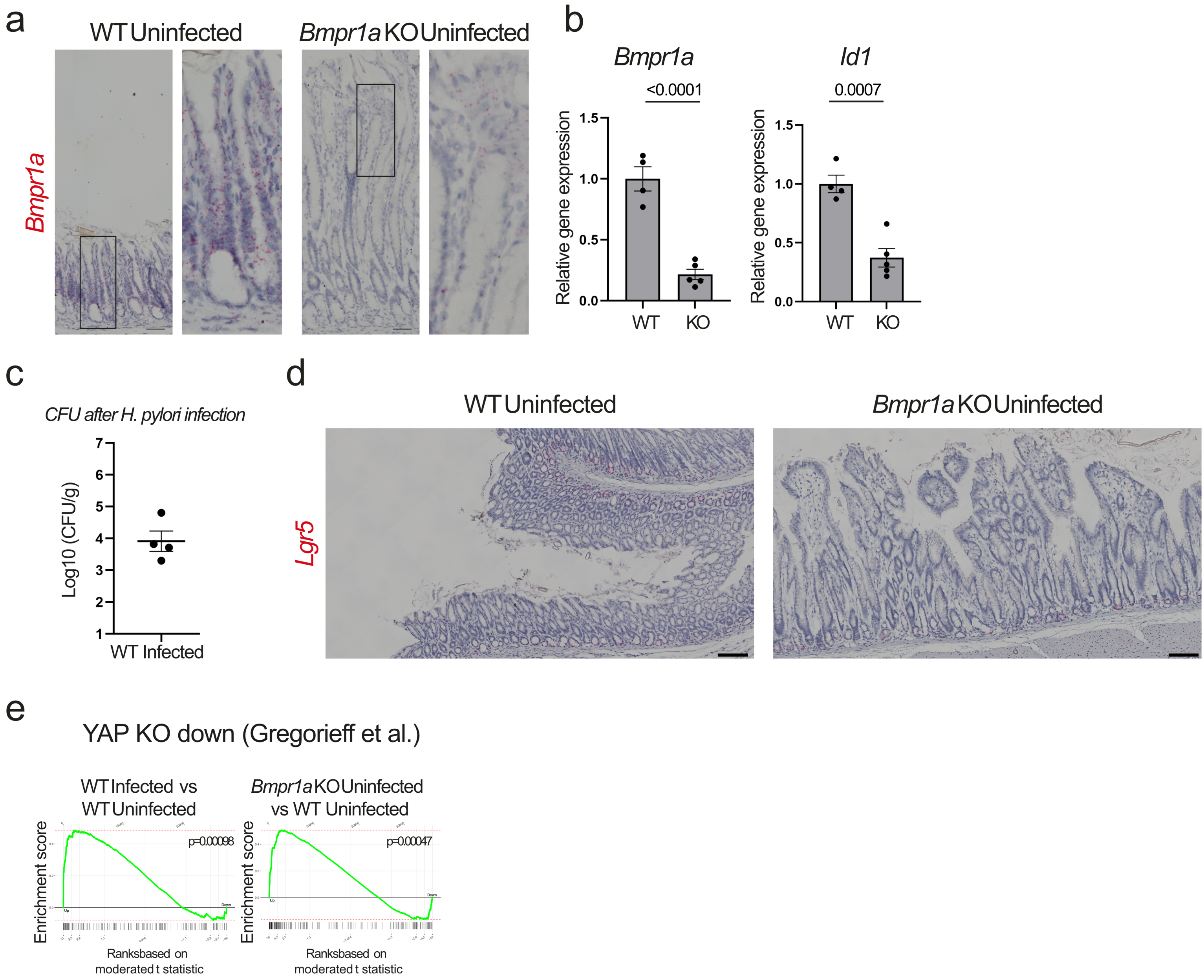
a
WT Uninfected
Bmpr1a KO Uninfected
Bmpr1a
b
Bmpr1a
Id1
<0.0001
0.0007
Relative gene expression
WT
KO
c
CFU after H. pylori infection
Log10 (CFU/g)
WT Infected
d
WT Uninfected
Bmpr1a KO Uninfected
Lgr5
e
YAP KO down (Gregorieff et al.)
WT Infected vs WT Uninfected
Bmpr1a KO Uninfected vs WT Uninfected
Enrichment score
p=0.00098
p=0.00047
Ranksbased on moderated t statistic

Supplementary Fig.1 Characterization of *Bmpr1a* KO mice and *H. pylori* infection

a ISH images for *Bmpr1a* in antral tissues from uninfected WT (*n* = 4) and *Bmpr1a* KO (*n* = 5) mice. Scale bars: 50 µm. **b** mRNA expression of *Bmpr1a* and *Id1* in uninfected WT (*n* = 4 mice) and *Bmpr1a* KO (*n* = 5 mice) antral tissues. **c** Colony-forming unit (CFU) quantification for gastric tissues of 2-month-infected WT mice (*n* = 4 mice). **d** ISH images for *Lgr5* in sections from uninfected WT and *Bmpr1a* KO mice. **e** GSEA of microarray data comparing antral tissue from infected WT vs. uninfected WT mice, as well as from uninfected *Bmpr1a* KO vs. WT mice (*n* = 2 mice per group), for the YAP target gene signature (YAP KO down). Scale bar: 100 µm. Data are presented as mean ± SEM. Statistical analyses were performed using Student's *t*-test (two-tailed) for **b**; using unadjusted *p*-values of permutation test provided by R package fgsea for **e**. Images are representative of at least three biological replicates. Source data are provided as a Source Data file.

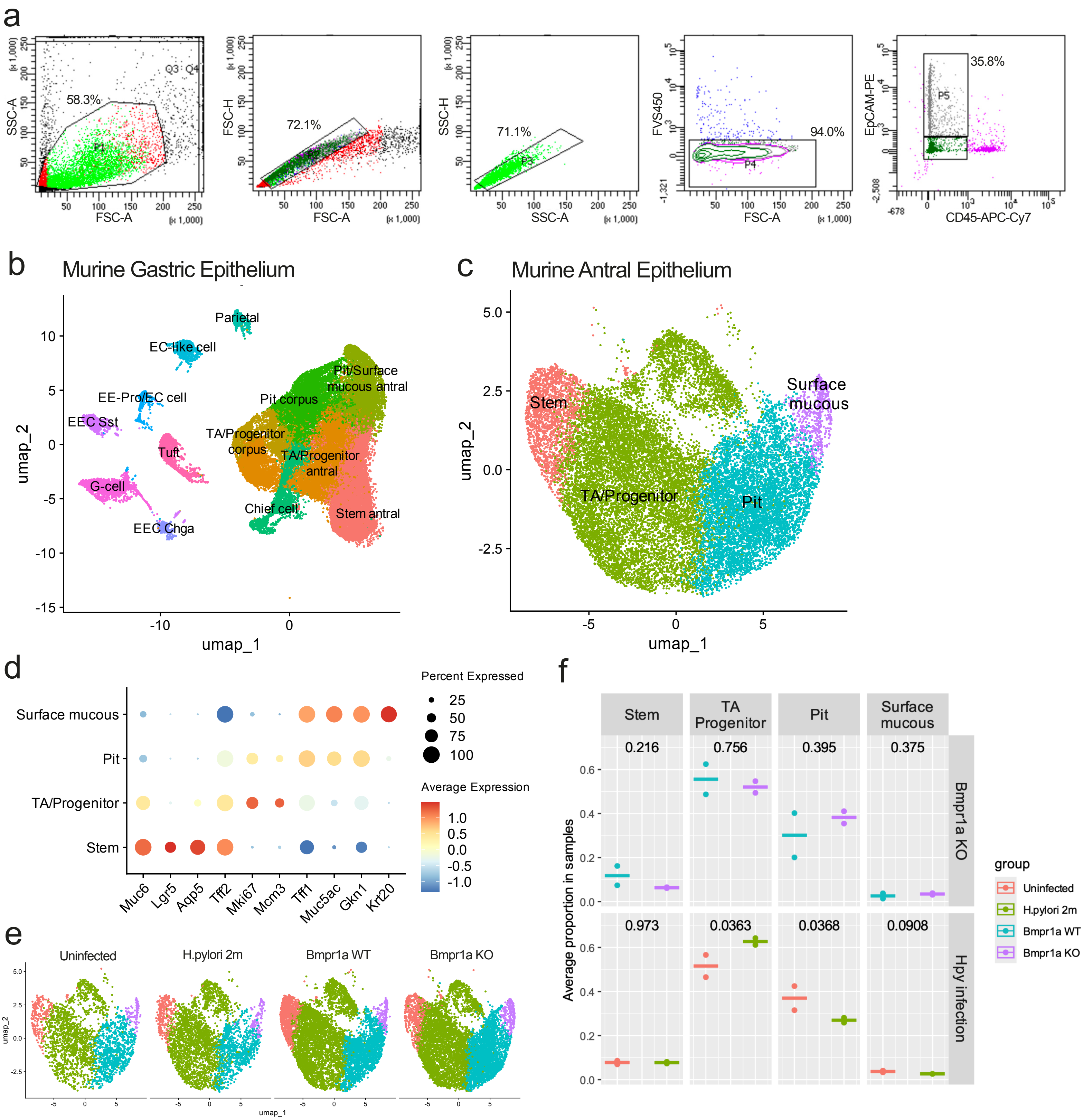
a
SSC-A
FSC-A
58.3%
P1
Q3
Q4
FSC-H
72.1%
SSC-H
SSC-A
71.1%
P3
FVS450
94.0%
P4
EpCAM-PE
CD45-APC-Cy7
35.8%
P5
b
Murine Gastric Epithelium
Parietal
EC-like cell
EE-Pro/EC cell
EEC Sst
Tuft
G-cell
EEC Chga
Pit corpus
Pit/Surface mucous antral
TA/Progenitor corpus
TA/Progenitor antral
Chief cell
Stem antral
umap_1
umap_2
c
Murine Antral Epithelium
Stem
TA/Progenitor
Pit
Surface mucous
umap_1
umap_2
d
Surface mucous
Pit
TA/Progenitor
Stem
Muc6
Lgr5
Aqp5
Tff2
Mki67
Mcm3
Tff1
Muc5ac
Gkn1
Krt20
Percent Expressed
25
50
75
100
Average Expression
1.0
0.5
0.0
-0.5
-1.0
e
Uninfected
H.pylori 2m
Bmpr1a WT
Bmpr1a KO
umap_1
umap_2
f
Stem
TA Progenitor
Pit
Surface mucous
0.216
0.756
0.395
0.375
0.973
0.0363
0.0368
0.0908
Bmpr1a KO
Hpy infection
Average proportion in samples
group
Uninfected
H.pylori 2m
Bmpr1a WT
Bmpr1a KO

Supplementary Fig. 2 Single-cell RNA-sequencing of murine gastric epithelium

a Single cells were gated based on SSC-A, FSC-A, FSC-H, and SSC-H; live cells were identified by negative FVS450 staining, and epithelial cells were selected as EpCAM-positive and CD45-negative. Gate P5 indicates the epithelial cell population selected for downstream processing. **b-c** UMAP plots of the scRNA-seq dataset showing integrated clusters and cells from uninfected WT vs. infected WT mice, as well as from uninfected *Bmpr1a* KO vs. WT mice. Main epithelial cell clusters from the antrum were isolated for further analysis (**c**). **d** Dot plot showing the expression of cluster marker genes across identified subpopulations in the antral epithelium. Circle size indicates the within-cluster proportion of gene detection; fill color represents the scaled average expression level. **e** Individual UMAP plots showing antral epithelial cells from uninfected WT vs. infected WT mice, as well as from uninfected *Bmpr1a* KO vs. WT mice. **f** Comparison of the proportions of antral epithelial subpopulations in uninfected WT vs. infected WT mice, as well as from uninfected *Bmpr1a* KO vs. WT mice. Numbers indicate *p*-values of proportion differences between groups (*n* = 2 mice per group; moderated *t*-test after logit-transform of proportions).

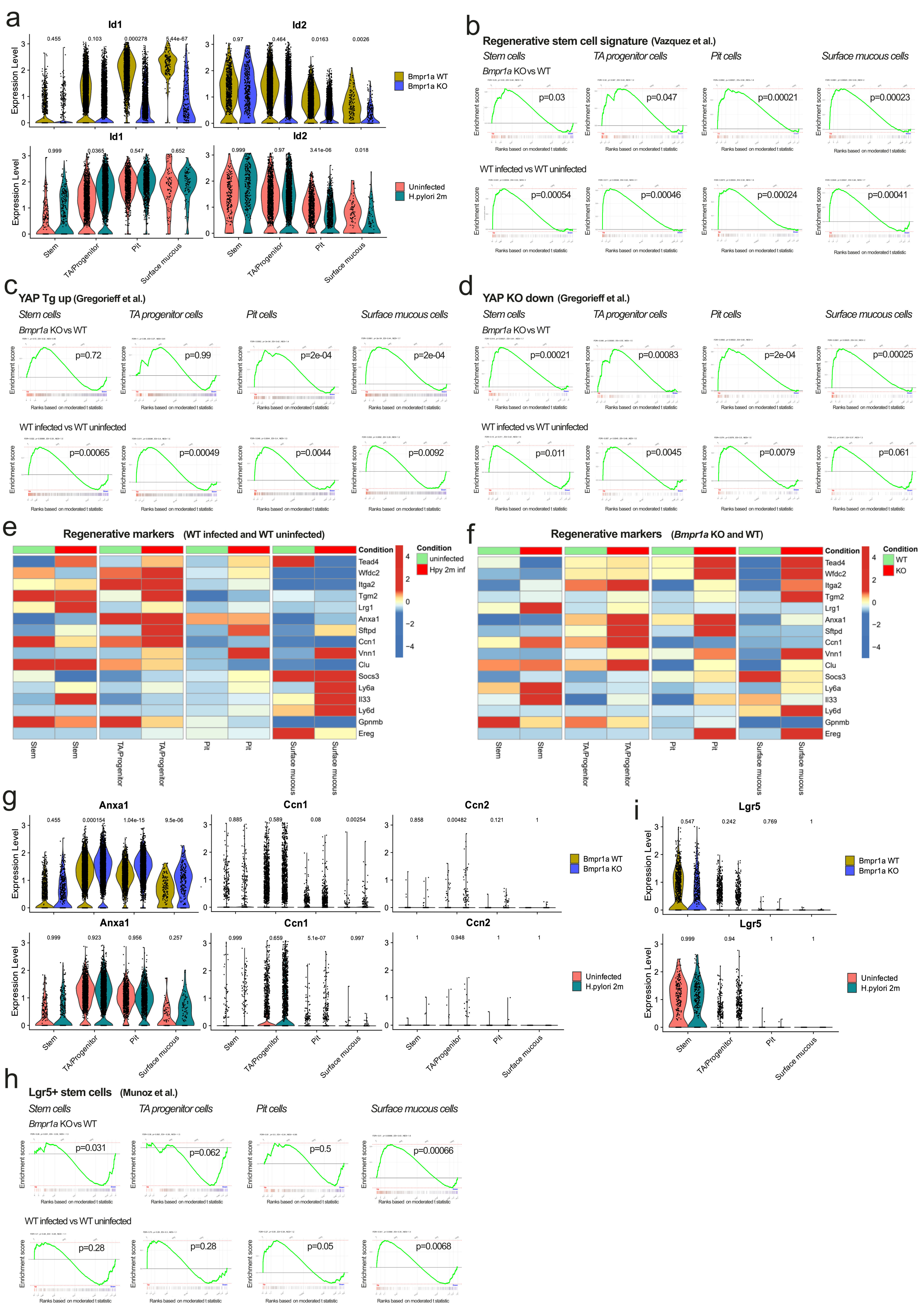
a
Id1
Id2
Bmpr1a WT
Bmpr1a KO
Uninfected
H.pylori 2m
Expression Level
Stem
TA/Progenitor
Pit
Surface mucous
b
Regenerative stem cell signature (Vazquez et al.)
Stem cells
TA progenitor cells
Pit cells
Surface mucous cells
Bmpr1a KO vs WT
WT infected vs WT uninfected
p=0.03
p=0.047
p=0.00021
p=0.00023
p=0.00054
p=0.00046
p=0.00024
p=0.00041
Enrichment score
Ranks based on moderated t statistic
c
YAP Tg up (Gregorieff et al.)
p=0.72
p=0.99
p=2e-04
p=2e-04
p=0.00065
p=0.00049
p=0.0044
p=0.0092
d
YAP KO down (Gregorieff et al.)
p=0.00021
p=0.00083
p=2e-04
p=0.00025
p=0.011
p=0.0045
p=0.0079
p=0.061
e
Regenerative markers (WT infected and WT uninfected)
Condition
uninfected
Hpy 2m inf
f
Regenerative markers (Bmpr1a KO and WT)
WT
KO
Tead4
Wfdc2
Itga2
Tgm2
Lrg1
Anxa1
Sftpd
Ccn1
Vnn1
Clu
Socs3
Ly6a
Il33
Ly6d
Gpnmb
Ereg
g
Anxa1
Ccn1
Ccn2
i
Lgr5
h
Lgr5+ stem cells (Munoz et al.)
p=0.031
p=0.062
p=0.5
p=0.00066
p=0.28
p=0.28
p=0.05
p=0.0068

Supplementary Fig. 3 Gene expression and signature analyses of murine gastric epithelium

a Violin plot showing the expression level of *Id1* and *Id2* in antral epithelial cells from uninfected *Bmpr1a* KO vs. WT mice, as well as from infected WT vs. uninfected WT mice. **b-d** GSEA of scRNA-seq pseudo-bulk expression differences comparing antral tissue from uninfected *Bmpr1a* KO vs. WT mice, as well as from infected WT vs. uninfected WT mice, for regenerative stem cell signature (**b**), and YAP target gene signatures YAP Tg up (**c**) and YAP KO down (**d**). **e-f** Heatmap showing average expression of regenerative marker genes in antral epithelial cell populations. **g** Violin plot showing the expression level of *Anxa1*, *Ccn1* (*Cyr61*), and *Ccn2* (*Ctgf*) in antral epithelial cells from uninfected *Bmpr1a* KO vs. WT mice, as well as from infected WT vs. uninfected WT mice. **h** GSEA of scRNA-seq comparing antral tissue from uninfected *Bmpr1a* KO vs. WT mice, as well as from infected WT vs. uninfected WT mice, for *Lgr5*+ stem cell signature. **i** Violin plot showing the expression level of *Lgr5* in antral epithelial cells from uninfected *Bmpr1a* KO vs. WT mice, as well as from infected WT vs. uninfected WT mice. For **a**, **g**, **i**, numbers at the top indicate *p*-values for mean differences between conditions for each cluster (n = 2 mice per group), derived from genome-wide pseudo-bulk differential expression analysis of antral scRNA-seq data. Statistical analyses were performed using unadjusted *p*-values of permutation test provided by R package fgsea for **b**-**d** and **h.**

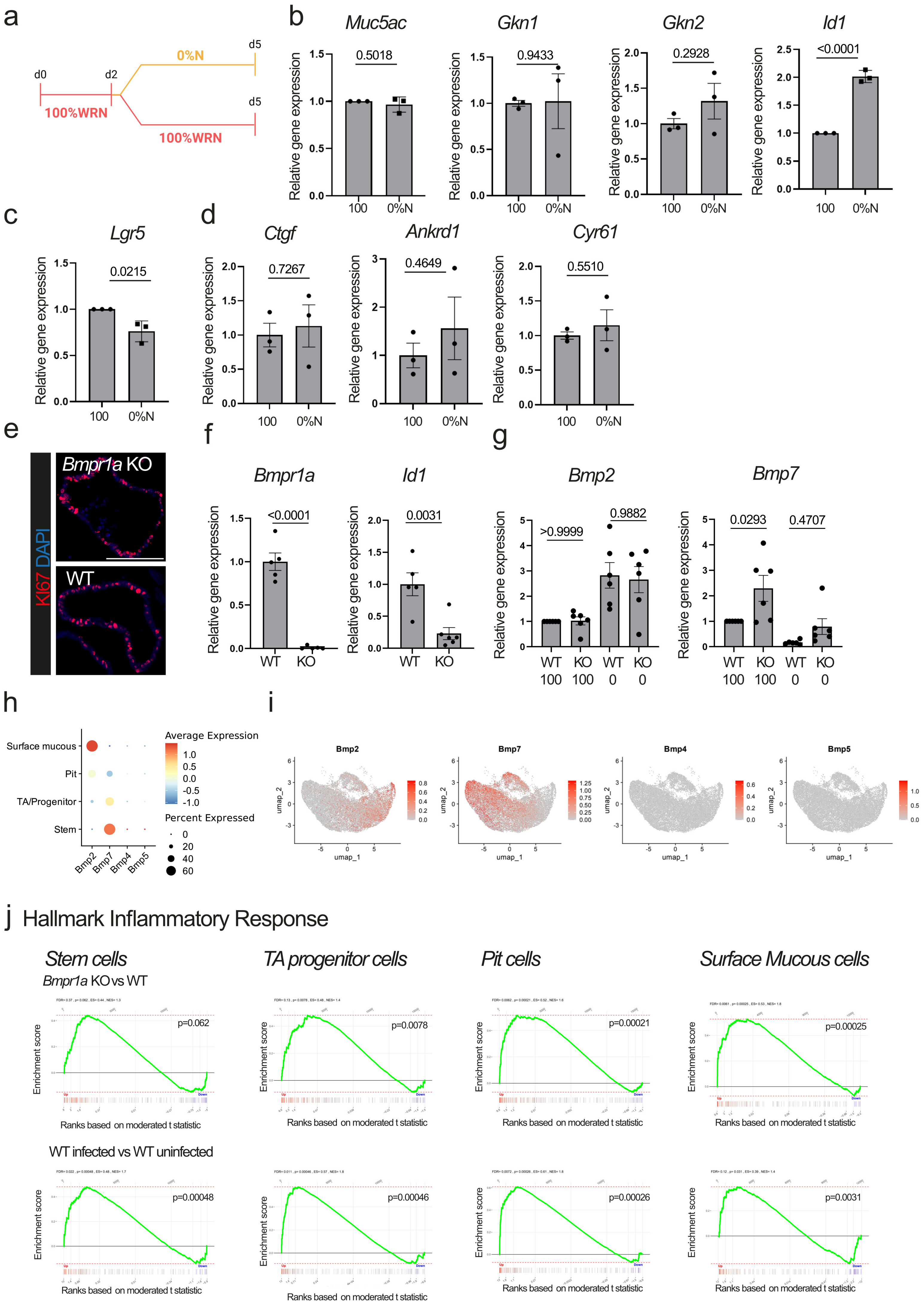

a
d0
d2
d5
0%N
d5
100%WRN
100%WRN
b
Muc5ac
0.5018
Gkn1
0.9433
Gkn2
0.2928
Id1
<0.0001
Relative gene expression
100
0%N
c
Lgr5
0.0215
d
Ctgf
0.7267
Ankrd1
0.4649
Cyr61
0.5510
e
Bmpr1a KO
WT
KI67 DAPI
f
Bmpr1a
<0.0001
Id1
0.0031
WT
KO
g
Bmp2
>0.9999
0.9882
Bmp7
0.0293
0.4707
WT
KO
100
0
h
Surface mucous
Pit
TA/Progenitor
Stem
Bmp2
Bmp7
Bmp4
Bmp5
Average Expression
1.0
0.5
0.0
-0.5
-1.0
Percent Expressed
0
20
40
60
i
Bmp2
Bmp7
Bmp4
Bmp5
umap_1
umap_2
j Hallmark Inflammatory Response
Stem cells
TA progenitor cells
Pit cells
Surface Mucous cells
Bmpr1a KO vs WT
p=0.062
p=0.0078
p=0.00021
p=0.00025
WT infected vs WT uninfected
p=0.00048
p=0.00046
p=0.00026
p=0.0031
Enrichment score
Ranks based on moderated t statistic

Supplementary Fig. 4 Inhibition of epithelial BMP signaling and the epithelial inflammatory response upon infection or *Bmpr1a* KO

a Organoid treatment schematic. Created in BioRender. Müllerke, S. (2026) https://BioRender.com/l9g4426. **b-d** mRNA expression of the differentiated cell markers *Muc5Ac, Gkn1* and *Gkn2* (**b**), the BMP signaling target *Id1* (**b**), the stem cell marker *Lgr5* (**c**), and YAP target genes *Ctgf, Ankrd1* and *Cyr61* (**d**) in WT organoids grown in 100%WRN or 0%N medium (*n* = 3 biological replicates per group). **e** Confocal microscopy images of KI67 and DAPI staining in WT and *Bmpr1a* KO organoids. Scale bar: 100 µm. The experiment was performed three times independently with similar results. **f** mRNA expression of *Bmpr1a* and *Id1* in WT (*n* = 5 biological replicates) and *Bmpr1a* KO (*n* = 6 biological replicates) organoids. **g** mRNA expression of the BMP ligands *Bmp2* and *Bmp7* in WT and *Bmpr1a* KO organoids grown in 100%WRN or 0%WRN medium (*n* = 6 biological replicates per group). **h** Dot plot showing the expression of BMP ligands across identified cellular components in the murine antral epithelium. Circle size indicates the within-cluster proportion of gene detection; fill color represents the scaled average expression level. **i** UMAP plots of scRNA-seq showing expression levels of BMP ligands in antral epithelial cells. **j** GSEA of scRNA-seq pseudo-bulk expression differences comparing antral tissue from uninfected *Bmpr1a* KO vs. WT mice, as well as from infected WT vs. uninfected WT mice, for MsigDB HALLMARK_INFLAMMATORY_RESPONSE signature. Data are presented as mean ± SEM. Statistical analyses were performed using Student's *t*-test (two-tailed) for **b-d** and **f**; using one-way ANOVA, followed by Tukey's multiple comparisons test for **g**; using unadjusted *p*-values of permutation test provided by R package fgsea for **j**. Source data are provided as a Source Data file.

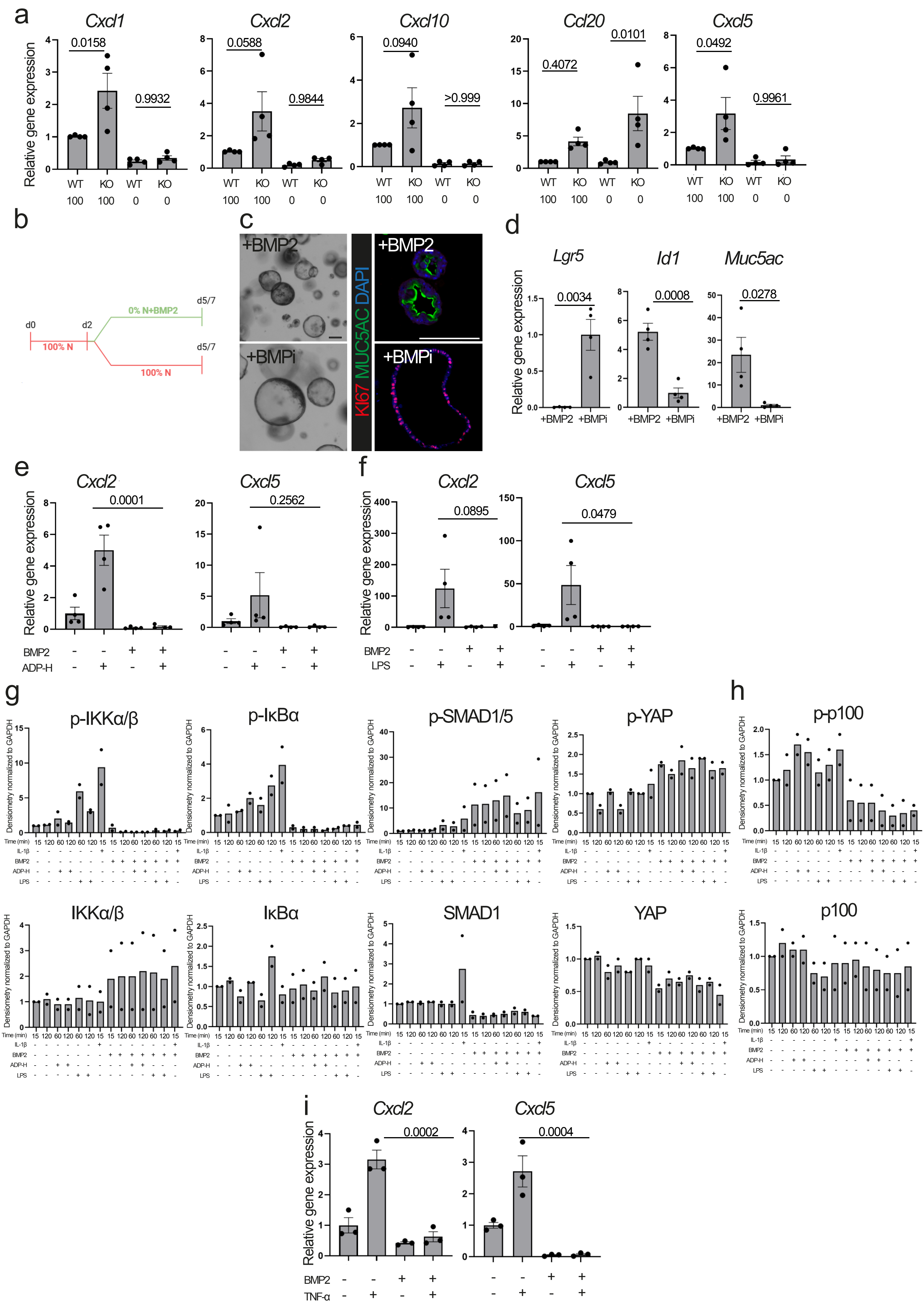
a
Cxcl1
Cxcl2
Cxcl10
Ccl20
Cxcl5
Relative gene expression
0.0158
0.9932
0.0588
0.9844
0.0940
>0.999
0.4072
0.0101
0.0492
0.9961
WT KO WT KO
100 100 0 0
b
d0
d2
100% N
0% N+BMP2
100% N
d5/7
d5/7
c
+BMP2
+BMPi
KI67 MUC5AC DAPI
d
Lgr5
Id1
Muc5ac
0.0034
0.0008
0.0278
+BMP2 +BMPi
e
Cxcl2
Cxcl5
0.0001
0.2562
BMP2
ADP-H
f
Cxcl2
Cxcl5
0.0895
0.0479
BMP2
LPS
g
p-IKKα/β
p-IκBα
p-SMAD1/5
p-YAP
IKKα/β
IκBα
SMAD1
YAP
Densiometry normalized to GAPDH
Time (min)
IL-1β
BMP2
ADP-H
LPS
h
p-p100
p100
i
Cxcl2
Cxcl5
0.0002
0.0004
BMP2
TNF-α

Supplementary Fig. 5 Epithelial BMP signaling regulates proinflammatory response

a mRNA expression of the proinflammatory cytokines *Cxcl1, Cxcl2, Cxcl10, Ccl20 and Cxcl5* in WT and *Bmpr1a* KO organoids grown in 100%WRN or 0%WRN (*n* = 4 biological replicates per group). **b** Organoid treatment schematic. Created in BioRender. Müllerke, S. (2026) https://BioRender.com/ijz9u7g. **c** Brightfield and confocal images for KI67, MUC5AC, and DAPI co-staining in antral gastric organoids cultured in standard medium containing the BMP inhibitor Noggin (+BMPi) or in Noggin-deprived medium supplemented with BMP2 (+BMP2). Scale bars: 100 µm. The experiment was performed three times independently with similar results. **d** mRNA expression of stem cell marker *Lgr5*, BMP target gene *Id1,* and differentiated pit cell marker *Muc5ac* in murine gastric organoids cultured in standard full medium (+BMPi) or in Noggin-deprived medium supplemented with BMP2 (+BMP2) (*n* = 4 biological replicates per group). **e-f** mRNA expression of *Cxcl2* and *Cxcl5* in organoids treated with BMP2 and/or ADP heptose (ADP-H) (**e**), and treated with BMP2 and/or LPS (**f**) (*n* = 4 biological replicates per group). ADP-H/LPS treatment was performed for 2 hours. **g-h** Densitometric quantification of the Western blot shown in **Figures 3g-h** (*n* = 2 biological replicates per group). **i** mRNA expression for *Cxcl2* and *Cxcl5* in organoids treated with BMP2 and/or TNF-α. TNF-α treatment was performed for 2 hours (*n* = 3 biological replicates per group). Data are presented as mean ± SEM. Statistical analyses were performed using Student's *t*-test (two-tailed) for **d**; using one-way ANOVA, followed by Tukey's multiple comparisons test for **a**, **e**, and **i**. Source data are provided as a Source Data file.

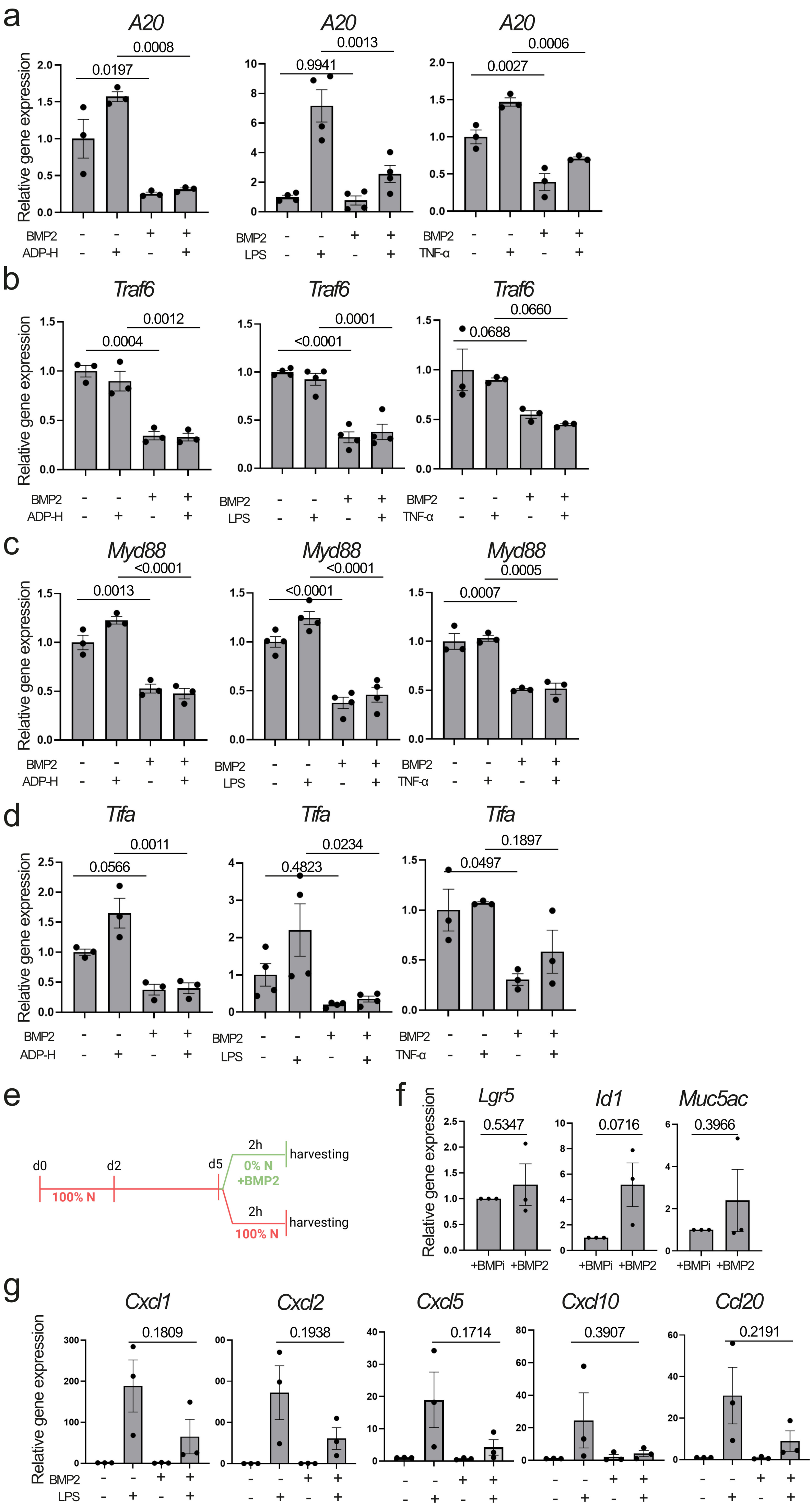
a
A20
A20
A20
0.0197
0.0008
0.9941
0.0013
0.0027
0.0006
Relative gene expression
BMP2
ADP-H
LPS
TNF-α
b
Traf6
Traf6
Traf6
0.0004
0.0012
<0.0001
0.0001
0.0688
0.0660
c
Myd88
Myd88
Myd88
0.0013
<0.0001
<0.0001
<0.0001
0.0007
0.0005
d
Tifa
Tifa
Tifa
0.0566
0.0011
0.4823
0.0234
0.0497
0.1897
e
d0
d2
d5
100% N
2h
0% N
+BMP2
harvesting
2h
100% N
harvesting
f
Lgr5
Id1
Muc5ac
0.5347
0.0716
0.3966
+BMPi
+BMP2
g
Cxcl1
Cxcl2
Cxcl5
Cxcl10
Ccl20
0.1809
0.1938
0.1714
0.3907
0.2191
BMP2
LPS

Supplementary Fig. 6 BMP signaling inhibits the expression of NF-κB signaling mediator genes

a-d mRNA expression of A20, *Traf6*, *Myd88*, and *Tifa* in organoids treated with BMP2 and/or ADP-H (*n* = 3 biological replicates per group), LPS (*n* = 4 biological replicates per group) or TNF-α (*n* = 3 biological replicates per group). Treatment with ADP-H, LPS, or TNF-α was performed for 2 hours. **e** Organoid treatment schematic. Created in BioRender. Müllerke, S. (2026) https://BioRender.com/nskn9y8. **f** mRNA expression of stem cell marker *Lgr5*, BMP target gene *Id1,* and pit cell marker *Muc5ac* in murine gastric organoids cultured in standard full medium (+BMPi) or in Noggin-deprived medium supplemented with BMP2 (+BMP2) (*n* = 3 biological replicates per group). **g** mRNA expression of NF-κB target genes (*Cxcl1, Cxcl2, Cxcl5, Cxcl10, Ccl20*) in organoids treated with BMP2 and/or LPS (*n* = 3 biological replicates per group). LPS and BMP2 treatments were performed for 2 h. Data are presented as mean ± SEM. Statistical analyses were performed using Student's *t*-test (two-tailed) for **f**; using one-way ANOVA, followed by Tukey's multiple comparisons test for **a-d** and **g**. Source data are provided as a Source Data file.

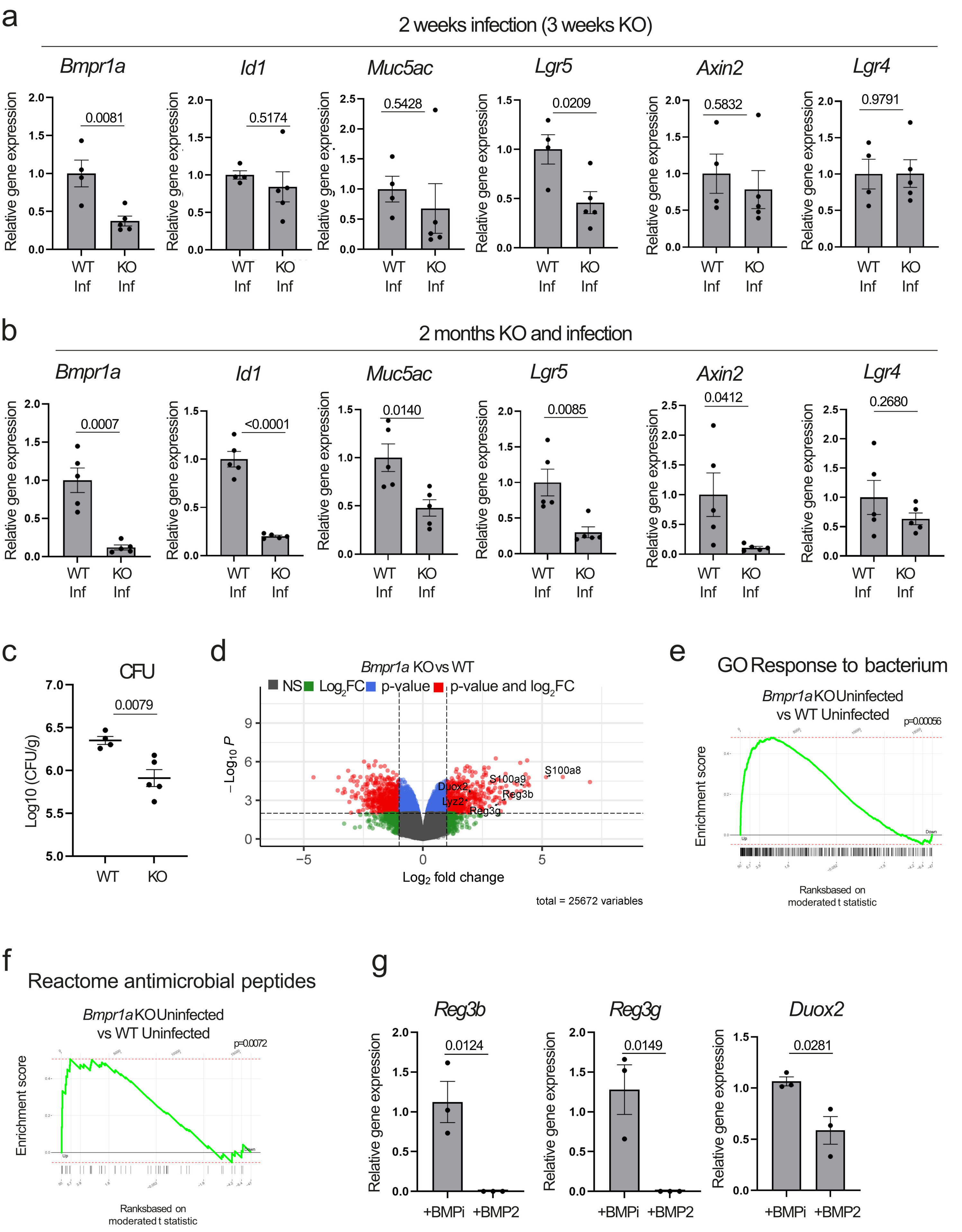
a
2 weeks infection (3 weeks KO)
Bmpr1a
Id1
Muc5ac
Lgr5
Axin2
Lgr4
Relative gene expression
0.0081
0.5174
0.5428
0.0209
0.5832
0.9791
WT Inf
KO Inf
b
2 months KO and infection
Bmpr1a
Id1
Muc5ac
Lgr5
Axin2
Lgr4
Relative gene expression
0.0007
<0.0001
0.0140
0.0085
0.0412
0.2680
WT Inf
KO Inf
c
CFU
Log10 (CFU/g)
0.0079
WT
KO
d
Bmpr1a KO vs WT
NS
Log2FC
p-value
p-value and log2FC
− Log10 P
Log2 fold change
S100a8
S100a9
Duox2
Reg3b
Lyz2
Reg3g
total = 25672 variables
e
GO Response to bacterium
Bmpr1a KO Uninfected
vs WT Uninfected
p=0.00056
Enrichment score
Ranks based on
moderated t statistic
f
Reactome antimicrobial peptides
Bmpr1a KO Uninfected
vs WT Uninfected
p=0.0072
Enrichment score
Ranks based on
moderated t statistic
g
Reg3b
Reg3g
Duox2
Relative gene expression
0.0124
0.0149
0.0281
+BMPi
+BMP2

Supplementary Fig. 7 Inhibition of BMP signaling promotes antimicrobial response

a-b mRNA expression of *Bmpr1a*, *Id1, Muc5Ac, Lgr5, Axin2, Lgr4* in WT and *Bmpr1a* KO antral tissue after 2 weeks (WT: *n* = 4 mice; KO: *n* = 5 mice) (**a**) or 2 months (WT: *n* = 5 mice; KO: *n* = 5 mice) (**b**) of infection with *H. pylori.* **c** Colony-forming unit (CFU) quantification from 2-week-infected WT (*n* = 4) and *Bmpr1a* KO (*n* = 5) mice. **d** Volcano plot comparing microarray data of uninfected *Bmpr1a* KO vs WT antral tissue. Antimicrobial genes are highlighted. **e-f** GSEA of microarray data comparing antral tissues from uninfected *Bmpr1a* KO and WT mice, for response-to-bacterium (**e**), and antimicrobial peptide (**f**) gene signatures (*n* = 2 mcie per group). **g** mRNA expression of the antimicrobial genes *Reg3b, Reg3g,* and *Duox2* in murine gastric organoids cultured in standard medium containing the BMP inhibitor Noggin (+BMPi) or in Noggin-deprived medium supplemented with BMP2 (+BMP2) (*n* = 3 biological replicates per group). Data are presented as mean ± SEM. Statistical analyses were performed using Student's *t*-test (two-tailed) for **a-c** and **g**; using unadjusted *p*-values of permutation test provided by R package fgsea for **e**-**f**. Source data are provided as a Source Data file.

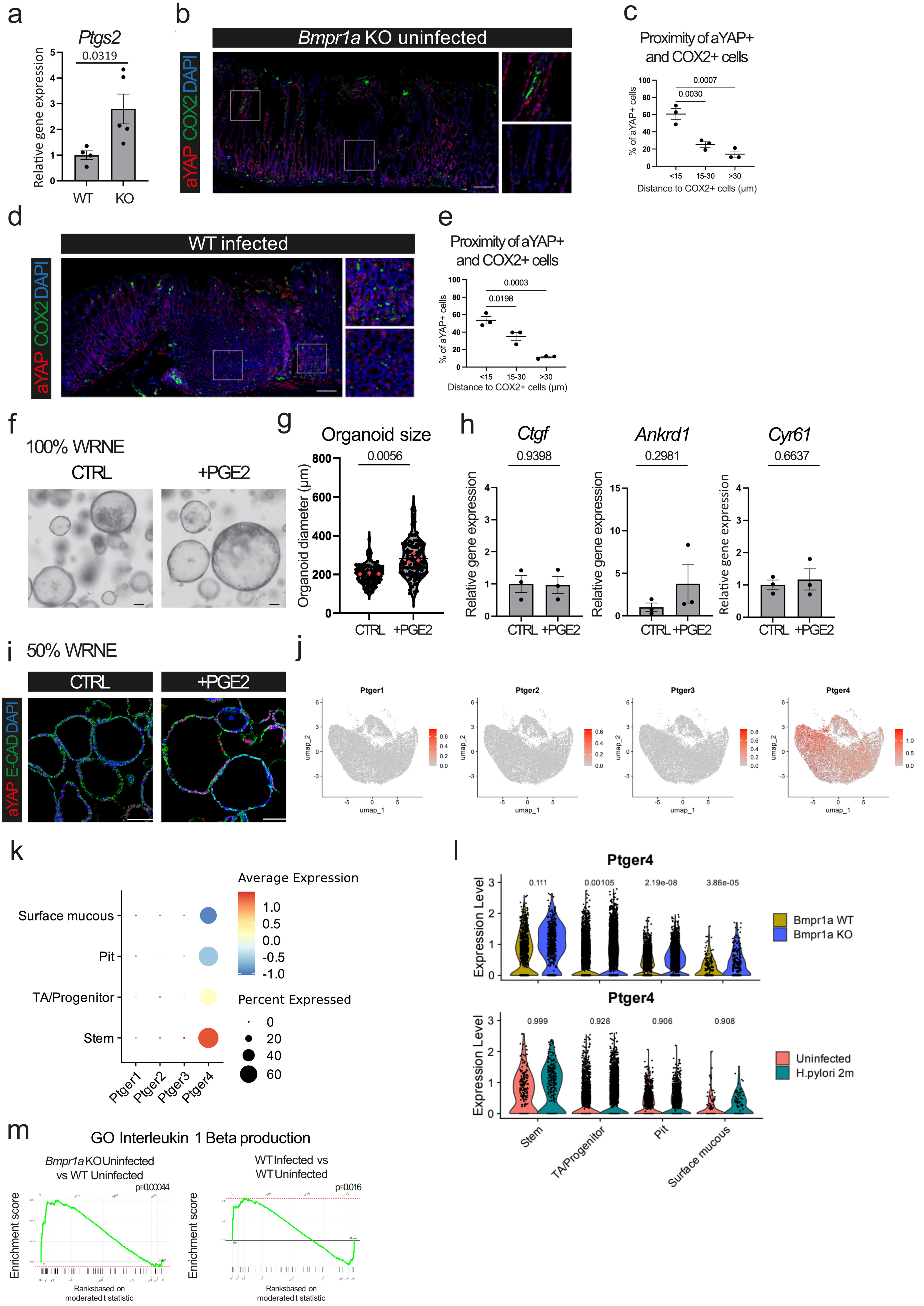

a
Ptgs2
0.0319
Relative gene expression
WT
KO
b
Bmpr1a KO uninfected
aYAP COX2 DAPI
c
Proximity of aYAP+ and COX2+ cells
0.0007
0.0030
% of aYAP+ cells
<15
15-30
>30
Distance to COX2+ cells (μm)
d
WT infected
aYAP COX2 DAPI
e
Proximity of aYAP+ and COX2+ cells
0.0003
0.0198
% of aYAP+ cells
<15
15-30
>30
Distance to COX2+ cells (μm)
f
100% WRNE
CTRL
+PGE2
g
Organoid size
0.0056
Organoid diameter (μm)
CTRL
+PGE2
h
Ctgf
0.9398
Ankrd1
0.2981
Cyr61
0.6637
Relative gene expression
CTRL
+PGE2
i
50% WRNE
CTRL
+PGE2
aYAP E-CAD DAPI
j
Ptger1
Ptger2
Ptger3
Ptger4
umap_1
umap_2
k
Average Expression
Percent Expressed
Surface mucous
Pit
TA/Progenitor
Stem
Ptger1
Ptger2
Ptger3
Ptger4
l
Ptger4
0.111
0.00105
2.19e-08
3.86e-05
Expression Level
Bmpr1a WT
Bmpr1a KO
Ptger4
0.999
0.928
0.906
0.908
Uninfected
H.pylori 2m
Stem
TA/Progenitor
Pit
Surface mucous
m
GO Interleukin 1 Beta production
Bmpr1a KO Uninfected vs WT Uninfected
p=0.00044
WT Infected vs WT Uninfected
p=0.016
Enrichment score
Ranks based on moderated t statistic

Supplementary Fig. 8 Stromal PGE2 activates YAP in gastric epithelial cells

a mRNA expression of *Ptgs2* in WT (*n* = 4 mice) and *Bmpr1a* KO (*n* = 5 mice) uninfected antral gastric tissues. **b-e** Confocal images and proximity analysis of aYAP+ cells and COX2+ cells in uninfected *Bmpr1a* KO (**b-c**) and infected WT (**d-e**) gastric tissues (*n* = 3 mice per group). **f** Brightfield images of WT gastric antral organoids grown for 4 days in standard medium (100%WRNE) with or without PGE2. Scale bars: 100 µm. The experiment was performed three times independently with similar results. **g** Quantification of organoid diameter. The mean values for each biological replicate are highlighted in red (*n* = 3 biological replicates per group). **h** mRNA expression analysis of the YAP target genes *Ctgf*, *Ankrd1* and *Cyr61* in WT organoids grown in standard medium with or without PGE2 (*n* = 3 biological replicates per group). **i** Confocal microscopy images of E-CAD+ aYAP+ cells in control and PGE2-treated gastric organoids grown in 50%WRNE medium. Scale bars: 100 µm. **j** UMAP plots of scRNA-seq showing expression levels of PGE2 receptors (*Ptger1-4*) in antral epithelial cells. **k** Dot plot showing the expression of *Ptger1-4* across cell subpopulations in the murine antral epithelium. Circle size indicates the within-cluster probability of gene detection; fill color represents the normalized average expression level. **l** Violin plot showing the expression level of *Ptger4* in antral epithelial cells from uninfected *Bmpr1a* KO vs. WT mice, as well as from infected WT vs. uninfected WT mice. Numbers at the top indicate p-values for mean differences between conditions for each cluster (*n* = 2 mice per group), derived from genome-wide pseudo-bulk differential expression analysis of antral scRNA data. **m** GSEA of microarray data comparing antral tissues from uninfected *Bmpr1a* KO vs. WT mice, as well as from infected WT vs. uninfected WT mice, for IL-1β production gene signatures (*n* = 2 mice per group). Data are presented as mean ± SEM. Statistical analyses were performed using Student's *t*-test (two-tailed) for **a** and **g-h**; using one-way ANOVA, followed by Tukey's multiple comparisons test for **c** and **e**; using unadjusted *p*-values of permutation test provided by R package fgsea for **m**. Images are representative of at least three biological replicates. Source data are provided as a Source Data file.

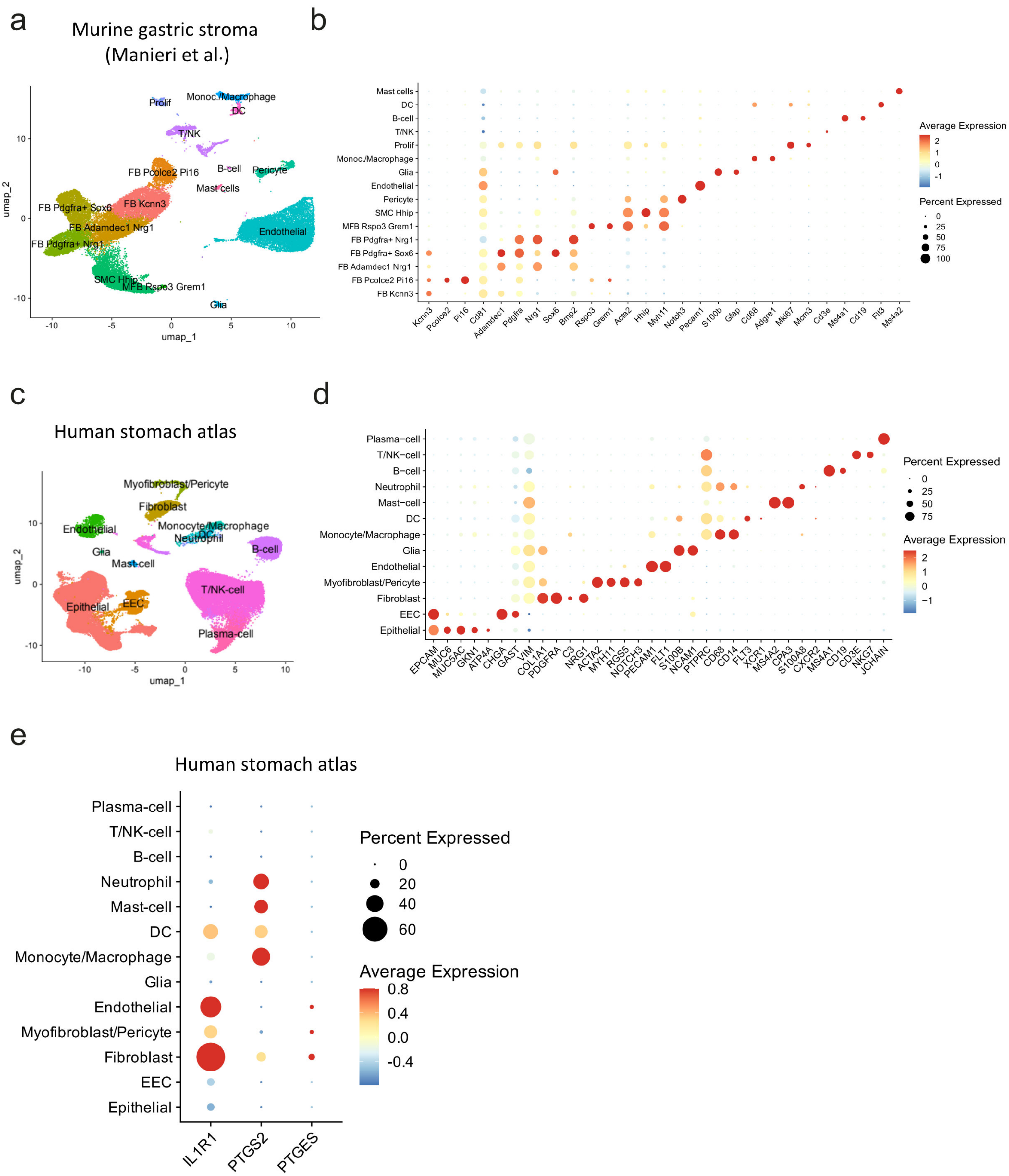
a
Murine gastric stroma
(Manieri et al.)
Prolif
Monoc./Macrophage
DC
T/NK
FB Pcolce2 Pi16
B-cell
Pericyte
Mast cells
FB Pdgfra+ Sox6
FB Kcnn3
FB Adamdec1 Nrg1
FB Pdgfra+ Nrg1
Endothelial
SMC Hhip
MFB Rspo3 Grem1
Glia
umap_1
umap_2
b
Mast cells
DC
B-cell
T/NK
Prolif
Monoc./Macrophage
Glia
Endothelial
Pericyte
SMC Hhip
MFB Rspo3 Grem1
FB Pdgfra+ Nrg1
FB Pdgfra+ Sox6
FB Adamdec1 Nrg1
FB Pcolce2 Pi16
FB Kcnn3
Kcnn3
Pcolce2
Pi16
Cd81
Adamdec1
Pdgfra
Nrg1
Sox6
Bmp2
Rspo3
Grem1
Acta2
Hhip
Myh11
Notch3
Pecam1
S100b
Gfap
Cd68
Adgre1
Mki67
Mcm3
Cd3e
Ms4a1
Cd19
Flt3
Ms4a2
Average Expression
Percent Expressed
c
Human stomach atlas
Myofibroblast/Pericyte
Fibroblast
Endothelial
Monocyte/Macrophage
Neutrophil
DC
Glia
B-cell
Mast-cell
T/NK-cell
Epithelial
EEC
Plasma-cell
umap_1
umap_2
d
Plasma−cell
T/NK−cell
B−cell
Neutrophil
Mast−cell
DC
Monocyte/Macrophage
Glia
Endothelial
Myofibroblast/Pericyte
Fibroblast
EEC
Epithelial
EPCAM
MUC6
MUC5AC
GKN1
ATP4A
CHGA
GAST
VIM
COL1A1
PDGFRA
C3
NRG1
ACTA2
MYH11
RGS5
NOTCH3
PECAM1
FLT1
S100B
NCAM1
PTPRC
CD68
CD14
FLT3
XCR1
MS4A2
CPA3
S100A8
CXCR2
MS4A1
CD19
CD3E
NKG7
JCHAIN
Percent Expressed
Average Expression
e
Human stomach atlas
Plasma-cell
T/NK-cell
B-cell
Neutrophil
Mast-cell
DC
Monocyte/Macrophage
Glia
Endothelial
Myofibroblast/Pericyte
Fibroblast
EEC
Epithelial
IL1R1
PTGS2
PTGES
Percent Expressed
Average Expression

Supplementary Fig. 9 scRNA-seq of murine gastric stroma and human stomach tissue

a UMAP showing cell subsets in the stromal compartment from healthy murine WT stomach tissue. **b** Dot plot showing the expression of cluster marker genes across identified subpopulations in the gastric stroma. Circle size indicates the within-cluster proportion of gene detection; fill color represents the scaled average expression level. **c** UMAP showing cell subsets from human stomach tissue. **b** Dot plot showing the expression of cluster marker genes across identified subpopulations in human stomach tissue. **e** Dot plot showing the expression of *IL1R1*, *PTGS2*, and *PTGES* in cell subpopulations from human stomach tissue.

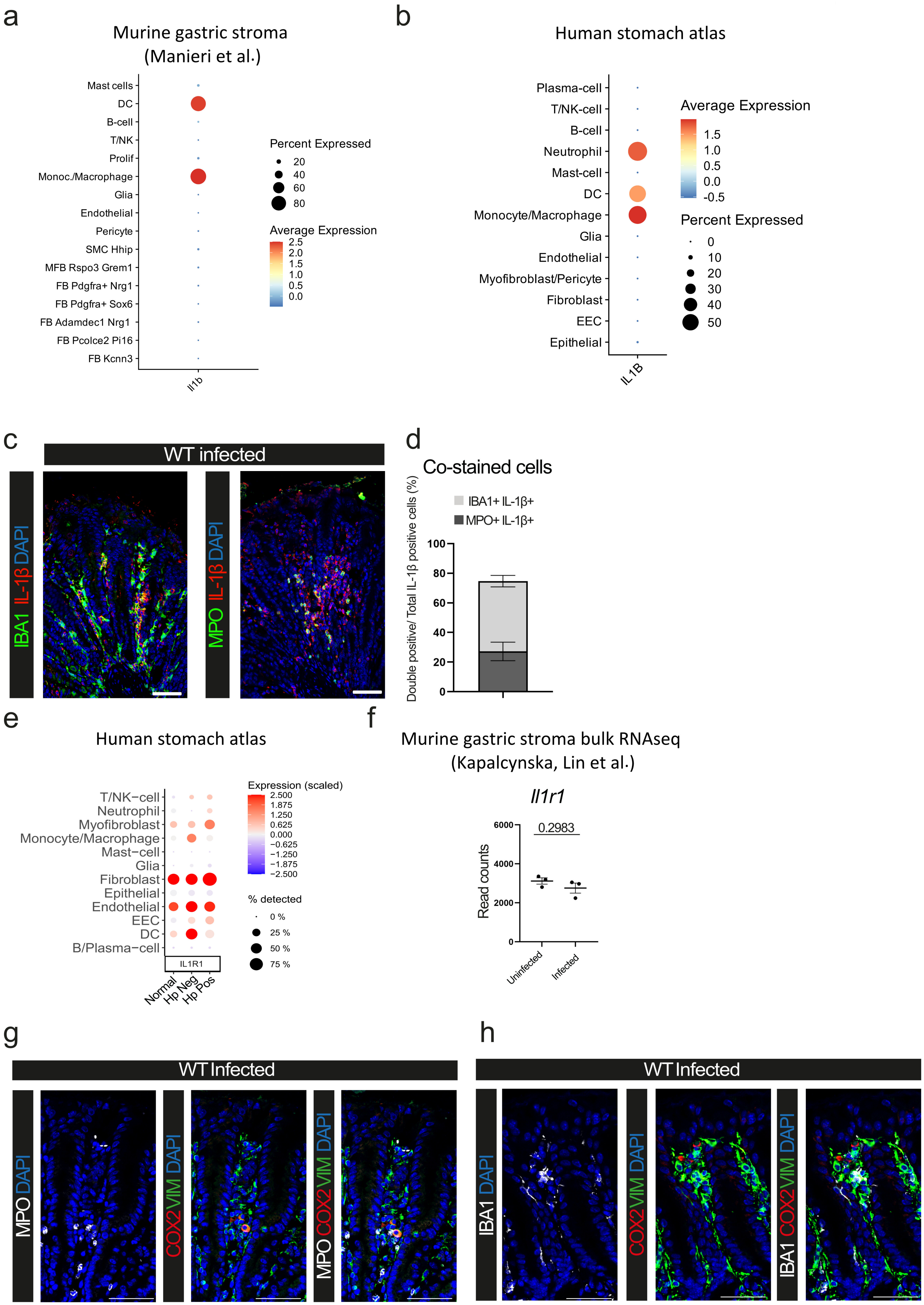
a
Murine gastric stroma
(Manieri et al.)
Mast cells
DC
B-cell
T/NK
Prolif
Monoc./Macrophage
Glia
Endothelial
Pericyte
SMC Hhip
MFB Rspo3 Grem1
FB Pdgfra+ Nrg1
FB Pdgfra+ Sox6
FB Adamdec1 Nrg1
FB Pcolce2 Pi16
FB Kcnn3
Il1b
Percent Expressed
20
40
60
80
Average Expression
2.5
2.0
1.5
1.0
0.5
0.0
b
Human stomach atlas
Plasma-cell
T/NK-cell
B-cell
Neutrophil
Mast-cell
DC
Monocyte/Macrophage
Glia
Endothelial
Myofibroblast/Pericyte
Fibroblast
EEC
Epithelial
IL1B
Average Expression
1.5
1.0
0.5
0.0
-0.5
Percent Expressed
0
10
20
30
40
50
c
WT infected
IBA1 IL-1β DAPI
MPO IL-1β DAPI
d
Co-stained cells
IBA1+ IL-1β+
MPO+ IL-1β+
Double positive/ Total IL-1β positive cells (%)
100
80
60
40
20
0
e
Human stomach atlas
T/NK−cell
Neutrophil
Myofibroblast
Monocyte/Macrophage
Mast−cell
Glia
Fibroblast
Epithelial
Endothelial
EEC
DC
B/Plasma−cell
IL1R1
Normal
Hp Neg
Hp Pos
Expression (scaled)
2.500
1.875
1.250
0.625
0.000
−0.625
−1.250
−1.875
−2.500
% detected
0 %
25 %
50 %
75 %
f
Murine gastric stroma bulk RNAseq
(Kapalcynska, Lin et al.)
Il1r1
0.2983
Read counts
6000
4000
2000
0
Uninfected
Infected
g
WT Infected
MPO DAPI
COX2 VIM DAPI
MPO COX2 VIM DAPI
h
WT Infected
IBA1 DAPI
COX2 VIM DAPI
IBA1 COX2 VIM DAPI

Supplementary Fig. 10 Analysis of IL-1β-producing cells and their interaction with stromal cells

a-b Dot plot showing the expression of IL-1β in cell subpopulations from murine (**a**) and human (**b**) stomach tissues. **c** Confocal microscopy images of co-staining of IL-1β with IBA1 or MPO in antral gastric tissue from infected WT mice. Scale bars: 50 µm. **d** IL-1β+MPO+ (*n* = 4 mice) or IL-1β+IBA1+ (*n* = 3 mice) co-stained cells normalized to the total IL-1β+ cells per section, expressed in percentage. **e** Dot plot of human stomach scRNA-seq showing the cell populations that express *IL1R1* under normal and gastritis (*H. pylori*-negative or -positive) conditions. **f** The expression level of *Il1r1* in the bulk RNA-seq dataset of murine stromal cells under uninfected and infected conditions (*n* = 3 mice per group). **g-h** Confocal microscopy images of COX2, VIM, and MPO, and images of COX2, VIM, and IBA1 in antral gastric tissue from infected WT mice. Scale bars: 50 µm. Data are presented as mean ± SEM. Statistical analyses were performed using Student's *t*-test (two-tailed) for **f**. Images are representative of at least three biological replicates. Source data are provided as a Source Data file.

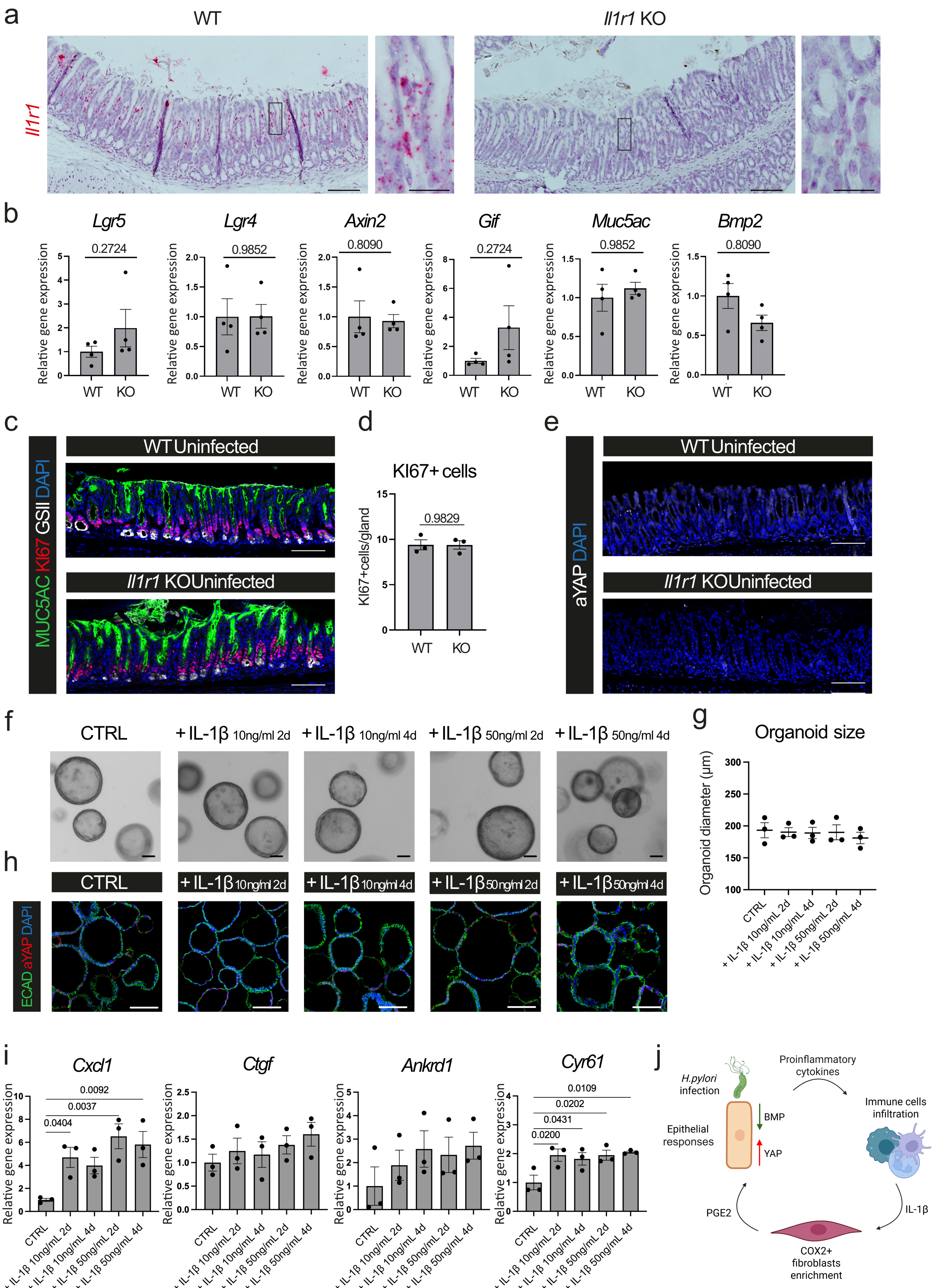
a
WT
Il1r1 KO
Il1r1
b
Lgr5
Lgr4
Axin2
Gif
Muc5ac
Bmp2
0.2724
0.9852
0.8090
0.2724
0.9852
0.8090
Relative gene expression
WT
KO
c
WT Uninfected
Il1r1 KOUninfected
MUC5AC KI67 GSII DAPI
d
KI67+ cells
0.9829
KI67+cells/gland
WT
KO
e
WT Uninfected
Il1r1 KOUninfected
aYAP DAPI
f
CTRL
+ IL-1β 10ng/ml 2d
+ IL-1β 10ng/ml 4d
+ IL-1β 50ng/ml 2d
+ IL-1β 50ng/ml 4d
g
Organoid size
Organoid diameter (μm)
CTRL
+ IL-1β 10ng/mL 2d
+ IL-1β 10ng/mL 4d
+ IL-1β 50ng/mL 2d
+ IL-1β 50ng/mL 4d
h
CTRL
+ IL-1β 10ng/ml 2d
+ IL-1β 10ng/ml 4d
+ IL-1β 50ng/ml 2d
+ IL-1β 50ng/ml 4d
ECAD aYAP DAPI
i
Cxcl1
Ctgf
Ankrd1
Cyr61
0.0092
0.0037
0.0404
0.0109
0.0202
0.0431
0.0200
Relative gene expression
CTRL
+ IL-1β 10ng/mL 2d
+ IL-1β 10ng/mL 4d
+ IL-1β 50ng/mL 2d
+ IL-1β 50ng/mL 4d
j
H.pylori
infection
Proinflammatory
cytokines
Epithelial
responses
BMP
YAP
Immune cells
infiltration
PGE2
IL-1β
COX2+
fibroblasts
enrichment

Supplementary Fig. 11 Characterization of *Il1r1* KO mouse model and the direct effect of IL-1β on epithelial cells

a ISH images of *Il1r1* in tissue from uninfected WT and *Il1r1* KO mice. Scale bars: 100 µm, inserts: 20 µm. **b** mRNA expression of *Lgr5*, *Lgr4*, *Axin2*, *Gif*, *Muc5ac*, and *Bmp2* in uninfected WT and *Il1r1* KO antral gastric tissue (*n* = 4 mice per group). **c** Confocal microscopy images of MUC5AC, KI67, GSII, and DAPI staining in antral gastric tissue from uninfected WT and *Il1r1* KO mice. Scale bars: 100 µm. **d** Quantification of KI67+ cells in antral gastric glands of uninfected WT and *Il1r1* KO mice (*n* = 3 mice per group). **e** Confocal microscopy images of aYAP and DAPI staining in antral gastric tissue from uninfected WT and *Il1r1* KO mice. Scale bars: 100 µm. **f-g** Brightfield images and diameter quantification of gastric organoids grown for 4 days in 50% WRNE medium following IL-1β treatment at the indicated concentrations and time points (*n* = 3 biological replicates per group). Scale bars: 100 µm. The experiment was performed three times independently with similar results. **h** Confocal microscopy images of E-CAD+ aYAP+ cells in control and IL-1β-treated organoids grown in 50%WRNE medium. Scale bars: 100 µm. The experiment was performed three times independently with similar results. **i** mRNA expression of NF-κB target genes *Cxcl1* and YAP target genes *Ctgf, Ankrd1,* and *Cyr61* in control and IL-1β-treated organoids grown in 50%WRNE medium (*n* = 3 biological replicates per group). **j** Schematic representation of the epithelial-immune-stromal crosstalk during *H. pylori* infection. Created in BioRender. Müllerke, S. (2026) https://BioRender.com/tuugyrh. Infection downregulates BMP activity in gastric epithelial cells, driving increased production of proinflammatory cytokines that recruit IL-1β-producing immune cells. This immune infiltration leads to the enrichment of COX2+ fibroblasts via IL-1β signaling. The resulting stromal PGE2 is sensed by neighboring epithelial cells, triggering YAP activation and fetal-like transcriptional responses. Data are presented as mean ± SEM. Statistical analyses were performed using Student's *t*-test (two-tailed) for **b** and **d**; using one-way ANOVA, followed by Tukey's multiple comparisons test for **g** and **i**. Images are representative of at least three biological replicates. Source data are provided as a Source Data file.
