# Supplementary material for: Helicobacter pylori triggers gastric mucosal remodeling toward a fetal-like transcriptional program via stromal IL-1β signaling
Source: Nat Commun. 2026 Sep 5;17:9510. doi: 10.1038/s41467-026-77520-1 (PMC13546300; doi:10.1038/s41467-026-77520-1)
Supplement: Supplementary file 2 [file 41467_2026_77520_MOESM2_ESM.pdf]

Description of Additional Supplementary Files

File Name: Supplementary Data 1

Description: Cell counts, markers and expected gland location of epithelial subsets from the murine gastric epithelium scRNA-seq dataset.

File Name: Supplementary Data 2

Description: DEG analysis results for mouse antral epithelial cell populations.

File Name: Supplementary Data 3

Description: qPCR primers.
