# Supplementary material for: Helicobacter pylori triggers gastric mucosal remodeling toward a fetal-like transcriptional program via stromal IL-1β signaling
Source: Nat Commun. 2026 Sep 5;17:9510. doi: 10.1038/s41467-026-77520-1 (PMC13546300; doi:10.1038/s41467-026-77520-1)
Supplement: Supplementary file 3 [file 41467_2026_77520_MOESM6_ESM.pdf]

Corresponding author(s): Michael Sigal

Last updated by author(s): Aug 5, 2026

Reporting Summary

Nature Portfolio wishes to improve the reproducibility of the work that we publish. This form provides structure for consistency and transparency in reporting. For further information on Nature Portfolio policies, see our Editorial Policies and the Editorial Policy Checklist.

Statistics

For all statistical analyses, confirm that the following items are present in the figure legend, table legend, main text, or Methods section.

n/a	Confirmed	
☐	☒	The exact sample size (n) for each experimental group/condition, given as a discrete number and unit of measurement
☐	☒	A statement on whether measurements were taken from distinct samples or whether the same sample was measured repeatedly
☐	☒	The statistical test(s) used AND whether they are one- or two-sided<br>*Only common tests should be described solely by name; describe more complex techniques in the Methods section.*
☒	☐	A description of all covariates tested
☐	☒	A description of any assumptions or corrections, such as tests of normality and adjustment for multiple comparisons
☐	☒	A full description of the statistical parameters including central tendency (e.g. means) or other basic estimates (e.g. regression coefficient) AND variation (e.g. standard deviation) or associated estimates of uncertainty (e.g. confidence intervals)
☐	☒	For null hypothesis testing, the test statistic (e.g. F, t, r) with confidence intervals, effect sizes, degrees of freedom and P value noted<br>*Give P values as exact values whenever suitable.*
☒	☐	For Bayesian analysis, information on the choice of priors and Markov chain Monte Carlo settings
☒	☐	For hierarchical and complex designs, identification of the appropriate level for tests and full reporting of outcomes
☒	☐	Estimates of effect sizes (e.g. Cohen's d, Pearson's r), indicating how they were calculated

Our web collection on statistics for biologists contains articles on many of the points above.

Software and code

Policy information about availability of computer code

Data collection	QuantStudioTM Design and Analysis software (version 1.4.3, Applied Biosystems)<br>Leica Application Suite X (LAS X, v3.7.4.23463)<br>10x CellRanger v9.0.1<br>Image Analysis/Feature Extraction software G2567AA 12.2.07 (Agilent Technologies)
Data analysis	Fiji/ImageJ (1.53c)<br>R 4.5.2<br>R packages (Seurat v5.0.1, UCell, DESeq2, fgsea, MsigDB v7.1)<br>GraphPad Prism 10.3.1<br>QuPath (v0.6.0)

For manuscripts utilizing custom algorithms or software that are central to the research but not yet described in published literature, software must be made available to editors and reviewers. We strongly encourage code deposition in a community repository (e.g. GitHub). See the Nature Portfolio guidelines for submitting code & software for further information.

Data

Policy information about availability of data

All manuscripts must include a data availability statement. This statement should provide the following information, where applicable:

- Accession codes, unique identifiers, or web links for publicly available datasets
- A description of any restrictions on data availability
- For clinical datasets or third party data, please ensure that the statement adheres to our policy

The data for GSEA of the microarray data in this manuscript from uninfected Bmpr1a KO and WT antral tissue can be accessed under the Gene Expression Omnibus database (GSE283811). The data of microarray analysis from infected Il1r1 KO and WT antral tissue can be accessed under the Gene Expression Omnibus database (GSE283927). The data of microarray analysis from primary stromal cells treated with IL1-β can be accessed under the Gene Expression Omnibus database (GSE302280). The bulk RNA-seq data for WT infected vs uninfected antral tissue 20 can be accessed via the Gene Expression Omnibus database (GSE136658). The scRNA-seq data for primary gastric epithelial cells can be accessed in the Gene Expression Omnibus (GSE319373). Source data are provided with this paper.

All computational codes can be accessed at https://github.com/Sigal-Lab/Beccaceci_Stomach_Bmp1r_KO, https://github.com/Sigal-Lab/Beccaceci_Stomach_Il1r_KO, and https://github.com/Sigal-Lab/Beccaceci_Il1b_stroma.

Research involving human participants, their data, or biological material

Policy information about studies with human participants or human data. See also policy information about sex, gender (identity/presentation), and sexual orientation and race, ethnicity and racism.

Reporting on sex and gender	NA
Reporting on race, ethnicity, or other socially relevant groupings	NA
Population characteristics	NA
Recruitment	NA
Ethics oversight	NA

Note that full information on the approval of the study protocol must also be provided in the manuscript.

Field-specific reporting

Please select the one below that is the best fit for your research. If you are not sure, read the appropriate sections before making your selection.

☒ Life sciences ☐ Behavioural & social sciences ☐ Ecological, evolutionary & environmental sciences

For a reference copy of the document with all sections, see nature.com/documents/nr-reporting-summary-flat.pdf

Life sciences study design

All studies must disclose on these points even when the disclosure is negative.

Sample size	No statistics were done to predetermine sample sizes. The number of mice per group was used based on availability of mice of the respective mouse line ans was based on previous experience
Data exclusions	No data were excluded from the analyses.
Replication	Experiments were performed at least three times independently with similar results, using a minimum of three biological replicates in total, unless stated otherwise.
Randomization	The mice and assembloids/organoids/stromal cells were randomized for respective treatments.
Blinding	Group allocation and analysis were done in a blinded fashion.

Reporting for specific materials, systems and methods

We require information from authors about some types of materials, experimental systems and methods used in many studies. Here, indicate whether each material, system or method listed is relevant to your study. If you are not sure if a list item applies to your research, read the appropriate section before selecting a response.

Materials & experimental systems

n/a	Involved in the study
☐	☒ Antibodies
☒	☐ Eukaryotic cell lines
☒	☐ Palaeontology and archaeology
☐	☒ Animals and other organisms
☒	☐ Clinical data
☒	☐ Dual use research of concern
☒	☐ Plants

Methods

n/a	Involved in the study
☒	☐ ChIP-seq
☐	☒ Flow cytometry
☒	☐ MRI-based neuroimaging

Antibodies

Antibodies used

The following antibodies were used: mouse anti-COX2 (BD Biosciences, 610204, 1:150), mouse anti-E-cadherin (clone 36) (BD, 610181, 1:200), Alexa Fluor 647-conjugated lectin GSII (Thermo Scientific, L32451, 1:200), rabbit anti-IBA1 (Fujifilm Wako Chemicals, 019-19741, 1:500), rabbit anti-IL-1β (WH295161) (ABClonal, A11370, 1:150), rabbit anti-KI67 (D3B5) (Cell Signaling Technology, 9129S, 1:200); mouse anti-MUC5AC (45M1) (Invitrogen, 12178, 1:200), rabbit anti-MPO (Abcam, ab208670, 1:200), goat anti-MPO (R&D, AF3667, 1:200), rabbit anti-vimentin (D21H3) (Cell Signaling Technology, 5741S, 1:200); rat anti-vimentin (R&D Systems, MAB2105, 1:200), rabbit anti-active YAP1 (Abcam, ab205270, clone EPR19812, 1:150); goat anti-mouse Alexa Fluor 488 (Jackson Immunoresearch, catalog 115-545-003, 1:300), and goat anti-rabbit Cy3 (Jackson Immunoresearch, catalog 115-165-146, 1:300), donkey anti-rabbit Alexa Fluor 488 (Jackson Immunoresearch, catalog 711-545-152, 1:300), donkey anti-mouse Cy3 (Jackson Immunoresearch, catalog 715-166-020 1:300).
p-IKKα/β (CST #2697, 1:1000 in 5% BSA), IKKα (CST #2682, 1:1000 in 5% BSA), p-IκBα (CST, #9246, 1:1000 in 5% milk), IκBα (CST, #4812, 1:1000 in 5% BSA), p-SMAD1/5 (CST, #9516, 1:1000 in 5% milk), SMAD1 (CST, #6944, 1:1000 in 5% milk), p-YAP (CST, #53749, 1:1000 in 5% milk), YAP (CST, # 14074, 1:1000 in 5% BSA),p100 (CST #4882, 1:1000 in 5% BSA), p-p100 (CST, #4810, 1:1000 in 5% BSA) and GAPDH (Millipore MAB374, 1:5000 in 5% milk).
PE-conjugated rat anti-CD326 (EpCAM) (Miltenyi Biotec, 130-117-779, clone caa7-9G8, 1:200), APC-Cy7-conjugated rat anti-CD45 (BD, 557659, clone 30-F11, 1:200), and Fixable Viability Stain 450 (FVS-450) (BD, 562247, 1:2000)

Validation

All antibodies used in the study were commercially available and validated by the manufacturer for respective applications. We used for all antibodies secondary only controls. Furthermore, internal validation was performed in stomach or intestine tissue to confirm the correct pattern and specificity of staining.

Validation information of commercial antibodies used in the study:
mouse anti-COX2 (BD Biosciences, 610204), https://www.bdbiosciences.com/en-de/products/reagents/microscopy-imaging-reagents/immunohistochemistry-reagents/purified-mouse-anti-cox-2.610204

mouse anti-E-cadherin (BD Biosciences, 610181), https://www.bdbiosciences.com/en-de/products/reagents/microscopy-imaging-reagents/immunohistochemistry-reagents/purified-mouse-anti-e-cadherin.610181

Alexa Fluor 647-conjugated lectin GSII (Thermo Scientific, L32451), https://www.thermofisher.com/order/catalog/product/L32451

rabbit anti-IBA1 (Fujifilm Wako, 019-19741), https://labchem-wako.fujifilm.com/us/product/detail/019-19741.html

rabbit anti-IL-1β (ABClonal, A11370), https://static.abclonal.com/datasheet/A11370.pdf

rabbit anti-KI67 (Cell Signaling Technology, 9129S), https://www.cellsignal.com/products/primary-antibodies/ki-67-d3b5-rabbit-monoclonal-antibody/9129

mouse anti-MUC5AC (Invitrogen, 12178), https://www.thermofisher.com/order/catalog/product/MA5-12178

rabbit anti-MPO (Abcam, ab208670), https://www.abcam.com/en-de/products/primary-antibodies/ab208670.html

goat anti-MPO (R&D Systems, AF3667), https://www.rndsystems.com/products/human-myeloperoxidase-mpo-antibody_af3667

rabbit anti-vimentin (Cell Signaling Technology, 5741S), https://www.cellsignal.com/products/primary-antibodies/vimentin-d21h3-xp-rabbit-monoclonal-antibody/5741

rat anti-vimentin (R&D Systems, MAB2105), https://www.rndsystems.com/products/mouse-vimentin-antibody-286206_mab2105

rabbit anti-active YAP1 (Abcam, ab205270), https://www.abcam.com/en-de/products/primary-antibodies/ab205270.html

goat anti-mouse Alexa Fluor 488 (Jackson Immunoresearch, 115-545-003), https://www.jacksonimmuno.com/catalog/products/115-545-003

goat anti-rabbit Cy3 (Jackson Immunoresearch, 115-165-146), https://www.jacksonimmuno.com/catalog/products/115-165-146

donkey anti-rabbit Alexa Fluor 488 (Jackson Immunoresearch, 711-545-152), https://www.jacksonimmuno.com/catalog/products/711-545-152

donkey anti-mouse Cy3 (Jackson Immunoresearch, 715-166-020), https://www.jacksonimmuno.com/catalog/products/715-166-020

p-IKKα/β (Cell Signaling Technology, 2697), https://www.cellsignal.com/products/primary-antibodies/phospho-ikkalpha-beta-ser176-180-16a6-rabbit-monoclonal-antibody/2697

IKKα (Cell Signaling Technology, 2682), https://www.cellsignal.com/products/primary-antibodies/ikkalpha-3g12-mouse-monoclonal-antibody/2682

p-IκBα (Cell Signaling Technology, 9246), https://www.cellsignal.com/products/primary-antibodies/phospho-ikbalpha-ser32-36-5a5-mouse-monoclonal-antibody/9246

IκBα (Cell Signaling Technology, 4812), https://www.cellsignal.com/products/primary-antibodies/ikbalpha-44d4-rabbit-monoclonal-antibody/4812

p-SMAD1/5 (Cell Signaling Technology, 9516), https://www.cellsignal.com/products/primary-antibodies/phospho-smad1-ser463-465-smad5-ser463-465-smad9-ser465-467-d5b10-rabbit-monoclonal-antibody/9516

SMAD1 (Cell Signaling Technology, 6944), https://www.cellsignal.com/products/primary-antibodies/smad1-d59d7-xp-rabbit-monoclonal-antibody/6944

p-YAP (Cell Signaling Technology, 53749),
https://www.cellsignal.com/products/primary-antibodies/phospho-yap-ser109-e5i9g-rabbit-monoclonal-antibody/53749

YAP (Cell Signaling Technology, 14074), https://www.cellsignal.com/products/primary-antibodies/yap-d8h1x-rabbit-monoclonal-antibody/14074

p100 (Cell Signaling Technology, 4882), https://www.cellsignal.com/products/primary-antibodies/nf-kappab2-p100-p52-antibody/4882

p-p100 (Cell Signaling Technology, 4810), https://www.cellsignal.com/products/primary-antibodies/phospho-nf-kappab2-p100-ser866-870-antibody/4810

GAPDH (EMD Millipore, MAB374), https://www.sigmaaldrich.com/DE/en/product/mm/mab374

PE-conjugated rat anti-CD326 (EpCAM) (Miltenyi Biotec, 130-117-779), https://www.miltenyibiotec.com/DE-en/products/cd326-epcam-antibody-anti-mouse-caa7-9g8.html#Conjugate=PE:size=30-ug-in-200-uL

APC-Cy7-conjugated rat anti-CD45 (BD Biosciences, 557659), https://www.bdbiosciences.com/en-de/products/reagents/flow-cytometry-reagents/research-reagents/single-color-antibodies-ruo/apc-cy-7-rat-anti-mouse-cd45.557659

Fixable Viability Stain 450 (FVS-450) (BD Biosciences, 562247), https://www.bdbiosciences.com/en-de/products/reagents/flow-cytometry-reagents/research-reagents/single-color-antibodies-ruo/fixable-viability-stain-450.562247

Animals and other research organisms

Policy information about studies involving animals; ARRIVE guidelines recommended for reporting animal research, and Sex and Gender in Research

Laboratory animals	C57BL/6 mice were obtained from Charles River Laboratory. Axin2CreErt2Bmpr1afl/fl (epithelial-specific Bmpr1a KO) mice were generated in our lab previously. Il1r1 fl/flCol1a2CreErt2 (stromal-specific Il1r1 KO) mice were generated by crossing Il1r1 fl/fl mice with Col1a2CreErt2 mice.
Wild animals	No wild animals were used in the study.
Reporting on sex	Male and female mice were used for Il1r1 fl/flCol1a2CreErt2 mouse experiments, and they were randomly allocated to experimental groups. Male mice were used for other experiments in this study.
Field-collected samples	No filed-collected samples were used in the study.
Ethics oversight	All animal experiments were performed under guidance and approval of institutional, local, and national legal authorities (Landesamt für Gesundheit und Soziales (LaGeSo) Berlin, licenses G0084_17 and G0144_22).

Note that full information on the approval of the study protocol must also be provided in the manuscript.

Plants

Seed stocks	NA
Novel plant genotypes	NA
Authentication	NA

Flow Cytometry

Plots

Confirm that:

☒ The axis labels state the marker and fluorochrome used (e.g. CD4-FITC).

☒ The axis scales are clearly visible. Include numbers along axes only for bottom left plot of group (a 'group' is an analysis of identical markers).

☒ All plots are contour plots with outliers or pseudocolor plots.

☒ A numerical value for number of cells or percentage (with statistics) is provided.

Methodology

Sample preparation	Mouse gastric tissue was isolated and incubated in PBS with 0.5 mM DTT and 10 mM EDTA for 20 minutes at 37 °C. Gastric glands were mechanically dissociated from the tissue and washed with PBS. Gastric glands were collected and treated with TrypLE at 37 °C for 8 minutes, in order to obtain a single-cell suspension.
Instrument	FACS Aria III (BD)
Software	FACSDiva 9.4
Cell population abundance	The purity of cells was verified by re-sorting of sorted cells during the establishment of the sorting protocol for the experiments presented here and was confirmed to be more than 95%.
Gating strategy	Single cells were gated based on SSC-A, FSC-A, FSC-H, and SSC-H; live cells were identified by negative FVS450 staining, and epithelial cells were selected as EpCAM-positive and CD45-negative.

☒ Tick this box to confirm that a figure exemplifying the gating strategy is provided in the Supplementary Information.
