# Supplementary material for: Helicobacter pylori triggers gastric mucosal remodeling toward a fetal-like transcriptional program via stromal IL-1β signaling
Source: Nat Commun. 2026 Sep 5;17:9510. doi: 10.1038/s41467-026-77520-1 (PMC13546300; doi:10.1038/s41467-026-77520-1)
Supplement: Supplementary file 4 [file 41467_2026_77520_MOESM7_ESM.pdf]

nature portfolio

Peer Review File

Helicobacter pylori triggers gastric mucosal remodeling toward a fetal-like transcriptional program via stromal IL-1β signaling

Corresponding Author: Professor Michael Sigal

This file contains all reviewer reports in order by version, followed by all author rebuttals in order by version.

Version 0:

Reviewer comments:

Reviewer #1

(Remarks to the Author)
Sigal lab has demonstrated that Helicobacter pylori infection induces a fetal-like state in the gastric epithelium through mesenchymal PGE_2 production. The authors observed hyperproliferation, YAP activation, and fetal-like gene expression patterns in the gastric epithelium upon H. pylori infection in a Bmpr1a-dependent manner. They further showed that H. pylori-induced NF-κB signaling acts synergistically with BMP inhibition. Using single-cell RNA sequencing, organoid culture, and genetically engineered mouse models, the study suggests that YAP activation is driven by IL-1β–mediated PGE_2 production from intestinal stromal cells. While it is known that immune responses to infection can induce fetal-like states, the underlying mechanisms have remained unclear. This study provides valuable insights into how infection and inflammation drive fetal-like transcriptional and regenerative programs via YAP activation in gut epithelium. I find this manuscript highly interesting and informative. However, several points need to be addressed prior to publication.

Major Comments
Figure 2: niche factor comparison is insufficient.
The comparison between WRN and no WRN conditions is overly simplistic. To better assess the impact of BMP signaling, the authors should compare WRN vs. WR (i.e., excluding Noggin). This distinction is critical, as R-spondin-conditioned medium may contain unknown factors that could confound interpretation. In the Methods section, the authors mention "sWnt (U-Protein Express)"—presumably referring to surrogate Wnt—but this must be clearly defined and described to ensure experimental reproducibility.

Line 282: PGE_2 and Wnt signaling.
The sentence “PGE_2 can activate YAP signaling in gastric epithelial cells that have reduced Wnt signaling activity” is misleading. It remains unclear whether PGE_2 reduces Wnt activity through YAP or through other GPCR-mediated pathways. To support this claim, the authors should block YAP signaling using TEAD inhibitors or genetic inactivation strategies.

BMP2 inhibition of NF-κB activation lacks mechanistic support.
The finding that BMP2 suppresses PAMP-induced NF-κB activation is intriguing, yet the underlying mechanism is not explored. While detailed mechanistic dissection may not be necessary, some additional experiments would strengthen this point. For example, analysis of upstream regulators such as TRAF6 or A20, or modulation of NF-κB activation by cytokines like IL-17 or TNF-α (instead of PAMPs), would be informative.

Line 287: Typo and immunostaining controls.
The sentence “IL-1β is upregulated in in the inter-crypt regions...” contains a typo (“in in”). In addition, the immunolabeling images would benefit from co-staining with lineage-specific markers to verify cellular identity.

Line 297: IL-1β–producing cell types.
The authors state that IL-1β–expressing cells are primarily monocytes/macrophages, neutrophils, and to a lesser extent, dendritic cells. However, this analysis is based on scRNA-seq data from normal human stomach. Since IL1b and IL1r1 expression levels are likely modulated by infection or inflammation, the authors should validate these findings using in situ hybridization (ISH) or immunohistochemistry (IHC) on gastric mucosa with and without H. pylori infection.

Minor Points
GSEA abbreviation
Please spell out “GSEA” at first mention. Also, avoid redundancy—“GSEA analysis” should simply be “GSEA.”

Inconsistent strain names
Line 314 should read: Col1a2-CreERT2 Il1r1^fl/fl^, hereafter referred to as Il1r1^KO^ mice.
Line 324: “KO mice” should be replaced with “Il1r1^KO^ mice” for clarity and consistency.

Reviewer #2

(Remarks to the Author)
I think that your current study presents meaningful insights as a continuation of your previous work on H. pylori-induced inflammation and hyperplasia. Notably, you demonstrated that Bmpr1a KO mice exhibit an expansion of KI67^{+} proliferative cells—mimicking gland hyperplasia observed in the antrum of PMSS1-infected WT mice—even in the absence of infection, which is quite compelling. This also appears to be the first reported instance of a fetal-like regenerative response in the stomach, as indicated by the expansion of the Anxa1^{+}aYAP^{+} compartment following injury.
Additionally, your data clearly show that tissue reprogramming driven by H. pylori infection is regulated via IL-1 signaling in stromal cells, as supported by the use of Il1r KO mice. I agree with your interpretation that crosstalk among epithelial, stromal, and immune cells contributes to the fetal-like regenerative state, and I believe the conditional KO mouse systems you employed are appropriate to investigate this phenomenon.
However, I do have concerns that some key observations remain unconfirmed, and I wonder if additional validation might be possible using the models you previously established in your earlier studies.
1. From what I have seen, this point also seems to be missing in your previous studies related to H. pylori infection. When presenting histopathological images of H. pylori-infected mice, it appears that you have not shown H. pylori colonization on the epithelium. While it's generally assumed that H. pylori are present in the infected group, I believe it is important to provide visual confirmation of the infection status by staining for H. pylori to clearly distinguish between infected and uninfected conditions. This becomes particularly important if you aim to demonstrate that the uninfected Bmpr1a KO mice resemble the pathological features of WT infected mice.
2. The gland length in Bmpr1a KO mice appears to be approximately 1.5 to 2 times greater than that in WT infected mice, and I would appreciate further explanation regarding this observation (Fig. 1a, g, i, Fig. 4b, g). This suggests that hyperplasia may have been induced in the absence of infection, and I believe it is important to consider whether there are potential pitfalls or limitations in the mechanistic interpretation of this finding.
3. According to clinical reports, more than half of individuals infected with H. pylori remain asymptomatic. Given that, is the two-month period following oral infection with 10^8 CFU of H. pylori sufficient to induce chronic infection symptoms in your mouse model?
Alternatively, do you selectively analyze only those mice that exhibit symptoms among the infected group? Some clarification on this aspect of your experimental design would be helpful.
4. I strongly agree with your observation in Figure 2 that fetal-like regeneration and YAP signaling regulation following the loss of BMP signaling during in vivo H. pylori infection cannot be recapitulated in conventional epithelial-only organoid systems, nor can they be fully explained by epithelial cell-intrinsic mechanisms. I was particularly impressed by your group’s previous work on gastrointestinal assembloids that incorporate mesenchymal niches (M Lin, et al., Nat Commun 14.1 (2023): 3025., https://doi.org/10.1038/s41467-023-38780-3), and I would like to suggest considering whether this advanced culture platform could be applied to the current study. If feasible, I believe it could greatly enhance the physiological relevance of the experimental validation.
5. H. pylori infection is known to begin with colonization in the gastric antrum and, over time, lead to the loss of parietal cells in the corpus, thereby increasing the risk of cancer development in the corpus region. In this context, I would appreciate it if you could elaborate on why your study focused specifically on the antrum, and, if available, provide any histopathological data or observations regarding H. pylori-induced pathology in the mouse corpus, along with further explanation of these findings.

Reviewer #3

(Remarks to the Author)
This manuscript by Beccaceci et al. investigates how Helicobacter pylori (H. pylori) infection promotes fetal-like epithelial reprogramming in the gastric antrum through suppression of BMP signaling and activation of an epithelial–immune–stromal signaling cascade. The authors demonstrate that inhibition of BMP signaling—either through infection or epithelial-specific deletion of Bmpr1a—leads to NF-κB–mediated proinflammatory chemokine expression and immune cell infiltration. IL-1β, produced by infiltrating macrophages and neutrophils, induces COX2 and prostaglandin E2 (PGE2) production in stromal cells, which in turn activates YAP signaling in the adjacent epithelium, promoting a regenerative, fetal-like state. Deletion of Il1r1 in stromal cells blunts these changes and prevents H. pylori–induced hyperplasia.
While this study adds potentially valuable mechanistic links between microbial sensing, inflammation, and epithelial regeneration, there are significant concerns regarding the novelty of the findings, the specificity and clarity of the experimental design, the anatomical limitation to the antrum, and the depth of mechanistic validation. Several key conclusions appear overstated based on the current data, and additional experiments are required to substantiate them.
Many of the experiments presented in this study are largely descriptive and somewhat limited in scope. For example, early in the Results section, the authors claim that epithelial Bmpr1a knockout mimics H. pylori infection. However, this conclusion appears overstated, as it is based mainly on histological observations of mucosal hyperplasia and increased proliferation,

along with a few selected bulk RNA-seq snapshots. A more comprehensive and quantitative comparison is necessary to support this claim.

Additionally, key aspects of the proposed mechanism remain insufficiently characterized. For example, the cellular identity of IL-1β–producing immune cells and the specific stromal subtypes responding to IL-1β are not adequately defined. To strengthen the mechanistic claims and provide high-resolution insights into epithelial–immune–stromal interactions, incorporation of single-cell RNA sequencing and/or spatial transcriptomic analysis is strongly recommended.

Major Comments

1. Limited Novelty and Overlap with Prior Work

The role of BMP signaling in gastric epithelial homeostasis and hyperplasia is well documented (Bleuming et al., 2007; Ye et al., 2018; Takabayashi et al., 2023; Huh et al., 2010), including by the authors in prior work using Bmpr1a-deficient models (Kapalczybska et al., 2022). While the involvement of immune and stromal components adds some depth, the conceptual novelty is limited. The authors should clearly define what aspects of the current study are new.

2. Anatomical Scope and Misinterpretation

The study focuses solely on the antrum, yet the Introduction and Discussion cite studies on the corpus and cardia without distinguishing anatomical context. This may lead to misinterpretation. The authors should clearly specify the region of interest throughout and acknowledge that the findings may not generalize to other gastric compartments.

3. Mechanistic Gaps in BMP–NF-κB–YAP Crosstalk

The proposed link between BMP suppression and NF-κB/YAP activation lacks mechanistic clarity. BMP signaling classically proceeds through SMADs, and it is unclear why non-SMAD pathways are activated here. Suggested additional experiments include:

o Assessing SMAD activation in response to BMP2 and testing whether SMAD inhibition alters NF-κB/YAP activation or chemokine expression.

o Western blot of total and phospho-IKKα/β, IκBα, p65, YAP, and upstream regulators (e.g., LATS1/2), with densitometric quantification.

o Time-course and dose-response experiments with BMP2.

o Use of NF-κB and YAP inhibitors or genetic mutants.

4. Lack of Temporal and Spatial Context in Bmpr1a KO Model

The timing and progression of changes following tamoxifen induction are not shown. Time-course analyses of proliferation, stem/differentiation markers (Lgr5, Lgr4, Axin2, Muc5ac), and signaling molecules are necessary. Histological data rely heavily on high-magnification images of limited gland numbers; low-magnification overviews and robust quantification should be provided.

5. Confounding Organoid Conditions and BMP Source

Organoid culture conditions are inconsistently described. Because the base medium contains Noggin (a BMP antagonist), it is difficult to isolate the effects of BMP modulation. Ideally, experiments should manipulate BMP ligands alone. Additionally, the source of BMPs in organoids is unclear—are epithelial cells themselves producing BMPs? BMP ligand expression in WT and Bmpr1a-KO organoids should be tested.

6. Interpretation of NF-κB Activation and p65 Staining

Figure 3B's p65 staining is ambiguous. The cytoplasmic localization of p65 in isthmal progenitors does not clearly reflect NF-κB activation. Co-staining with markers of proliferation/differentiation and quantification of p65+ cells (number, position, nuclear localization) are needed. Western blots in Figures 3D and 3F should include total protein levels, not just phospho-signals, and should be presented by condition and time point to enable interpretation.

7. Inadequate Justification and Testing of IL-1β Axis

IL-1β is introduced abruptly, with no rationale for its prioritization over other cytokines. The source of IL-1β (macrophages, neutrophils) is not directly demonstrated—co-staining or FACS-based validation is required. Foundational literature on IL-1β in the stomach (e.g., Tu et al., 2008; Serizawa et al., 2014) should be cited. IL-1α, another ligand of IL1R1, is not discussed—its expression and role should be considered.

8. IL1R Knockout Analysis Is Incomplete

The Il1r1 stromal KO model is under-analyzed. Its phenotype under uninfected conditions is not described. Since in situ hybridization shows epithelial Il1r1 expression, an epithelial-specific KO (e.g., Axin2-CreERT2) should be tested. Markers such as Muc5ac, p65, chemokines, and BMP components should be examined. The increased H. pylori CFU in KO mice also requires mechanistic interpretation.

9. Unclear BMP Dynamics During H. pylori Infection

The manuscript lacks time-course data on BMP ligand, receptor, and SMAD expression following H. pylori infection. Furthermore, it is unknown whether H. pylori infection has additional or redundant effects in Bmpr1a-deficient mice. These experiments are essential to distinguish direct infection-induced changes from BMP-dependent ones.

10. Ambiguous Interpretation of PGE2/COX2 Axis

The focus on COX2/PGE2 signaling in Figure 4 appears sudden and lacks explanation. Was this pathway selected from unbiased transcriptomic analysis? COX2 is involved in multiple prostanoid pathways (e.g., PGI2, TXA2, PGD2), none of which are examined. The COX2 IHC appears overexposed and does not demonstrate epithelial or immune expression. PGE2 receptors and downstream YAP regulators (e.g., EP4, LATS1/2) should be analyzed. The spatial disconnect between COX2 (pit) and aYAP (isthmus) is not addressed.

11. Stromal Heterogeneity Overlooked

Recent scRNA-seq studies (e.g., Manieri et al., 2023) emphasize the diversity of gastric stromal cells. The IL1R1+ population appears distinct from the Rspo3+ myofibroblasts previously described by the authors. Yet IL-1 induces Rspo3 in culture, implying potential heterogeneity. More refined cell classification and functional testing (e.g., single-cell, lineage tracing) are needed.

Minor Comments

• Specify whether LPS stimulation is TLR4-dependent. H. pylori-derived LPS may be more relevant than Klebsiella LPS.
• Clearly define organoid culture conditions, especially the presence or absence of Noggin.
• Provide total protein blots (not just phospho-signals) in Figures 3D and 3F.
• Improve image quality for COX2 (Figure 4B) and include low-magnification overviews of histological data.
• Explain how BMP2 suppresses chemokine induction (Figure 3C)—is this due to cytotoxicity at high concentrations?

Version 1:

Reviewer comments:

Reviewer #1

(Remarks to the Author)
The authors have addressed all points I raised in the previous review.

Reviewer #2

(Remarks to the Author)
I have reviewed the responses to the points we raised and find the explanations appropriate. However, the data presented in Supplementary Figure 1 do not appear to be consistent with the main text. For example, panels a and c in Supplementary Figure 1 seem to need to be swapped to align with the revised text. Please carefully recheck all figures and correct them to ensure consistency with the text.

Reviewer #3

(Remarks to the Author)
The authors have made a substantial effort to address the reviewers' previous comments and have added additional analyses and experiments in the revised manuscript. These additions improve several aspects of the study. However, several of the key conceptual issues raised in the previous review remain insufficiently resolved. Importantly, the concerns discussed below were already raised during the previous round of review, and although the authors have attempted to address them in the response letter, the underlying issues remain only partially addressed and in some cases unresolved.

Major Comment 1-1 – Whether Bmpr1a deletion truly mimics H. pylori infection
In my previous review, I raised concerns regarding the interpretation that loss of BMP signaling "mimics" the epithelial and inflammatory responses induced by Helicobacter pylori infection, and questioned whether H. pylori infection exerts additional or redundant effects in Bmpr1a-deficient mice. In the response letter, the authors cite their previous study (Nature Communications, 2022) as supporting this concept. While that work provides important context, a careful comparison between the two studies raises additional questions regarding the relationship between BMP suppression and infection-induced signaling.
In the 2022 study, the comparison between BMP-deficient and infected conditions primarily relied on measurements of gland height and epithelial proliferation (Ki67). By contrast, in the current manuscript, additional readouts—such as immune cell infiltration, YAP activation, and stromal COX2 expression—are evaluated across WT, WT+Hp, and BMP-KO conditions, without a systematic comparison including BMP-KO+Hp across these same parameters. As a result, the phenotypic relationships across conditions are not assessed in a fully comparable manner across studies. The available data suggest a pattern closer to:
WT < WT + Hp < BMP-KO ≈ BMP-KO + Hp, rather than
WT < WT + Hp ≈ BMP-KO ≈ BMP-KO + Hp.
This pattern implies that BMP deficiency induces a broader perturbation of gastric epithelial homeostasis than infection alone, rather than faithfully recapitulating the infection-induced state.
Despite this, the current manuscript repeatedly uses language implying equivalence between the two conditions. For example, the Results section states that Bmpr1a deletion "mimics" or "recapitulates" epithelial responses observed during H. pylori infection. These statements appear stronger than what the current data support.
It would therefore be important for the authors to more carefully distinguish between (partial) phenotypic overlap and true mechanistic equivalence between BMP suppression and infection-induced signaling, and to revise statements implying that Bmpr1a deletion directly mimics Helicobacter infection.

Major Comment 1-2 – Interpretation of BMP signaling in the organoid PAMP experiments
A related issue concerns the interpretation of the organoid experiments presented in Fig. 3. In this system, epithelial cells are cultured in Noggin-containing medium, which functionally suppresses BMP signaling and therefore approximates a BMP-low state. Under these conditions, stimulation with bacterial PAMPs robustly activates NF-κB signaling and chemokine expression, whereas addition of BMP2 suppresses these responses. However, the experiments presented here primarily demonstrate that BMP stimulation suppresses PAMP-induced NF-κB activation. They do not directly establish that loss of BMP signaling is sufficient to induce NF-κB activation or to prime epithelial cells for inflammatory responses in organoids. The interpretation that BMP loss actively drives or sensitizes NF-κB signaling in this context therefore appears stronger than what the current data directly support.
Furthermore, in their response to my previous suggestion to examine BMP signaling under Noggin-free conditions, the authors state that they intentionally used full medium containing BMP inhibitors to mimic the stem cell niche and again refer to their 2022 study. While this rationale is understandable, the current experimental design makes it difficult to disentangle

the specific contribution of BMP signaling from the combined effects of multiple niche factors present in the culture system. If BMP suppression were truly a major determinant of infection-induced inflammatory signaling, one might expect BMP-deficient tissue in vivo to exhibit enhanced inflammatory responses upon Helicobacter infection. However, the in vivo data presented in the authors' previous work do not clearly demonstrate such an interaction.
To clarify these issues, it would be appreciated to more directly evaluate the relationship between BMP signaling and infection-induced inflammatory responses in vivo. Direct comparison of key components of the proposed signaling cascade —including epithelial NF-κB activation, IL-1–related signaling, stromal COX2/PGE2 activation, and epithelial YAP activation —across WT, WT+Hp, BMP-KO, and BMP-KO+Hp conditions would help determine whether BMP suppression truly sensitizes epithelial tissue to microbial inflammatory signaling.

Major Comment 2 – Insufficient presentation, integration, and methodological description of the single-cell RNA-seq analyses
In my previous review, I suggested that single-cell or spatially resolved analyses would be required to clarify the proposed epithelial–immune–stromal signaling cascade. In the response letter, the authors state that several scRNA-seq analyses have been incorporated in the revised manuscript. While these additions are appreciated, the current presentation remains difficult to interpret and does not fully address the concerns raised previously.
2-1 Dataset provenance and sample composition remain unclear
First, the provenance and composition of the "newly generated" murine scRNA-seq datasets are not clearly described. The Methods section describes epithelial cell isolation and sequencing of EPCAM-positive cells, but it is not clearly specified which genotypes or infection conditions are represented in these datasets. Based on the figure labeling, the epithelial datasets appear to include conditions such as WT, BMP-KO, and Hp-infected samples (with the uninfected control possibly corresponding to WT), but this is not explicitly stated. Given that two biological replicates were analyzed per condition, this would correspond to approximately six to eight individual samples, yet the manuscript does not clarify whether these samples were processed and sequenced separately, multiplexed within the same run, or processed across different sequencing batches. Because no description of pooling, hashtag-based multiplexing, or sample indexing strategies is provided, it is difficult to determine how the individual samples were handled experimentally and how batch structure may relate to the downstream analyses.

2-2 Cross-dataset inference and lack of a unified analytical framework
Second, the manuscript draws on several heterogeneous datasets, including newly generated murine epithelial datasets, previously published murine stromal datasets, and human scRNA-seq atlas data. These datasets differ substantially in species, tissue context, and infection status. While each dataset may be appropriate for addressing specific questions, the current presentation gives the impression that different datasets are selectively used to support individual components of the proposed signaling cascade, rather than providing a unified and internally consistent analysis across conditions.
It would therefore be important to clarify how each dataset contributes to the overall model, and to more explicitly define which conclusions are supported by direct comparisons within the same dataset versus those inferred across different datasets. As a result, it remains unclear whether the proposed signaling cascade is supported within a unified experimental framework or inferred from observations distributed across independent datasets.

2-3 Lack of transparency in data presentation and validation
Third, and perhaps most importantly, key information required to evaluate the datasets is not provided. The manuscript does not show UMAP visualizations labeled by sample or condition, does not report cell numbers per cluster or per sample, and does not provide clear validation of cluster annotations using canonical markers. In addition, although the analysis appears to involve lineage-specific subclustering (e.g., epithelial and stromal subsets), the manuscript does not present a clear overview of the global dataset before these subset analyses. Providing an initial global view of the integrated dataset, followed by the corresponding subclustering analyses, would make the analytical workflow substantially easier to interpret and evaluate. These details are critical for assessing the robustness and reproducibility of the single-cell analyses.

2-4 Limitations of data representation using averaged expression
Finally, the analyses rely largely on heatmaps of averaged expression values. Such representations do not allow assessment of the fraction of expressing cells, the magnitude of expression differences, or statistical significance of differential expression.
Overall, a more transparent, reproducible, and systematic presentation of the scRNA-seq analyses—including clear dataset descriptions and direct comparisons between infection and BMP-deficient conditions—would substantially strengthen the study. The authors should perform direct single-cell–level comparisons of transcriptional programs between these conditions and present a systematic comparison of transcriptional similarities or differences between BMP signaling loss and infection-induced responses, rather than relying on favorable snapshots from bulk/single-cell RNA-seq analyses.

Major Comment 3 – Spatial relationship between stromal COX2 expression and epithelial YAP activation remains unresolved
In my previous review, I noted that the proposed stromal–epithelial signaling axis does not clearly account for the spatial organization of the gastric gland. In the response letter, the authors state that COX2-positive stromal cells are distributed throughout the gland axis. However, the immunostaining data shown in the manuscript appear to indicate that COX2-positive stromal cells are enriched in the pit region rather than being evenly distributed along the gland. At the same time, epithelial YAP activation is described as occurring primarily in the isthmus region. The spatial relationship between these two signals therefore remains unclear.
The additional scRNA-seq analyses also do not resolve this issue. The stromal datasets contain heterogeneous stromal populations, but the manuscript does not clearly identify which stromal subsets represent the functional source of COX2/PGE2 signaling. In addition, the samples in the stromal dataset are derived from uninfected WT corpus and antrum

tissues, which may not fully represent the conditions or anatomical regions relevant to the authors' proposed model. Similarly, although epithelial expression of the PGE2 receptor is shown, the manuscript does not clearly define which epithelial populations are capable of responding to this signaling, nor does it indicate whether receptor expression is altered under BMP-deficient or Helicobacter-infected conditions.
Consequently, the spatial organization of the proposed IL-1–COX2–PGE2–YAP signaling axis remains insufficiently supported. Additional spatial analyses linking stromal COX2 expression with epithelial YAP activation along the gland axis, including quantitative analysis of cell distribution and co-staining experiments, would help clarify this relationship.
Importantly, the current study does not include functional perturbation experiments testing whether COX2/PGE2 signaling is required for epithelial YAP activation in vivo. Experiments examining the effects of COX2/PGE2 inhibition (e.g., Cox2 inhibitors in vivo or alternatively in assembloids) would therefore provide important support for the proposed stromal–epithelial signaling axis.

Major Comment 4 – The mechanistic link between BMP signaling and NF-κB/YAP responses remains insufficiently established
In response to the initial review, the authors have added several experiments examining signaling responses in organoids, including Western blot analyses of NF-κB pathway activation, demonstration of BMP-induced SMAD phosphorylation, and short-term BMP2 stimulation experiments. These additions are helpful and clarify certain aspects of BMP signaling within the organoid system.
However, the central mechanistic question raised in the initial review—namely how BMP suppression leads to NF-κB and infammatory signaling and YAP activation in the proposed pathway—remains insufficiently resolved.
First, although the authors demonstrate NF-κB activation and modulation of YAP activity in organoids, the revised experiments do not establish the functional hierarchy linking BMP signaling, NF-κB activation, chemokine expression, and YAP activity. The experiments presented show correlations between these pathways but do not demonstrate whether NF-κB or YAP activity is required for the observed transcriptional responses.
Second, regarding pathway inhibition, the authors indicate that they attempted pharmacological inhibition experiments but were unable to obtain interpretable results. However, the original comment specifically suggested testing NF-κB and YAP pathway inhibition, whereas the response primarily discusses attempts to inhibit BMP signaling and YAP signaling. As a result, the functional requirement of NF-κB signaling, which is central to the proposed inflammatory pathway, remains experimentally untested.
Finally, the authors state that BMP directly suppresses epithelial YAP activity in organoids (Fig. 3g–h). This observation suggests that epithelial YAP activation could occur directly downstream of BMP signaling loss. However, the central model of the manuscript proposes that epithelial YAP activation is driven by stromal COX2–PGE2 signaling downstream of IL-1β. Therefore, it is not evident whether stromal PGE2 signaling is required for epithelial YAP activation or whether BMP loss alone is sufficient to induce this response, which needs to be clarified.

Major Comment 5 – Interpretation of epithelial IL-1 signaling remains incomplete
In the original review, I noted that Il1r1 expression appears to be present not only in stromal cells but also in epithelial cells, and therefore suggested that the potential contribution of epithelial IL-1 signaling should be considered.
In the revised manuscript, the authors provide further characterization of stromal Il1r1 knockout mice. To argue against a role for epithelial IL-1 signaling, they claim that IL-1β treatment of epithelial organoids does not activate YAP signaling.
However, the conclusion that IL-1β does not activate YAP signaling in epithelial cells is based on limited analysis. The assessment of YAP activity appears to rely mainly on p-YAP at a single short time point and one IL-1β concentration. Given that the in vivo part of the study uses nuclear active YAP (aYAP) as the relevant readout, a more direct analysis of epithelial YAP activation would be needed, for example by assessing nuclear aYAP localization across multiple time points and IL-1β concentrations.
Indeed, their western blotting data indicate that IL-1 can directly activate NF-κB pathway in organoids (Fig.3g), thus there remains a possibility that IL-1 signaling may also act directly on epithelial cells. If so, the directionality of the proposed signaling cascade (BMP suppression → epithelial NF-κB/chemokine → immune IL-1β → stromal COX2/PGE2 → epithelial YAP) may be more complex than currently depicted, and epithelial IL-1 signaling could represent an additional or parallel regulatory layer.
A more careful analysis of epithelial IL-1 responses in organoids, including YAP activation as well as effects on NF-κB/chemokine pathway, would provide a more robust assessment of whether epithelial IL-1 signaling plays a role here. While additional in vivo models may not be essential for the current study, the current conclusion that epithelial IL-1 signaling is irrelevant appears premature based on the limited analysis presented.

Minor Point 1 – Overstatement and unsupported causal language in the Abstract and main text
Throughout the manuscript, and particularly in the Abstract, several conclusions are stated in a manner that appears stronger than what the current data directly support. In multiple instances, associative or partially supported observations are presented as mechanistically established causal relationships. For example, statements such as "gland hyperplasia is fueled by fetal-like reprogramming," "epithelial cells that lack BMP signaling are primed to respond to microbes," "these in turn release IL-1β, promoting stromal remodeling," and "this epithelial-immune-stromal cascade gives rise to H. pylori-driven gastric pathology" appear to go beyond what is directly demonstrated by the current data. In several of these cases, the manuscript provides evidence partially consistent with the proposed model, but not definitive functional proof of the stated causal hierarchy. I would therefore encourage the authors to carefully revise the Abstract and corresponding sections of the main text to distinguish more clearly between direct experimental findings and model-based interpretation.
Minor Point 2 – Definition of "fetal-like reprogramming" and its relationship to BMP–YAP signaling
The concept of "fetal-like reprogramming" represents a central theme of the manuscript, yet its definition and usage throughout the study remain somewhat unclear. Throughout the manuscript, the authors refers interchangeably to a "fetal-like reprogramming" or a "fetal-like state," even in the Title and Abstract. However, based on the data presented, these terms

appear to be inferred primarily from changes in the expression of selected fetal-associated genes. It therefore remains unclear whether the study demonstrates a bona fide fetal-like cellular state or rather a fetal-like transcriptional signature. Given the strength of the terminology used, it would be helpful for the authors to clarify how “fetal-like reprogramming” or “fetal-like state” is operationally defined in this study.
In addition, the relationship between fetal-like reprogramming and epithelial YAP activation remains somewhat ambiguous. As shown in the organoid experiments (Fig. 3g–h), BMP stimulation directly suppresses epithelial YAP activity, suggesting that loss of BMP signaling could intrinsically promote YAP activation in epithelial cells. However, the Abstract states that loss of epithelial BMP signaling is sufficient to induce the fetal-like state in vivo but not in organoids, implying that BMP loss alone is not sufficient to induce this program in epithelial cells in isolation. It would therefore be helpful if the authors could clarify how the fetal-like transcriptional changes relate mechanistically to epithelial YAP activation and whether these processes consistently occur in the same epithelial populations across the datasets presented.
Minor Point 3 – Clarification of the proposed signaling model
The signaling framework proposed in this manuscript involves multiple interacting cellular compartments and signaling pathways, including epithelial BMP signaling, inflammatory responses, immune-derived IL-1β, stromal COX2/PGE2 signaling, and epithelial YAP activation. For clarity, it would be helpful if the authors could include a schematic diagram summarizing the proposed signaling model and the directionality of the interactions between epithelial, immune, and stromal compartments.

Finally, because several major conceptual and mechanistic issues remain to be addressed, I have focused the comments above on the principal points that are most critical for interpreting the study. Additional minor issues and questions regarding specific data presentation are present but are not listed here at this stage.

Version 2:

Reviewer comments:

Reviewer #3

(Remarks to the Author)
The revised manuscript is substantially improved compared with the previous version. I appreciate the authors’ careful and constructive efforts to address my prior concerns. In particular, the authors have added new experiments and analyses, clarified several aspects of the proposed epithelial–immune–stromal pathway, and appropriately toned down a number of statements that previously implied direct equivalence between Bmpr1a deletion and H. pylori infection. Overall, many of my previous mechanistic concerns have now been adequately addressed or appropriately revised.
However, I still have concerns regarding the analysis and presentation of the newly generated epithelial scRNA-seq dataset. This dataset has the potential to substantially strengthen the manuscript, but the current presentation does not yet fully clarify how the single-cell data support the histological phenotypes and the proposed model.

1. The authors now clarify that epithelial cells from eight mice were processed together in a single multiplexed 10x GEM-X Flex run. However, the Methods also state that sample integration was performed using Harmony to correct for sample effects. Given that the samples were processed in a single multiplexed run, the rationale for Harmony-based integration should be more clearly explained, particularly because integration may reduce condition-dependent biological differences. This point is relevant because Bmpr1a KO tissue shows marked gland hyperplasia histologically, whereas the integrated UMAP does not clearly show a distinct KO-associated epithelial state. I do not necessarily think that extensive additional computational analyses are required here, but the authors should explain whether the main conclusions, particularly the condition-dependent changes in epithelial cell populations, are preserved after integration.

2. The revised manuscript includes epithelial cluster annotations and marker dot plots, which improve the presentation. However, the basis for the relatively fine-grained epithelial subcluster annotation remains insufficiently explained. The authors define 18 epithelial subsets, including multiple stem, TA, and progenitor cells. At the same time, many corpus-associated cell types are also present in the dataset. Because most downstream analyses depend on clustering and annotation, the authors should more carefully explain and validate how these epithelial populations were defined.
Specifically, the authors should provide:
1)A table listing each epithelial cluster, the key marker genes used for annotation, the assigned cell identity, the expected anatomical location along the gastric gland axis, and the corresponding published gastric epithelial cell type when available.
2)A clear explanation of how antral and corpus epithelial cells were distinguished during tissue collection and during computational annotation.
3)Reanalysis focused on the relevant antral epithelial compartment after excluding corpus-associated cells and rare specialized epithelial populations such as chief, parietal, tuft, and endocrine cells.
4)The authors should quantitatively assess the proportions of the relevant epithelial populations at the biological sample level after refined annotation. In Supplementary Fig. 2c, the authors show the proportions of each epithelial subpopulation in individual samples. However, it remains unclear whether the populations that the authors consider to represent or drive the hyperplastic response are reproducibly increased in Bmpr1a KO and/or H. pylori-infected samples. The authors should present the proportions of the relevant epithelial populations in each group and indicate which population(s) correspond to the expanded hyperplastic/proliferative compartment observed histologically.
3. The current biological interpretation of the epithelial scRNA-seq dataset relies largely on predefined UCell gene signature scores and selected marker heatmaps. However, they do not fully show, from an unbiased perspective, what epithelial transcriptional programs are most altered by H. pylori infection or Bmpr1a deletion, or which changes are shared versus distinct between these two perturbations. Since the Methods state that pseudobulk differential expression analysis was

performed by aggregating counts by sample and cluster and analyzing them with DESeq2, the corresponding pseudobulk DEG results should be clearly presented in the main text or supplementary data. Specifically, the authors should provide Cluster-level or major-lineage-level DEG analyses for WT+Hp versus WT and Bmpr1a KO versus Bmpr1a WT, and complete DEG lists as supplementary tables, including gene names, log fold changes, p values, adjusted p values, and the cluster or lineage in which each comparison was performed.

4. In Fig. 1, the authors show that H. pylori infection and Bmpr1a deletion are associated with reduced Lgr5 expression, increased Anxa1 expression, reduced Lgr5 ISC signature, increased regenerative stem cell signature, and increased YAP activity. In Fig. 2, the organoid data show that Bmpr1a deletion reduces Id1 expression. In Fig. 4, PGE2 treatment induces YAP target genes such as Ctgf, Ankrd1, and Cyr61. However, the current scRNA-seq presentation mainly shows selected signatures and marker heatmaps, and does not clearly demonstrate whether these same genes or pathways display concordant changes across the relevant epithelial populations and experimental conditions.

Thus, they should present condition-specific feature plots and/or violin plots for Lgr5, Anxa1, Id1, Ctgf, Ankrd1, and Cyr61 across the relevant antral epithelial populations. In addition, the authors should show whether the Lgr5 ISC signature and YAP target gene signature are consistently altered in the scRNA-seq data in a manner concordant with Fig. 1. These analyses should be presented at the level of major epithelial populations or refined antral epithelial clusters, rather than only as broad global UMAP projections. At present, the data do not clearly define an “epithelial reprogramming” state.

Minor Comments

Related to the point 4, I remain concerned about the use of the term “reprogramming.” The following statements should therefore be revised for consistency.

1. Title

Current wording: “Helicobacter pylori triggers gastric mucosal reprogramming into the fetal-like transcriptional state via stromal IL1-β signaling”

Suggested revision: “Helicobacter pylori triggers gastric mucosal remodeling toward a fetal-like transcriptional program via stromal IL-1β signaling”

2. Page 3, Abstract

Current wording:

“Loss of epithelial BMP signaling is sufficient to induce epithelial reprogramming in vivo via epithelial-stromal-immune crosstalk.”

Suggested revision:

“Loss of epithelial BMP signaling is sufficient to induce epithelial transcriptional changes in vivo via epithelial-stromal-immune crosstalk.”

3. Page 3, Abstract

Current wording:

“Our data provide mechanistic insights into fetal-like regenerative reprogramming…”

Suggested revision:

“Our data provide mechanistic insights into fetal-like transcriptional responses…”

4. Page 4, Introduction

Current wording:

“ultimately leading to fetal-like transcriptional reprogramming of the adjacent epithelium.”

Suggested revision:

“ultimately contributing to a fetal-like transcriptional response in the adjacent epithelium.”

5. Page 4, Introduction

Current wording:

“depletion of the IL-1 receptor in stromal cells prevents epithelial reprogramming and gland hyperplasia…”

Suggested revision:

“depletion of the IL-1 receptor in stromal cells prevents epithelial transcriptional changes and gland hyperplasia…”

6. Page 9, Results heading

Current wording:

“Stromal-derived PGE2 triggers epithelial reprogramming into the fetal-like transcriptional state”

Suggested revision:

“Stromal-derived PGE2 promotes epithelial YAP activation and a fetal-like transcriptional response”

7. Page 11, Results heading

Current wording:

“Epithelial reprogramming depends on IL-1β-driven remodeling of gastric stromal cells”

Suggested revision:

“Epithelial YAP activation and fetal-like transcriptional changes depend on IL-1β-driven remodeling of gastric stromal cells”

8. Page 13, Discussion

Current wording:

“COX2-PGE2 signaling in mesenchymal stromal cells induces YAP activation in the neighbouring epithelium and cellular reprogramming into the fetal-like transcriptional state.”

Suggested revision:

“COX2-PGE2 signaling in mesenchymal stromal cells promotes YAP activation in the neighbouring epithelium and contributes to fetal-like transcriptional changes.”

Open Access This Peer Review File is licensed under a Creative Commons Attribution 4.0 International License, which permits use, sharing, adaptation, distribution and reproduction in any medium or format, as long as you give appropriate credit to the original author(s) and the source, provide a link to the Creative Commons license, and indicate if changes were made.
In cases where reviewers are anonymous, credit should be given to 'Anonymous Referee' and the source.
The images or other third party material in this Peer Review File are included in the article's Creative Commons license, unless indicated otherwise in a credit line to the material. If material is not included in the article's Creative Commons license and your intended use is not permitted by statutory regulation or exceeds the permitted use, you will need to obtain permission directly from the copyright holder.
To view a copy of this license, visit https://creativecommons.org/licenses/by/4.0/

Point-by-point response

We would like to thank all of the reviewers for their time in assessing our manuscript and for their constructive suggestions. Their insights have helped us to significantly improve the manuscript and strengthen our findings. Below, we address each of their points in turn.

Reviewer #1 (Remarks to the Author):

Sigal lab has demonstrated that Helicobacter pylori infection induces a fetal-like state in the gastric epithelium through mesenchymal PGE_2 production. The authors observed hyperproliferation, YAP activation, and fetal-like gene expression patterns in the gastric epithelium upon H. pylori infection in a Bmpr1a-dependent manner. They further showed that H. pylori-induced NF-κB signaling acts synergistically with BMP inhibition. Using single-cell RNA sequencing, organoid culture, and genetically engineered mouse models, the study suggests that YAP activation is driven by IL-1β–mediated PGE_2 production from intestinal stromal cells. While it is known that immune responses to infection can induce fetal-like states, the underlying mechanisms have remained unclear. This study provides valuable insights into how infection and inflammation drive fetal-like transcriptional and regenerative programs via YAP activation in gut epithelium. I find this manuscript highly interesting and informative. However, several points need to be addressed prior to publication.

Major Comments
1- Figure 2: niche factor comparison is insufficient.
The comparison between WRN and no WRN conditions is overly simplistic. To better assess the impact of BMP signaling, the authors should compare WRN vs. WR (i.e., excluding Noggin). This distinction is critical, as R-spondin-conditioned medium may contain unknown factors that could confound interpretation. In the Methods section, the authors mention "sWnt (U-Protein Express)"—presumably referring to surrogate Wnt—but this must be clearly defined and described to ensure experimental reproducibility.

> We thank the reviewer for their suggestion. The idea behind comparing WRN vs no WRN medium was to mimic exposure of cells to the stem cell niche in the gland vs the differentiated cell niche in the pit. We followed the suggestion of the reviewer and removed noggin alone to explore the effects of endogenous BMP and found that within the same passage this is not sufficient to shift the lineage to pit cells (or to cause any effect on YAP targets gene expression). We have included this new data in **Supplementary Figure 2a-d**.

SupplFigure2

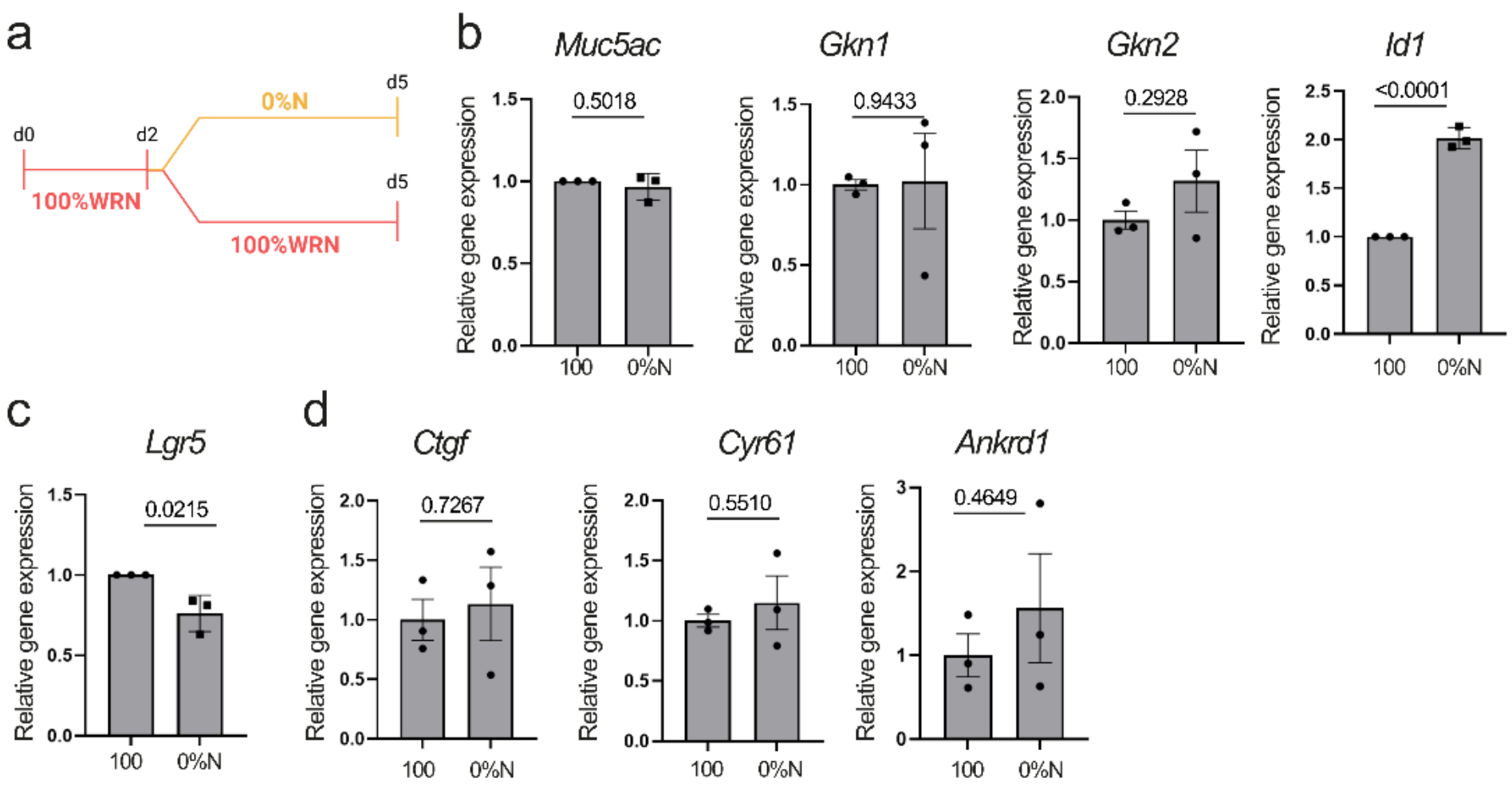

We also modified the description of surrogate Wnt in the Methods section ("Organoid culture paragraph, Lines 640-641).

2- Line 282: PGE_2 and Wnt signaling.
The sentence "PGE_2 can activate YAP signaling in gastric epithelial cells that have reduced Wnt signaling activity" is misleading. It remains unclear whether PGE_2 reduces Wnt activity through YAP or through other GPCR-mediated pathways. To support this claim, the authors should block YAP signaling using TEAD inhibitors or genetic inactivation strategies.

We believe that there is a misunderstanding: we do not want to claim that PGE2 reduces Wnt, but rather that it acts stronger on cells with lower Wnt activity. To clarify this, we have adjusted the text (lines 330-331).

3- BMP2 inhibition of NF-κB activation lacks mechanistic support.
The finding that BMP2 suppresses PAMP-induced NF-κB activation is intriguing, yet the underlying mechanism is not explored. While detailed mechanistic dissection may not be necessary, some additional experiments would strengthen this point. For example, analysis of upstream regulators such as TRAF6 or A20, or modulation of NF-κB activation by cytokines like IL-17 or TNF-α (instead of PAMPs), would be informative.

We thank the reviewer for this important suggestion. We have now performed experiments that demonstrate that responsiveness to TNF-α is also reduced upon treatment with BMP2, thus indicating that BMP2 inhibits inflammation in general and is not restricted to inflammatory pathways triggered by PAMPS (**Figure 3i, Supplementary Figure 3i**).

Figure3

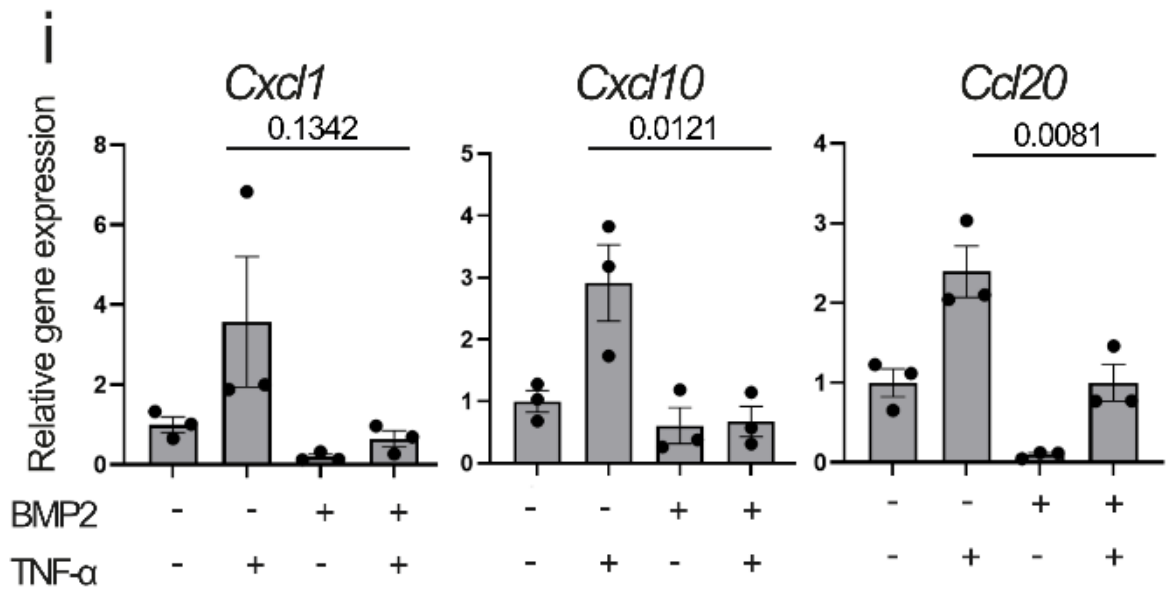

SupplFigure3

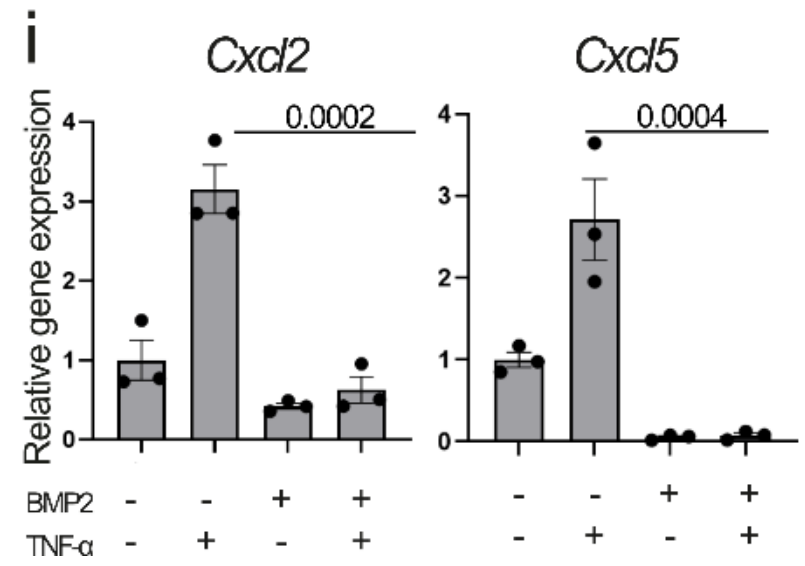

Furthermore, using PAMPS as well as TNF-α as triggers, we have now comprehensively explored various downstream mediators of the NF-κB pathway, such as A20, Traf6, Myd88 and Tifa, and found that they are also downregulated upon exposure to BMP2, indicating that various mediators are affected by BMP signaling (**Supplementary Figure 4a-d**).

SupplFigure4

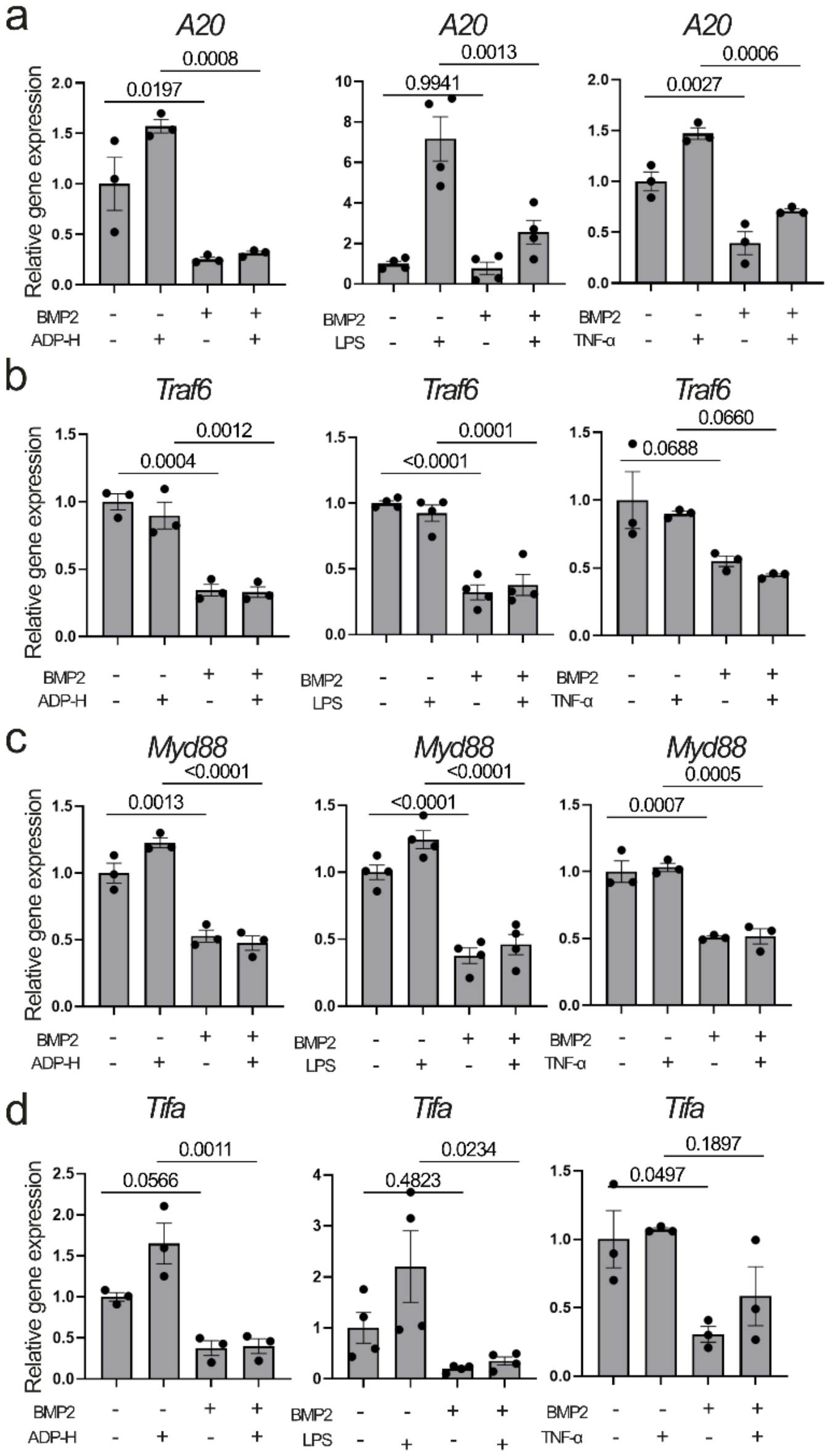

4- Line 287: Typo and immunostaining controls.
The sentence "IL-1β is upregulated in in the inter-crypt regions..." contains a typo ("in in"). In addition, the immunolabeling images would benefit from co-staining with lineage-specific markers to verify cellular identity.

We have now corrected the typo. Furthermore, we have performed immunolabelling with lineage-specific markers and found that the vast majority of cells expressing Il1-β are either macrophages or neutrophils, both in *Bmpr1a* KO and *H. pylori* infected mice. The data are now included in **Figure 5 h-i** and **Supplementary Figure 7b-c**, respectively.

Figure5

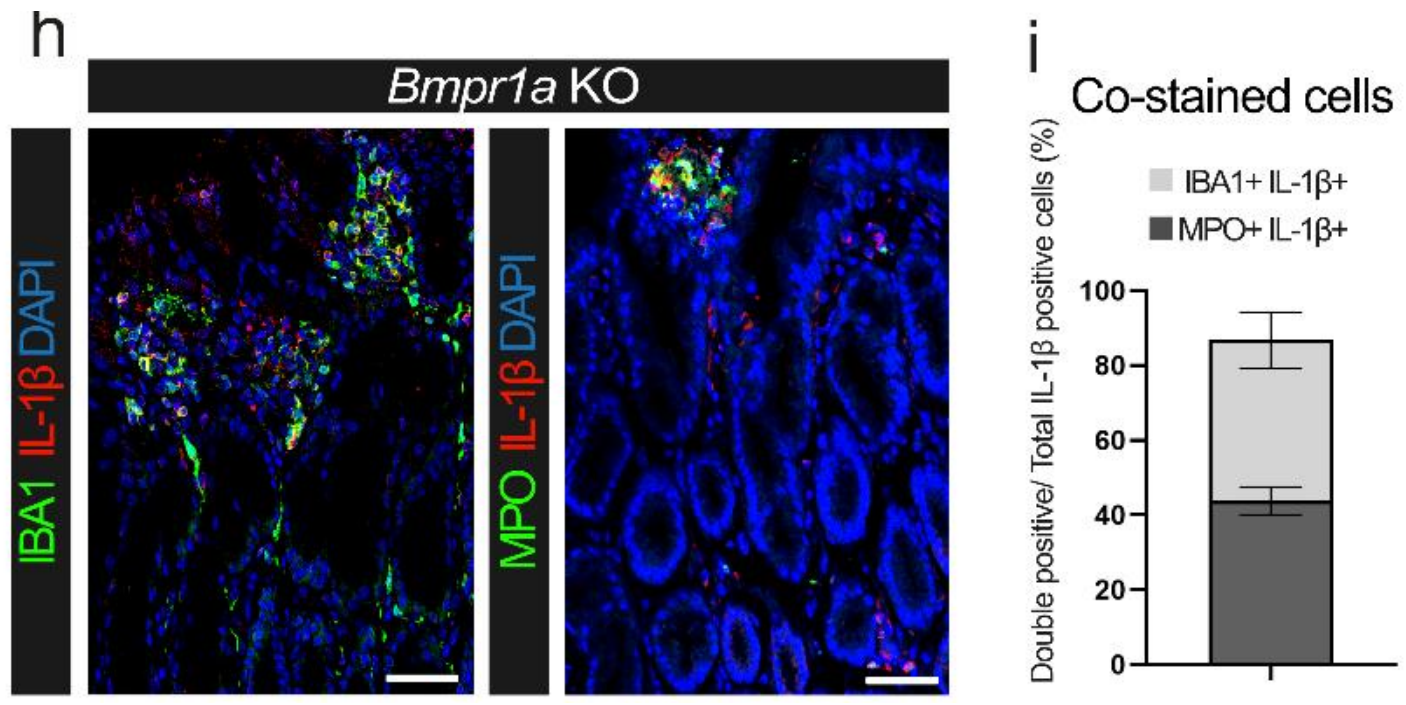

SupplFigure7

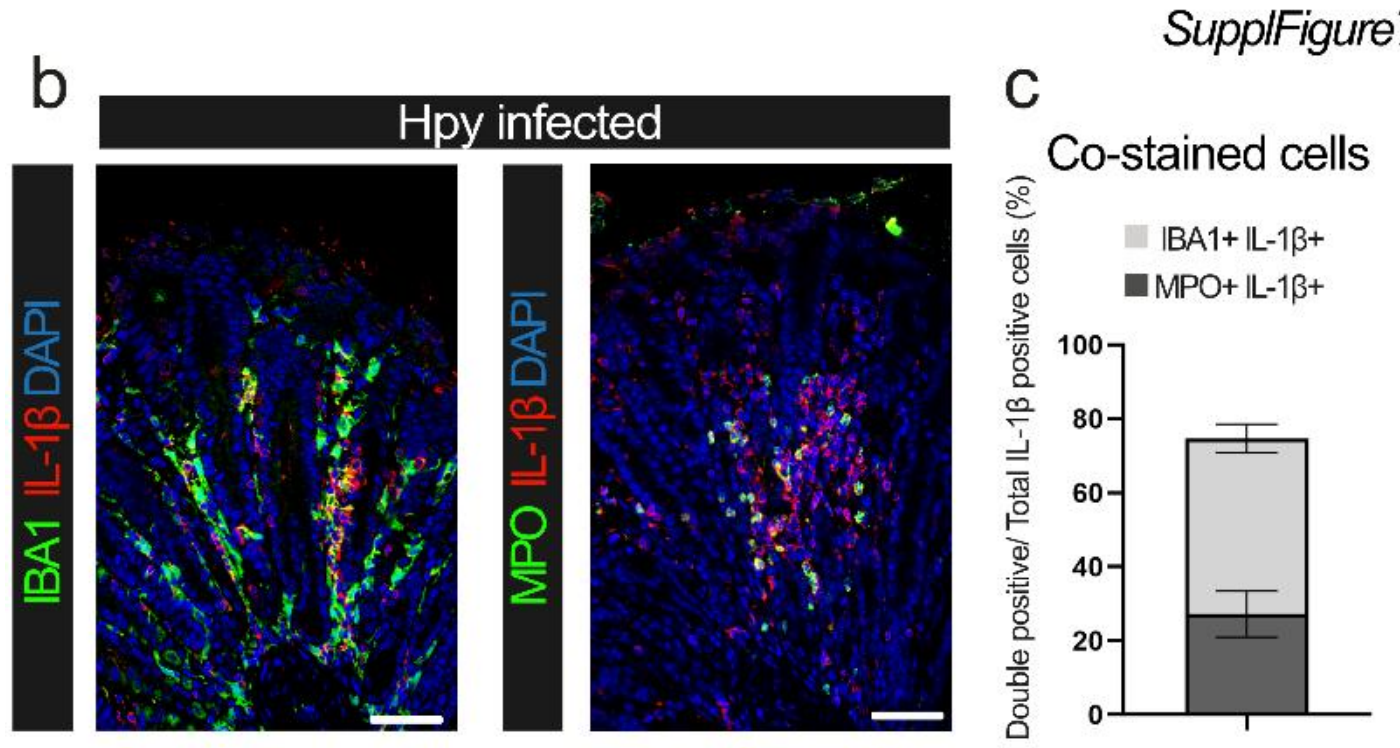

5- Line 297: IL-1β–producing cell types.
The authors state that IL-1β–expressing cells are primarily monocytes/macrophages, neutrophils, and to a lesser extent, dendritic cells. However, this analysis is based on scRNA-seq data from normal human stomach. Since IL1b and IL1r1 expression levels are likely modulated by infection or inflammation, the authors should validate these findings using in situ hybridization (ISH) or immunohistochemistry (IHC) on gastric mucosa with and without H. pylori infection.

To explore the cellular source of IL-1β, we have performed lineage-specific marker stainings and found that in infected as well as uninfected gastric tissue, the vast majority of cells expressing Il1beta are either macrophages or neutrophils (see figure above).

Regarding the receptor: based on available data sets (single-cell RNAseq from uninfected and infected human tissue, Il1r1 expression in murine mesenchymal stroma in infected and uninfected samples), the receptor levels appear to be unaffected by infection. Thus, we believe that it is mainly the high influx of IL1-β expressing cells and much less the modulation of receptor expression that induces IL1 signaling in the stroma. These data are now included in **Supplementary Figure 7d-e**.

SupplFigure7

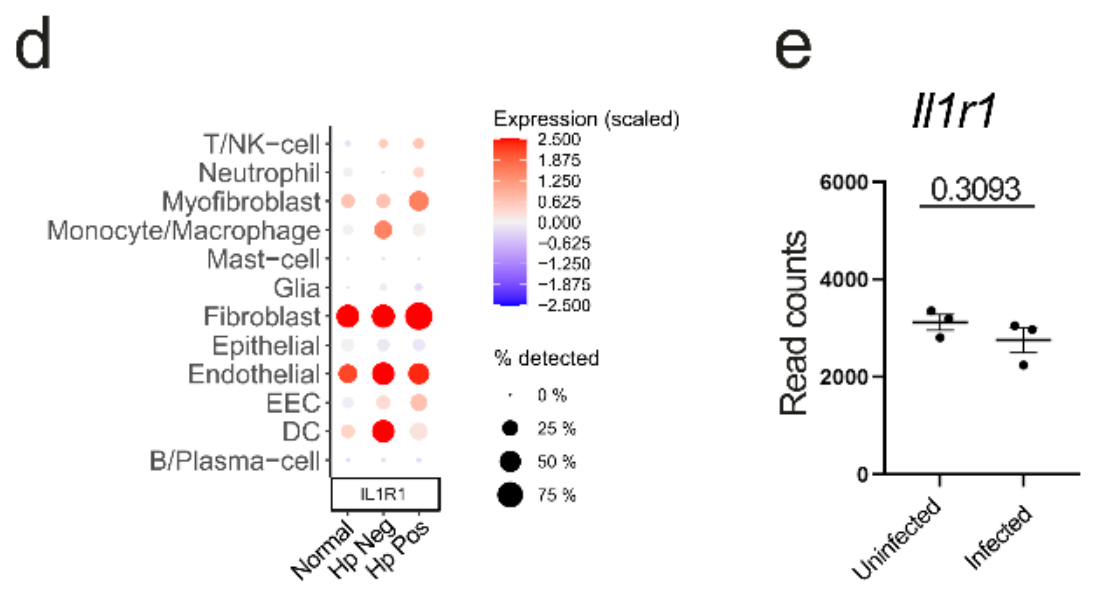

-

Minor Points
GSEA abbreviation
Please spell out "GSEA" at first mention. Also, avoid redundancy—"GSEA analysis" should simply be "GSEA."

We have now changed the text accordingly.

Inconsistent strain names
Line 314 should read: Col1a2-CreERT2 Il1r1^fl/fl^, hereafter referred to as Il1r1^KO^ mice.
Line 324: "KO mice" should be replaced with "Il1r1^KO^ mice" for clarity and consistency.

We now changed the text accordingly.

Reviewer #2 (Remarks to the Author):

I think that your current study presents meaningful insights as a continuation of your previous work on H. pylori-induced inflammation and hyperplasia. Notably, you demonstrated that Bmpr1a KO mice exhibit an expansion of KI67⁺ proliferative cells—mimicking gland hyperplasia observed in the antrum of PMSS1-infected WT mice—even in the absence of infection, which is quite compelling. This also appears to be the first reported instance of a fetal-like regenerative response in the stomach, as indicated by the expansion of the Anxa1⁺aYAP⁺ compartment following injury.
Additionally, your data clearly show that tissue reprogramming driven by H. pylori infection is regulated via IL-1 signaling in stromal cells, as supported by the use of Il1r KO mice. I agree with your interpretation that crosstalk among epithelial, stromal, and immune cells contributes to the fetal-like regenerative state, and I believe the conditional KO mouse systems you employed are appropriate to investigate this phenomenon.
However, I do have concerns that some key observations remain unconfirmed, and I wonder if additional validation might be possible using the models you previously established in your earlier studies.

1. From what I have seen, this point also seems to be missing in your previous studies related to H. pylori infection. When presenting histopathological images of H. pylori-infected mice, it appears that you have not shown H. pylori colonization on the epithelium. While it's generally assumed that H. pylori are present in the infected group, I believe it is important to provide visual confirmation of the infection status by staining for H. pylori to clearly distinguish between infected and uninfected conditions. This becomes particularly important if you aim to demonstrate that the uninfected Bmpr1a KO mice resemble the pathological features of WT infected mice.

We would like to thank the reviewer for their comment. To validate a successful infection, we always perform a culture-based analysis: a piece of stomach tissue is homogenized and the homogenate is plated on *H. pylori*-selective plates. This method is highly reliable and reproducible: uninfected mice never show *H. pylori* growth, mice that we infect always show colony forming units of *H. pylori.* We have learned in the past that this tool is also useful for quantifying the infection density. Where required, we have used fluorescent microscopy in previous studies to demonstrate the presence and localization of individual bacteria (Sigal M, et al. R-spondin-3 induces secretory, antimicrobial Lgr5⁺ cells in the stomach. Nat Cell Biol. 2019 Jul;21(7):812-823. doi: 10.1038/s41556-019-0339-9). However, these experiments have also revealed that imaging can become less reliable in conditions with low density of *H. pylori*, which is

particularly true upon a longer-term infection. Thus, CFU analysis is the most reliable way to confirm or exclude active infection. We now provide CFU data for infected WT mice in the paper (**Supplementary Figure 1c**), while the CFU counting protocol can be found in the Methods section ("*H. pylori* infection and CFU analysis"). No CFUs are found in controls that were not infected with PMSS1.

SupplFigure1

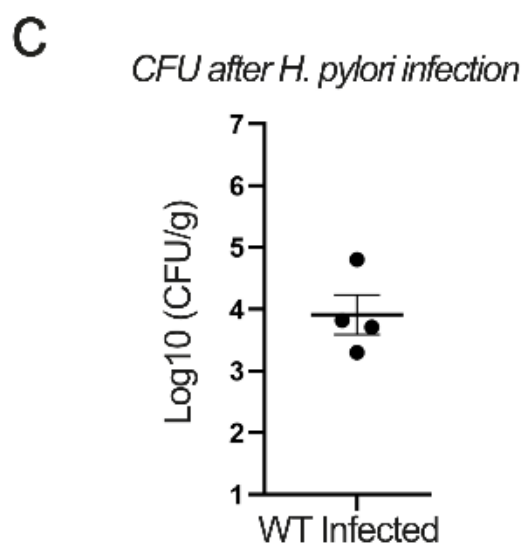

2. The gland length in Bmpr1a KO mice appears to be approximately 1.5 to 2 times greater than that in WT infected mice, and I would appreciate further explanation regarding this observation (Fig. 1a, g, i, Fig. 4b, g). This suggests that hyperplasia may have been induced in the absence of infection, and I believe it is important to consider whether there are potential pitfalls or limitations in the mechanistic interpretation of this finding.

We agree that the phenotype in *Bmpr1a* KO mice is stronger than in mice infected with *H. pylori*. Our interpretation is that although there is a reduction of BMP signaling upon infection, this is even more pronounced in the knockout. We now discuss this in the manuscript (see lines 123-125).

3. According to clinical reports, more than half of individuals infected with H. pylori remain asymptomatic. Given that, is the two-month period following oral infection with 10^8 CFU of H. pylori sufficient to induce chronic infection symptoms in your mouse model?
Alternatively, do you selectively analyze only those mice that exhibit symptoms among the infected group? Some clarification on this aspect of your experimental design would be helpful.

This is an interesting point. Indeed, while infection in humans only causes cancer in a small subset of individuals, all infected individuals develop chronic gastritis, which for most patients remains asymptomatic. This aspect is indeed reflected in the model. After 2 months of infection, we observe chronic gastritis in our mice, as well as development of early pre-malignant lesions, such as gland hyperplasia, similarly to what is seen in patients. Therefore, while of course not all aspects of the human disease are mimicked faithfully, the model does reflect key features of human infection. We now clarify that infection for 2 months with the PMSS1 strain of *H. pylori* in mice resembles aspects of chronic gastritis and early premalignant tissue responses in humans (lines 120-122).

4. I strongly agree with your observation in Figure 2 that fetal-like regeneration and YAP signaling regulation following the loss of BMP signaling during in vivo H. pylori infection cannot be recapitulated in conventional epithelial-only organoid systems, nor can they be fully explained by epithelial cell-intrinsic mechanisms. I was particularly impressed by your group's previous work on gastrointestinal assembloids that incorporate mesenchymal niches (M Lin, et al., Nat Commun 14.1 (2023): 3025., https://doi.org/10.1038/s41467-023-38780-3), and I would like to suggest considering

whether this advanced culture platform could be applied to the current study. If feasible, I believe it could greatly enhance the physiological relevance of the experimental validation.

We thank the reviewer for this comment. We have now carried out experiments with gastric assembloids to strengthen our findings. Specifically, we treated assembloids with IL-1β and observed that the number of COX2+ Vimentin+ stromal cells was significantly increased upon treatment (**Figure 4 k-l**). Moreover, IL-1β treatment increased the number of epithelial cells with nuclear active YAP (aYAP) (**Figure 6 j-k)**.

Figure4

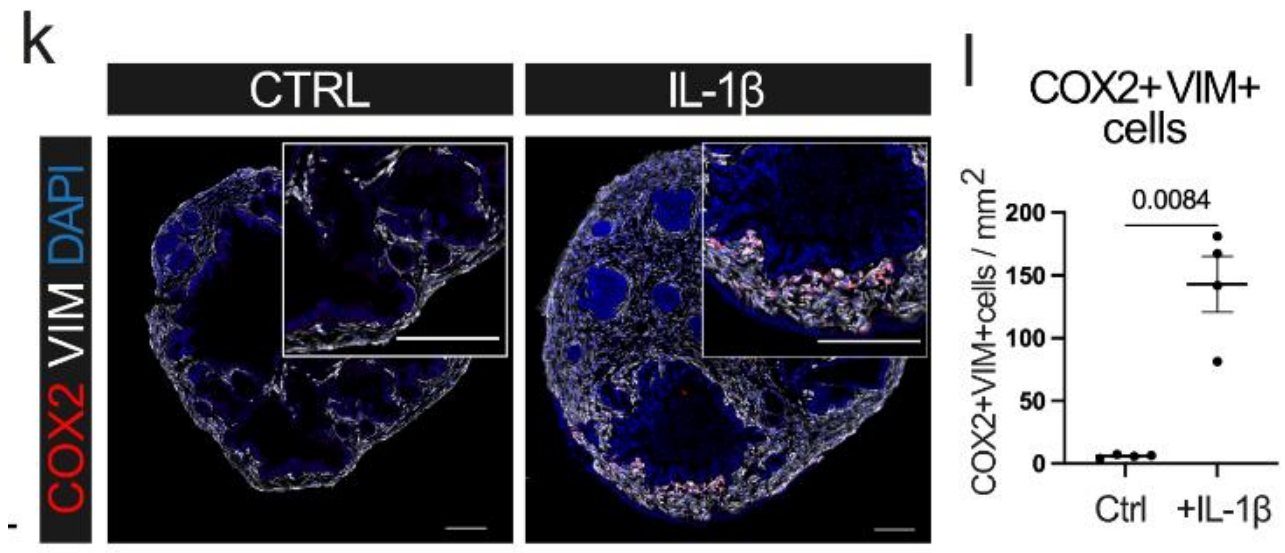

Figure6

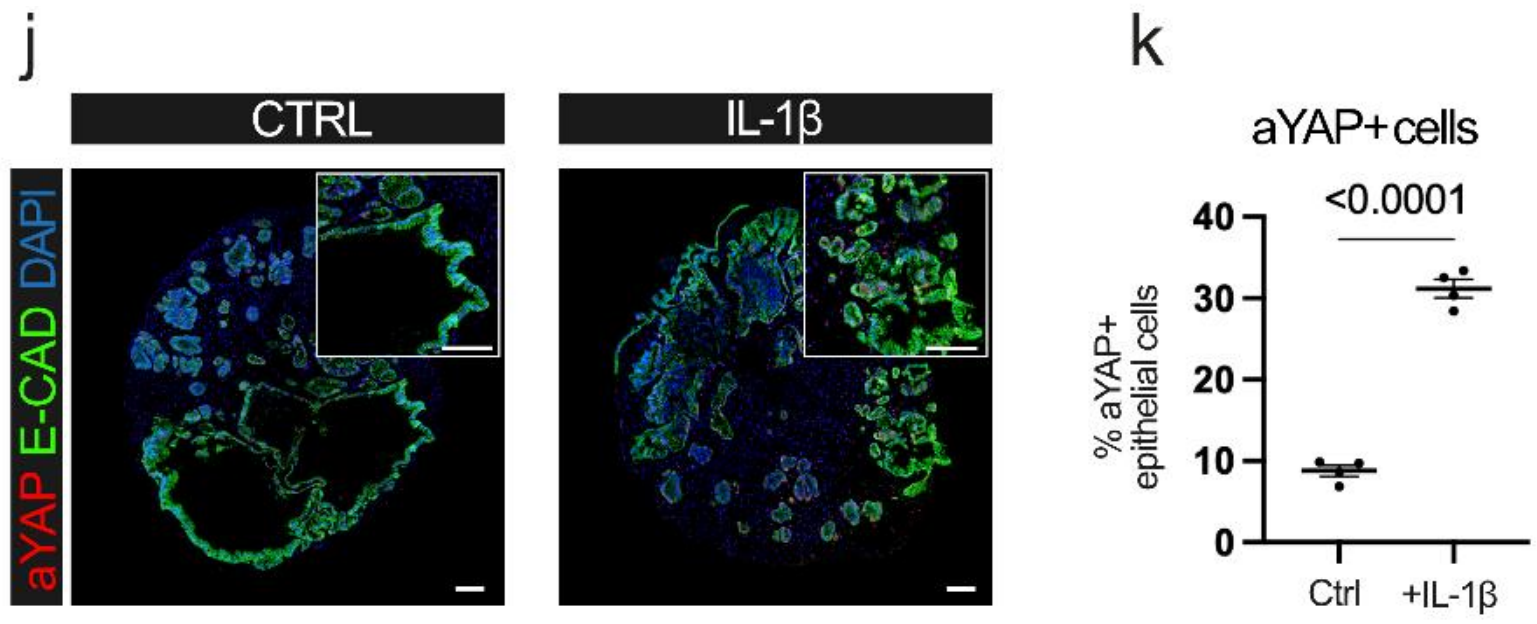

5. H. pylori infection is known to begin with colonization in the gastric antrum and, over time, lead to the loss of parietal cells in the corpus, thereby increasing the risk of cancer development in the corpus region. In this context, I would appreciate it if you could elaborate on why your study focused specifically on the antrum, and, if available, provide any histopathological data or observations regarding H. pylori-induced pathology in the mouse corpus, along with further explanation of these findings.

We thank the reviewer for their comment. We chose to focus on the antrum because this (along with the transition zone between antrum and corpus) is the region where human gastric cancer most frequently occurs (now specified in lines 107-108). Although metaplasia is often seen in the corpus and the occurrence of metaplasia there is linked to a higher risk for cancer development, the cancer itself is often in the antrum and in the transition zone between antrum and corpus regions. Moreover, also in murine models the hyperplastic areas are mostly observed in the transitional zone.

Reviewer #3 (Remarks to the Author):

This manuscript by Beccaceci et al. investigates how Helicobacter pylori (H. pylori) infection promotes fetal-like epithelial reprogramming in the gastric antrum through suppression of BMP signaling and

activation of an epithelial–immune–stromal signaling cascade. The authors demonstrate that inhibition of BMP signaling—either through infection or epithelial-specific deletion of Bmpr1a—leads to NF-κB–mediated proinflammatory chemokine expression and immune cell infiltration. IL-1β, produced by infiltrating macrophages and neutrophils, induces COX2 and prostaglandin E2 (PGE2) production in stromal cells, which in turn activates YAP signaling in the adjacent epithelium, promoting a regenerative, fetal-like state. Deletion of Il1r1 in stromal cells blunts these changes and prevents H. pylori–induced hyperplasia.

While this study adds potentially valuable mechanistic links between microbial sensing, inflammation, and epithelial regeneration, there are significant concerns regarding the novelty of the findings, the specificity and clarity of the experimental design, the anatomical limitation to the antrum, and the depth of mechanistic validation. Several key conclusions appear overstated based on the current data, and additional experiments are required to substantiate them.

Many of the experiments presented in this study are largely descriptive and somewhat limited in scope. For example, early in the Results section, the authors claim that epithelial Bmpr1a knockout mimics H. pylori infection. However, this conclusion appears overstated, as it is based mainly on histological observations of mucosal hyperplasia and increased proliferation, along with a few selected bulk RNA-seq snapshots. A more comprehensive and quantitative comparison is necessary to support this claim.

Additionally, key aspects of the proposed mechanism remain insufficiently characterized. For example, the cellular identity of IL-1β–producing immune cells and the specific stromal subtypes responding to IL-1β are not adequately defined. To strengthen the mechanistic claims and provide high-resolution insights into epithelial–immune–stromal interactions, incorporation of single-cell RNA sequencing and/or spatial transcriptomic analysis is strongly recommended.

Major Comments

1. Limited Novelty and Overlap with Prior Work

The role of BMP signaling in gastric epithelial homeostasis and hyperplasia is well documented (Bleuming et al., 2007; Ye et al., 2018; Takabayashi et al., 2023; Huh et al., 2010), including by the authors in prior work using Bmpr1a-deficient models (Kapalczybska et al., 2022). While the involvement of immune and stromal components adds some depth, the conceptual novelty is limited. The authors should clearly define what aspects of the current study are new.

We would like to thank the reviewer for this critical comment. In the revised manuscript we have now highlighted the new insights provided by this study more clearly. We particularly emphasized the fact that infection-driven proliferation is not simply driven by an expansion of homeostatic stem cells, but rather by reprogramming of the epithelium into the fetal-like state, which is orchestrated by multicompartmental communication involving immune, stromal and epithelial cells. In addition, we identify IL-1β as the crucial signal that triggers reprogramming. These data are not only relevant to understand gastric pathology, but are also of relevance to other fields: epithelial fetal-like reprogramming has been observed in the small and large intestine, while mechanisms are not well-understood. It is likely that mechanisms we describe here are also translatable to other tissues underscoring the value and novelty of our findings.

To further strengthen our study, we have now included various new experiments and data sets, including a new scRNA sequencing data set from gastric epithelial cells (from *Bmpr1a* KO and *H. pylori* infected mice), to obtain a detailed picture of proinflammatory and regenerative responses

(**Figure 3b**, **Supplementary Fig. 1e-f**). These additional data bolster the overall impact of this study, which reveals the molecular mechanisms behind the interplay between epithelium, stroma and the immune system that ultimately links inflammation and regeneration. Although certain aspects of the relevant pathways were indeed already known, we believe our study is the first to bring together the full picture, by considering all three elements in our experiments.

2*. Anatomical Scope and Misinterpretation*
The study focuses solely on the antrum, yet the Introduction and Discussion cite studies on the corpus and cardia without distinguishing anatomical context. This may lead to misinterpretation. The authors should clearly specify the region of interest throughout and acknowledge that the findings may not generalize to other gastric compartments.

We agree that it is important to explicitly state that the focus of this study was on the antrum to avoid misinterpretation We have now revised the text accordingly (Lines 107-108). We would like to point out that while corpus pathology has been extensively studied and metaplastic responses in the corpus are linked to gastric carcinogenesis, the malignant lesions themselves are more frequently found in the antrum, which is a relatively understudied anatomical site.

3. Mechanistic Gaps in BMP–NF-κB–YAP Crosstalk
The proposed link between BMP suppression and NF-κB/YAP activation lacks mechanistic clarity. BMP signaling classically proceeds through SMADs, and it is unclear why non-SMAD pathways are activated here. Suggested additional experiments include:

o Assessing SMAD activation in response to BMP2 and testing whether SMAD inhibition alters NF-κB/YAP activation or chemokine expression.

o Western blot of total and phospho-IKKα/β, IκBα, p65, YAP, and upstream regulators (e.g., LATS1/2), with densitometric quantification.

o Time-course and dose-response experiments with BMP2.
o Use of NF-κB and YAP inhibitors or genetic mutants.

We would like to thank the reviewer for their suggestions. We now provide several sets of new data that together provide additional mechanistic depth.

First, we dissect the NF-κB signaling pathway using Western blotting and provide additional data demonstrating NF-κB activation in organoids grown in full medium, as well as the suppression of this response upon BMP stimulation. We also confirm that BMP causes SMAD phosphorylation. Furthermore, we find that the basal YAP activity in organoids is already present in the absence of proinflammatory stimulation (which implies baseline regenerative activity in this system) and is suppressed by BMP. This demonstrates that BMP directly suppresses YAP, and that its loss renders cells capable of entering and sustaining the regenerative state (**Figure 3g-h – Quantifications: Supplementary Figure 3g-h**).

Figure3

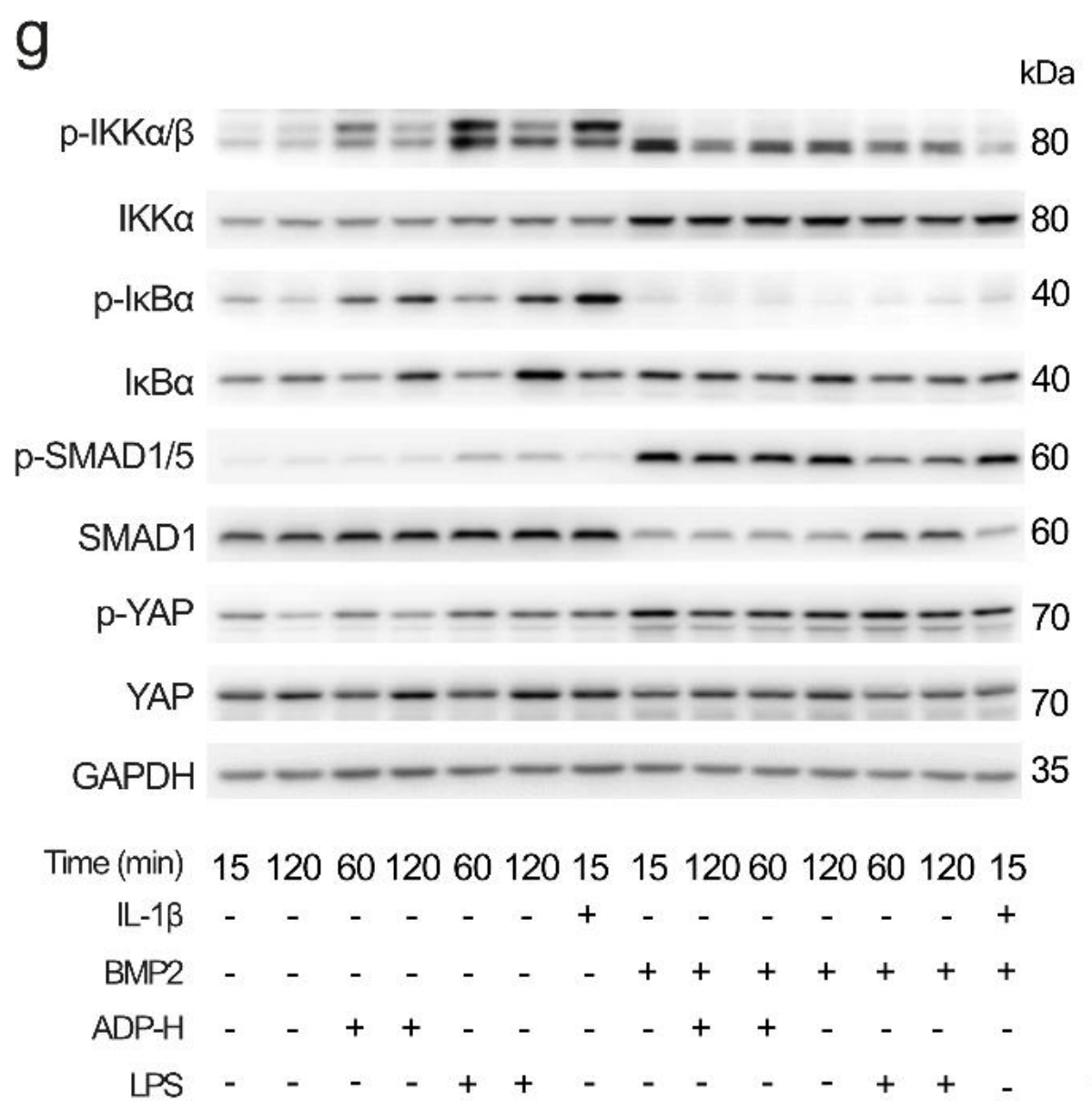

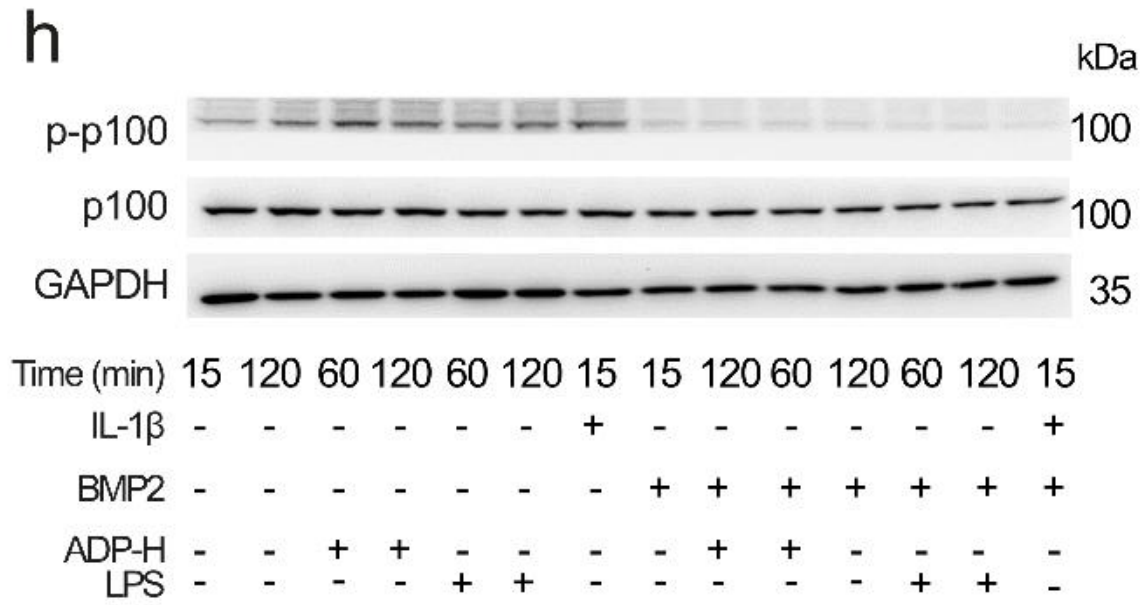

SupplFigure3

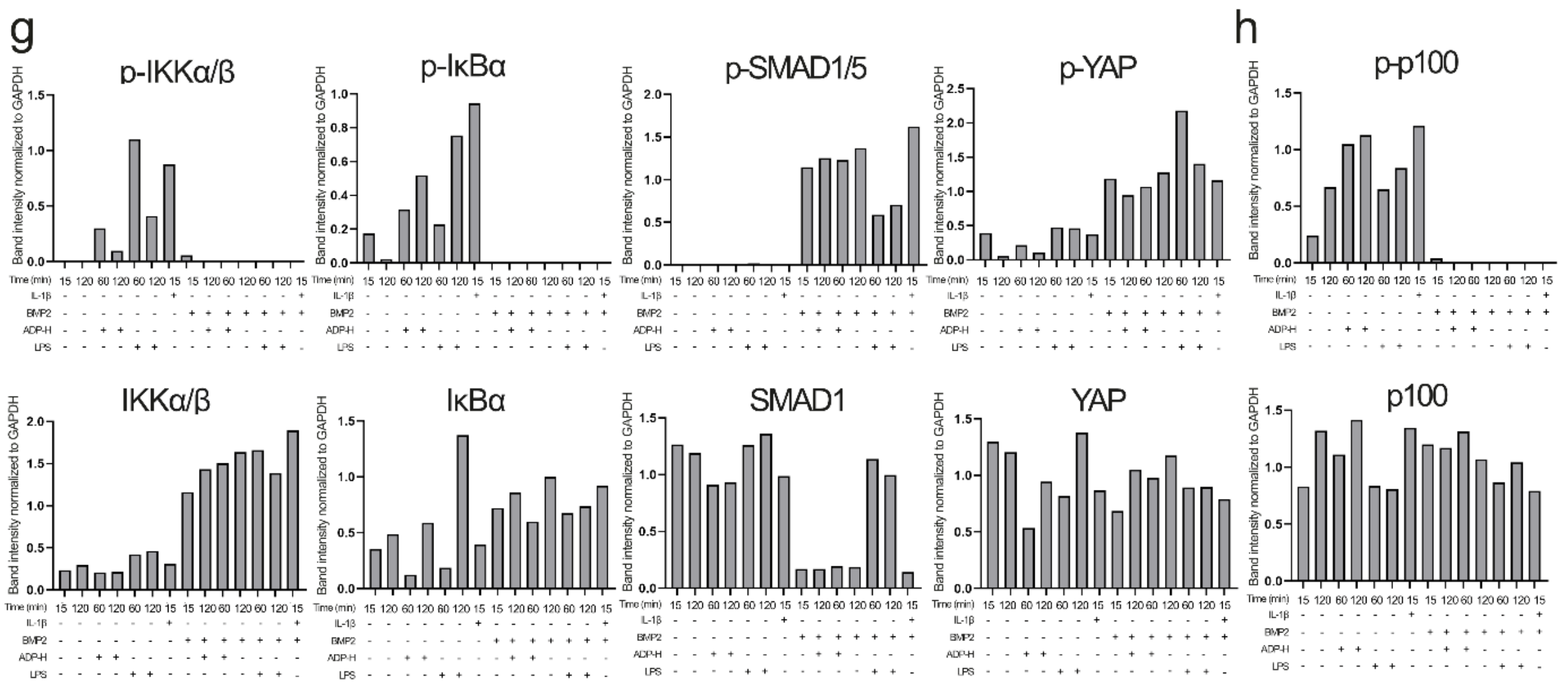

We also provide new data from short-term treatment (2 hours) of organoids with BMP2: the results reveal that this is sufficient to induce BMP target genes such as Id1, while differentiation markers are not yet significantly altered. However, exposure of these cells to LPS (added simultaneously to BMP2 on the last day of culture) triggers a diminished chemokine response – this means that BMP-driven anti-inflammatory effects occur early and precede the stem cell loss/enrichment of Muc5ac positive cells (**Supplementary Figure 4e-g**).

SupplFigure4

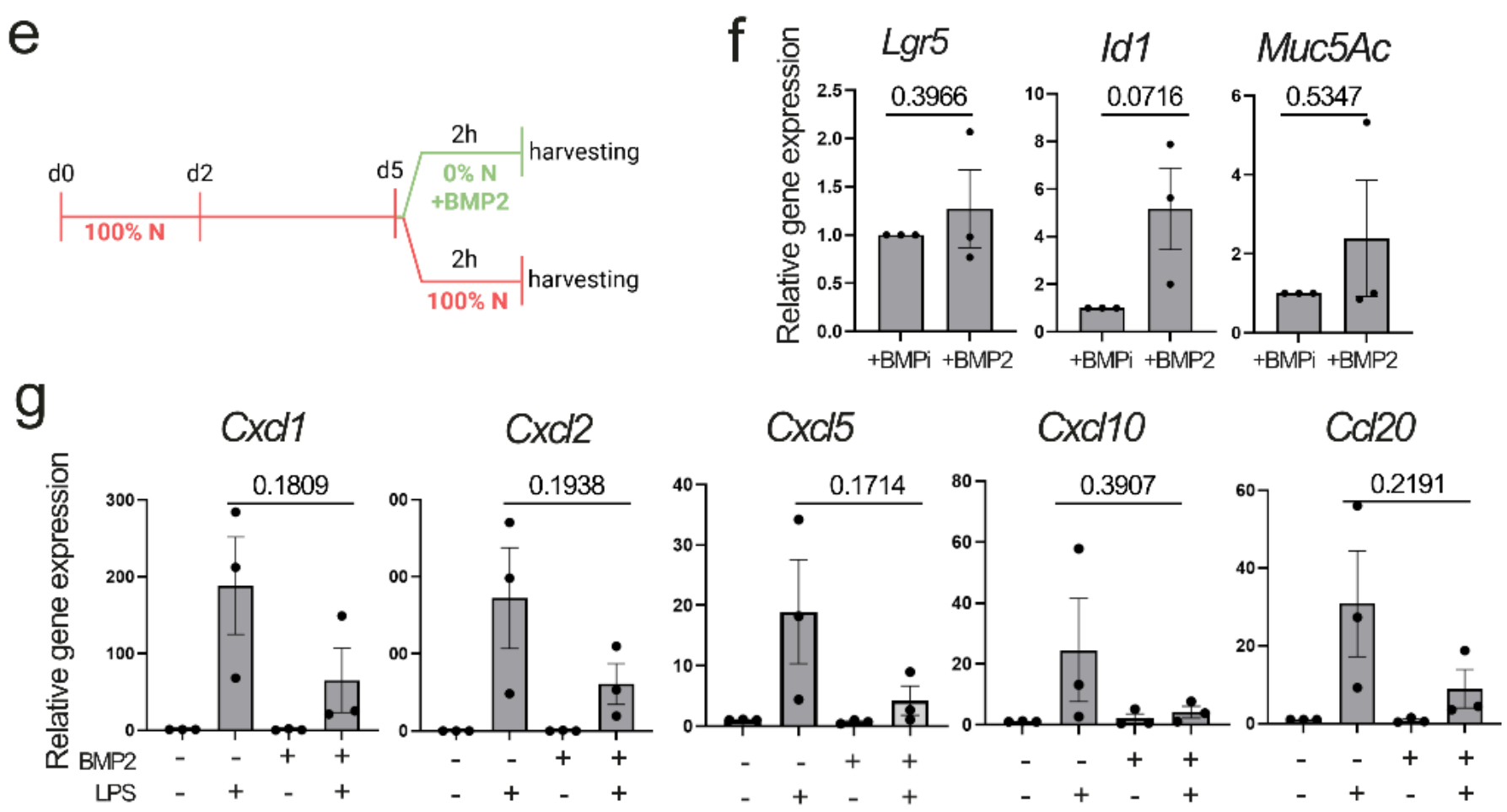

As for the use of inhibitors, we have tried to assess the effects of BMP or YAP signaling inhibition *in vitro* in our organoid system. However, we were not able to achieve a convincing downregulation of the signaling activity.

In various media conditions LDN193189 did not affect BMP target gene expression.

Regarding YAP inhibition: our attempts to inhibit YAP via Verteporfin turned out to be technically challenging (no YAP inhibition early on, highly cytotoxic effects at later time points). It is possible that this is due to off target effects, alternatively YAP signaling might be critical for organoids and its pharmacological inhibition may have severe effects on cell survival.

Given these technical constraints, and because the conclusions of this study do not rely on direct pathway inhibition, we believe that these experiments are not essential for supporting the main findings and cannot be readily implemented in a robust manner within the scope of the current study.

4. Lack of Temporal and Spatial Context in Bmpr1a KO Model
The timing and progression of changes following tamoxifen induction are not shown. Time-course analyses of proliferation, stem/differentiation markers (Lgr5, Lgr4, Axin2, Muc5ac), and signaling molecules are necessary. Histological data rely heavily on high-magnification images of limited gland numbers; low-magnification overviews and robust quantification should be provided.

We now provide additional data from an early time point upon BMP depletion *in vivo* and show that the effects are already evident, although the severity of pathology is less pronounced (**Supplementary Figure 5a-b**).

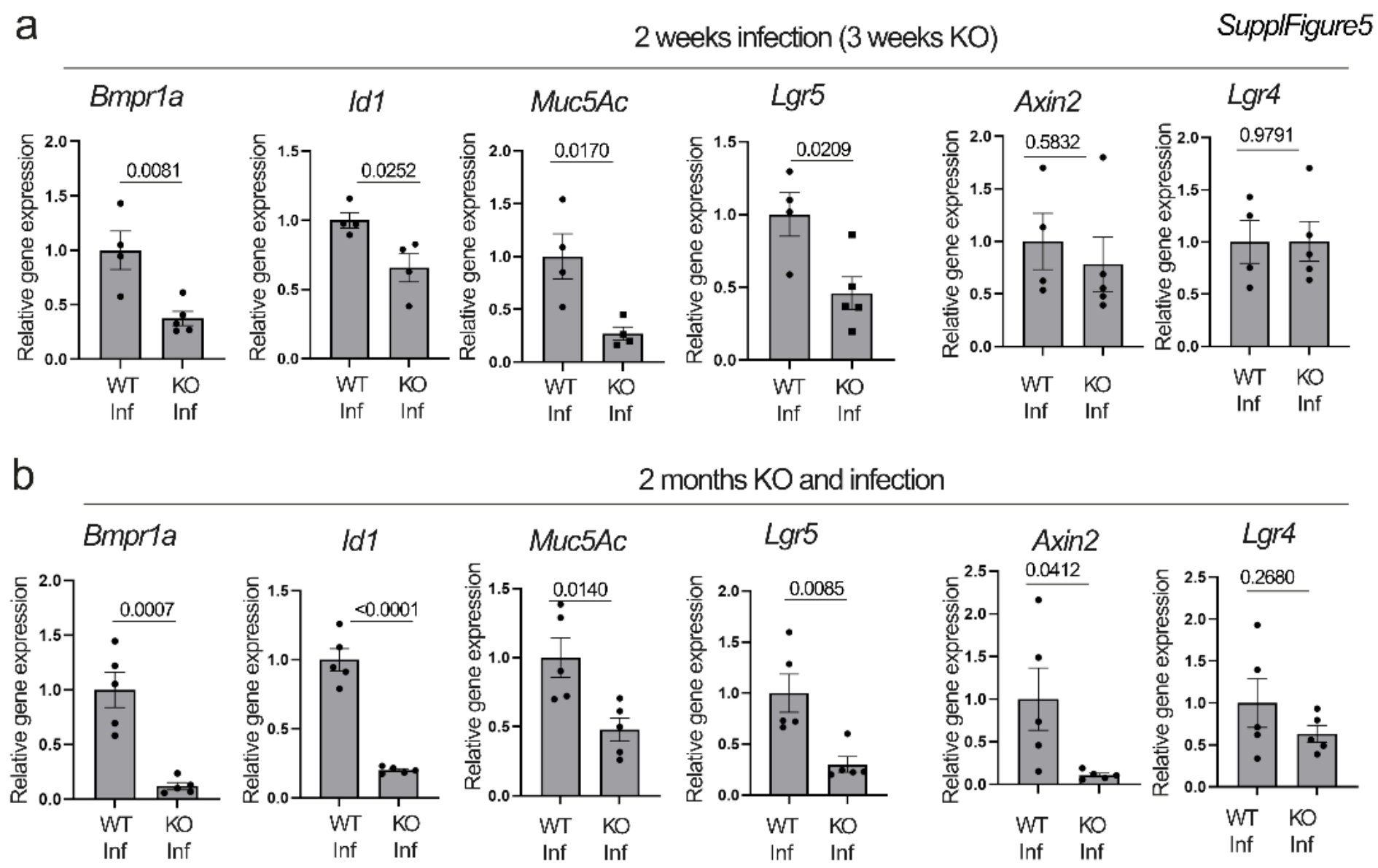

Furthermore, we provide additional low-magnification images and quantifications (**Supplementary Figure 1d , Figure 1 a-e-i-j-k,**).

SupplFigure1

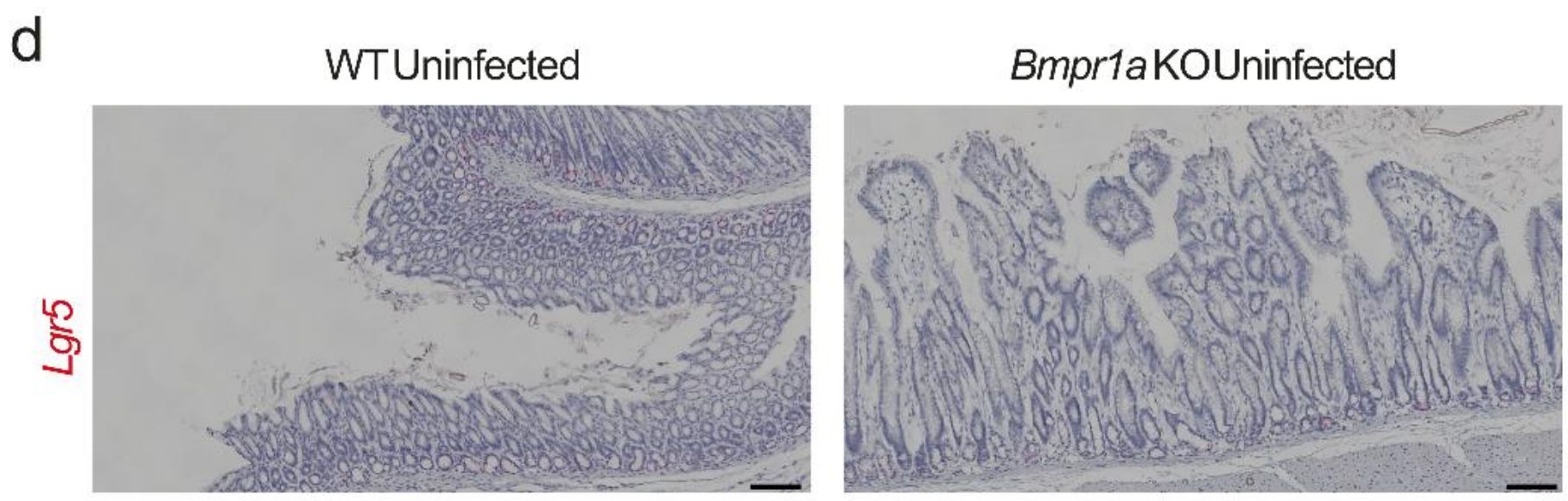

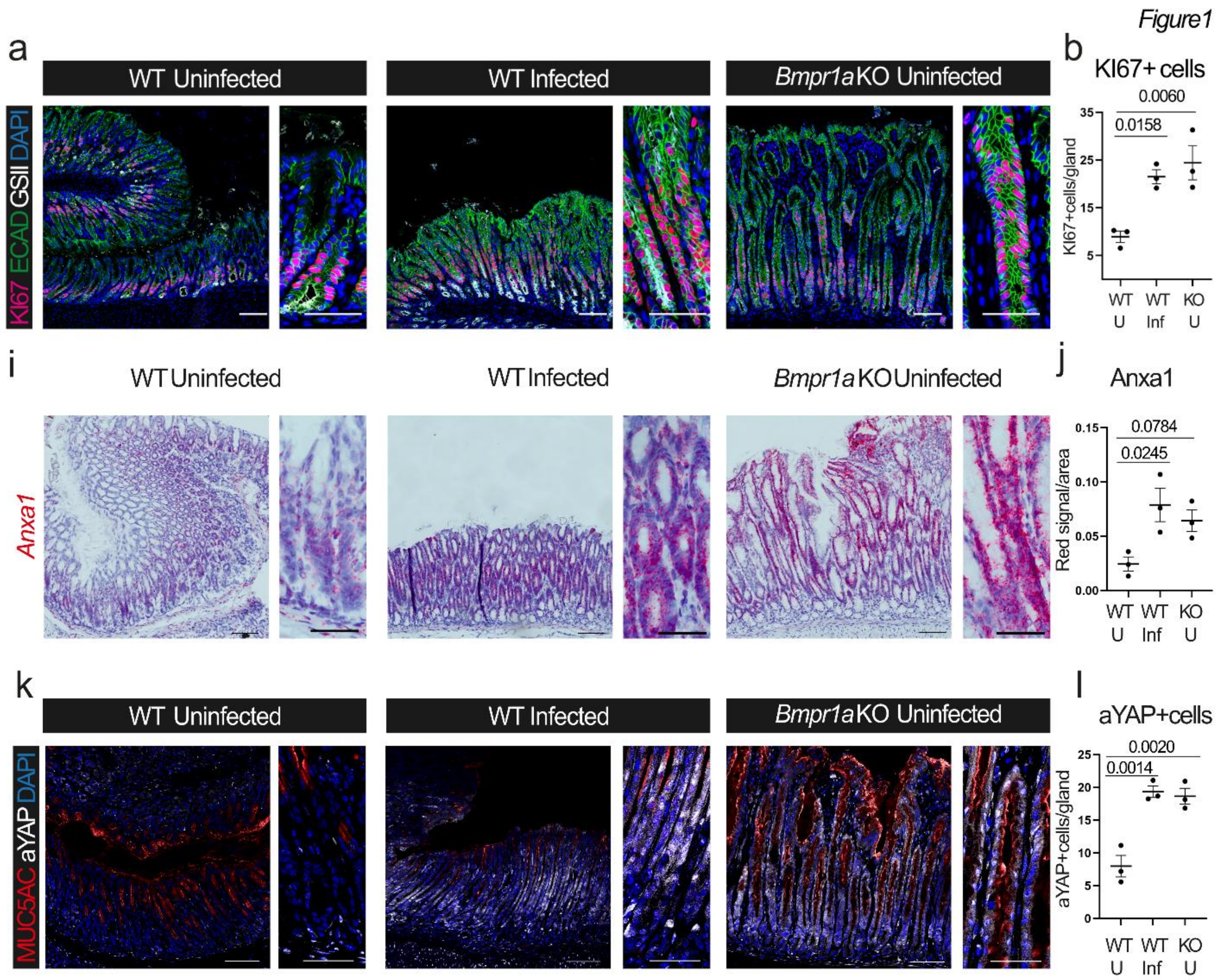

5. Confounding Organoid Conditions and BMP Source
Organoid culture conditions are inconsistently described.

Because the base medium contains Noggin (a BMP antagonist), it is difficult to isolate the effects of BMP modulation. Ideally, experiments should manipulate BMP ligands alone.

Additionally, the source of BMPs in organoids is unclear—are epithelial cells themselves producing BMPs? BMP ligand expression in WT and Bmpr1a-KO organoids should be tested.

We thank the reviewer for this comment. To address the first point and make it easier for readers to understand the culture conditions, we have now added a scheme to highlight how the organoid experiments were conducted (**Figure 2b, Figure 2h, Supplementary Figure 2a, Supplementary Figure 3b, Supplementary Figure 4e**).

We chose to grow organoids in full medium to mimic the stem cell niche that also is known to produce BMP inhibitors. The exposure of cells to inhibitors as well as ligands at the same time represents in our view the physiological situation. We would also like to refer to our previous paper that dissected the dosing of BMP with and without BMP inhibition in detail (Kapalczynska, Lin et al 2022).

We additionally analyzed the expression levels of BMP ligands in WT and *Bmpr1a* KO cells (**Supplementary Figure 2g**), observing that Bmp2 and Bmp7 are expressed by WT and KO epithelial cells. We confirmed this with our scRNA sequencing data, which shows that Bmp2 and Bmp7 are expressed, while Bmp4 and Bmp5 could not be detected (**Supplementary Figure 2i**). We have now included these findings in the text (lines 192-194).

SupplFigure2

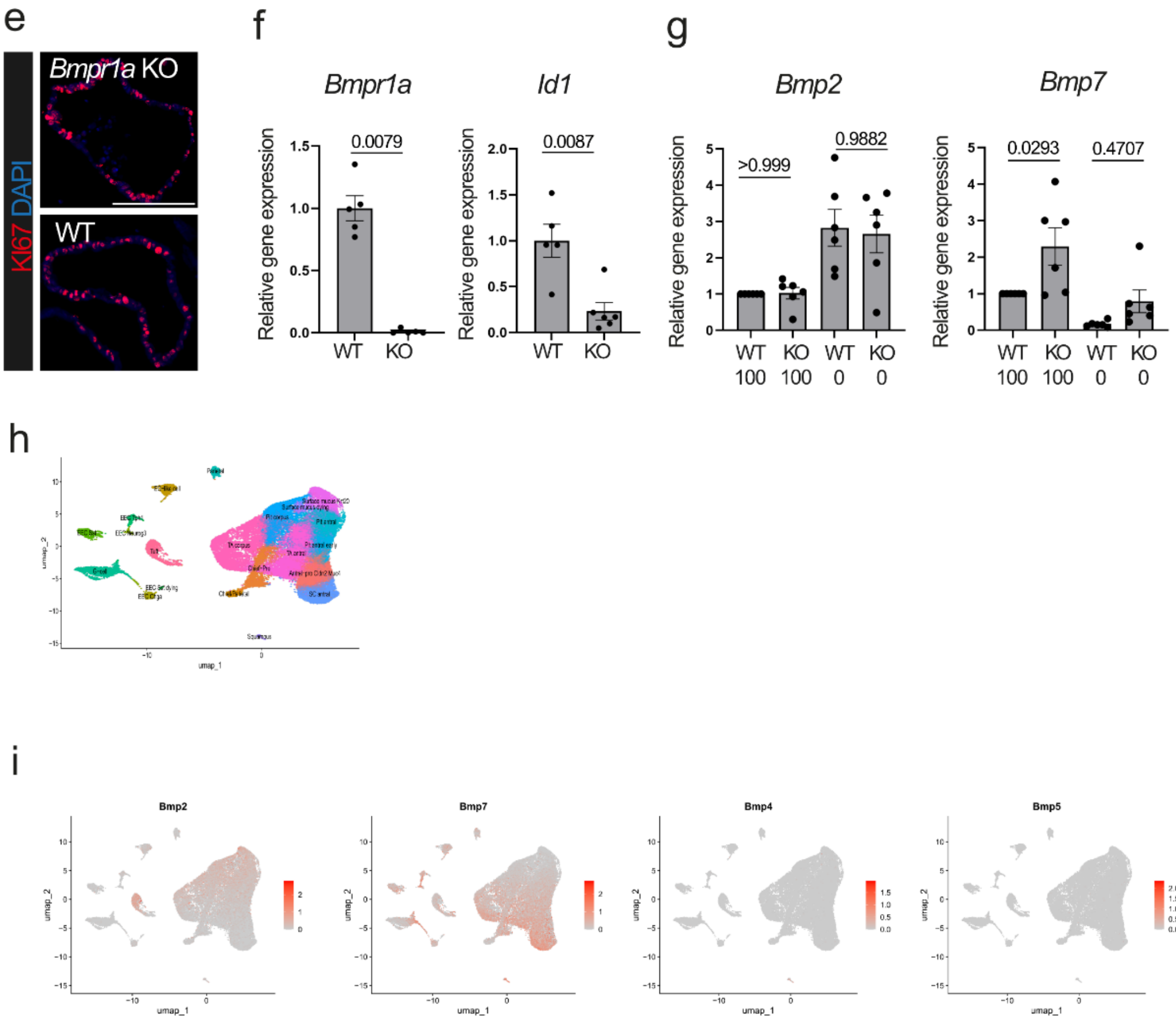

6. Interpretation of NF-κB Activation and p65 Staining

Figure 3B's p65 staining is ambiguous. The cytoplasmic localization of p65 in isthmal progenitors does not clearly reflect NF-κB activation. Co-staining with markers of proliferation/differentiation and quantification of p65+ cells (number, position, nuclear localization) are needed. Western blots in Figures 3D and 3F should include total protein levels, not just phospho-signals, and should be presented by condition and time point to enable interpretation.

We agree that the p65 staining was not ideal due to the fact that it is difficult to discriminate nuclear from cytoplastmatic signal. It is well known that NF-κB signaling shows fluctuating activity, which is not always easy to measure by staining in vivo. Therefore, we decided to analyze the expression of pro-inflammatory cytokines in epithelial cells via scRNA-sequencing analysis, both

upon infection and in the *Bmpr1a* KO model (**Figure 3b**). This method proved more powerful for determining which epithelial cell populations become pro-inflammatory upon infection.

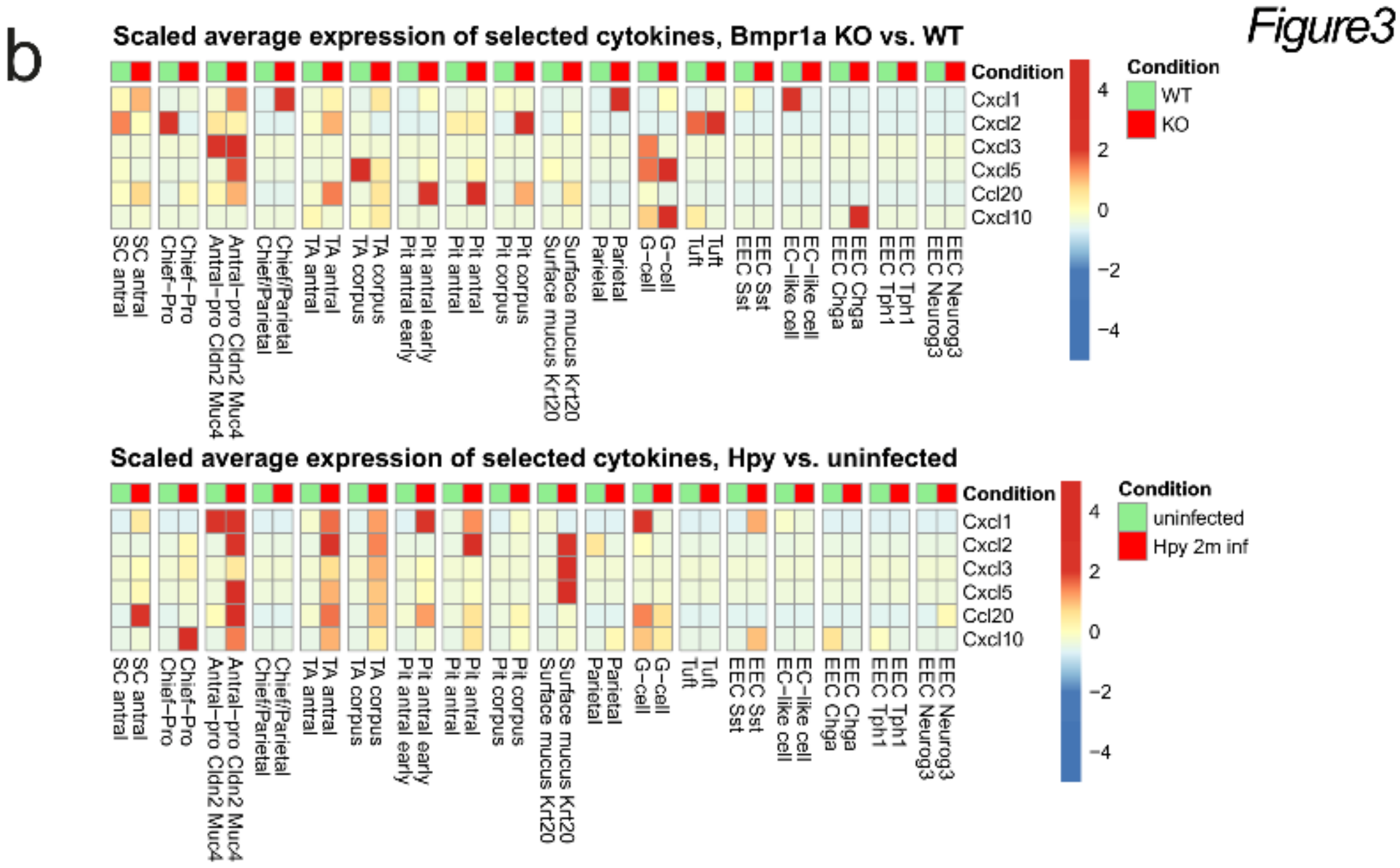

We thank the reviewer for their suggestion regarding the organoid Western blots. We have now included new blots for total proteins (**Figure 3g-h-Supplementary 3g-h,** see above at Point3) together with time points.

Overall, these data clearly demonstrate epithelial NF-κB activation as well as its suppression via BMP signaling.

7. Inadequate Justification and Testing of IL-1β Axis
IL-1β is introduced abruptly, with no rationale for its prioritization over other cytokines. The source of IL-1β (macrophages, neutrophils) is not directly demonstrated—co-staining or FACS-based validation is required. Foundational literature on IL-1β in the stomach (e.g., Tu et al., 2008; Serizawa et al., 2014) should be cited. IL-1α, another ligand of IL1R1, is not discussed—its expression and role should be considered.

We appreciate the insight that the introduction of IL-1β felt abrupt. As pointed out by the reviewer, IL1-β has been previously linked to gastric pathology, but the mechanisms were not

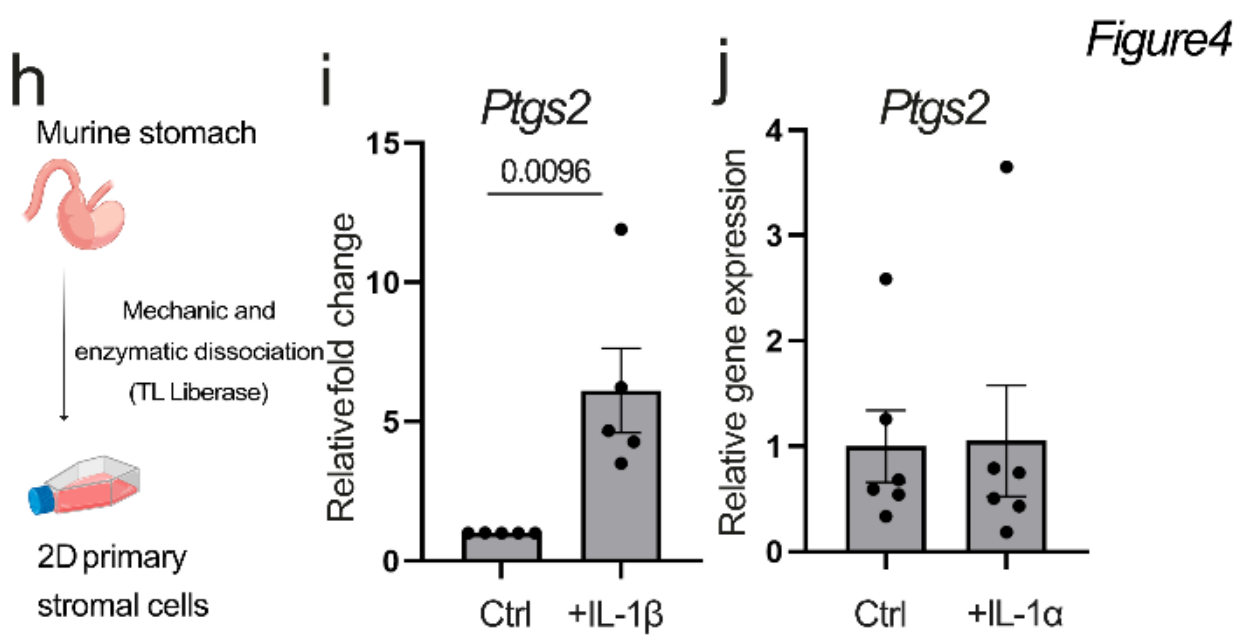

fully elucidated. We now refer to the key papers as indicated by the reviewer to explain the

rationale for exploring this key cytokine. We also provide new data on the cellular source of IL1-β and explore IL1-α as an alternative activator, showing that it fails to induce the same effect (**Figure 4h-j**).

8. IL1R Knockout Analysis Is Incomplete
The Il1r1 stromal KO model is under-analyzed. Its phenotype under uninfected conditions is not described. Since in situ hybridization shows epithelial Il1r1 expression, an epithelial-specific KO (e.g., Axin2-CreERT2) should be tested. Markers such as Muc5ac, p65, chemokines, and BMP components should be examined. The increased H. pylori CFU in KO mice also requires mechanistic interpretation.

We now provide more data and analysis on this issue (**Supplementary Fig. 8a-d**).

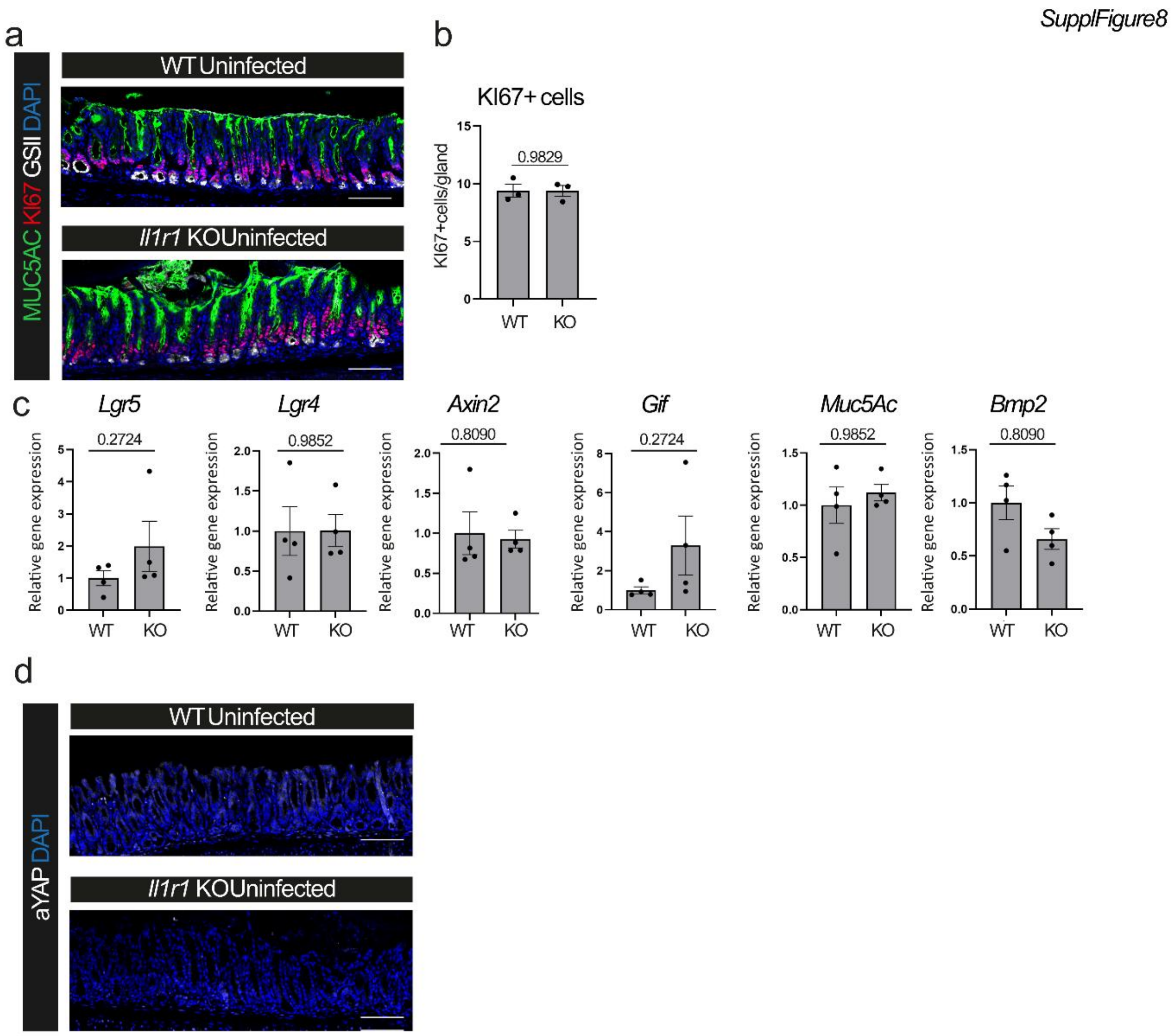

Our data indicate that IL-1β signaling in the stroma upon infection is important for tissue responses. This is based on stromal cell cultures treated with IL-1β. We have also performed epithelial organoid experiments where we treated organoids with IL-1β and did not observe activation of YAP signaling. We therefore believe that stroma-specific KO mice are the most appropriate model. While we in principle agree that it would also be interesting to confirm in vivo that epithelial IL-1β signaling is not involved, these experiments would fall outside the time frame of this revision (in addition to the time for breeding, this would require an additional, time-consuming approval process with uncertain outcome). We hope the reviewer agrees that this experiment would most likely provide negative data and is not essential.

Regarding the responses to stromal Il1r1 depletion in the absence of infection, we did not observe any phenotypic alterations at baseline without infection, which is explained by the fact that in this condition IL1β-expressing cells are absent or sparse (see Fig. 4 fg).

Regarding the issue of increased colonization levels in *Il1r1* knockout mice, we show that the lack of responsiveness to IL-1 in the stroma inhibits the induction of antibacterial genes normally observed upon infection. This leads to the seemingly paradoxical effect of increased bacterial expansion with simultaneous failure to induce tissue reprogramming and obvious pathology (**Figure6**).

9. Unclear BMP Dynamics During H. pylori Infection
The manuscript lacks time-course data on BMP ligand, receptor, and SMAD expression following H. pylori infection. Furthermore, it is unknown whether H. pylori infection has additional or redundant effects in Bmpr1a-deficient mice. These experiments are essential to distinguish direct infection-induced changes from BMP-dependent ones.

We thank the reviewer for this comment. We have previously performed an in-depth characterization of BMP signaling upon *H. pylori* infection (Kapalczynska, Lin et al 2022), to which we now refer in the text (lines 107-112). In Kapalczynska, Lin et al 2022 we showed that infection triggers a loss of BMP ligands early upon infection and that this is followed by increased expression of BMP inhibitors. Loss of BMP signaling is not the only response seen upon *H. pylori* infection, however, various features observed in uninfected *Bmpr1a* KO mice resemble those of infected mice.

10. Ambiguous Interpretation of PGE2/COX2 Axis
The focus on COX2/PGE2 signaling in Figure 4 appears sudden and lacks explanation. Was this pathway selected from unbiased transcriptomic analysis? COX2 is involved in multiple prostanoid pathways (e.g., PGI2, TXA2, PGD2), none of which are examined. The COX2 IHC appears overexposed and does not demonstrate epithelial or immune expression. PGE2 receptors and downstream YAP regulators (e.g., EP4, LATS1/2) should be analyzed. The spatial disconnect between COX2 (pit) and aYAP (isthmus) is not addressed.

We thank the reviewer for this comment. We have now included a more thorough analysis of the genes involved in the synthesis of different prostanoids. Our microarray data show that, upon treatment with IL-1β, gastric stromal cells selectively upregulate the expression of genes involved in the synthesis of PGE2, rather than the other prostanoids (**Figure 4m-n**).

Figure4

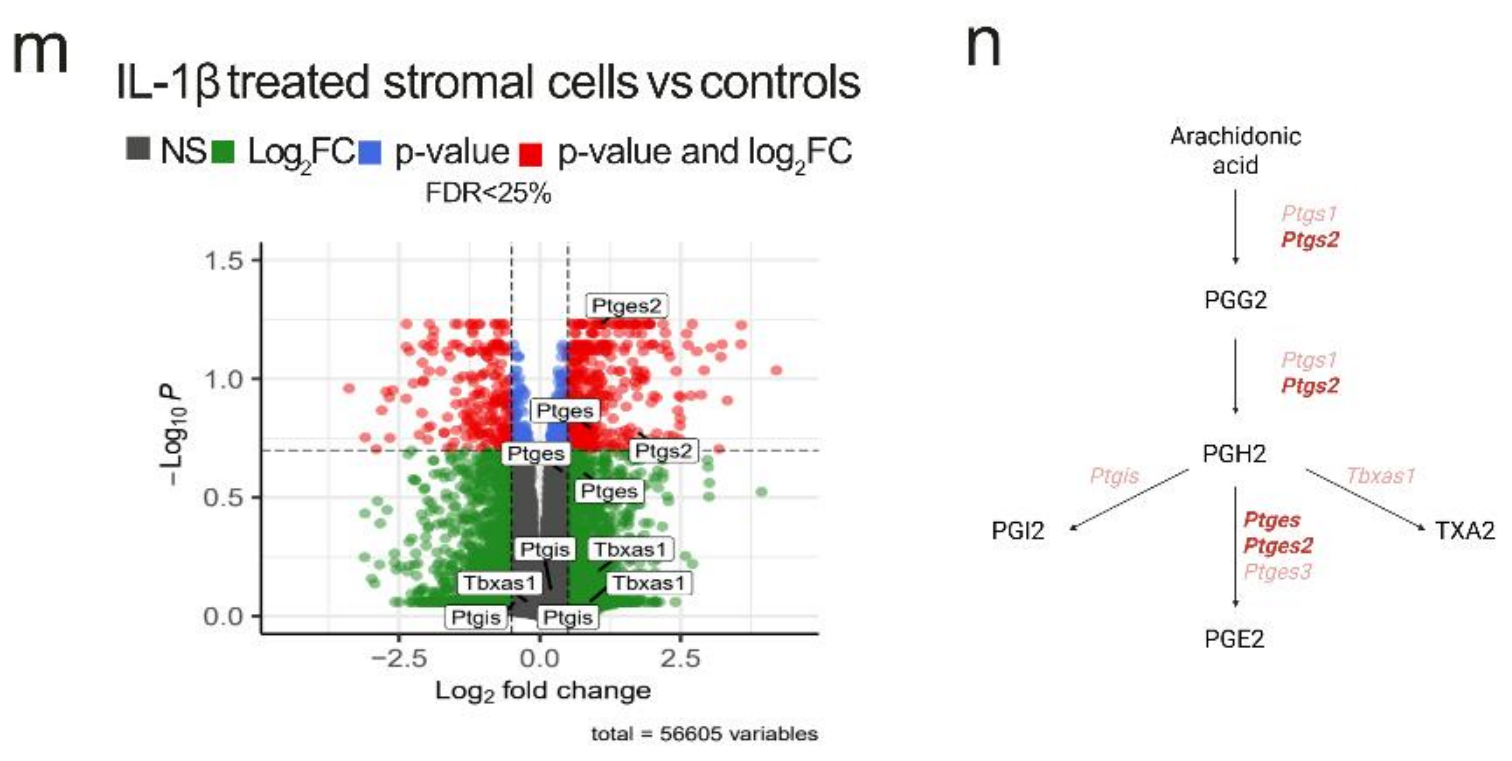

Moreover, we now provide a better quality COX2 IHC image (**Figure 4a**).

Figure4

a

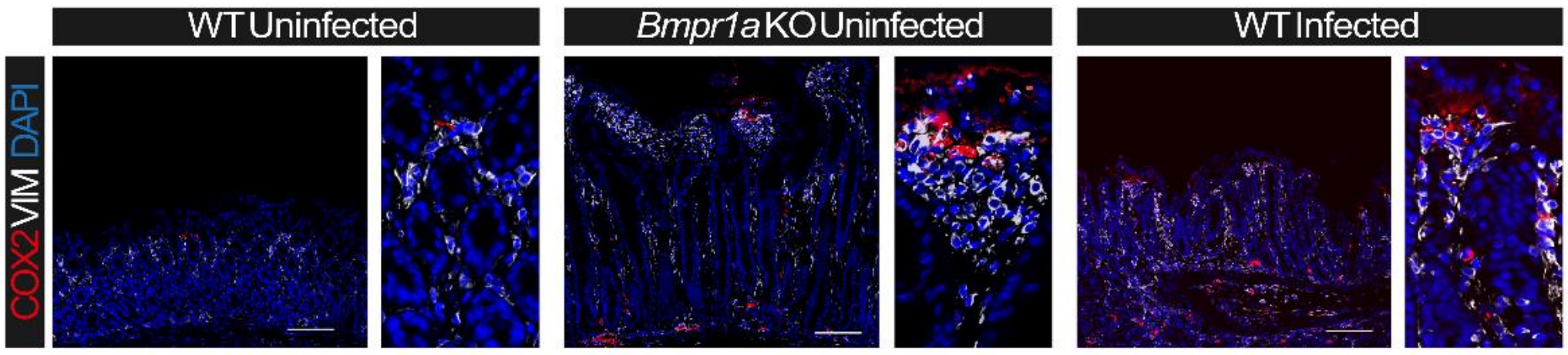

Additionally, we analyzed the expression levels of PGE2 receptors in our scRNA-seq dataset, finding that only Pger4 (EP4) is expressed in gastric epithelial cells (**Supplementary Fig. 6e**). We have included these results in lines 331-333.

SupplFigure6

e

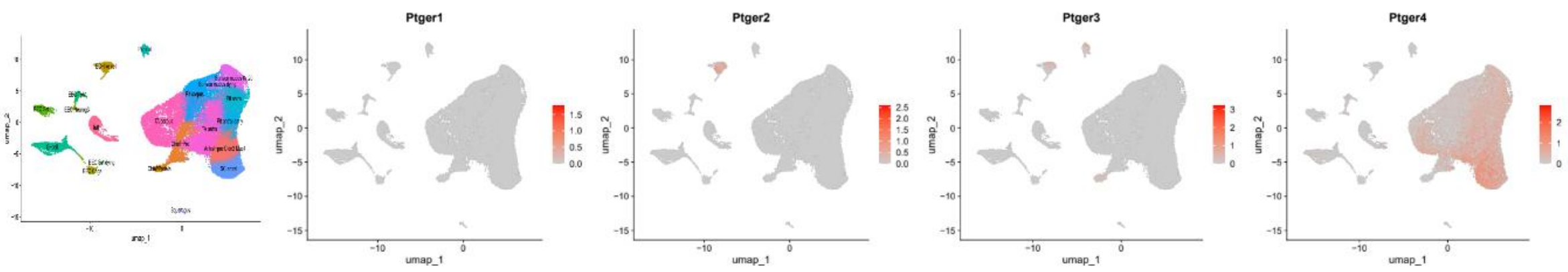

Regarding the spatial disconnect between COX2 (pit) and aYAP (isthmus): Our data show that there are multiple stromal cell populations that express COX2 (**Figure 4)**, confirming our finding that following infection stromal cells along the entire gland axis stain for COX2 **(Figure 4a)**. To address this point in more detail, we also analyzed a scRNA sequencing data from a previously published dataset (Manieri et al, 2023) (See below in Point 11).

11. Stromal Heterogeneity Overlooked
Recent scRNA-seq studies (e.g., Manieri et al., 2023) emphasize the diversity of gastric stromal cells. The IL1R1+ population appears distinct from the Rspo3+ myofibroblasts previously described by the authors. Yet IL-1 induces Rspo3 in culture, implying potential heterogeneity. More refined cell classification and functional testing (e.g., single-cell, lineage tracing) are needed. (FIGURE4)

Figure4

o

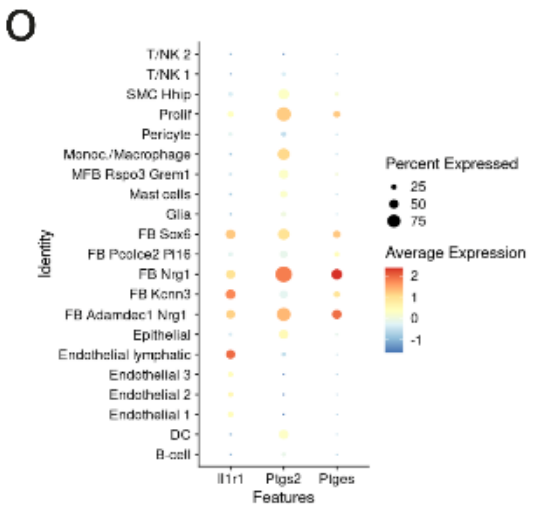

SupplFigure6

g

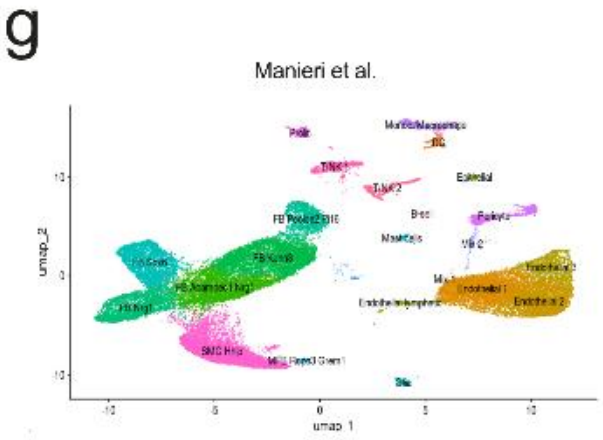

We fully agree with the reviewer regarding the importance of stromal heterogeneity. We have now exploited the data published by Manieri et al., 2023 to analyze which stromal subpopulations express Ptgs2, together with Ptges, which is also involved in PGE2 synthesis (**Figure 4o, Supplementary Figure 6g**).

Minor Comments

• Specify whether LPS stimulation is TLR4-dependent. H. pylori-derived LPS may be more relevant than Klebsiella LPS.

The effects that we describe are reproducible for different pro-inflammatory stimuli, namely *Klebsiella* LPS which acts via TLR, ADP-heptose (normally translocated through the T4SS but also able to diffuse through the cell membrane) and TNF-α, which binds to the TNF Receptor (TNFR). Therefore, we believe that the effect of BMP signaling on the pro-inflammatory ability of epithelial cells does not act at the receptor level, but rather downstream in the signaling cascade.

• Clearly define organoid culture conditions, especially the presence or absence of Noggin.

We have now provided schematics of how the organoid experiments were performed, to ensure the experimental set-up is clear to the reader.

• Provide total protein blots (not just phospho-signals) in Figures 3D and 3F

Western blots for total protein are now provided.

• Improve image quality for COX2 (Figure 4B) and include low-magnification overviews of histological data.

We have now provided an improved quality image for COX2, and included lower magnification overviews as requested.

• Explain how BMP2 suppresses chemokine induction (Figure 3C)—is this due to cytotoxicity at high concentrations?

Our data demonstrate that BMP signaling in epithelial cells blocks NF-κB signaling, causing a suppression of chemokine expression. We do not believe that this is a cytotoxic effect – while we observe cell differentiation, our data indicate that cells are alive.

We were pleased to see that Reviewers 1 and 2 were fully satisfied with our responses and revisions. We have now carefully addressed the remaining concerns raised by Reviewer 3, made every effort to clarify our conclusions, and performed additional experiments where feasible within the revision period.

Reviewer #1 (Remarks to the Author):

The authors have addressed all points I raised in the previous review.

Reviewer #2 (Remarks to the Author):

I have reviewed the responses to the points we raised and find the explanations appropriate. However, the data presented in Supplementary Figure 1 do not appear to be consistent with the main text. For example, panels a and c in Supplementary Figure 1 seem to need to be swapped to align with the revised text. Please carefully recheck all figures and correct them to ensure consistency with the text.

We thank the reviewer for their positive evaluation of our study and apologize for the inconsistency that has now been carefully addressed.

Reviewer #3 (Remarks to the Author):

The authors have made a substantial effort to address the reviewers' previous comments and have added additional analyses and experiments in the revised manuscript. These additions improve several aspects of the study. However, several of the key conceptual issues raised in the previous review remain insufficiently resolved. Importantly, the concerns discussed below were already raised during the previous round of review, and although the authors have attempted to address them in the response letter, the underlying issues remain only partially addressed and in some cases unresolved.

Major Comment 1-1 – Whether Bmpr1a deletion truly mimics H. pylori infection
In my previous review, I raised concerns regarding the interpretation that loss of BMP signaling "mimics" the epithelial and inflammatory responses induced by Helicobacter pylori infection, and questioned whether H. pylori infection exerts additional or redundant effects in Bmpr1a-deficient mice. In the response letter, the authors cite their previous study (Nature Communications, 2022) as supporting this concept. While that work provides important context, a careful comparison between the two studies raises additional questions regarding the relationship between BMP suppression and infection-induced signaling.
In the 2022 study, the comparison between BMP-deficient and infected conditions primarily relied on measurements of gland height and epithelial proliferation (Ki67). By contrast, in the current manuscript, additional readouts—such as immune cell infiltration, YAP activation, and stromal COX2 expression—are evaluated across WT, WT+Hp, and BMP-KO

conditions, without a systematic comparison including BMP-KO+Hp across these same parameters. As a result, the phenotypic relationships across conditions are not assessed in a fully comparable manner across studies. The available data suggest a pattern closer to:
WT < WT + Hp < BMP-KO ≈ BMP-KO + Hp, rather than
WT < WT + Hp ≈ BMP-KO ≈ BMP-KO + Hp.
This pattern implies that BMP deficiency induces a broader perturbation of gastric epithelial homeostasis than infection alone, rather than faithfully recapitulating the infection-induced state.
Despite this, the current manuscript repeatedly uses language implying equivalence between the two conditions. For example, the Results section states that Bmpr1a deletion "mimics" or "recapitulates" epithelial responses observed during H. pylori infection. These statements appear stronger than what the current data support.
It would therefore be important for the authors to more carefully distinguish between (partial) phenotypic overlap and true mechanistic equivalence between BMP suppression and infection-induced signaling, and to revise statements implying that Bmpr1a deletion directly mimics Helicobacter infection.

We thank the reviewer for this important point. We agree that the term "mimics" may overstate the relationship between BMP deficiency and *H. pylori* infection. Accordingly, we have revised the manuscript and removed statements indicating that *Bmpr1a* deletion "mimics" or "recapitulates" the epithelial responses observed during *H. pylori* infection.

Our intention was not to imply that BMP deficiency and *H. pylori* infection are equivalent. Rather, we use the *Bmpr1a* KO model to investigate the specific consequences of BMP loss in the gastric epithelium. While *H. pylori* infection induces a broad range of biological responses, some of which are likely independent of BMP signaling, the *Bmpr1a* KO model allows us to dissect the contribution of BMP suppression to epithelial reprogramming, inflammation, and tissue pathology. The manuscript has been revised accordingly.

Major Comment 1-2 – Interpretation of BMP signaling in the organoid PAMP experiments
A related issue concerns the interpretation of the organoid experiments presented in Fig. 3. In this system, epithelial cells are cultured in Noggin-containing medium, which functionally suppresses BMP signaling and therefore approximates a BMP-low state. Under these conditions, stimulation with bacterial PAMPs robustly activates NF-κB signaling and chemokine expression, whereas addition of BMP2 suppresses these responses. However, the experiments presented here primarily demonstrate that BMP stimulation suppresses PAMP-induced NF-κB activation. They do not directly establish that loss of BMP signaling is sufficient to induce NF-κB activation or to prime epithelial cells for inflammatory responses in organoids. The interpretation that BMP loss actively drives or sensitizes NF-κB signaling in this context therefore appears stronger than what the current data directly support.

We thank the reviewer for this important clarification. We agree that the organoid experiments directly demonstrate that BMP2 suppresses PAMP-induced NF-κB activation

and chemokine expression, but do not establish that loss of BMP signaling is by itself sufficient to induce or sensitize these responses.

Accordingly, we have revised the text throughout the manuscript to more accurately reflect the experimental findings. Specifically, we now state that BMP2 suppresses epithelial responses to PAMP stimulation in organoids and have removed statements implying that BMP loss actively drives or primes NF-κB signaling in this system.

Furthermore, in their response to my previous suggestion to examine BMP signaling under Noggin-free conditions, the authors state that they intentionally used full medium containing BMP inhibitors to mimic the stem cell niche and again refer to their 2022 study. While this rationale is understandable, the current experimental design makes it difficult to disentangle the specific contribution of BMP signaling from the combined effects of multiple niche factors present in the culture system.
If BMP suppression were truly a major determinant of infection-induced inflammatory signaling, one might expect BMP-deficient tissue in vivo to exhibit enhanced inflammatory responses upon Helicobacter infection. However, the in vivo data presented in the authors' previous work do not clearly demonstrate such an interaction.
To clarify these issues, it would be appreciated to more directly evaluate the relationship between BMP signaling and infection-induced inflammatory responses in vivo. Direct comparison of key components of the proposed signaling cascade—including epithelial NF-κB activation, IL-1–related signaling, stromal COX2/PGE2 activation, and epithelial YAP activation—across WT, WT+Hp, BMP-KO, and BMP-KO+Hp conditions would help determine whether BMP suppression truly sensitizes epithelial tissue to microbial inflammatory signaling.

We thank the reviewer for this suggestion. To address this point, we compared uninfected and *H. pylori*-infected *Bmpr1a* KO mice. As shown in the new data below, *H. pylori* infection did not result in a substantial increase in inflammation beyond that already present in the KO mice, indicating that there is no major hyper-additive inflammatory response following infection.

These findings are consistent with our observation that loss of BMP signaling induces inflammation even in the absence of *H. pylori* infection. This suggests that additional stimuli, including microbial products, PAMPs, or DAMPs that gain access to the gastric gland pits, may contribute to the inflammatory phenotype observed in BMP-deficient mice.

Importantly, BMP depletion also exerts antimicrobial effects that limit *H. pylori* colonization. As a result, comparisons between infected WT and infected KO mice are complicated by substantial differences in bacterial burden and the resulting inflammatory milieu. Consequently, the inflammatory responses observed in WT mice following *H. pylori* infection and those observed in KO mice occur in distinct biological contexts and cannot be directly compared.

For these reasons, although a comprehensive analysis of epithelial NF-κB activation, IL-1 signaling, stromal COX2/PGE2 activation, and epithelial YAP activity across all four experimental groups would be of interest, we believe that such experiments would be difficult to interpret and are unlikely to provide substantial additional mechanistic insight beyond the conclusions already supported by the current data.

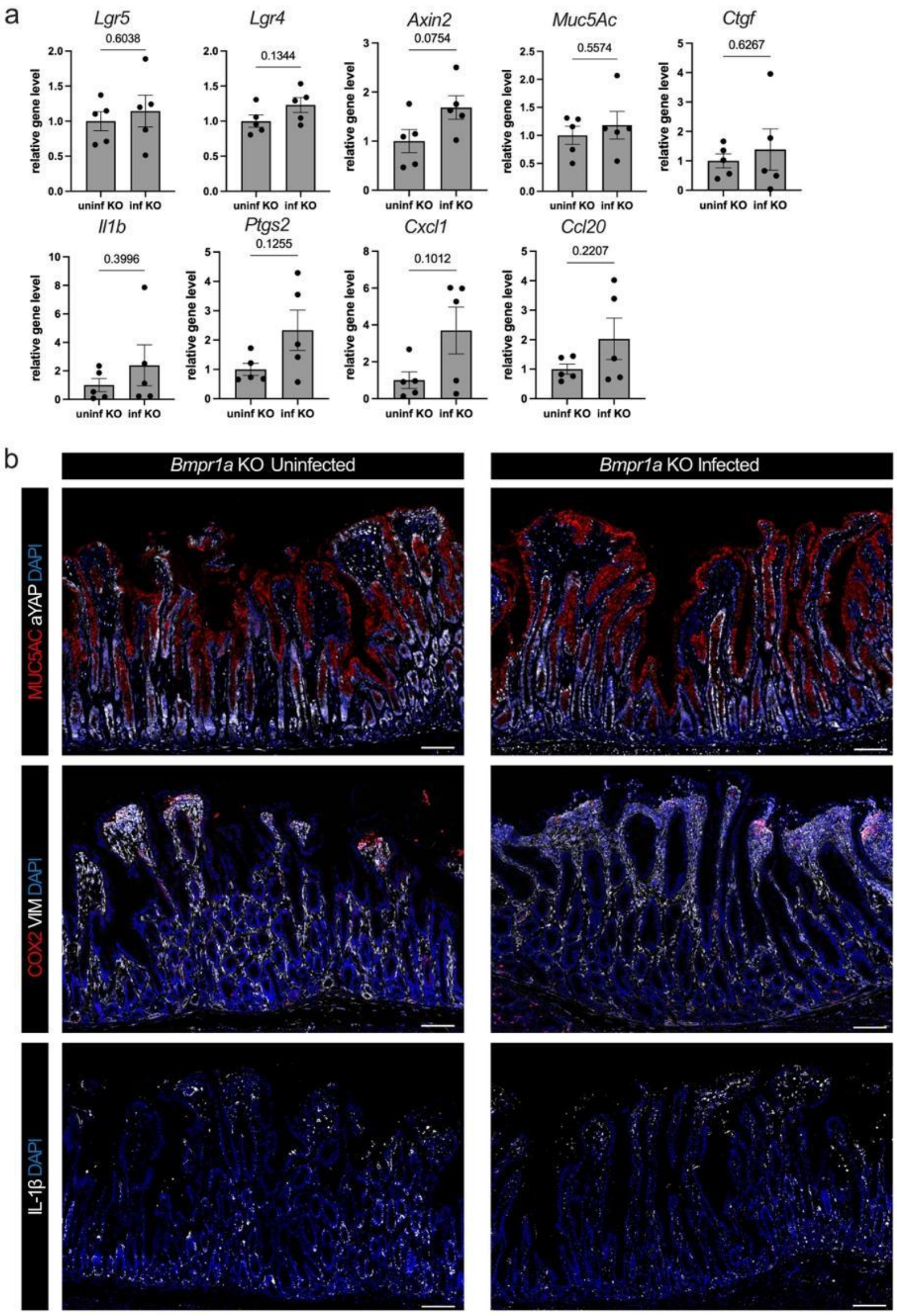

Major Comment 2 – Insufficient presentation, integration, and methodological description of the single-cell RNA-seq analyses

In my previous review, I suggested that single-cell or spatially resolved analyses would be required to clarify the proposed epithelial–immune–stromal signaling cascade. In the response letter, the authors state that several scRNA-seq analyses have been incorporated in the revised manuscript. While these additions are appreciated, the current presentation remains difficult to interpret and does not fully address the concerns raised previously.

2-1 Dataset provenance and sample composition remain unclear

First, the provenance and composition of the “newly generated” murine scRNA-seq datasets are not clearly described. The Methods section describes epithelial cell isolation and

sequencing of EPCAM-positive cells, but it is not clearly specified which genotypes or infection conditions are represented in these datasets. Based on the figure labeling, the epithelial datasets appear to include conditions such as WT, BMP-KO, and Hp-infected samples (with the uninfected control possibly corresponding to WT), but this is not explicitly stated. Given that two biological replicates were analyzed per condition, this would correspond to approximately six to eight individual samples, yet the manuscript does not clarify whether these samples were processed and sequenced separately, multiplexed within the same run, or processed across different sequencing batches. Because no description of pooling, hashtag-based multiplexing, or sample indexing strategies is provided, it is difficult to determine how the individual samples were handled experimentally and how batch structure may relate to the downstream analyses.

We have now described the details of our murine epithelial scRNA-seq datasets as requested:

(ll. 614-617) "For murine scRNA-seq analysis, we performed epithelial cell isolation from *Bmpr1a* WT and KO mice (uninfected *Bmpr1a*$^{fl/fl}$ and *Axin2*^{CreErt2}*Bmpr1a*$^{fl/fl}$) and from 2-month *H.pylori* uninfected and infected WT mice, following the method described in the section "Organoid culture". Two mice for each condition (eight mice in total) were used."

(ll. 623-624) "Single cells from eight mice were sequenced together without pooling in one multiplexed run using 10x GEM-X Flex fixed RNA profiling…"

2-2 Cross-dataset inference and lack of a unified analytical framework
Second, the manuscript draws on several heterogeneous datasets, including newly generated murine epithelial datasets, previously published murine stromal datasets, and human scRNA-seq atlas data. These datasets differ substantially in species, tissue context, and infection status. While each dataset may be appropriate for addressing specific questions, the current presentation gives the impression that different datasets are selectively used to support individual components of the proposed signaling cascade, rather than providing a unified and internally consistent analysis across conditions.
It would therefore be important to clarify how each dataset contributes to the overall model, and to more explicitly define which conclusions are supported by direct comparisons within the same dataset versus those inferred across different datasets. As a result, it remains unclear whether the proposed signaling cascade is supported within a unified experimental framework or inferred from observations distributed across independent datasets.

We have now systematically analyzed IL-1 signaling molecules IL-1β and IL1R1, as well as PGE2 biosynthetic pathway molecules PTGS2 and PTGES in the stromal/immune compartment using both murine and human datasets.

(ll. 367-374) "To determine which stromal subsets that can produce PGE2, we analyzed a published scRNA-seq dataset of murine gastric stroma (Supplementary Fig. 8a-b, Fig. 4n), revealing that *Ptgs2* and *Ptges* were mainly expressed in lamina propria fibroblasts (FB *Adamdec1 Nrg1*) and pit fibroblasts (FB *Pdgfra Nrg1* and FB *Pdgfra Sox6*), and those subpopulations also expressed high levels of the IL-1β receptor *Il1r1* (Fig. 4n). Consistently, a

published human stomach atlas confirmed that fibroblasts highly express *IL1R1*, while human gastric fibroblasts expressed PGE2 biosynthetic pathway molecules *PTGS2* and almost exclusively *PTGES* (Supplementary Fig. 8c-e).”

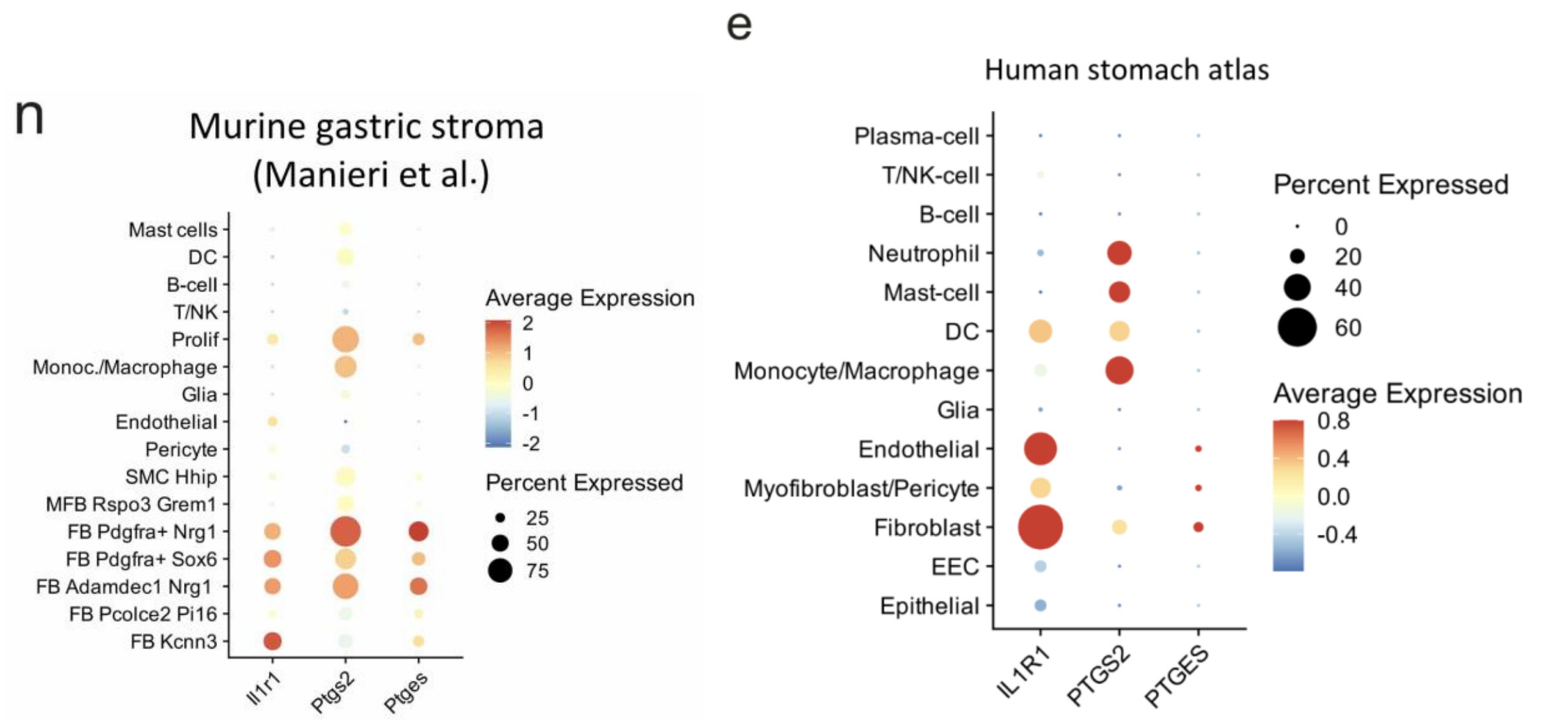

(ll. 375-378) “To characterize which cell populations express IL-1β in the gastric mucosa, we further investigated both murine and human stomach scRNA-seq data. These analyses revealed that IL-1β-expressing cells are mainly myeloid cells, including neutrophils, monocytes/macrophages, and dendritic cells (DCs) (Supplementary Fig. 9a-b).”

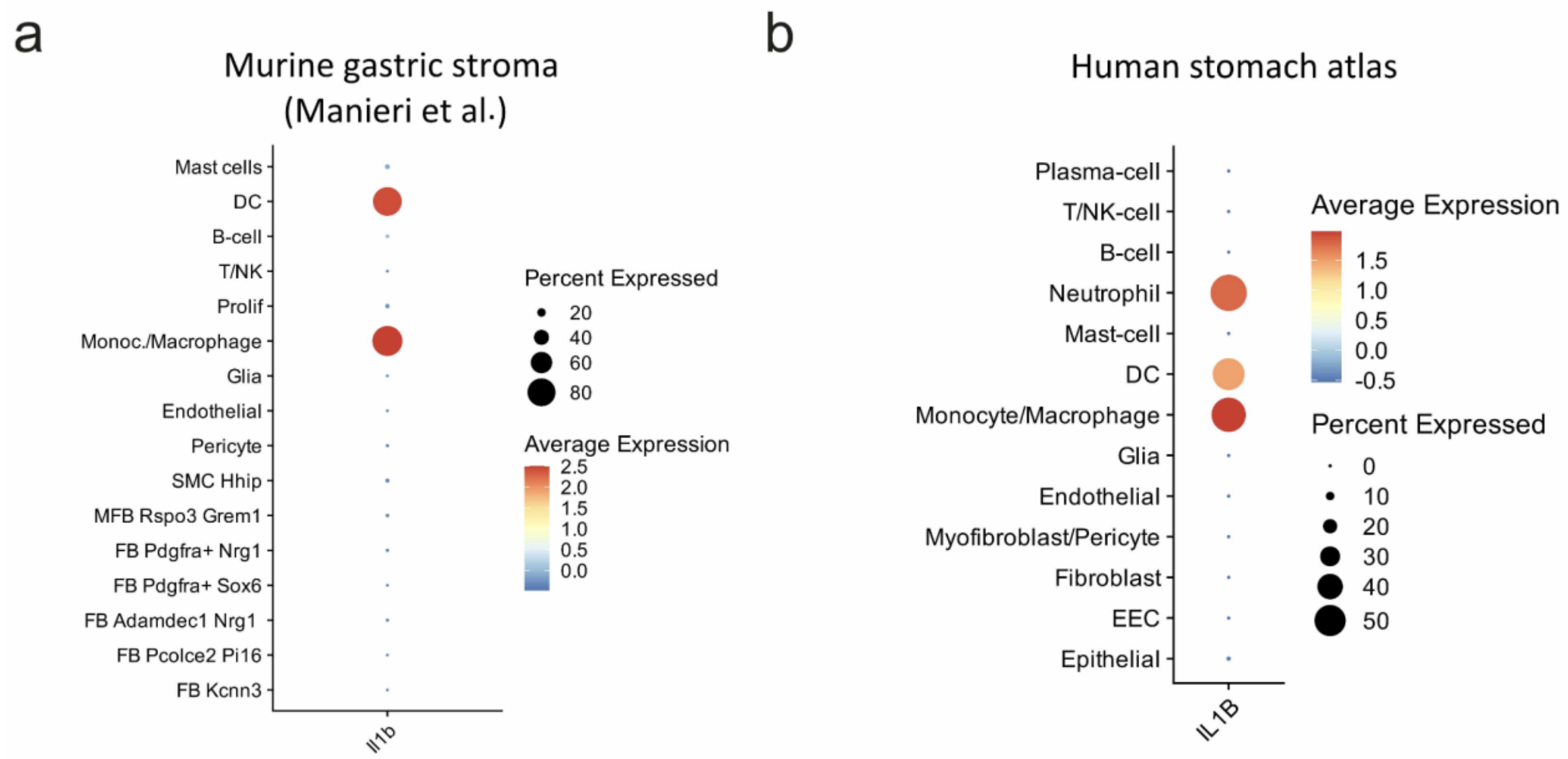

2-3 Lack of transparency in data presentation and validation

Third, and perhaps most importantly, key information required to evaluate the datasets is not provided. The manuscript does not show UMAP visualizations labeled by sample or condition, does not report cell numbers per cluster or per sample, and does not provide clear validation of cluster annotations using canonical markers. In addition, although the analysis appears to involve lineage-specific subclustering (e.g., epithelial and stromal subsets), the manuscript does not present a clear overview of the global dataset before these subset analyses. Providing an initial global view of the integrated dataset, followed by the corresponding subclustering analyses, would make the analytical workflow substantially

easier to interpret and evaluate. These details are critical for assessing the robustness and reproducibility of the single-cell analyses.

We have now provided UMAP and dot plots to show cell subpopulations and their canonical marker expression for our murine gastric epithelium dataset, as well as published murine gastric stroma and human gastric tissue datasets (unfortunately, we were not able to analyze the stroma in our dataset using ScRNA-seq due to a limited cell number, which had technical reasons). We have also provided a bar plot showing cell subset proportions and a table showing cell counts of each subset for our dataset. Additionally, we have now shown the global UMAP for the whole dataset as well as the separated UMAP for each experimental group.

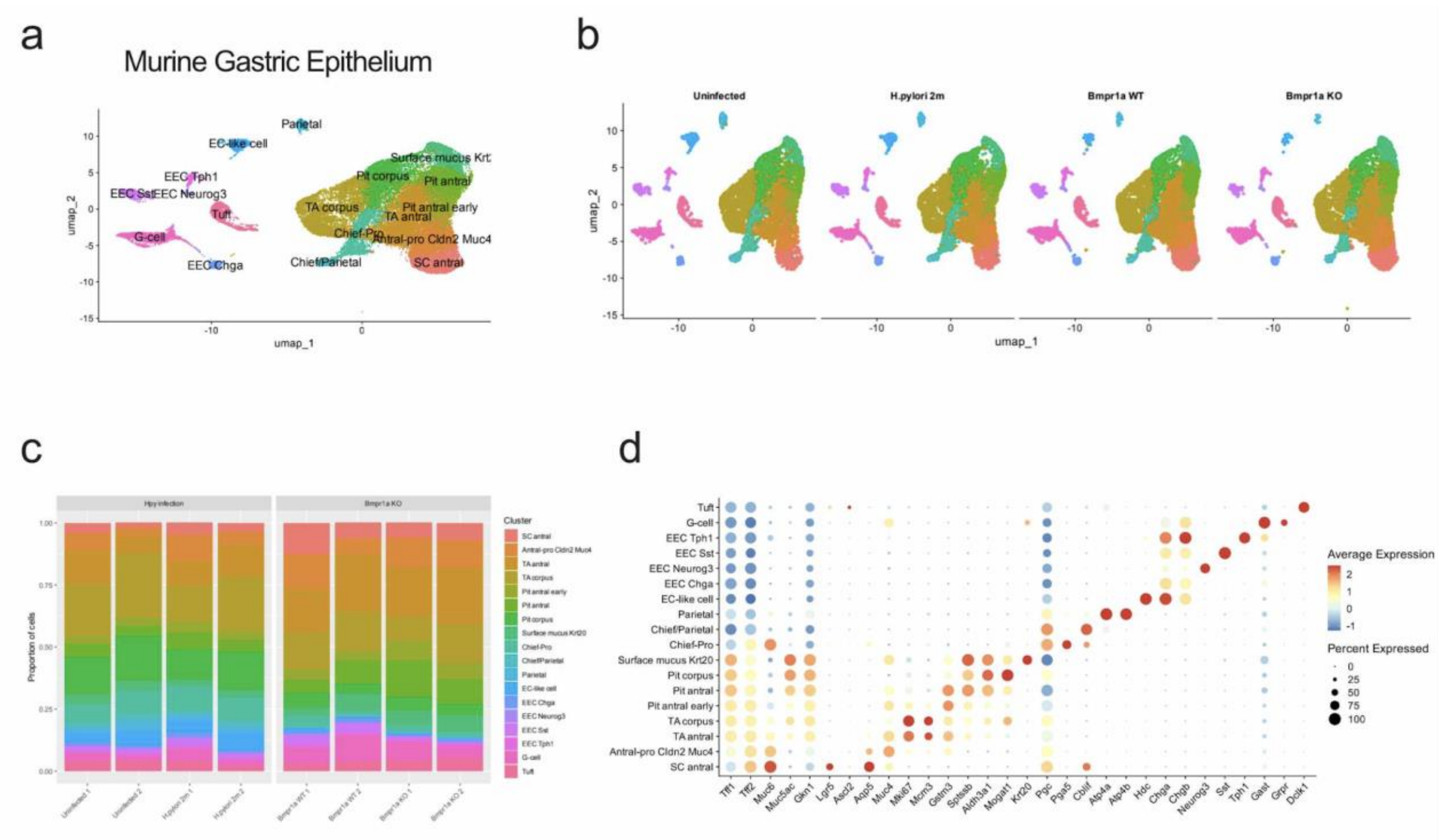

SupplFigure8

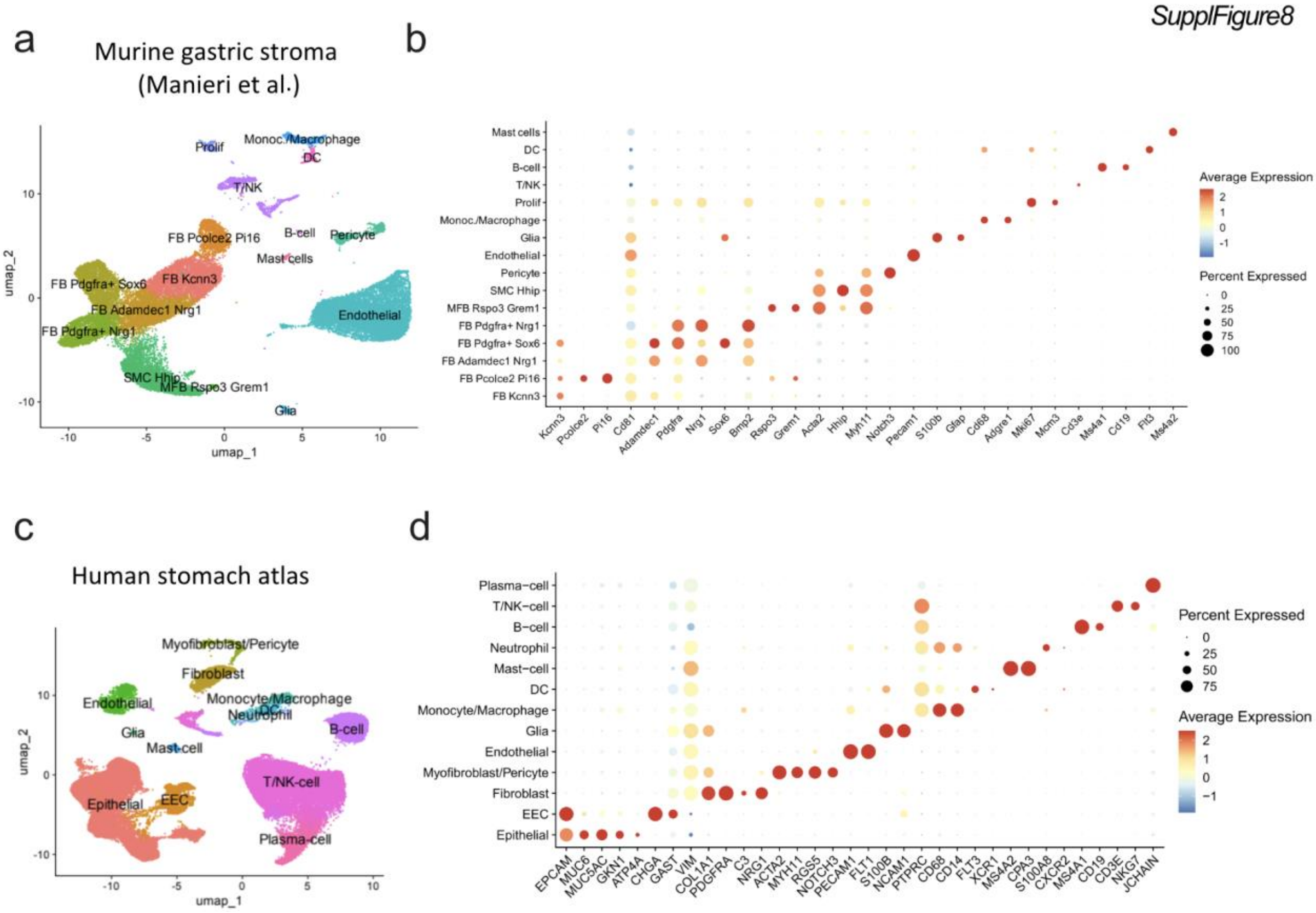

Supplementary Table 1 Cell counts of epithelial subsets from the murine gastric epithelium scRNA-seq dataset.

Cluster	Uninfected	Infected	*Bmpr1a* WT	*Bmpr1a* KO
Tuft	308	279	363	506
G-cell	210	357	922	644
EEC Tph1	53	86	164	154
EEC Sst	153	245	404	78
EEC Neurog3	22	51	67	20
EEC Chga	81	103	148	108
EC-like cell	344	559	80	32
Parietal	194	186	30	17
Chief/Parietal	149	177	38	37
Chief-Pro	613	896	394	265
Surface mucus Krt20	307	245	547	681
Pit corpus	1193	1257	573	534
Pit antral	354	524	792	1362
Pit antral early	239	361	443	760
TA corpus	1685	1627	1736	1362
TA antral	829	1003	2244	2256
Antral-pro Cldn2 Muc4	427	764	1191	1243
SC antral	259	384	1101	701
Total	7420	9104	11237	10760

2-4 Limitations of data representation using averaged expression

Finally, the analyses rely largely on heatmaps of averaged expression values. Such representations do not allow assessment of the fraction of expressing cells, the magnitude of expression differences, or statistical significance of differential expression.

Overall, a more transparent, reproducible, and systematic presentation of the scRNA-seq analyses—including clear dataset descriptions and direct comparisons between infection and BMP-deficient conditions—would substantially strengthen the study. The authors should perform direct single-cell–level comparisons of transcriptional programs between these conditions and present a systematic comparison of transcriptional similarities or differences between BMP signaling loss and infection-induced responses, rather than relying on favorable snapshots from bulk/single-cell RNA-seq analyses.

We have now additionally used UCell gene signature scores to show fetal-like regenerative stem cell and inflammatory response activity across all epithelial subsets under the conditions of infection and *Bmpr1a* KO.

(ll. 161-165) “Next, we investigated which gastric epithelial cell subpopulations upregulate the regenerative stem cell signature, both upon *H. pylori* infection and *Bmpr1a* KO induction. UCell gene signature scores revealed that the upregulation was most pronounced in antral stem/progenitor/TA cells following infection, and in antral TA and pit cells upon *Bmpr1a* KO (Supplementary Fig. 2e-f).”

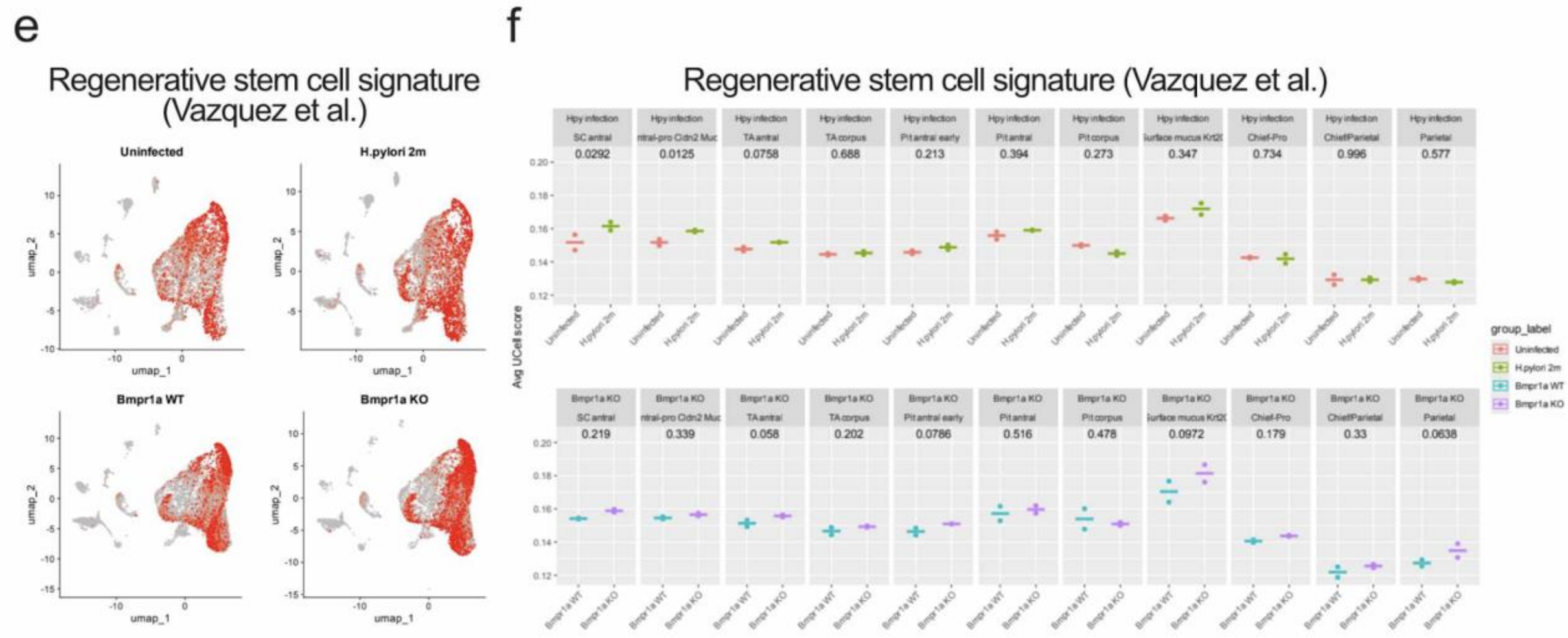

(ll. 217-218) “Consistently, the hallmark of inflammatory response was enriched in progenitor/TA and pit cells for both conditions (Supplementary Fig. 3j-k).”

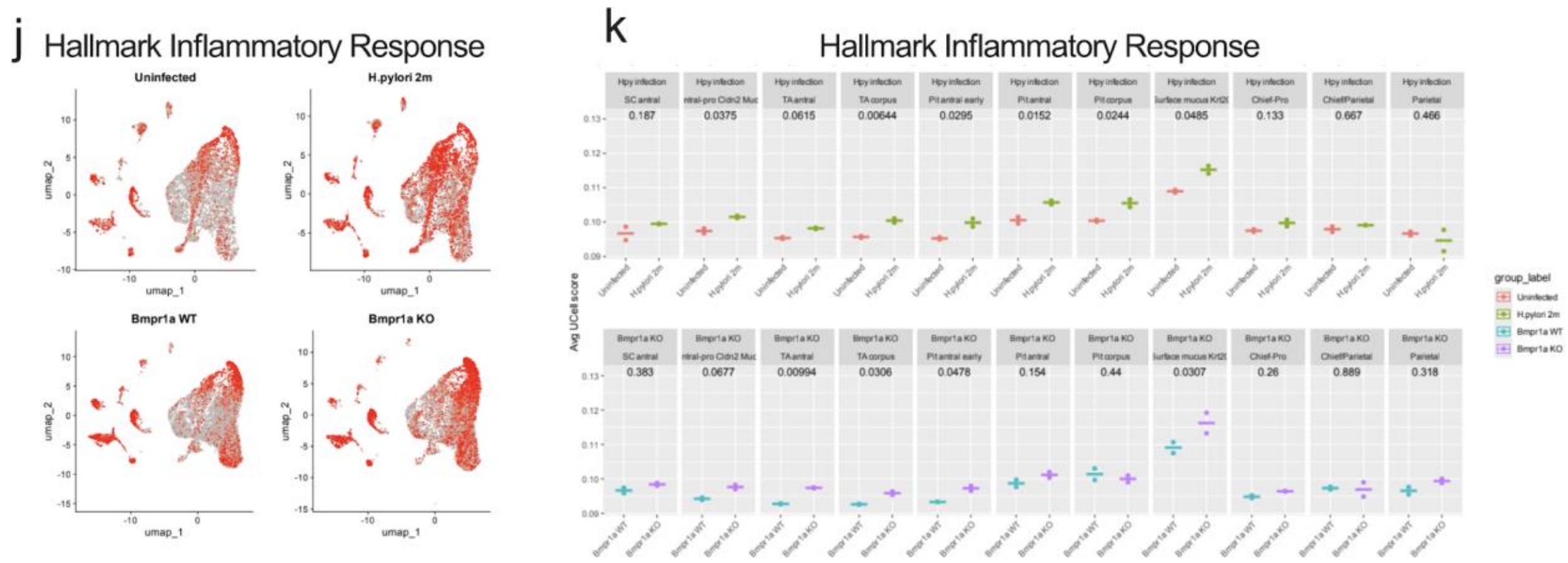

Major Comment 3 – Spatial relationship between stromal COX2 expression and epithelial YAP activation remains unresolved

In my previous review, I noted that the proposed stromal–epithelial signaling axis does not clearly account for the spatial organization of the gastric gland. In the response letter, the authors state that COX2-positive stromal cells are distributed throughout the gland axis. However, the immunostaining data shown in the manuscript appear to indicate that COX2-positive stromal cells are enriched in the pit region rather than being evenly distributed along the gland. At the same time, epithelial YAP activation is described as occurring primarily in the isthmus region. The spatial relationship between these two signals therefore remains unclear.

The additional scRNA-seq analyses also do not resolve this issue. The stromal datasets contain heterogeneous stromal populations, but the manuscript does not clearly identify which stromal subsets represent the functional source of COX2/PGE2 signaling. In addition, the samples in the stromal dataset are derived from uninfected WT corpus and antrum tissues, which may not fully represent the conditions or anatomical regions relevant to the authors' proposed model. Similarly, although epithelial expression of the PGE2 receptor is shown, the manuscript does not clearly define which epithelial populations are capable of responding to this signaling, nor does it indicate whether receptor expression is altered under BMP-deficient or Helicobacter-infected conditions.
Consequently, the spatial organization of the proposed IL-1–COX2–PGE2–YAP signaling axis remains insufficiently supported. Additional spatial analyses linking stromal COX2 expression with epithelial YAP activation along the gland axis, including quantitative analysis of cell distribution and co-staining experiments, would help clarify this relationship.

We have now added an additional spatial analysis that demonstrates that COX2-positive cells are indeed in close proximity to aYAP-positive epithelial cells. These responses are not exclusive to specific gland regions, and we observe them throughout the gland. We have also adjusted the text accordingly.

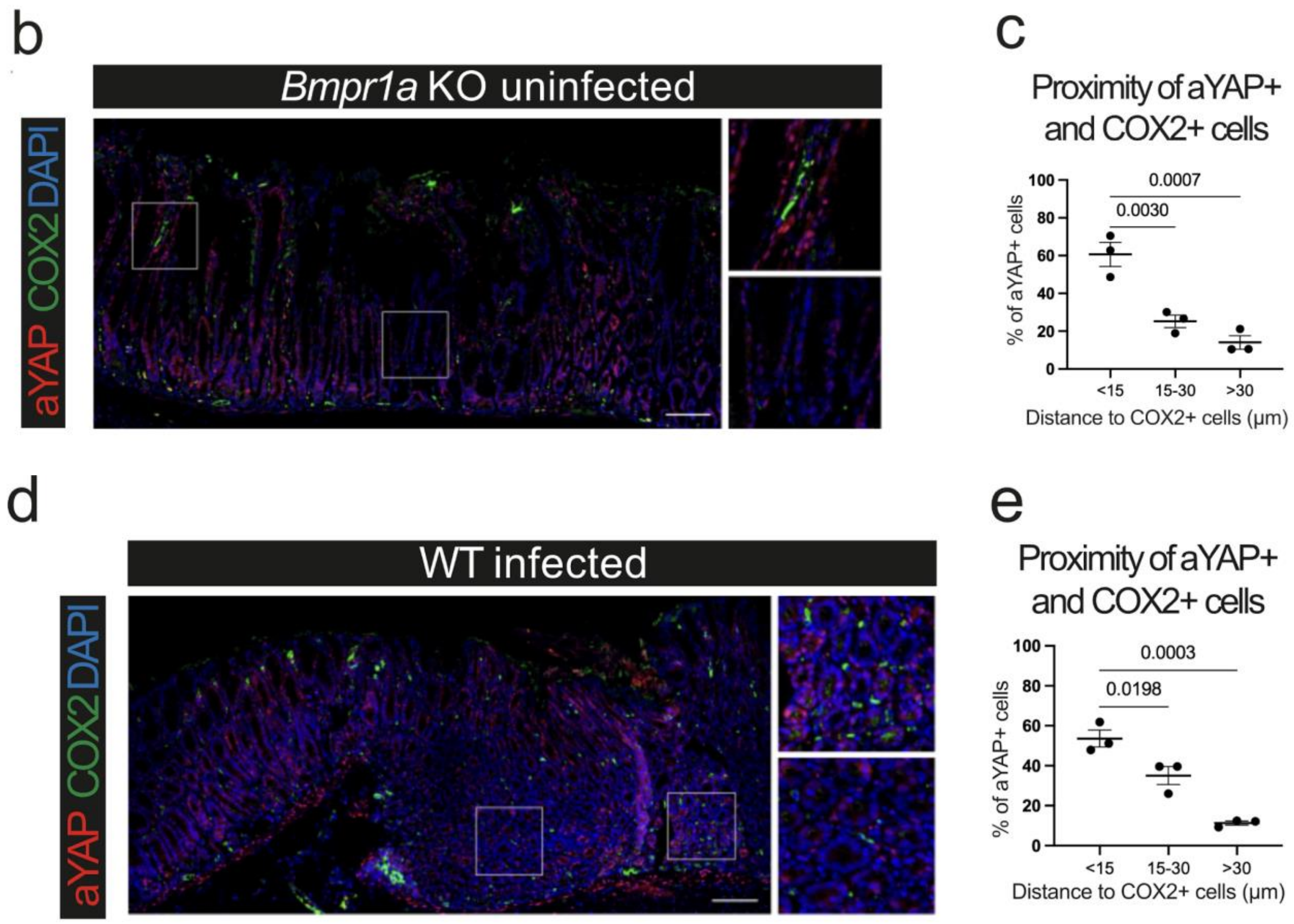

We have now provided both UMAP and dot plots to more clearly show the gene expression of PGE2 receptors in epithelial cell subpopulations. We have also analyzed the alteration of the primary receptor *Ptger4* under the conditions of *Bmpr1a* KO or infection.

(ll. 336-344) "To understand how epithelial cells sense stromal PGE2 signals, we analyzed PGE2 receptor expression using our epithelial scRNA-seq. *Ptger4* was broadly expressed in antral

epithelial cells, including the stem, progenitor, TA, and pit cell lineages (Supplementary Fig. 7j-k). However, *Ptger2* and *Ptger3* were only expressed in a small number of endocrine, parietal, and chief cells, with *Ptger1* showing almost no expression in any epithelial cell type (Supplementary Fig. 7j-k), indicating that *Ptger4* is the primary PGE2 receptor in the antral epithelium. Interestingly, the expression of *Ptger4* was upregulated in the epithelium upon *Bmpr1a* KO (antral stem/progenitor/TA and pit cells) (Supplementary Fig. 7l), suggesting an increased responsiveness to PGE2."

j

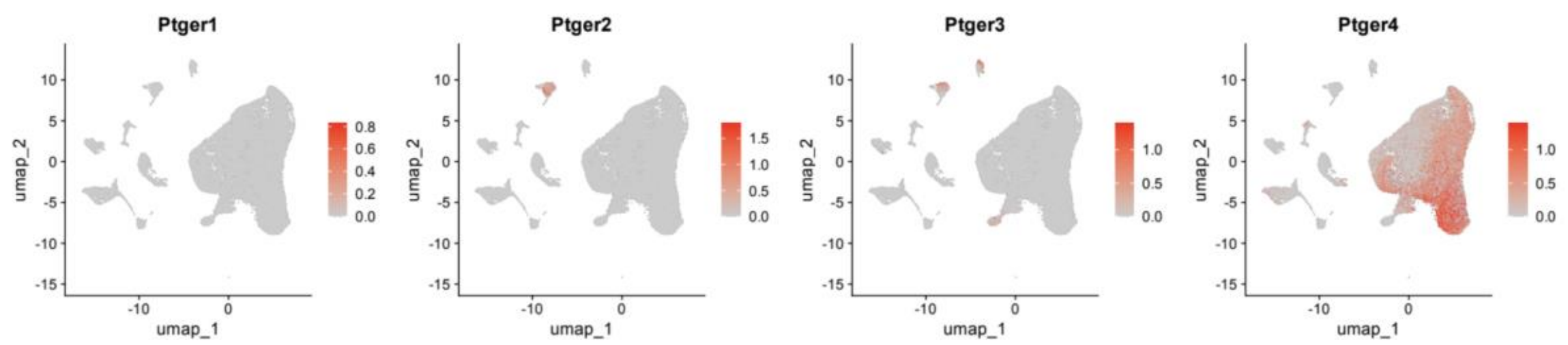

k

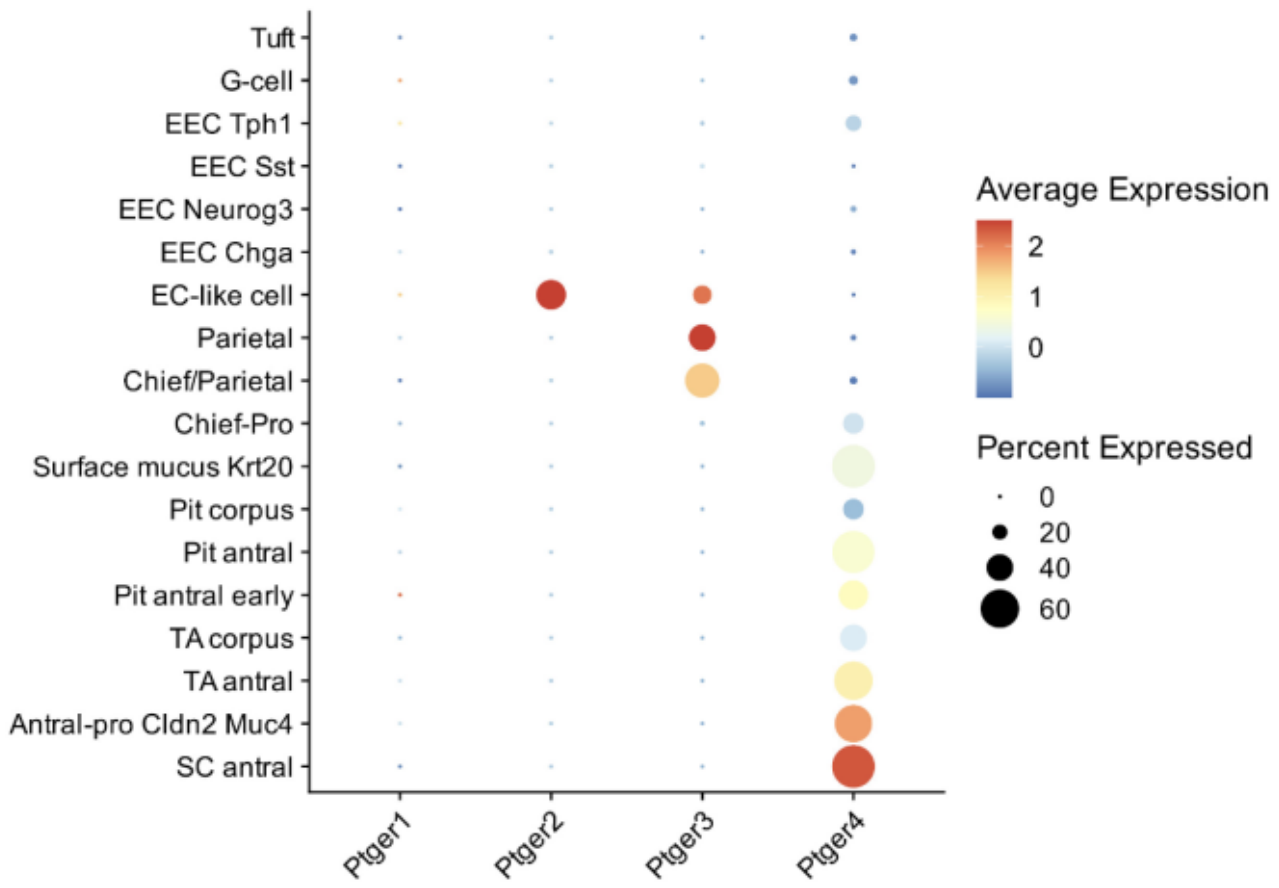

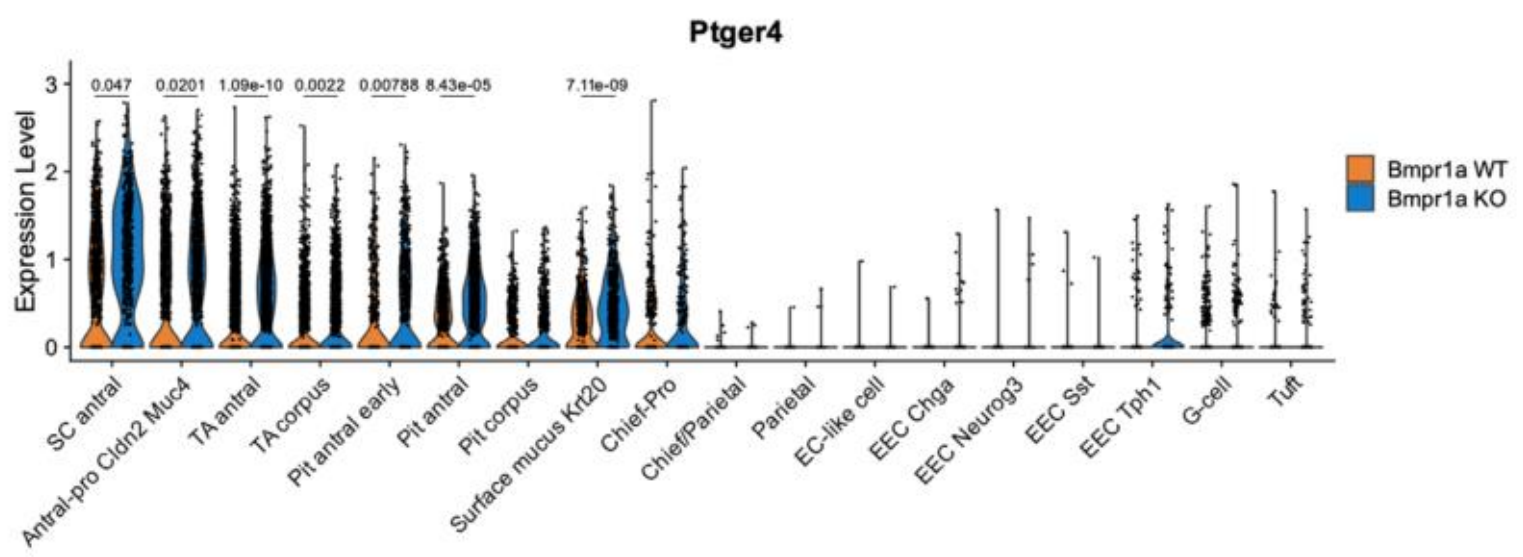

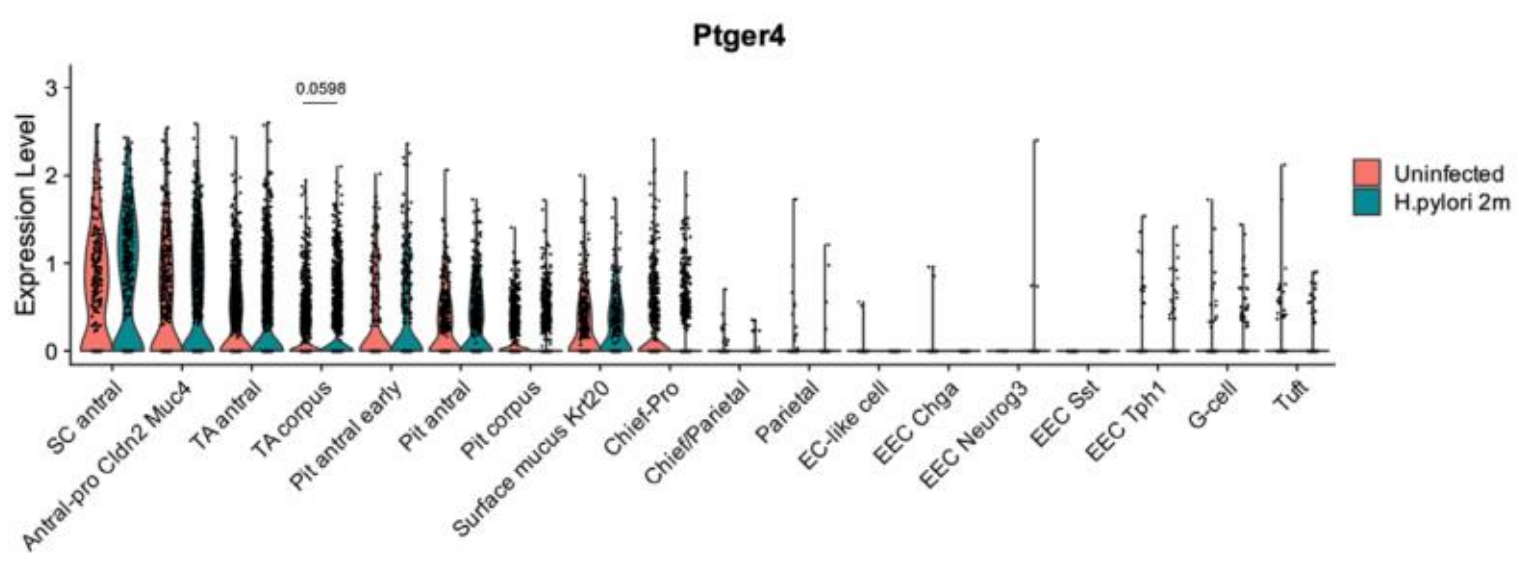

Importantly, the current study does not include functional perturbation experiments testing whether COX2/PGE2 signaling is required for epithelial YAP activation in vivo. Experiments examining the effects of COX2/PGE2 inhibition (e.g., Cox2 inhibitors in vivo or alternatively in assembloids) would therefore provide important support for the proposed stromal–epithelial signaling axis.

We now provide new data using an inhibitor of the PGE2 receptor EP4 in assembloids that reveals a significant reduction of YAP signaling upon IL-1ß stimulation.

(ll. 425-428) "To confirm that IL-1β activates epithelial YAP via stromal COX2-PGE2 pathway, we added PGE2 receptor EP4 (encoded by *Ptger4*) inhibitor L-161,982 to IL-1β-treated assembloids. aYAP staining showed a significant reduction of aYAP+ epithelial cells upon EP4 inhibition (Figure 6i-j)."

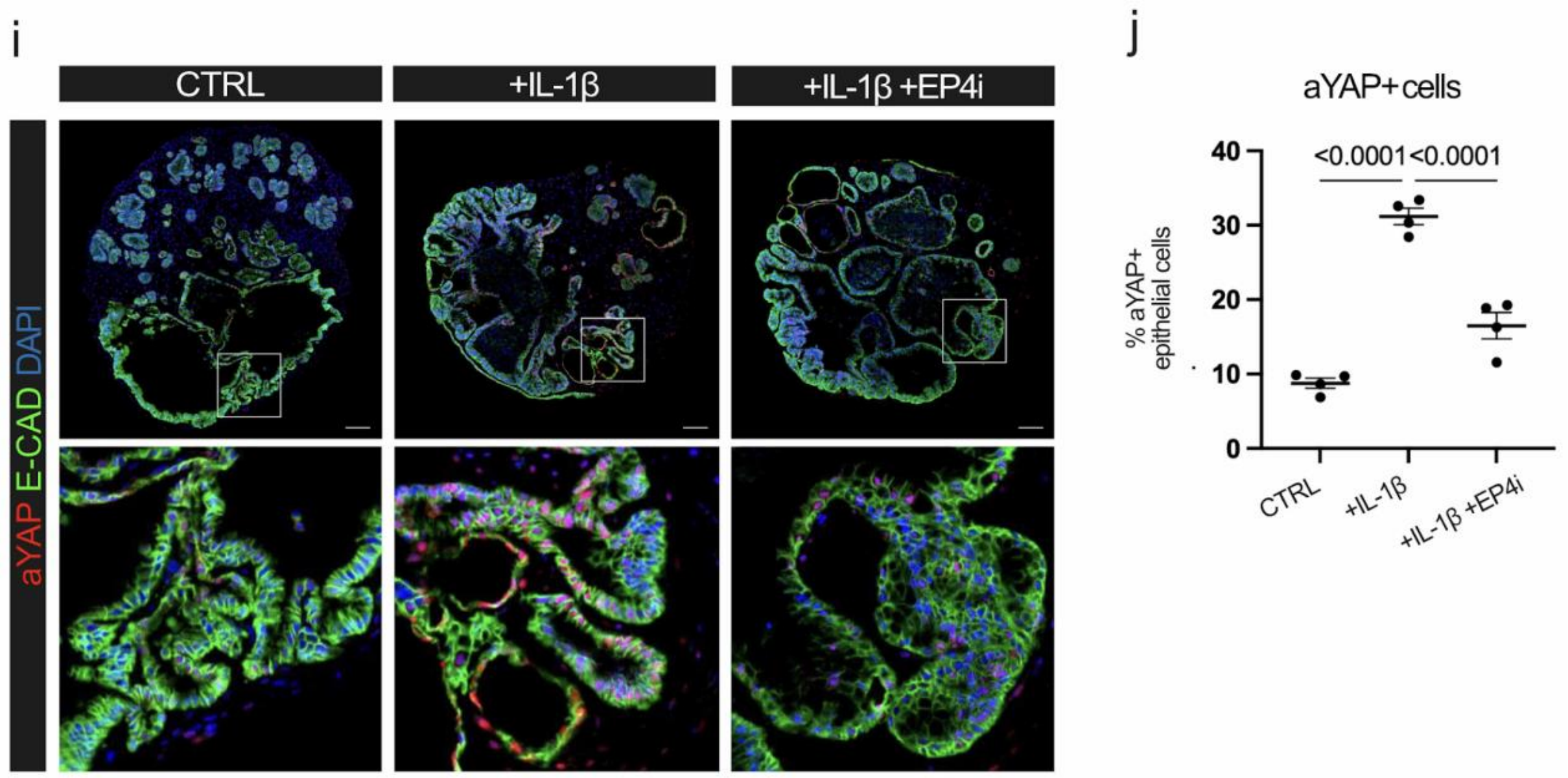

Major Comment 4 – The mechanistic link between BMP signaling and NF-κB/YAP responses remains insufficiently established

In response to the initial review, the authors have added several experiments examining signaling responses in organoids, including Western blot analyses of NF-κB pathway activation, demonstration of BMP-induced SMAD phosphorylation, and short-term BMP2 stimulation experiments. These additions are helpful and clarify certain aspects of BMP signaling within the organoid system.

However, the central mechanistic question raised in the initial review—namely how BMP suppression leads to NF-κB and infammatory signaling and YAP activation in the proposed pathway—remains insufficiently resolved.

First, although the authors demonstrate NF-κB activation and modulation of YAP activity in organoids, the revised experiments do not establish the functional hierarchy linking BMP signaling, NF-κB activation, chemokine expression, and YAP activity. The experiments presented show correlations between these pathways but do not demonstrate whether NF-κB or YAP activity is required for the observed transcriptional responses.

Second, regarding pathway inhibition, the authors indicate that they attempted pharmacological inhibition experiments but were unable to obtain interpretable results.

However, the original comment specifically suggested testing NF-κB and YAP pathway inhibition, whereas the response primarily discusses attempts to inhibit BMP signaling and YAP signaling. As a result, the functional requirement of NF-κB signaling, which is central to the proposed inflammatory pathway, remains experimentally untested.
Finally, the authors state that BMP directly suppresses epithelial YAP activity in organoids (Fig. 3g–h). This observation suggests that epithelial YAP activation could occur directly downstream of BMP signaling loss. However, the central model of the manuscript proposes that epithelial YAP activation is driven by stromal COX2–PGE2 signaling downstream of IL-1β. Therefore, it is not evident whether stromal PGE2 signaling is required for epithelial YAP activation or whether BMP loss alone is sufficient to induce this response, which needs to be clarified.

We agree with the reviewer that additional functional studies would further refine the relationships among the signaling pathways altered by BMP perturbation in vivo. The experiments presented in this study identify a specific epithelial–stromal signaling cascade that links epithelial BMP loss to inflammatory cytokine production, stromal activation, and epithelial proliferation. We have revised the manuscript to clarify that this pathway likely operates in concert with additional cell-intrinsic and cell-extrinsic mechanisms that contribute to the tissue response during *H. pylori* infection.

We also agree that experiments directly testing the hierarchical relationships among BMP, NF-κB, chemokine expression, stromal PGE2 signaling, and YAP activity would be valuable. However, addressing these questions would require extensive additional genetic and/or pharmacological perturbation studies that go beyond the primary scope of the current work, which is to establish the existence and physiological relevance of the epithelial–stromal signaling axis. We therefore believe that these mechanistic questions are more appropriately addressed in future studies. To avoid overstating our conclusions, we have revised the text to clarify the level of mechanistic insight provided by the current data and to explicitly acknowledge these remaining open questions.

Major Comment 5 – Interpretation of epithelial IL-1 signaling remains incomplete
In the original review, I noted that Il1r1 expression appears to be present not only in stromal cells but also in epithelial cells, and therefore suggested that the potential contribution of epithelial IL-1 signaling should be considered.
In the revised manuscript, the authors provide further characterization of stromal Il1r1 knockout mice. To argue against a role for epithelial IL-1 signaling, they claim that IL-1β treatment of epithelial organoids does not activate YAP signaling.
However, the conclusion that IL-1β does not activate YAP signaling in epithelial cells is based on limited analysis. The assessment of YAP activity appears to rely mainly on p-YAP at a single short time point and one IL-1β concentration. Given that the in vivo part of the study uses nuclear active YAP (aYAP) as the relevant readout, a more direct analysis of epithelial YAP activation would be needed, for example by assessing nuclear aYAP localization across multiple time points and IL-1β concentrations.

Indeed, their western blotting data indicate that IL-1 can directly activate NF-κB pathway in organoids (Fig.3g), thus there remains a possibility that IL-1 signaling may also act directly on epithelial cells. If so, the directionality of the proposed signaling cascade (BMP suppression → epithelial NF-κB/chemokine → immune IL-1β → stromal COX2/PGE2 → epithelial YAP) may be more complex than currently depicted, and epithelial IL-1 signaling could represent an additional or parallel regulatory layer.
A more careful analysis of epithelial IL-1 responses in organoids, including YAP activation as well as effects on NF-κB/chemokine pathway, would provide a more robust assessment of whether epithelial IL-1 signaling plays a role here. While additional in vivo models may not be essential for the current study, the current conclusion that epithelial IL-1 signaling is irrelevant appears premature based on the limited analysis presented.

We thank the reviewer for this suggestion and have now performed additional experiments to directly assess epithelial responses to IL-1β. Consistent with our previous findings, IL-1β treatment of epithelial organoids activated NF-κB signaling and induced expression of the NF-κB target gene *Cxcl1*, confirming that epithelial cells are capable of responding directly to IL-1β.

However, despite testing multiple IL-1β concentrations and time points, we did not observe evidence for robust YAP activation. In contrast to PGE2, IL-1β failed to promote organoid growth (Supplementary Fig. 10f-g). Furthermore, qPCR analysis revealed only minimal and inconsistent changes in YAP target gene expression that did not reach statistical significance (Supplementary Fig. 10i). Importantly, immunofluorescence analysis did not demonstrate increased nuclear YAP localization following IL-1β treatment (Supplementary Fig. 10h), whereas clear YAP activation was observed in response to PGE2 (Fig. 4e, Supplementary Fig. 7i).

Together, these data indicate that although epithelial cells can directly respond to IL-1β through NF-κB signaling, IL-1β alone is not sufficient to induce the YAP-dependent regenerative program observed in our model. These findings support our conclusion that stromal responses to IL-1β, including induction of the COX2–PGE2 axis, are the dominant drivers of epithelial YAP activation and tissue remodeling in this setting.

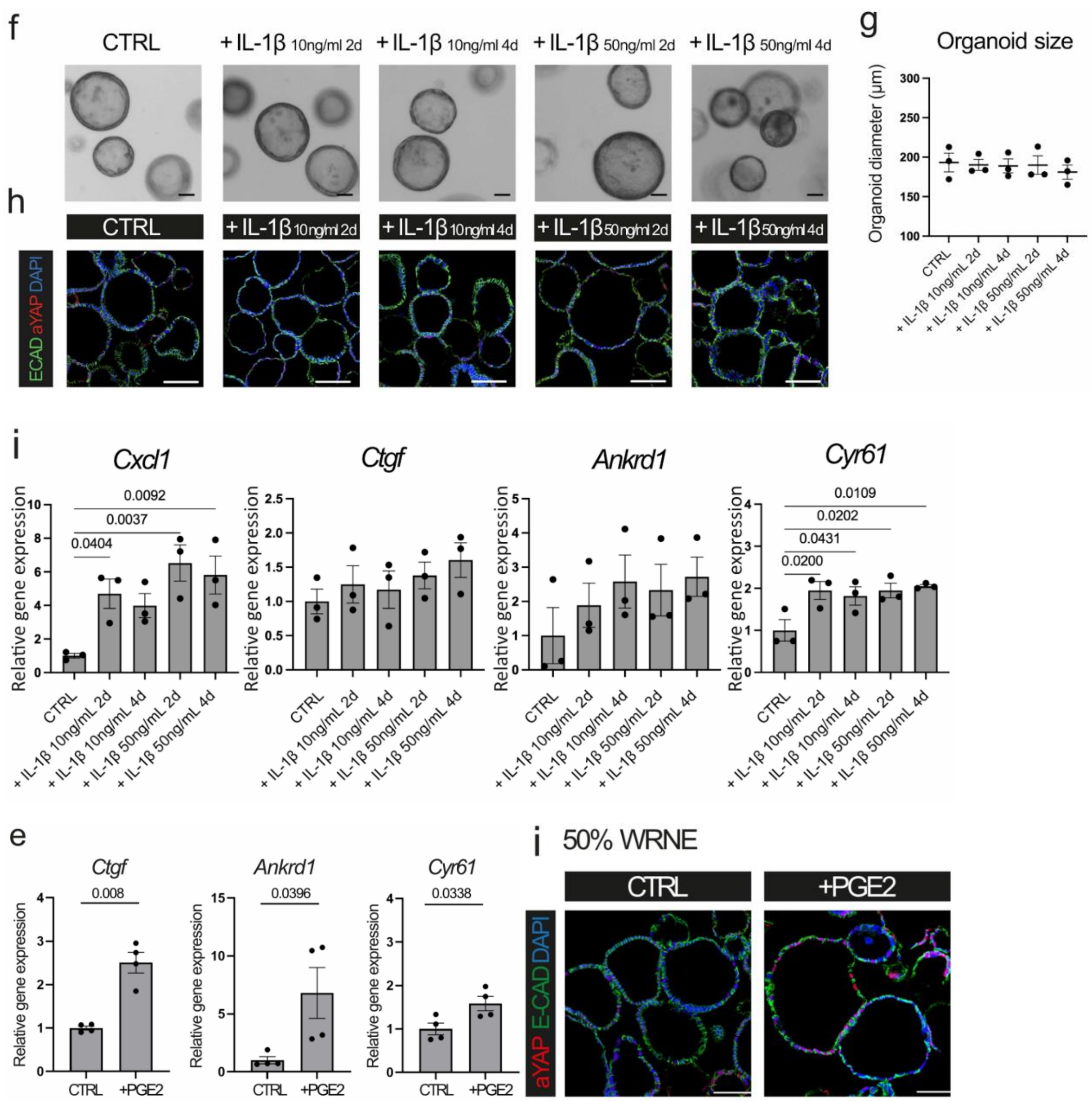

Minor Point 1 – Overstatement and unsupported causal language in the Abstract and main text

Throughout the manuscript, and particularly in the Abstract, several conclusions are stated in a manner that appears stronger than what the current data directly support. In multiple instances, associative or partially supported observations are presented as mechanistically established causal relationships. For example, statements such as “gland hyperplasia is fueled by fetal-like reprogramming,” “epithelial cells that lack BMP signaling are primed to respond to microbes,” “these in turn release IL-1β, promoting stromal remodeling,” and “this epithelial-immune-stromal cascade gives rise to H. pylori-driven gastric pathology” appear to go beyond what is directly demonstrated by the current data. In several of these cases, the manuscript provides evidence partially consistent with the proposed model, but not definitive functional proof of the stated causal hierarchy. I would therefore encourage the authors to carefully revise the Abstract and corresponding sections of the main text to

distinguish more clearly between direct experimental findings and model-based interpretation.

We have now carefully revised the manuscript to distinguish more clearly between direct experimental findings and model-based interpretation.

Minor Point 2 – Definition of "fetal-like reprogramming" and its relationship to BMP–YAP signaling

The concept of "fetal-like reprogramming" represents a central theme of the manuscript, yet its definition and usage throughout the study remain somewhat unclear. Throughout the manuscript, the authors refers interchangeably to a "fetal-like reprogramming" or a "fetal-like state," even in the Title and Abstract. However, based on the data presented, these terms appear to be inferred primarily from changes in the expression of selected fetal-associated genes. It therefore remains unclear whether the study demonstrates a bona fide fetal-like cellular state or rather a fetal-like transcriptional signature. Given the strength of the terminology used, it would be helpful for the authors to clarify how "fetal-like reprogramming" or "fetal-like state" is operationally defined in this study.

In addition, the relationship between fetal-like reprogramming and epithelial YAP activation remains somewhat ambiguous. As shown in the organoid experiments (Fig. 3g–h), BMP stimulation directly suppresses epithelial YAP activity, suggesting that loss of BMP signaling could intrinsically promote YAP activation in epithelial cells. However, the Abstract states that loss of epithelial BMP signaling is sufficient to induce the fetal-like state in vivo but not in organoids, implying that BMP loss alone is not sufficient to induce this program in epithelial cells in isolation. It would therefore be helpful if the authors could clarify how the fetal-like transcriptional changes relate mechanistically to epithelial YAP activation and whether these processes consistently occur in the same epithelial populations across the datasets presented.

We thank the reviewer for this important comment. To address this concern, we have revised the manuscript and now use the term *fetal-like transcriptional signature* when referring to the transcriptional changes observed in our datasets, rather than implying the establishment of a bona fide fetal-like cellular state.

Regarding the relationship between BMP signaling, YAP activation, and the fetal-like transcriptional program, our data indicate that BMP loss alone can directly modulate epithelial YAP activity, as demonstrated in organoids. However, BMP loss in epithelial cells is not sufficient to induce the full fetal-like transcriptional signature in vitro, suggesting that additional microenvironmental signals are required. Consistent with this interpretation, our in vivo data support a model in which epithelial BMP loss initiates a signaling cascade involving stromal activation and PGE2 production that promotes epithelial regeneration and the fetal-like transcriptional signature.

With respect to cell-type specificity, our data indicate that the transcriptional changes, as well as alterations in aYAP and *Anxa1*, which are established markers associated with the fetal-like regenerative response, are not restricted to a single epithelial cell population. A

more detailed dissection of the cellular populations contributing to these responses will be an important objective for future studies.

Minor Point 3 – Clarification of the proposed signaling model
The signaling framework proposed in this manuscript involves multiple interacting cellular compartments and signaling pathways, including epithelial BMP signaling, inflammatory responses, immune-derived IL-1β, stromal COX2/PGE2 signaling, and epithelial YAP activation. For clarity, it would be helpful if the authors could include a schematic diagram summarizing the proposed signaling model and the directionality of the interactions between epithelial, immune, and stromal compartments.

We fully agree that a schematic diagram is helpful. We have now provided a scheme summarizing our signaling model and the proposed epithelial-immune-stromal interaction (Supplementary Fig. 10j).

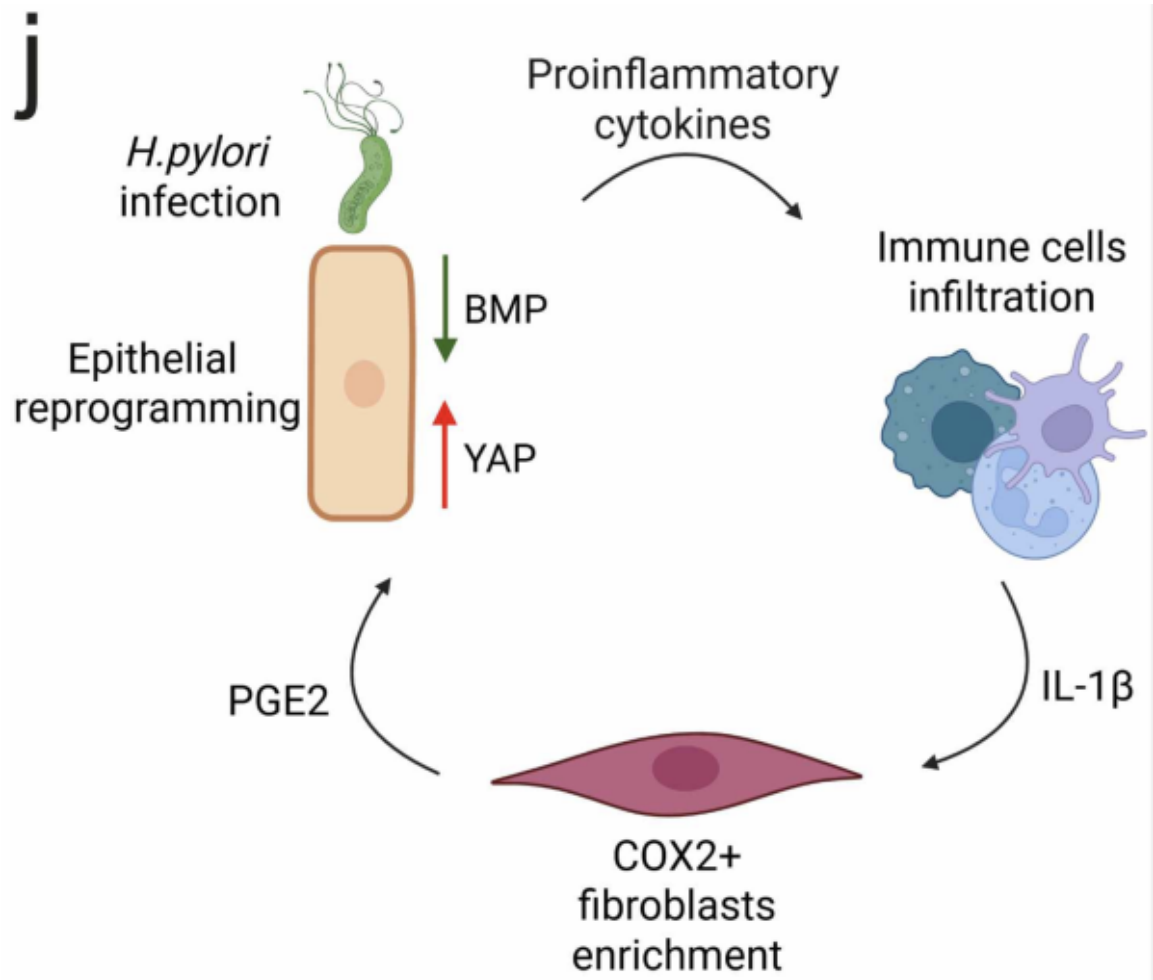

Finally, because several major conceptual and mechanistic issues remain to be addressed, I have focused the comments above on the principal points that are most critical for interpreting the study. Additional minor issues and questions regarding specific data presentation are present but are not listed here at this stage.

We thank the reviewer for their critical and constructive suggestions. In addition to addressing the issues raised above, we have thoroughly revised the entire manuscript to ensure clarity and accuracy.

We appreciate Reviewer 3’s positive evaluation. We have now further addressed the remaining concerns and revised the manuscript based on their suggestions.

Reviewer #3 (Remarks to the Author):

The revised manuscript is substantially improved compared with the previous version. I appreciate the authors’ careful and constructive efforts to address my prior concerns. In particular, the authors have added new experiments and analyses, clarified several aspects of the proposed epithelial–immune–stromal pathway, and appropriately toned down a number of statements that previously implied direct equivalence between Bmpr1a deletion and H. pylori infection. Overall, many of my previous mechanistic concerns have now been adequately addressed or appropriately revised.
However, I still have concerns regarding the analysis and presentation of the newly generated epithelial scRNA-seq dataset. This dataset has the potential to substantially strengthen the manuscript, but the current presentation does not yet fully clarify how the single-cell data support the histological phenotypes and the proposed model.

1. The authors now clarify that epithelial cells from eight mice were processed together in a single multiplexed 10x GEM-X Flex run. However, the Methods also state that sample integration was performed using Harmony to correct for sample effects. Given that the samples were processed in a single multiplexed run, the rationale for Harmony-based integration should be more clearly explained, particularly because integration may reduce condition-dependent biological differences. This point is relevant because Bmpr1a KO tissue shows marked gland hyperplasia histologically, whereas the integrated UMAP does not clearly show a distinct KO-associated epithelial state. I do not necessarily think that extensive additional computational analyses are required here, but the authors should explain whether the main conclusions, particularly the condition-dependent changes in epithelial cell populations, are preserved after integration.

Although we processed all samples in a single multiplexed 10x GEM-X Flex run, we performed integration as a necessary step for embedding to remove technical artifacts and sample-specific biological effects in order to identify comparable cell subpopulations. Gene expression differences within subpopulations are preserved and inform on biologically relevant regulation. Indeed, we observed no alteration in the proportions of cell subsets in *Bmpr1a* KO mice. This suggests that BMP deficiency leads to the expansion of multiple cell lineages with no significant change in their relative proportions. Nonetheless, we found profound transcriptional changes in most antral epithelial cell types following *Bmpr1a* KO or *H. pylori* infection, including the enrichment of regenerative stem cell and inflammatory response signatures.
For comparison, we also generated UMAP embeddings and clusters using the non-integrated data (see below). While this yielded largely similar clusters, cells from distinct lineages in the infected samples were aberrantly aggregated into a new cluster (Epi inflamed) driven by the high expression of inflammatory genes. Importantly, an enriched inflammatory response was also observed in infected epithelial cell subsets after integration (see Supplementary Fig. 4j). Furthermore, consistent with our integrated analysis, we observed no significant alterations in the relative proportions of cell populations in *Bmpr1a*

KO mice.

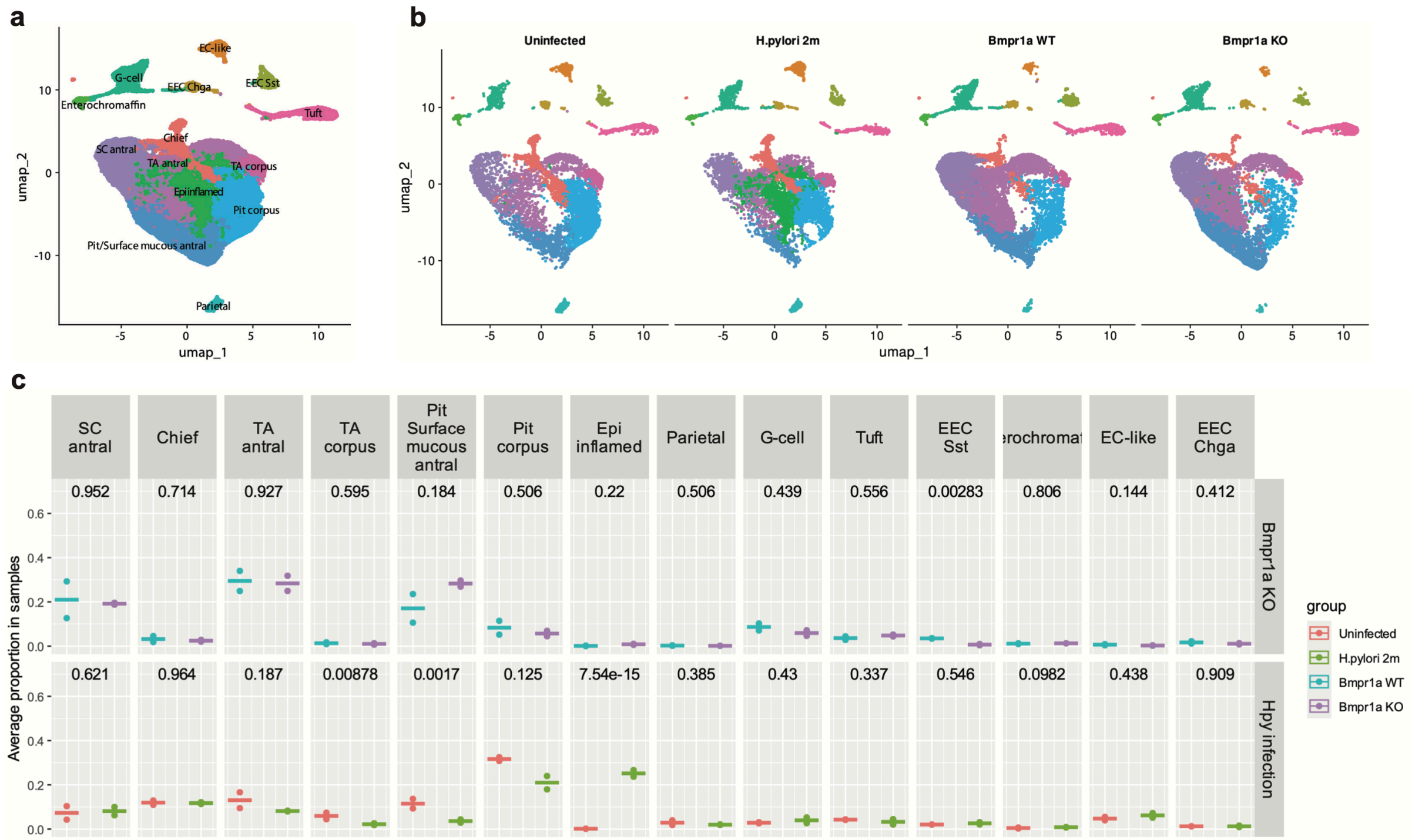

2. The revised manuscript includes epithelial cluster annotations and marker dot plots, which improve the presentation. However, the basis for the relatively fine-grained epithelial subcluster annotation remains insufficiently explained. The authors define 18 epithelial subsets, including multiple stem, TA, and progenitor cells. At the same time, many corpus-associated cell types are also present in the dataset. Because most downstream analyses depend on clustering and annotation, the authors should more carefully explain and validate how these epithelial populations were defined. Specifically, the authors should provide:

1)A table listing each epithelial cluster, the key marker genes used for annotation, the assigned cell identity, the expected anatomical location along the gastric gland axis, and the corresponding published gastric epithelial cell type when available.

We have reclustered the full data to reduce the number of epithelial clusters (Supplementary Fig. 2a) and have now provided a table clearly showing relevant information for each of them (Supplementary Table 1). We have finally restricted all further analyses to the antral epithelial subpopulations only (Supplementary Fig. 2b).

2)A clear explanation of how antral and corpus epithelial cells were distinguished during tissue collection and during computational annotation.

During tissue collection, we identified the borders of the corpus, antrum, and pylorus based on the color and structure of the gastric tissue. This approach is consistent with previous studies (DOI: 10.1002/aja.1001640302).

For scRNA-seq experiments, we isolated the antrum and the transitional zone between the antrum and the corpus from uninfected *Bmpr1a*$^{fl/fl}$ and *Axin2*^{CreErt2}*Bmpr1a*$^{fl/fl}$ mice, since *Axin2* is not expressed in the corpus. However, we collected the whole stomach from 2-month *H.pylori* uninfected and infected WT mice. During computational annotation, we used the expression of *Pdx1*, a gene specifically expressed in the stomach antrum but not the

corpus (DOI: 10.1210/endo.140.11.7122), and identified clusters with a higher mean normalized expression (mean > 0.2) as being of antral origin.
We have now added this information and the related references to the Methods section.

3)Reanalysis focused on the relevant antral epithelial compartment after excluding corpus-associated cells and rare specialized epithelial populations such as chief, parietal, tuft, and endocrine cells.
We have now updated all analyses related to the mouse gastric epithelial cells, focusing specifically on the major antral epithelial cell types (Fig. 3b, Supplementary Figs. 2-3, 4h-j, 8j-l).

4)The authors should quantitatively assess the proportions of the relevant epithelial populations at the biological sample level after refined annotation. In Supplementary Fig. 2c, the authors show the proportions of each epithelial subpopulation in individual samples. However, it remains unclear whether the populations that the authors consider to represent or drive the hyperplastic response are reproducibly increased in Bmpr1a KO and/or H. pylori-infected samples. The authors should present the proportions of the relevant epithelial populations in each group and indicate which population(s) correspond to the expanded hyperplastic/proliferative compartment observed histologically.
We have now quantitatively assessed the proportion of antral cell populations and observed an expansion of TA/progenitor cells and a reduction of pit cells upon *H. pylori* infection, whereas no significant alteration in the proportion of cell subsets was detected in *Bmpr1a* KO mice (Supplementary Fig. 2e). This suggests BMP deficiency leads to the expansion of multiple cell lineages without significantly altering their relative proportions. Furthermore, our GSEA data (see Supplementary Figs. 3b-d) indicate that the regenerative responses, including regenerative stem cell expansion and YAP signaling activation, are not confined to a single cell type but are rather broad responses involving various gland cell lineages.

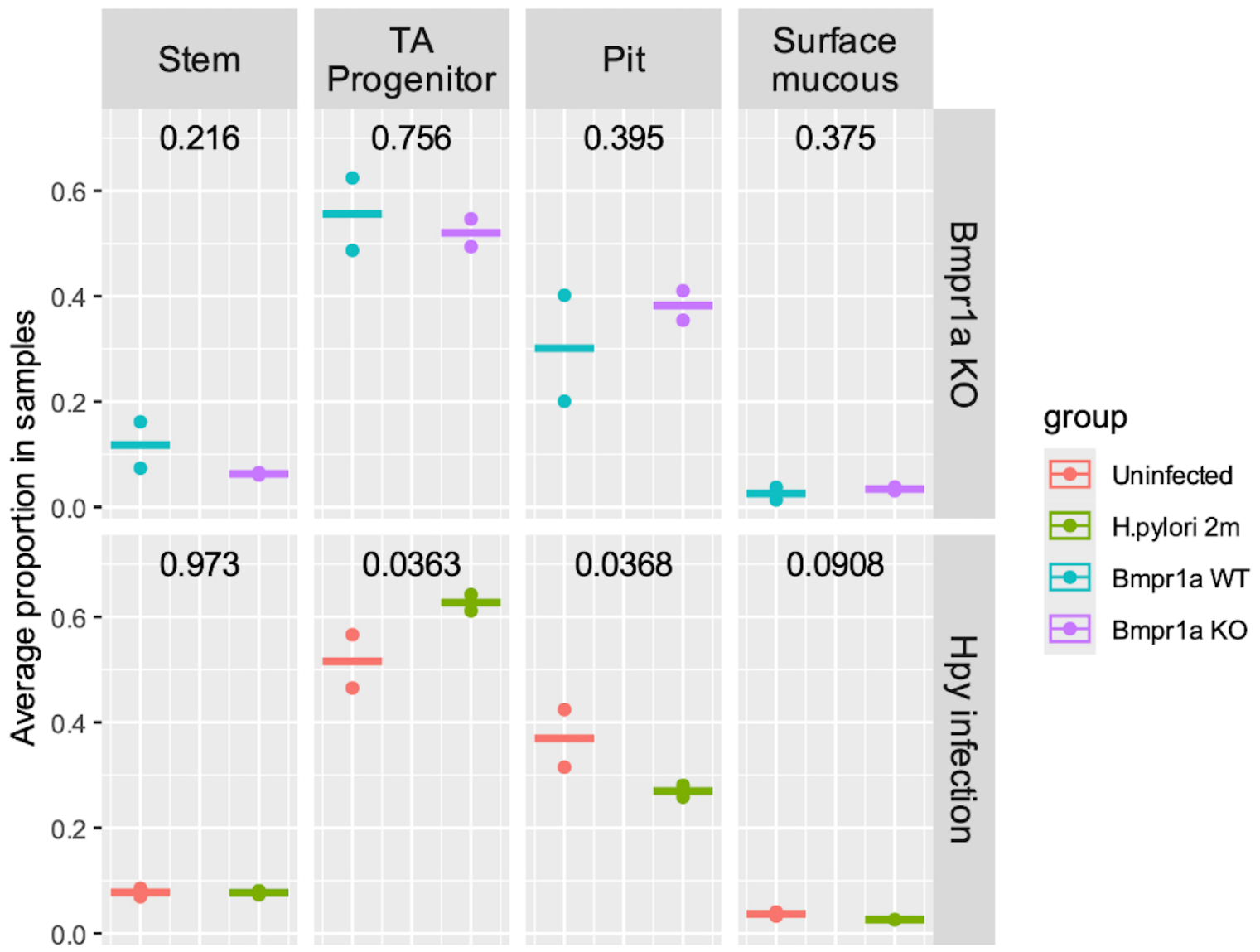

3. The current biological interpretation of the epithelial scRNA-seq dataset relies largely on predefined UCell gene signature scores and selected marker heatmaps. However, they do not fully show, from an unbiased perspective, what epithelial transcriptional programs are

most altered by H. pylori infection or Bmpr1a deletion, or which changes are shared versus distinct between these two perturbations. Since the Methods state that pseudobulk differential expression analysis was performed by aggregating counts by sample and cluster and analyzing them with DESeq2, the corresponding pseudobulk DEG results should be clearly presented in the main text or supplementary data. Specifically, the authors should provide Cluster-level or major-lineage-level DEG analyses for WT+Hp versus WT and Bmpr1a KO versus Bmpr1a WT, and complete DEG lists as supplementary tables, including gene names, log fold changes, p values, adjusted p values, and the cluster or lineage in which each comparison was performed.
We have now provided a table showing the complete DEG analysis results (Supplementary Table 2).

4. In Fig. 1, the authors show that H. pylori infection and Bmpr1a deletion are associated with reduced Lgr5 expression, increased Anxa1 expression, reduced Lgr5 ISC signature, increased regenerative stem cell signature, and increased YAP activity. In Fig. 2, the organoid data show that Bmpr1a deletion reduces Id1 expression. In Fig. 4, PGE2 treatment induces YAP target genes such as Ctgf, Ankrd1, and Cyr61. However, the current scRNA-seq presentation mainly shows selected signatures and marker heatmaps, and does not clearly demonstrate whether these same genes or pathways display concordant changes across the relevant epithelial populations and experimental conditions.
Thus, they should present condition-specific feature plots and/or violin plots for Lgr5, Anxa1, Id1, Ctgf, Ankrd1, and Cyr61 across the relevant antral epithelial populations. In addition, the authors should show whether the Lgr5 ISC signature and YAP target gene signature are consistently altered in the scRNA-seq data in a manner concordant with Fig. 1. These analyses should be presented at the level of major epithelial populations or refined antral epithelial clusters, rather than only as broad global UMAP projections. At present, the data do not clearly define an “epithelial reprogramming” state.
To further validate our bulk GSEA results, we have performed GSEA after pseudo-bulk DE analysis between conditions for each antral epithelial subset for *Lgr5*+ stem cell signature and YAP target gene signatures. We have now also provided violin plots for *Id1*, *Id2*, *Anxa1*, *Ccn1* (*Cyr61*), *Ccn2* (*Ctgf*), and *Lgr5*, while *Ankrd1* was not detectable in our scRNA-seq dataset:
(ll. 167-192) “As expected, BMP target genes *Id1* and *Id2* were downregulated in *Bmpr1a* KO pit and surface mucous cells, while infected pit and surface mucous cells showed decreased expression of *Id2* (**Supplementary Fig. 3a**). Notably, GSEA revealed that the regenerative stem cell signature was enriched across all antral cell types, including stem, TA/progenitor, pit, and surface mucous cells, following *Bmpr1a* KO or infection (**Supplementary Fig. 3b**). Moreover, YAP target gene signatures were enriched in most antral cell types upon *Bmpr1a* KO or infection (**Supplementary Fig. 3c-d**). Consistently, the upregulation of regeneration-associated genes (e.g., *Ly6a*) was broadly observed in antral epithelial cells for both conditions (**Supplementary Fig. 3e-f**), while *Anxa1* was significantly upregulated only in *Bmpr1a* KO TA/progenitor, pit, and surface mucous cells (**Supplementary Fig. 3e-f, g**). In addition, YAP target gene *Ccn1* (also known as *Cyr61*) was upregulated in *Bmpr1a* KO surface mucous cells as well as infected pit cells, while another YAP target, *Ccn2* (also known as *Ctgf*), was increased in *Bmpr1a* KO TA/progenitor cells (**Supplementary Fig. 3g**). These data indicate that the regenerative transcriptional responses are not confined to a single cell type but are rather broad responses involving various gland cell lineages.

We also analyzed the *Lgr5*+ stem cell signature and found that it was either unchanged (e.g., infected stem, TA/progenitor, and pit cells; *Bmpr1a* KO TA/progenitor and pit cells) or downregulated (e.g., *Bmpr1a* KO stem) in most cell types upon *Bmpr1a* KO or infection (**Supplementary Fig. 3h**). Although *Lgr5*+ stem cell signature was enriched in *Bmpr1a* KO or infected surface mucous cells (**Supplementary Fig. 3h**), actual *Lgr5* expression was barely detected in this epithelial subset. Moreover, we did not observe significant alteration in *Lgr5* expression in any infected or *Bmpr1a* KO epithelial subsets (**Supplementary Fig. 3i**). Taken together, our data suggest that rather than a Wnt-driven homeostatic stem cell program, a YAP-associated regenerative stem cell state likely plays a key role in driving the glandular hyperplasia observed in *H. pylori*-infected and BMP-deficient tissues."

SupplFigure3

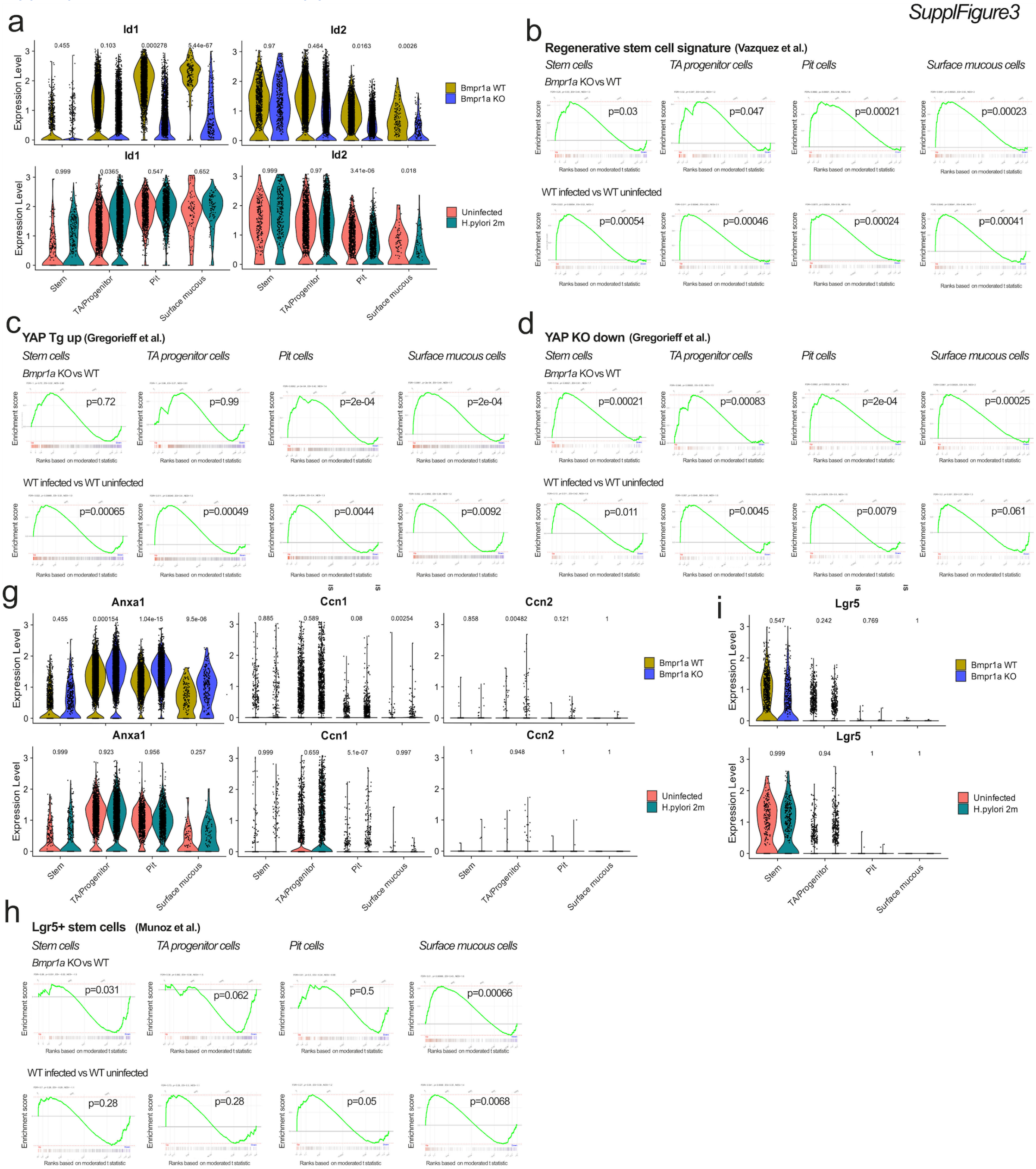

Minor Comments

Related to the point 4, I remain concerned about the use of the term "reprogramming." The

following statements should therefore be revised for consistency.
1. Title
Current wording: “Helicobacter pylori triggers gastric mucosal reprogramming into the fetal-like transcriptional state via stromal IL1-β signaling”
Suggested revision: “Helicobacter pylori triggers gastric mucosal remodeling toward a fetal-like transcriptional program via stromal IL-1β signaling”
2. Page 3, Abstract
Current wording:
“Loss of epithelial BMP signaling is sufficient to induce epithelial reprogramming in vivo via epithelial-stromal-immune crosstalk.”
Suggested revision:
“Loss of epithelial BMP signaling is sufficient to induce epithelial transcriptional changes in vivo via epithelial-stromal-immune crosstalk.”
3. Page 3, Abstract
Current wording:
“Our data provide mechanistic insights into fetal-like regenerative reprogramming…”
Suggested revision:
“Our data provide mechanistic insights into fetal-like transcriptional responses…”
4. Page 4, Introduction
Current wording:
“ultimately leading to fetal-like transcriptional reprogramming of the adjacent epithelium.”
Suggested revision:
“ultimately contributing to a fetal-like transcriptional response in the adjacent epithelium.”
5. Page 4, Introduction
Current wording:
“depletion of the IL-1 receptor in stromal cells prevents epithelial reprogramming and gland hyperplasia…”
Suggested revision:
“depletion of the IL-1 receptor in stromal cells prevents epithelial transcriptional changes and gland hyperplasia…”
6. Page 9, Results heading
Current wording:
“Stromal-derived PGE2 triggers epithelial reprogramming into the fetal-like transcriptional state”
Suggested revision:
“Stromal-derived PGE2 promotes epithelial YAP activation and a fetal-like transcriptional response”
7. Page 11, Results heading
Current wording:
“Epithelial reprogramming depends on IL-1β-driven remodeling of gastric stromal cells”
Suggested revision:
“Epithelial YAP activation and fetal-like transcriptional changes depend on IL-1β-driven remodeling of gastric stromal cells”
8. Page 13, Discussion
Current wording:
“COX2-PGE2 signaling in mesenchymal stromal cells induces YAP activation in the neighbouring epithelium and cellular reprogramming into the fetal-like transcriptional

state."
Suggested revision:
"COX2-PGE2 signaling in mesenchymal stromal cells promotes YAP activation in the neighbouring epithelium and contributes to fetal-like transcriptional changes."

We have now revised the statements accordingly.
